# Regioselective Pd-Catalyzed Hydroalkynylation of Allenyl-Containing α-Amino Acid Derivatives with Terminal Alkynes

**DOI:** 10.3390/molecules30173623

**Published:** 2025-09-04

**Authors:** Alexandra S. Bubnova, Daria V. Vorobyeva, Ivan A. Godovikov, Anna N. Philippova, Pavel S. Gribanov, Evgenia P. Antoshkina, Sergey N. Osipov

**Affiliations:** A. N. Nesmeyanov Institute of Organoelement Compounds, Russian Academy of Sciences, 28/1 Vavilova Str., 119334 Moscow, Russia; bubnova.axs@gmail.com (A.S.B.); vorobyeva-daria@yandex.ru (D.V.V.); pr0vider@ineos.ac.ru (I.A.G.); anyfil96@gmail.com (A.N.P.); gribanovps@mail.ru (P.S.G.); antoshkina.ep@phystech.edu (E.P.A.)

**Keywords:** 1,3-enynes, allenes, alkynes, hydroalkynylation, catalysis

## Abstract

An efficient access to the novel representatives of α,α-disubstituted α-amino acids with 1,3-enyne unit located at their side chain has been elaborated. The method is based on Pd(II)-catalyzed hydroalkynylation of α-allenyl-α-dimethylamino esters with terminal acetylenes. The developed strategy is the first example of the metal-catalyzed allene-alkyne coupling to provide a convenient route to new unsaturated α-amino acid derivatives in good yields and high selectivity.

## 1. Introduction

The development of facile and selective methods enabling rapid access to value complex molecules from readily available starting materials remains a major focus in modern organic synthesis [1,2,3,4,5]. In this context, conjugated 1,3-enynes have been identified as important synthetic blocks and essential units found in a variety of biologically active compounds [6,7,8,9,10,11,12]. As a consequence, the elaboration of efficient and atom-economic pathways to 1,3-enynes has been a subject of considerable interest during the past decade. Transition metal-catalyzed cross-coupling of allenes with terminal alkynes [13,14,15] has emerged as one of the most flexible synthetic routes to a variety of functionally substituted 1,3-enynes via the formation of a new C-C bond. Although several efficient protocols have been developed [16,17,18,19], control of the regio- and stereoselectivity of this transformation is often difficult to achieve due to the different reactivity modes of the unique allene system consisting two 1,2-comulative double bonds. The outcome of these reactions is greatly affected by the nature of metal species and ligand, the steric and electronic properties of the substituents, as well as their location in the allene system.

**Scheme 1:**
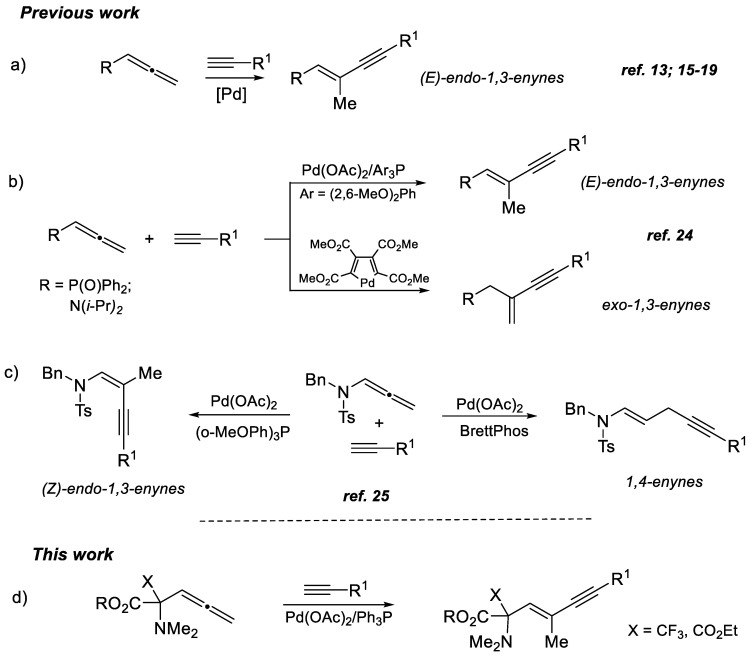
Pd-catalyzed hydroalkynylation of allenes: previous (**a**–**c**) and present work (**d**) [13,15,16,17,18,19,24,25].

While Rh- [20,21], Ru- [22] and Co- [23] catalyzed hydroalkynylation of allenes typically yields exo-1,3-enynes, Pd-catalyzed reactions result in the formation of (*E)*-endo-1,3-enynes [13,15,16,17,18,19] in a majority of published examples (Scheme 1a). However, in the case of some particular substrates, such as allenylamides and allenylphosphine oxides, capable of specific coordination with catalytically active Pd-complexes, the corresponding exo-1,3-enynes [24], (*Z*)-endo-1,3-enynes or linear 1,4-enynes [25] can also be selectively obtained depending on the choice of palladium complex (Scheme 1b) or ligand (Scheme 1c).

On the other hand, the incorporation of latent (bio)orthogonal reactive unsaturated functionalities into α-amino acid side chains not only enriches their biological properties, but also creates a universal platform for the implementation of numerous useful transformations leading to structurally diverse non-proteinogenic α-amino acids [26,27,28]. In this regard, the development of efficient synthetic protocols that would provide straightforward access to novel α-amino acids with 1,3-enyne fragments in their structure is of great importance for the sustainable drug discovery process.

Our current research program connects with the elaboration of efficient synthetic approaches to new α-amino acid derivatives through the functionalization of their unsaturated precursors under metal catalysis [29]. Recently we have revealed that readily available α-amino acid derivatives with terminal allenyl group at the side chain are unique substrates for a variety of transition metal-catalyzed transformations [30], such as hydroamination [31], C-H activation/annulation [32], and hydroarylation [33] reactions under Cu-, Rh-, and Pd-catalysis, respectively. In continuation of this study, herein we want to disclose a palladium-catalyzed regioselective hydroalkynylation of allenyl α-amino acid derivatives with different terminal alkynes as a simple and robust route to a new family of α,α-disubstituted α-amino acids bearing a 1,3-enyne moiety at their side chains (Scheme 1d). To the best of our knowledge allenyl-containing α-amino acids have not been applied in this catalytic process before.

## 2. Results

Given the fact that many α-amino acids bearing fluoroalkyl groups at the α-position can act as selective inhibitors of pyridoxal phosphate-dependent enzymes, often exhibiting a number of interesting pharmacological properties [34,35,36], we commenced our study with the model reaction of readily available CF_3_-containing α-allenyl-α-amino carboxylate **1a [37]** with phenyl acetylene. Palladium acetate/phosphine mixture was selected as the most competent catalytic system to initiate this process. Initially, we found that the reaction takes place at the presence of 5 mol% Pd(OAc)_2_ and 10 mol% of PPh_3_ under heating in toluene at 60 °C, affording the corresponding α-amino acid derivative **3a** in 80% NMR yield after 4 h, along with noticeable amount of starting allene **1a** (Table 1, entry 1). No reaction occurs at ambient temperature.

The isolated product was identified as (*E)*-endo-1,3-enyne-containing *N,N*-dimethyl-α-amino ester **3a** using standard physicochemical methods including 2D ^1^H-^19^F HOESY NMR spectroscopy (see Supporting Info). The utilization of 1,4-dioxane instead of toluene as a solvent slightly increased the conversion of **1a**. The further improvement was achieved by changing PPh_3_ ligand for dppf (entry 3). Other screened phosphines, such as Xantphos and (4-CF_3_-Ph)_3_P, did not improve the conversion of starting allene (entries 4 and 5). It is noteworthy that no products were detected in the reaction mixture with the presence of BrettPhos ligand (entry 6) as well as under catalysis with PdCl_2_(Ph_3_P)_2_ complex (entry 7). Then, it was revealed that the decrease in Pd(OAc)_2_ loading to 2 mol% almost did not affect the outcome of the process. Consequently, the following optimal conditions for the reaction were established: the heating of the allene with a small excess of acetylene in dioxane in the presence of 2 mol% Pd(OAc)_2_ and 4 mol% PPh_3_ at 55 °C for 4 h. The preference for PPh_3_ is due to its low cost and small difference in activity compared to the dppf ligand. It should be noted that the reaction conditions found require the use of 2.5 times less palladium catalyst compared to a similar process described earlier for the alkynylation of allene derivatives [18]. The further reduction in catalyst loading (to 1 mol%) significantly increased the reaction time (entries 9 and 11). In addition, the last experiment was performed in 2-MeTHF, affording the product in good yield for 4 h (entry 13), demonstrating a possibility to use green solvent for the process.

**Scheme 2:**
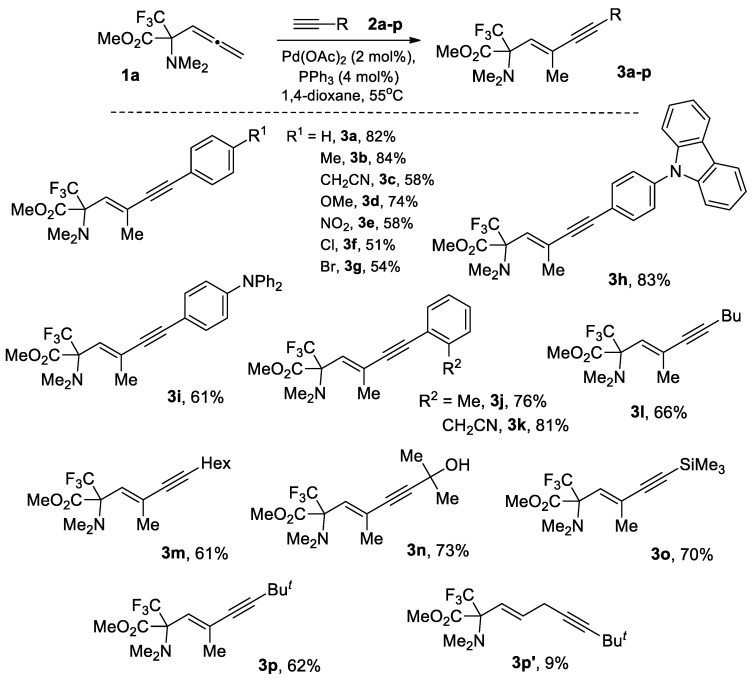
Synthesis of α-CF_3_-α-amino acid derivatives **3**.

With these conditions in hand, the scope of the alkynylation of allene **1a** with respect to different aryl and alkyl acetylene substrates **2a-p** was investigated (Scheme 2). As a result, it was found that the electronic and steric nature of substituents in terminal aryl(alkyl) acetylene does not significantly affect the yield of the corresponding (*E)*-endo-1,3-enyne-containing α-amino acid derivatives **3a-p**, which were the sole products of allene *β*-alkynylation reaction. However, in the case of bulky *t*-Bu acetylene, the conversion of the reaction with allene **1a** did not exceed 50% even under heating a mixture of the reagents at 80 °C for 8 h. This problem was successfully solved by replacing the PPh_3_ ligand with dppf and maintaining the reaction mixture at an elevated temperature (80 °C) for 4 h, which allowed achieving almost full conversion of **1a** and a good yield of **3p** (79%). However, in this case, insignificant amount of 1,4-enyne **3p’** was isolated from the reaction mixture as a result of γ-alkynylation by-process.

**Scheme 3:**
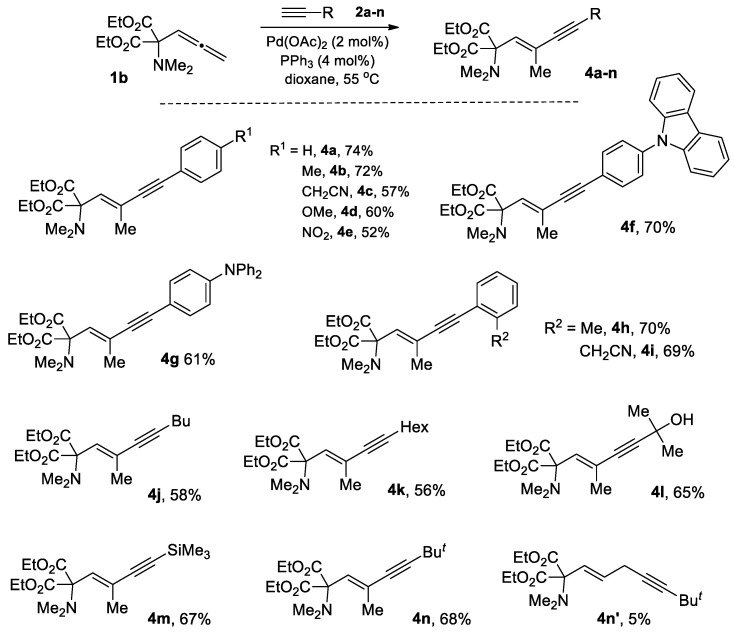
Hydroalkynylation of allene **1b**.

To expand the scope of the hydroalkynylation reaction, we investigated the reactivity of allene **1b** [30] using the same conditions found for CF_3_-allene **1a**. As it turned out, these conditions are also well-suited for the reactions of **1b** with terminal alkynes. Thus, a series of new α-amino acid derivatives **4a-n** (Scheme 3) decorated with 1,3-enyne moiety were obtained in moderate to good yields with high degree of regio- and stereoselectivity. No alternative isomers were detected in all studied reactions, except the only example with bulky *t*-Bu acetylene. As it was for fluorinated allene **1a**, the reaction of **1b** with this alkyne was accomplished under heating at 80 °C for 6 h to reach an acceptable conversion of **1b**, yielding the desired product **4n** along with insignificant admixture of 1,4-enyne **4n’** (see Supplementary Materials).

**Scheme 4:**
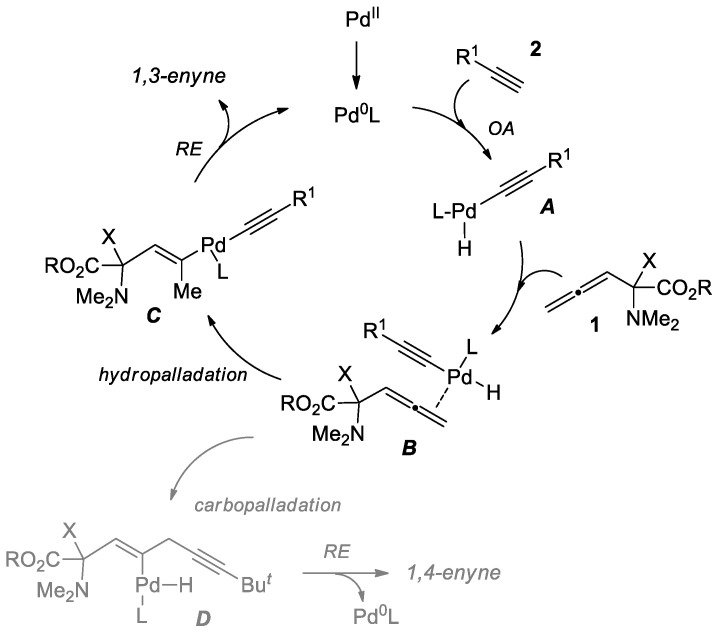
Plausible mechanism of hydroalkynylation of allene **1**.

Based on the literature examples described for Pd-catalyzed hydroalkynylation of allenes [13,14,15,16,17,18,19,25] and the experimental data obtained, plausible mechanism for the reaction of allene **1** with terminal acetylene is depicted in Scheme 4. After oxidative addition (OA) step to obtain intermediate complex **A**, the coordination allene **1** with **A** occurs to form **B**. The latter undergoes hydropalladation leading to complex **C**. Finally, the reductive elimination (RE) of **C** gives the 1,3-enyne product, liberating an active Pd species for the next catalytic cycle. The formation of minor 1,4-enyne in the case of *t*-Bu acetylene can likely be rationalized by the realization of a less favorable carbopalladation process via intermediate **D** (Scheme 4).

**Scheme 5:**
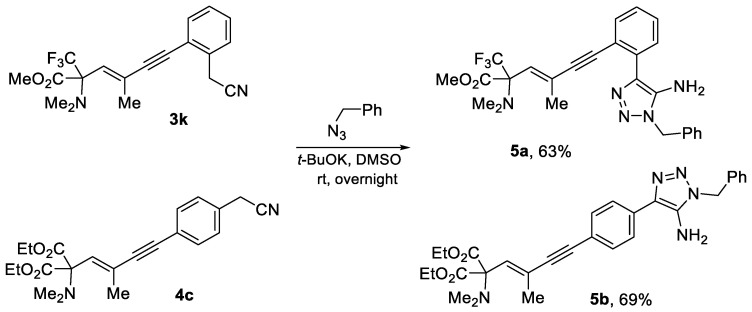
Synthesis of 5-amino-1,2,3-triazoles **5a,b** by nitrile–azide coupling.

Furthermore, 1,3-enyne α-amino esters **3k** and **4c** containing CH_2_-CN group can be successfully utilized for the installation of an additional pharmacophore unit via well-established nitrile–azide coupling [38] to afford the corresponding 5-amino-1,2,3-triazole derivatives **5a,b** in acceptable yields (Scheme 5).

## 3. Materials and Methods

### 3.1. General Information

All the reactions were carried out under argon atmosphere, and the solvents were distilled from appropriate drying agents prior to use. All reagents were used as purchased from Sigma-Aldrich (Munich, Germany). Analytical TLC was performed with Merck silica gel 60 F 254 plates (Darmstadt, Germany); visualization was accomplished with UV light, iodine vapors or Ce(SO_4_)_2_ solution in 5% H_2_SO_4_. Chromatography was carried out using Merck silica gel (Kieselgel 60, 0.063–0.200 mm, Darmstadt, Germany) and petroleum ether/ethyl acetate as an eluent. NMR spectra were obtained with Bruker AV-300 (Karlsruhe, Germany) and Inova-400 (Varian, Palo Alto, CA, USA) spectrometers operating at 300 and 400 MHz, respectively, for ^1^H (TMS reference), at 101 MHz for ^13^C, and at 282 and 376 MHz for ^19^F (CCl_3_F reference). High-resolution mass spectra were recorded on a LCMS-9030 device (Shimadzu, Japan) by electrospray ionization mass spectrometry (ESI-MS). Measurements were carried out in positive ion mode; samples were dissolved in acetonitrile and injected into the mass-spectrometer chamber from an HPLC system LC-40 Nexera (Shimadzu, Japan).

### 3.2. General Procedure for Hydroalkynylation of Allenes with Terminal Alkynes

Under argon in a Schlenk tube with a magnetic stirring bar, corresponding allene (100 mg, 0.44 mmol, 1.0 equiv.) and corresponding alkyne (0.54 mmol, 1.2 equiv.) were dissolved in dry 1,4-dioxane (2 mL). Then, Pd(OAc)_2_ (2 mg, 9 μmol, 2 mol%) and PPh_3_ (4.7 mg, 18 μmol, 4 mol%) were added, and the reaction mixture was stirred at 55 °C for 4–8 h until the completion of the reaction monitored by TLC and ^19^F-NMR. The reaction mixture was cooled to room temperature and concentrated under reduced pressure. Purification by chromatography (gradient elution: petroleum ether/dichloromethane = 1:1, petroleum ether/ethyl acetate = 15:1) gave analytically pure desired product.

### 3.3. General Procedure for Preparation of 5-Amino-1,2,3-Triazole Derivatives ***5a*** and ***5b*** via Dipolar Azide–Nitrile Cycloaddition (DCR)

5-amino-1,2,3-triazole derivatives **5a** and **5b** were synthesized according to literature procedure [39] with minor modifications. A screw-cap vial equipped with a magnetic stir bar was charged with 0.2 mmol of **3k** or **4c**, 3.0 equiv. of benzyl azide, DMSO (4 mL), and 0.5 equiv. of powdered potassium tert-butoxide. The reaction mixture was allowed to stir for 24 h at room temperature. Upon completion, the mixture was poured into water and extracted with dichloromethane. The combined organic phases were washed with brine, dried over MgSO_4_, filtered, and concentrated under reduced pressure. Purification by chromatography (eluent–hexane: ethyl acetate 1:1) gave analytically pure desired product.

### 3.4. Characterization Data of New Compounds

*(E)-Methyl 2-(dimethylamino)-4-methyl-6-phenyl-2-(trifluoromethyl)hex-3-en-5-ynoate* (**3a**). Yield 82% (110.7 mg) as a pale yellow thick oil. ^1^H-NMR (400 MHz, CDCl_3_) *δ* 7.45–7.42 (m, 2H, Ar), 7.31–7.30 (m, 3H, Ar), 5.97 (s, 1H, CH), 3.80 (s, 3H, OCH_3_), 2.52 (s, 6H, N(CH_3_)_2_), 2.11 (s, 3H, CH_3_). ^13^C-NMR (101 MHz, CDCl_3_) *δ* 167.3, 131.6, 128.5, 128.4, 128.3, 127.5, 125.8 (q, *J* = 297.0 Hz, CF_3_), 122.8, 91.5, 88.7, 73.8 (q, *J* = 24.0 Hz, >*C*<), 52.7, 39.9, 18.6. ^19^F-NMR (376 MHz, CDCl_3_) *δ* -66.70 (s, 3F, CF_3_). HRMS (ESI): *m*/*z* calcd. for C_17_H_19_F_3_NO_2_ [M + H]^+^ 326.1362, found 326.1362.

*(E)-Methyl 2-(dimethylamino)-4-methyl-6-p-tolyl-2-(trifluoromethyl)hex-3-en-5-ynoate* (**3b**). Yield 84% (83.7 mg) as a pale yellow thick oil. ^1^H-NMR (400 MHz, CDCl_3_) *δ* 7.32 (d, *J* = 8.0 Hz, 2H, Ar), 7.11 (d, *J* = 7.8 Hz, 2H, Ar), 5.93 (s, 1H, CH), 3.79 (s, 3H, OCH_3_), 2.51 (s, 6H, N(CH_3_)_2_), 2.33 (s, 3H, CH_3_), 2.08 (s, 3H, CH_3_). ^13^C-NMR (101 MHz, CDCl_3_) *δ* 167.3, 138.6, 131.5, 129.1, 128.6, 127.1, 125.8 (q, *J* = 297.1 Hz, CF_3_), 119.7, 90.9, 88.9, 73.8 (q, *J* = 24.3 Hz, >*C*<), 52.7, 40.0, 21.4, 18.7. ^19^F-NMR (376 MHz, CDCl_3_) *δ* -66.72 (s, 3F, CF_3_). HRMS (ESI): *m*/*z* calcd. for C_18_H_21_F_3_NO_2_ [M + H]^+^ 340.1519, found 340.1522.

*(E)-Methyl 6-(4-(cyanomethyl)phenyl)-2-(dimethylamino)-4-methyl-2-(trifluoromethyl) hex-3-en-5-ynoate* (**3c**). Yield 58% (57.5 mg) as a pale yellow thick oil. ^1^H-NMR (400 MHz, CDCl_3_) *δ* 7.43 (d, *J* = 8.1 Hz, 2H, Ar), 7.27 (d, *J* = 8.1 Hz, 2H, Ar), 5.96 (s, 1H, CH), 3.79 (s, 3H, OCH_3_), 3.73 (s, 2H, CH_2_), 2.50 (s, 6H, N(CH_3_)_2_), 2.08 (s, 3H, CH_3_). ^13^C-NMR (101 MHz, CDCl_3_) *δ* 167.2, 132.2, 130.1, 128.3, 128.0, 127.9, 125.7 (q, *J* = 296.9 Hz, CF_3_), 122.8, 117.3, 92.3, 87.7, 73.8 (q, *J* = 24.0 Hz, >*C*<), 52.7, 40.0, 23.5, 18.6. ^19^F-NMR (376 MHz, CDCl_3_) *δ* -66.72 (s, 3F, CF_3_). HRMS (ESI): *m*/*z* calcd. for C_19_H_20_F_3_N_2_O_2_ [M + H]^+^ 365.1471, found 365.1477.

*(E)-Methyl 2-(dimethylamino)-6-(4-methoxyphenyl)-4-methyl-2-(trifluoromethyl)hex-3-en- 5-ynoate* (**3d**). Yield 74% (91.2 mg) as a pale yellow thick oil. ^1^H-NMR (400 MHz, CDCl_3_) *δ* 7.36 (d, *J* = 8.8 Hz, 2H, Ar), 6.82 (d, *J* = 8.8 Hz, 2H, Ar), 5.91 (s, 1H, CH), 3.79 (s, 6H, 2OCH_3_), 2.50 (s, 6H, N(CH_3_)_2_), 2.08 (s, 3H, CH_3_). ^13^C-NMR (101 MHz, CDCl_3_) *δ* 167.3, 159.7, 133.0, 128.7, 126.7, 125.8 (q, *J* = 297.0 Hz, CF_3_), 114.8, 113.9, 90.3, 88.7, 73.8 (q, *J* = 24.1 Hz, >*C*<), 55.2, 52.6, 39.9, 18.7. ^19^F-NMR (376 MHz, CDCl_3_) *δ* -66.73 (s, 3F, CF_3_). HRMS (ESI): *m*/*z* calcd. for C_18_H_21_F_3_NO_3_ [M + H]^+^ 356.1468, found 356.1471.

*(E)-Methyl 2-(dimethylamino)-4-methyl-6-(4-nitrophenyl)-2-(trifluoromethyl)hex-3-en-5- ynoate* (**3e**). Yield 58% (67.3 mg) as a pale yellow thick oil. ^1^H-NMR (400 MHz, CDCl_3_) *δ* 8.17 (d, *J* = 7.2 Hz, 2H, Ar), 7.56 (d, *J* = 8.0 Hz, 2H, Ar), 6.03 (s, 1H, CH), 3.81 (s, 3H, OCH_3_), 2.50 (s, 6H, N(CH_3_)_2_), 2.11 (s, 3H, CH_3_). ^13^C-NMR (101 MHz, CDCl_3_) *δ* 167.0, 147.1, 132.3, 129.7, 129.6, 127.8, 125.6 (q, *J* = 296.8 Hz, CF_3_), 123.6, 96.4, 86.6, 73.8 (q, *J* = 23.9 Hz, >*C*<), 52.8, 40.0, 18.4. ^19^F-NMR (376 MHz, CDCl_3_) *δ* -66.68 (s, 3F, CF_3_). HRMS (ESI): *m*/*z* calcd. for C_17_H_18_F_3_N_2_O_4_ [M + H]^+^ 371.1213, found 371.1224.

*(E)-Methyl 6-(4-chlorophenyl)-2-(dimethylamino)-4-methyl-2-(trifluoromethyl)hex-3-en-5- ynoate* (**3f**). Yield 51% (52.6 mg) as a pale yellow thick oil. ^1^H-NMR (400 MHz, CDCl_3_) *δ* 7.35 (d, *J* = 8.5 Hz, 2H, Ar), 7.28 (d, *J* = 8.4 Hz, 2H, Ar), 5.95 (s, 1H, CH), 3.80 (s, 3H, OCH_3_), 2.50 (s, 6H, N(CH_3_)_2_), 2.08 (s, 3H, CH_3_). ^13^C-NMR (101 MHz, CDCl_3_) *δ* 167.2, 134.5, 132.8, 128.6, 128.3, 127.9, 125.7 (q, *J* = 297.0 Hz, CF_3_), 121.3, 92.4, 87.5, 73.8 (q, *J* = 24.2 Hz, >*C*<), 52.7, 40.0, 18.5. ^19^F-NMR (376 MHz, CDCl_3_) *δ* -66.71 (s, 3F, CF_3_). HRMS (ESI): *m*/*z* calcd. for C_17_H_18_ClF_3_NO_2_ [M + H]^+^ 360.0973, found 360.0971.

*(E)-Methyl 6-(4-bromophenyl)-2-(dimethylamino)-4-methyl-2-(trifluoromethyl) hex-3-en-5- ynoate* (**3g**). Yield 54% (99.2 mg) as a pale yellow thick oil. ^1^H-NMR (400 MHz, CDCl_3_) *δ* 7.43 (d, *J* = 8.5 Hz, 2H, Ar), 7.28 (d, *J* = 8.5 Hz, 2H, Ar), 5.96 (s, 1H, CH), 3.80 (s, 3H, OCH_3_), 2.50 (s, 6H, N(CH_3_)_2_), 2.08 (s, 3H, CH_3_). ^13^C-NMR (101 MHz, CDCl_3_) *δ* 167.2, 132.9, 131.6, 128.3, 127.9, 125.7 (q, *J* = 296.9 Hz, CF_3_), 122.7, 121.7, 92.5, 87.5, 73.8 (q, *J* = 24.1 Hz, >*C*<), 52.7, 40.0, 18.5. ^19^F-NMR (376 MHz, CDCl_3_) *δ* -66.70 (s, 3F, CF_3_). HRMS (ESI): *m*/*z* calcd. for C_17_H_17_BrF_3_NO_2_ [M]^+^ 404.0468, found 404.0473.

*(E)-Methyl 6-(4-(9H-carbazol-9-yl)phenyl)-2-(dimethylamino)-4-methyl-2-(trifluoro methyl)hex-3-en-5-ynoate* (**3h**). Yield 83% (118.7 mg) as a pale yellow thick oil. ^1^H-NMR (400 MHz, CDCl_3_) *δ* 8.13 (d, *J* = 7.7 Hz, 2H, Ar), 7.66 (d, *J* = 8.3 Hz, 2H, Ar), 7.54 (d, *J* = 8.3 Hz, 2H, Ar), 7.44–7.39 (m, 4H, Ar), 7.31–7.27 (m, 2H, Ar), 6.03 (s, 1H, CH), 3.84 (s, 3H, OCH_3_), 2.55 (s, 6H, N(CH_3_)_2_), 2.15 (s, 3H, CH_3_). ^13^C-NMR (101 MHz, CDCl_3_) *δ* 167.3, 140.5, 137.7, 133.1, 128.4, 127.9, 126.8, 126.0, 125.8 (q, *J* = 297.1 Hz, CF_3_), 123.5, 121.7, 120.3, 120.2, 109.7, 92.4, 87.9, 73.7, 52.8, 40.1, 18.7. ^19^F-NMR (376 MHz, CDCl_3_) *δ* -66.66 (s, 3F, CF_3_). HRMS (ESI): *m*/*z* calcd. for C_29_H_26_F_3_N_2_O_2_ [M + H]^+^ 491.1936, found 491.1941.

*(E)-Methyl 2-(dimethylamino)-6-(4-(diphenylamino)phenyl)-4-methyl-2-(trifluoromethyl) hex-3-en-5-ynoate* (**3i**). Yield 61% (66.5 mg) as a pale yellow thick oil. ^1^H-NMR (400 MHz, CDCl_3_) *δ* 7.28–7.24 (m, 6H, Ar), 7.10–7.03 (m, 6H, Ar), 6.96 (d, *J* = 8.5 Hz, 2H, Ar), 5.91 (s, 1H, CH), 3.80 (s, 3H, OCH_3_), 2.52 (s, 6H, N(CH_3_)_2_), 2.08 (s, 3H, CH_3_). ^13^C-NMR (101 MHz, CDCl_3_) *δ* 167.3, 148.1, 147.1, 132.5, 129.4, 128.7, 126.7, 125.8 (q, *J* = 297.1 Hz, CF_3_), 125.0, 123.6, 122.0, 115.4, 90.9, 89.0, 73.8 (q, *J* = 24.7 Hz, >*C*<), 52.7, 40.0, 18.7. ^19^F-NMR (376 MHz, CDCl_3_) *δ* -66.69 (s, 3F, CF_3_). HRMS (ESI): *m*/*z* calcd. for C_29_H_27_F_3_N_2_O_2_ [M]^+^ 492.2019, found 492.2021.

*(E)-Methyl 2-(dimethylamino)-4-methyl-6-o-tolyl-2-(trifluoromethyl)hex-3-en-5-ynoate* (**3j**). Yield 76% (86.1 mg) as a pale yellow thick oil. ^1^H-NMR (400 MHz, CDCl_3_) *δ* 7.40 (d, *J* = 7.5 Hz, 1H, Ar), 7.23–7.18 (m, 2H, Ar), 7.15–7.11 (m, 1H, Ar), 5.95 (s, 1H, CH), 3.81 (s, 3H, OCH_3_), 2.53 (s, 6H, N(CH_3_)_2_), 2.43 (s, 3H, CH_3_), 2.12 (s, 3H, CH_3_). ^13^C-NMR (101 MHz, CDCl_3_) *δ* 167.3, 140.2, 131.9, 129.4, 128.7, 128.5, 126.9, 125.8 (q, *J* = 297.0 Hz, CF_3_), 125.5, 122.5, 95.5, 87.7, 73.8 (q, *J* = 24.1 Hz, >*C*<), 52.7, 40.0, 20.6, 18.8. ^19^F-NMR (376 MHz, CDCl_3_) *δ* -66.71 (s, 3F, CF_3_). HRMS (ESI): *m*/*z* calcd. for C_18_H_21_F_3_NO_2_ [M + H]^+^ 340.1519, found 340.1518.

*(E)-Methyl 6-(2-(cyanomethyl)phenyl)-2-(dimethylamino)-4-methyl-2-(trifluoromethyl) hex-3-en-5-ynoate* (**3k**). Yield 81% (78.7 mg) as a pale yellow thick oil. ^1^H-NMR (400 MHz, CDCl_3_) *δ* 7.47–7.45 (m, 2H, Ar), 7.36–7.27 (m, 2H, Ar), 5.98 (s, 1H, CH), 3.86 (s, 2H, CH_2_), 3.81 (s, 3H, OCH_3_), 2.51 (s, 6H, N(CH_3_)_2_), 2.12 (s, 3H, CH_3_). ^13^C-NMR (101 MHz, CDCl_3_) *δ* 167.1, 132.4, 131.7, 129.2, 128.4, 128.1, 127.9, 125.6 (q, *J* = 296.7 Hz, CF_3_), 122.3, 117.2, 97.6, 85.3, 73.8 (q, *J* = 24.3 Hz, >*C*<), 52.8, 40.0, 22.7, 18.6. ^19^F-NMR (376 MHz, CDCl_3_) *δ* -66.70 (s, 3F, CF_3_). HRMS (ESI): *m*/*z* calcd. for C_19_H_20_F_3_N_2_O_2_ [M + H]^+^ 365.1471, found 365.1472.

*(E)-Methyl 2-(dimethylamino)-4-methyl-2-(trifluoromethyl)dec-3-en-5-ynoate* (**3l**). Yield 66% (72.2 mg) as a pale yellow thick oil. ^1^H-NMR (400 MHz, CDCl_3_) *δ* 5.72 (s, 1H, CH), 3.75 (s, 3H, OCH_3_), 2.45 (s, 6H, N(CH_3_)_2_), 2.26 (t, *J* = 7.0 Hz, 2H, CH_2_), 1.93 (s, 3H, CH_3_), 1.51–1.44 (m, 2H, CH_2_), 1.42–1.33 (m, 2H, CH_2_), 0.88 (t, *J* = 7.2 Hz, 3H, CH_3_). ^13^C-NMR (101 MHz, CDCl_3_) *δ* 167.4, 128.9, 125.9, 125.8 (q, *J* = 297.0 Hz, CF_3_), 89.8, 82.9, 73.6 (q, *J* = 24.0 Hz, >*C*<), 52.5, 39.9, 30.6, 21.9, 18.9, 18.8, 13.5. ^19^F-NMR (376 MHz, CDCl_3_) *δ* -66.91 (s, 3F, CF_3_). HRMS (ESI): *m*/*z* calcd. for C_15_H_23_F_3_NO_2_ [M + H]^+^ 306.1676, found: 306.1682.

*(E)-Methyl 2-(dimethylamino)-4-methyl-2-(trifluoromethyl)dodec-3-en-5-ynoate* (**3m**). Yield 61% (63.8 mg) as a pale yellow thick oil. ^1^H-NMR (400 MHz, CDCl_3_) *δ* 5.74 (s, 1H, CH), 3.77 (s, 3H, OCH_3_), 2.46 (s, 6H, N(CH_3_)_2_), 2.26 (t, *J* = 7.1 Hz, 2H, CH_2_), 1.95 (s, 3H, CH_3_), 1.54–1.47 (m, 2H, CH_2_), 1.40–1.33 (m, 2H, CH_2_), 1.30–1.24 (m, 4H, CH_2_), 0.87 (t, *J* = 6.8 Hz, 3H, CH_3_). ^13^C-NMR (101 MHz, CDCl_3_) *δ* 167.4, 128.9, 125.9, 125.8 (q, *J* = 297.1 Hz, CF_3_), 89.9, 82.9, 73.6 (q, *J* = 23.9 Hz, >*C*<), 52.6, 39.9, 31.3, 28.5, 22.5, 19.2, 18.9, 13.9. ^19^F-NMR (376 MHz, CDCl_3_) *δ* -66.87 (s, 3F, CF_3_). HRMS (ESI): *m*/*z* calcd. for C_17_H_27_F_3_NO_2_ [M + H]^+^ 334.1988, found 334.1988.

*(E)-Methyl 2-(dimethylamino)-7-hydroxy-4,7-dimethyl-2-(trifluoromethyl)oct-3-en-5- ynoate* (**3n**). Yield 73% (81.4 mg) as a pale yellow thick oil. ^1^H-NMR (400 MHz, CDCl_3_) *δ* 5.77 (s, 1H, CH), 3.75 (s, 3H, OCH_3_), 2.44 (s, 6H, N(CH_3_)_2_), 2.29 (s, 1H, OH), 1.94 (s, 3H, CH_3_), 1.49 (s, 6H, 2CH_3_). ^13^C-NMR (101 MHz, CDCl_3_) *δ* 167.2, 128.0, 127.4, 125.7 (q, *J* = 296.9 Hz, CF_3_), 93.0, 84.2, 73.6 (q, *J* = 24.0 Hz, >*C*<), 65.3, 52.6, 39.9, 31.3, 18.6. ^19^F-NMR (376 MHz, CDCl_3_) *δ* -66.83 (s, 3F, CF_3_). HRMS (ESI): *m*/*z* calcd. for C_14_H_21_F_3_NO_3_ [M + H]^+^ 308.1468, found: 308.1468.

*(E)-Methyl 2-(dimethylamino)-4-methyl-2-(trifluoromethyl)-6-(trimethylsilyl)hex-3-en-5- ynoate* (**3o**). Yield 70% (58.6 mg) as a pale yellow thick oil. ^1^H-NMR (400 MHz, CDCl_3_) *δ* 5.87 (s, 1H, CH), 3.76 (s, 3H, OCH_3_), 2.46 (s, 6H, N(CH_3_)_2_), 1.97 (s, 3H, CH_3_), 0.16 (s, 9H, 3CH_3_). ^13^C-NMR (101 MHz, CDCl_3_) *δ* 167.1, 128.5, 128.2, 125.7 (q, *J* = 297.0 Hz, CF_3_), 107.0, 93.3, 73.7 (q, *J* = 24.0 Hz, >*C*<), 52.6, 39.9, 18.5, -0.2. ^19^F-NMR (376 MHz, CDCl_3_) *δ* -66.76 (s, 3F, CF_3_). HRMS (ESI): *m*/*z* calcd. for C_14_H_23_F_3_NO_2_Si [M + H]^+^ 322.1445, found: 322.1443.

*(E)-Methyl 2-(dimethylamino)-4,7,7-trimethyl-2-(trifluoromethyl)oct-3-en-5-ynoate* (**3p**). Yield 62% (52.4 mg) as a pale yellow thick oil. ^1^H-NMR (400 MHz, CDCl_3_) *δ* 5.70 (s, 1H, CH), 3.77 (s, 3H, OCH_3_), 2.46 (s, 6H, N(CH_3_)_2_), 1.94 (s, 3H, CH_3_), 1.21 (s, 9H, CH_3_). ^13^C-NMR (101 MHz, CDCl_3_) *δ* 167.4, 129.0, 125.8 (q, *J* = 297.1 Hz, CF_3_), 125.5, 97.8, 81.4, 73.6 (q, *J* = 24.2 Hz, >*C*<), 52.5, 39.9, 30.9, 27.7, 19.1. ^19^F-NMR (376 MHz, CDCl_3_) *δ* -66.80 (s, 3F, CF_3_). HRMS (ESI): *m*/*z* calcd. for C_15_H_23_F_3_NO_2_ [M + H]^+^ 306.1675, found: 306.1673.

*Methyl 2-(dimethylamino)-7,7-dimethyl-4-methylene-2-(trifluoromethyl)oct-5-ynoate* (**3p’**). Yield 9% (7.5 mg) as a pale yellow thick oil. ^1^H-NMR (300 MHz, CDCl_3_) *δ* 5.96–5.93 (m, 2H, 2CH), 3.85 (s, 3H, OCH_3_), 3.01 (d, *J* = 3.1 Hz, 2H, CH_2_), 2.52 (s, 6H, N(CH_3_)_2_), 1.23 (s, 9H, 3CH_3_). ^19^F-NMR (282 MHz, CDCl_3_) *δ* -66.65 (s, 3F, CF_3_). HRMS (ESI): *m*/*z* calcd. for C_15_H_23_F_3_NO_2_ [M + H]^+^ 306.1675, found: 306.1674.

*(E)-Diethyl 2-(dimethylamino)-2-(2-methyl-4-phenylbut-1-en-3-ynyl)malonate* (**4a**). Yield 74% (76.5 mg) as a pale yellow thick oil. ^1^H-NMR (400 MHz, CDCl_3_) *δ* 7.42–7.40 (m, 2H, Ar), 7.29–7.27 (m, 3H, Ar), 6.29 (s, 1H, CH), 4.26–4.20 (m, 4H, 2OCH_2_), 2.37 (s, 6H, N(CH_3_)_2_), 2.02 (s, 3H, CH_3_), 1.26 (t, *J* = 7.1 Hz, 6H, 2CH_3_). ^13^C-NMR (101 MHz, CDCl_3_) *δ* 167.5, 131.9, 131.5, 128.2, 128.1, 125.7, 123.1, 92.1, 87.8, 75.1, 61.3, 40.5, 18.6, 14.2. HRMS (ESI): *m*/*z* calcd. for C_20_H_26_NO_4_ [M + H]^+^ 344.1856, found 344.1854.

*(E)-Diethyl 2-(dimethylamino)-2-(2-methyl-4-p-tolylbut-1-en-3-ynyl)malonate* (**4b**). Yield 72% (72 mg) as a pale yellow thick oil. ^1^H-NMR (300 MHz, CDCl_3_) *δ* 7.35 (d, *J* = 8.1 Hz, 2H, Ar), 7.13 (d, *J* = 7.9 Hz, 2H, Ar), 6.30 (s, 1H, CH), 4.31–4.24 (m, 4H, 2OCH_2_), 2.41 (s, 6H, N(CH_3_)_2_), 2.36 (s, 3H, CH_3_), 2.05 (s, 3H, CH_3_), 1.31 (t, *J* = 7.1 Hz, 6H, 2CH_3_). ^13^C-NMR (101 MHz, CDCl_3_) *δ* 167.5, 138.2, 131.5, 131.4, 128.9, 125.8, 120.0, 91.5, 88.0, 75.1, 61.3, 40.4, 21.4, 18.6, 14.2. HRMS (ESI): *m*/*z* calcd. for C_21_H_28_NO_4_ [M + H]^+^ 358.2013, found 358.2007.

*(E)-Diethyl 2-(4-(4-(cyanomethyl)phenyl)-2-methylbut-1-en-3-ynyl)-2-(dimethylamino)malonate* (**4c**). Yield 57% (72.3 mg) as a pale yellow thick oil. ^1^H-NMR (300 MHz, CDCl_3_) *δ* 7.47 (d, *J* = 8.3 Hz, 2H, Ar), 7.31–7.28 (m, 2H, Ar), 6.35 (s, 1H, CH), 4.32–4.24 (m, 4H, 2OCH_2_), 3.77 (s, 2H, CH_2_), 2.42 (s, 6H, N(CH_3_)_2_), 2.06 (s, 3H, CH_3_), 1.31 (t, *J* = 7.1 Hz, 6H, 2CH_3_). ^13^C-NMR (101 MHz, CDCl_3_) *δ* 167.4, 132.4, 132.1, 129.8, 127.9, 125.4, 123.1, 117.4, 92.9, 86.9, 75.1, 61.3, 40.4, 23.4, 18.5, 14.2. HRMS (ESI): *m*/*z* calcd. for C_22_H_27_N_2_O_4_ [M + H]^+^ 383.1965, found 383.1966.

*(E)-Diethyl 2-(dimethylamino)-2-(4-(4-methoxyphenyl)-2-methylbut-1-en-3-ynyl)malonate* (**4d**). Yield 60% (65.7 mg) as a pale yellow thick oil. ^1^H-NMR (400 MHz, CDCl_3_) *δ* 7.35 (d, *J* = 8.9 Hz, 2H, Ar), 6.81 (d, *J* = 8.9 Hz, 2H, Ar), 6.24 (s, 1H, CH), 4.26–4.20 (m, 4H, 2OCH_2_), 3.78 (s, 3H, OCH_3_), 2.37 (s, 6H, N(CH_3_)_2_), 2.00 (s, 3H, CH_3_), 1.26 (t, *J* = 7.1 Hz, 6H, 2CH_3_). ^13^C-NMR (101 MHz, CDCl_3_) *δ* 167.5, 159.5, 132.9, 131.1, 125.9, 115.2, 113.9, 90.9, 87.8, 75.0, 61.3, 55.2, 40.5, 18.7, 14.2. HRMS (ESI): *m*/*z* calcd. for C_21_H_28_NO_5_ [M + H]^+^ 374.1962, found 374.1963.

*(E)-Diethyl 2-(dimethylamino)-2-(2-methyl-4-(4-nitrophenyl)but-1-en-3-ynyl)malonate* (**4e**). Yield 52% (53.1 mg) as a pale yellow thick oil. ^1^H-NMR (300 MHz, CDCl_3_) *δ* 8.20 (d, *J* = 8.9 Hz, 2H, Ar), 7.59 (d, *J* = 8.9 Hz, 2H, Ar), 6.43 (s, 1H, CH), 4.33–4.25 (m, 4H, 2OCH_2_), 2.42 (s, 6H, N(CH_3_)_2_), 2.09 (s, 3H, CH_3_), 1.32 (t, *J* = 7.1 Hz, 6H, 2CH_3_). ^13^C-NMR (101 MHz, CDCl_3_) *δ* 167.3, 146.9, 134.1, 132.2, 130.1, 125.0, 123.5, 97.2, 85.9, 75.1, 61.4, 40.4, 18.2, 14.2. HRMS (ESI): *m*/*z* calcd. for C_20_H_25_N_2_O_6_ [M + H]^+^ 389.1707, found 389.1709.

*(E)-Diethyl 2-(4-(4-(9H-carbazol-9-yl)phenyl)-2-methylbut-1-en-3-ynyl)-2-(dimethylamino)malonate* (**4f**). Yield 70% (75.1 mg) as a pale yellow thick oil. ^1^H-NMR (400 MHz, CDCl_3_) *δ* 8.13 (d, *J* = 7.7 Hz, 2H, Ar), 7.66 (d, *J* = 8.2 Hz, 2H, Ar), 7.52 (d, *J* = 8.1 Hz, 2H, Ar), 7.41–7.38 (m, 4H, Ar), 7.31–7.27 (m, 2H, Ar), 6.41 (s, 1H, CH), 4.29 (q, *J* = 7.0 Hz, 4H, 2OCH_2_), 2.44 (s, 6H, N(CH_3_)_2_), 2.11 (s, 3H, CH_3_), 1.31 (t, *J* = 7.1 Hz, 6H, 2CH_3_). ^13^C-NMR (101 MHz, CDCl_3_) *δ* 167.5, 140.5, 137.5, 133.1, 132.4, 126.7, 126.0, 125.6, 123.5, 122.1, 120.3, 120.2, 109.7, 93.0, 87.2, 75.2, 61.4, 40.5, 18.6, 14.3. HRMS (ESI): *m*/*z* calcd. for C_32_H_33_N_2_O_4_ [M + H]^+^ 509.2435, found 509.2430.

*(E)-Diethyl 2-(dimethylamino)-2-(4-(4-(diphenylamino)phenyl)-2-methylbut-1-en-3-ynyl) malonate* (**4g**). Yield 61% (63.6 mg) as a pale yellow thick oil. ^1^H-NMR (400 MHz, CDCl_3_) *δ* 7.27–7.23 (m, 6H, Ar), 7.09–7.07 (m, 4H, Ar), 7.05–7.01 (m, 2H, Ar), 6.95 (d, *J* = 8.7 Hz, 2H, Ar), 6.25 (s, 1H, CH), 4.24 (q, *J* = 7.1 Hz, 4H, 2OCH_2_), 2.38 (s, 6H, N(CH_3_)_2_), 2.02 (s, 3H, CH_3_), 1.28 (t, *J* = 7.1 Hz, 6H, 2CH_3_). ^13^C-NMR (101 MHz, CDCl_3_) *δ* 167.6, 147.8, 147.1, 132.5, 129.4, 124.9, 123.5, 122.2, 115.9, 91.4, 88.1, 75.1, 61.3, 40.6, 18.8, 14.2. HRMS (ESI): *m*/*z* calcd. for C_32_H_35_N_2_O_4_ [M + H]^+^ 511.2591, found 511.2581.

*(E)-Diethyl 2-(dimethylamino)-2-(2-methyl-4-o-tolylbut-1-en-3-ynyl)malonate* (**4h**). Yield 70% (76.4 mg) as a pale yellow thick oil. ^1^H-NMR (400 MHz, CDCl_3_) *δ* 7.37 (d, *J* = 7.6 Hz, 1H, Ar), 7.19–7.16 (m, 2H, Ar), 7.13–7.08 (m, 1H, Ar), 6.27 (s, 1H, CH), 4.24 (q, *J* = 7.1 Hz, 4H, 2OCH_2_), 2.41 (s, 3H, CH_3_), 2.38 (s, 6H, N(CH_3_)_2_), 2.04 (s, 3H, CH_3_), 1.27 (t, *J* = 7.1 Hz, 6H, 2CH_3_). ^13^C-NMR (101 MHz, CDCl_3_) *δ* 167.5, 140.1, 131.8, 131.4, 129.3, 128.2, 125.9, 125.5, 122.8, 96.1, 86.9, 75.1, 61.3, 40.4, 20.6, 18.7, 14.2. HRMS (ESI): *m*/*z* calcd. for C_21_H_28_NO_4_ [M + H]^+^ 358.2013, found 358.2015.

*(E)-Diethyl 2-(4-(2-(cyanomethyl)phenyl)-2-methylbut-1-en-3-ynyl)-2-(dimethylamino) malonate* (**4i**). Yield 69% (82.6 mg) as a pale yellow thick oil. ^1^H-NMR (400 MHz, CDCl_3_) *δ* 7.44–7.43 (m, 2H, Ar), 7.33–7.24 (m, 2H, Ar), 6.31 (s, 1H, CH), 4.23 (q, *J* = 6.8 Hz, 4H, 2OCH_2_), 3.85 (s, 2H, CH_2_), 2.36 (s, 6H, N(CH_3_)_2_), 2.04 (s, 3H, CH_3_), 1.26 (t, *J* = 6.9 Hz, 6H, 2CH_3_). ^13^C-NMR (101 MHz, CDCl_3_) *δ* 167.3, 132.9, 132.3, 131.6, 128.9, 128.1, 128.0, 125.2, 122.6, 117.3, 98.2, 84.5, 75.1, 61.4, 40.4, 22.6, 18.5, 14.2. HRMS (ESI): *m*/*z* calcd. for C_22_H_27_N_2_O_4_ [M + H]^+^ 383.1965, found 383.1966.

*(E)-Diethyl 2-(dimethylamino)-2-(2-methyloct-1-en-3-ynyl)malonate* (**4j**). Yield 58% (52.1 mg) as a pale yellow thick oil. ^1^H-NMR (300 MHz, CDCl_3_) *δ* 6.11 (s, 1H, CH), 4.25 (q, *J* = 7.1 Hz, 4H, 2OCH_2_), 2.38 (s, 6H, N(CH_3_)_2_), 2.31 (t, *J* = 6.9 Hz, 2H, CH_2_), 1.92 (s, 3H, CH_3_), 1.57–1.37 (m, 4H, 2CH_2_), 1.29 (t, *J* = 7.1 Hz, 6H, 2CH_3_), 0.93 (t, *J* = 7.1 Hz, 3H, CH_3_). ^13^C-NMR (101 MHz, CDCl_3_) *δ* 167.6, 130.1, 126.1, 88.8, 83.3, 74.9, 61.2, 40.4, 30.7, 21.9, 18.9, 18.9, 14.1, 13.5. HRMS (ESI): *m*/*z* calcd. for C_18_H_30_NO_4_ [M + H]^+^ 324.2169, found 324.2170.

*(E)-Diethyl 2-(dimethylamino)-2-(2-methyldec-1-en-3-ynyl)malonate* (**4k**). Yield 56% (61.6 mg) as a pale yellow thick oil. ^1^H-NMR (400 MHz, CDCl_3_) *δ* 6.05 (s, 1H, CH), 4.20 (q, *J* = 7.2 Hz, 4H, 2OCH_2_), 2.32 (s, 6H, N(CH_3_)_2_), 2.25 (t, *J* = 7.1 Hz, 2H, CH_2_), 1.87 (s, 3H, CH_3_), 1.48 (p, *J* = 7.1 Hz, 2H, CH_2_), 1.38–1.31 (m, 2H, CH_2_), 1.28–1.22 (m, 10H, 2CH_2_, 2CH_3_), 0.85 (t, *J* = 6.7 Hz, 3H, CH_3_). ^13^C-NMR (101 MHz, CDCl_3_) *δ* 167.6, 130.0, 126.2, 88.9, 83.3, 74.9, 61.2, 40.4, 31.3, 28.6, 28.5, 22.5, 19.2, 18.9, 14.2, 13.9. HRMS (ESI): *m*/*z* calcd. for C_20_H_34_NO_4_ [M + H]^+^ 352.2482, found 352.2477.

*(E)-Diethyl 2-(dimethylamino)-2-(5-hydroxy-2,5-dimethylhex-1-en-3-ynyl)malonate* (**4l**). Yield 65% (63.5 mg) as a pale yellow thick oil. ^1^H-NMR (300 MHz, CDCl_3_) *δ* 6.17 (s, 1H, CH), 4.29–4.21 (m, 4H, 2OCH_2_), 2.36 (s, 6H, N(CH_3_)_2_), 2.04 (br s, 1H, OH), 1.93 (s, 3H, CH_3_), 1.54 (s, 6H, 2CH_3_), 1.28 (t, *J* = 7.1 Hz, 6H, 2CH_3_). ^13^C-NMR (101 MHz, CDCl_3_) *δ* 167.4, 131.6, 125.2, 92.1, 84.6, 74.9, 65.3, 61.3, 40.4, 31.4, 18.5, 14.1. HRMS (ESI): *m*/*z* calcd. for C_17_H_28_NO_5_ [M + H]^+^ 326.1962, found 326.1968.

*(E)-Diethyl 2-(dimethylamino)-2-(2-methyl-4-(trimethylsilyl)but-1-en-3-ynyl)malonate* (**4m**). Yield 67% (68.8 mg) as a pale yellow thick oil. ^1^H-NMR (300 MHz, CDCl_3_) *δ* 6.27 (s, 1H, CH), 4.30–4.22 (m, 4H, 2OCH_2_), 2.38 (s, 6H, N(CH_3_)_2_), 1.96 (s, 3H, CH_3_), 1.30 (t, *J* = 7.1 Hz, 6H, 2CH_3_), 0.20 (s, 9H, 3CH_3_). ^13^C-NMR (101 MHz, CDCl_3_) *δ* 167.4, 132.6, 125.7, 107.7, 92.1, 74.9, 61.3, 40.4, 18.4, 14.1, -0.1. HRMS (ESI): *m*/*z* calcd. for C_17_H_30_NO_4_Si [M + H]^+^ 340.1939, found 340.1941.

*(E)-Diethyl 2-(dimethylamino)-2-(2,5,5-trimethylhex-1-en-3-ynyl)malonate* (**4n**). Yield 68% (85.6 mg) as a pale yellow thick oil. ^1^H-NMR (300 MHz, CDCl_3_) *δ* 6.08 (s, 1H, CH), 4.30–4.22 (m, 4H, 2OCH_2_), 2.38 (s, 6H, N(CH_3_)_2_), 1.91 (s, 3H, CH_3_), 1.29 (t, *J* = 7.1 Hz, 6H, 2CH_3_), 1.24 (s, 9H, 3CH_3_). ^13^C-NMR (101 MHz, CDCl_3_) *δ* 167.7, 129.7, 126.2, 96.8, 81.7, 74.9, 61.2, 40.4, 30.9, 27.7, 19.0, 14.2. HRMS (ESI): *m*/*z* calcd. for C_18_H_30_NO_4_ [M + H]^+^ 324.2169, found 324.2173.

*(E)-Diethyl 2-(dimethylamino)-2-(6,6-dimethylhept-1-en-4-ynyl)malonate* (**4n’**). Yield 5% (6.2 mg) as a pale yellow thick oil. ^1^H-NMR (300 MHz, CDCl_3_) *δ* 6.17 (dt, *J* = 15.7, 1.8 Hz, 1H, CH), 5.92 (dt, *J* = 15.8, 5.2 Hz, 1H, CH), 4.27 (q, *J* = 7.1 Hz, 4H, 2OCH_2_), 2.99 (dd, *J* = 5.2, 1.8 Hz, 2H, CH_2_), 2.42 (s, 6H, N(CH_3_)_2_), 1.30 (t, *J* = 7.1 Hz, 6H, 2CH_3_), 1.23 (s, 9H, 3CH_3_). ^13^C-NMR (101 MHz, CDCl_3_) *δ* 168.3, 130.8, 126.7, 74.4, 62.0, 61.3, 51.4, 40.5, 31.2, 27.3, 22.0, 14.1. HRMS (ESI): *m*/*z* calcd. for C_18_H_30_NO_4_ [M + H]^+^ 324.2169, found: 324.2173.

(E)-methyl 6-(2-(5-amino-1-benzyl-1H-1,2,3-triazol-4-yl)phenyl)-2-(dimethylamino)-4- methyl-2-(trifluoromethyl)hex-3-en-5-ynoate (**5a**). Yield 63% (49.0 mg) as a pale yellow oil. ^1^H-NMR (400 MHz, Chloroform-d) δ 7.70 (d, J = 7.7 Hz, 1H), 7.50 (d, J = 7.5 Hz, 1H), 7.40 (t, J = 8.6 Hz, 1H), 7.37–7.32 (m, 3H), 7.31–7.27 (m, 3H), 5.96 (s, 1H), 5.44 (s, 2H), 3.80 (s, 3H), 3.72 (s, 2H), 2.48 (s, 6H), 1.88 (s, 3H). ^19^F-NMR (376 MHz, Chloroform-d) δ -66.66 (s, 3F, CF_3_). ^13^C-NMR (101 MHz, Chloroform-d) δ 167.3, 138.1, 134.5, 133.9, 132.9, 132.1, 130.3, 129.4, 129.3, 128.7, 128.3, 128.2, 127.7, 127.5, 127.3, 125.9 (q, J = 295.8 Hz, CF_3_), 120.1, 95.4, 87.9, 73.9 (q, J = 24.0 Hz, >C<), 52.9, 50.9, 40.2, 18.3. HRMS (ESI): m/z calcd. for C_26_H_27_F_3_N_5_O_2_ [M + H]^+^ 498.2111, found 498.2114.

*(E)-diethyl 2-(4-(4-(5-amino-1-benzyl-1H-1,2,3-triazol-4-yl)phenyl)-2-methylbut-1-en- 3-yn-1-yl)-2-(dimethylamino)malonate* (**5b**). Yield 69% (54.0 mg) as a pale yellow oil. ^1^H-NMR (400 MHz, Chloroform-*d*) δ 7.59 (d, *J* = 8.0 Hz, 2H), 7.46 (d, *J* = 8.2 Hz, 2H), 7.36–7.31 (m, 3H), 7.22 (d, *J* = 6.6 Hz, 2H), 6.30 (s, 1H), 5.41 (s, 2H), 4.24 (dd, *J* = 7.0, 2.1 Hz, 4H), 3.81 (s, 2H), 2.38 (s, 6H), 2.03 (s, 3H), 1.28 (t, *J* = 7.1 Hz, 6H). ^13^C-NMR (101 MHz, Chloroform-*d*) δ 167.6, 137.7, 134.1, 132.3, 132.1, 131.6, 130.7, 129.4, 128.7, 127.4, 125.8, 125.3, 121.6, 92.8, 88.0, 75.2, 61.5, 50.8, 40.6, 29.8, 18.7, 14.3. HRMS (ESI+) of C_29_H_33_N_5_O_4_, *m*/*z*: calcd for [M + H]^+^ 516.2606, found 516.2601; for [M-NMe_2_]^+^ 471.2032, found 471.2024.

## 4. Conclusions

In summary, we have elaborated an efficient synthetic approach to the novel representative of α,α-disubstituted α-amino acid derivatives comprising 1,3-enyne moiety at their side chain. The method is based on Pd-catalyzed hydroalkynylation of functional allenes with various terminal acetylenes. The maximum conversion of starting compounds and the best yields of the target products have been achieved under moderate heating of reaction mixture in 1,4-dioxane in the presence of Pd(OAc)_2_/PPh_3_ (2/4 mol%) catalytic system for 4–8 h. The developed strategy is the first example of the metal-catalyzed allene–alkyne coupling to provide a convenient access to new unsaturated derivatives of α-amino acids in good yields and high regio- and stereoselectivity.

## Figures and Tables

**Table 1:** Optimization of the reaction conditions.

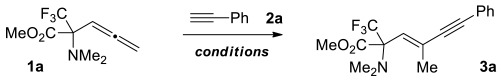				
Entry	Catalyst (mol%)	Ligand (mol%)	Solvent	Yield (%) ^a^
1	Pd(OAc)_2_ (5)	PPh_3_ (10)	toluene	80(71 ^b^)
2	Pd(OAc)_2_ (5)	PPh_3_ (10)	1,4-dioxane	87
3	Pd(OAc)_2_ (5)	dppf (5)	1,4-dioxane	95
4	Pd(OAc)_2_ (5)	(4-CF_3_Ph)_3_P (5)	1,4-dioxane	82
5	Pd(OAc)_2_ (5)	Xantphos (5)	1,4-dioxane	82
6	Pd(OAc)_2_ (5)	BrettPhos (5)	1,4-dioxane	NR
7	PdCl_2_(PPh_3_)_2_ (5)	-	toluene	NR
8	Pd(OAc)_2_ (2)	dppf (2)	1,4-dioxane	93
9	Pd(OAc)_2_ (1)	dppf (1)	1,4-dioxane	95 ^c^
**10**	**Pd(OAc)_2_ (2)**	**PPh_3_ (4)**	**1,4-dioxane**	**89(82 ^b^)**
11	Pd(OAc)_2_ (1)	PPh_3_ (2)	1,4-dioxane	94 ^c^
12	Pd(OAc)_2_ (2)	PPh_3_ (4)	toluene	84
13	Pd(OAc)_2_ (2)	PPh_3_ (4)	2-MeTHF	88 ^d^

Reagents and conditions: allene **1a** (0.1 mmol), acetylene **2a** (0.12 mmol), solvent (2 mL), 55 °C, 1 h; ^a^ determined by ^19^F-NMR spectroscopy; ^b^ isolated yield; ^c^ for 24 h; ^d^ for 4 h. The bold line indicates the best reaction conditions.

## Data Availability

Data are contained within the article and Supplementary Materials.

## Supplementary Materials

The following supporting information can be downloaded at: https://www.mdpi.com/article/10.3390/molecules30173623/s1. The following are available online: copies of ^1^H, ^19^F and ^13^C-NMR spectra for all novel compounds as well as copies of 2D ^1^H^19^F-HOESY NMR Spectrum for **3a**.
