# Supplementary material for: Regioselective Pd-Catalyzed Hydroalkynylation of Allenyl-Containing α-Amino Acid Derivatives with Terminal Alkynes
Source: Molecules. 2025 Sep 4;30(17):3623. doi: 10.3390/molecules30173623 (PMC12430138; doi:10.3390/molecules30173623)

Supporting information *for*

Regioselective Pd-catalyzed hydroalkynylation of allenyl-containing α-amino acid derivatives with terminal alkynes

Alexandra S. Bubnova, Daria V. Vorobyeva, Ivan A. Godovikov, Anna N. Philippova, Pavel S. Gribanov, Evgenia P. Antoshkina, Sergey N. Osipov*

[a]A.N. Nesmeyanov Institute of Organoelement compounds, Russian Academy of Sciences, Vavilov str. 28/1, 119334 Moscow, Russia

*Corresponding author: osipov@ineos.ac.ru

Table of contents

General information S2

General procedure for hydroalkynylation of allenes with terminal alkynes. S2

General Procedure for preparation of 5-Amino-1,2,3-triazole derivatives 5a and 5b via Dipolar Azide–Nitrile Cycloaddition (DCR) S2

Characterization Data of new compounds S3

^{1}H NMR, ^{19}F NMR and ^{13}C NMR Spectra. S14

2D ^{1}H^{19}F-HOESY NMR Spectrum for 3a S99

General information

All the reactions were carried out under argon atmosphere, and the solvents were distilled from appropriate drying agents prior to use. All reagents were used as purchased from Sigma-Aldrich (Munich, Germany). Analytical TLC was performed with Merck silica gel 60 F 254 plates (Darmstadt, Germany); visualization was accomplished with UV light, iodine vapors or $Ce(SO_4)_2$ solution in 5% H_2SO_4. Chromatography was carried out using Merck silica gel (Kieselgel 60, 0.063–0.200 mm, Darmstadt, Germany) and petroleum ether/ethyl acetate as an eluent. NMR spectra were obtained with Bruker AV-300 (Karlsruhe, Germany) and Inova-400 (Varian, Palo Alto, CA, USA) spectrometers operating at 300 and 400 MHz, respectively, for ^{1}H (TMS reference), at 101 MHz for ^{13}C, at 282 and 376 MHz for ^{19}F (CCl_3F reference). High-resolution mass spectra were recorded on a LCMS-9030 device (Shimadzu, Japan) by electrospray ionization mass spectrometry (ESI-MS). Measurements were carried out in positive ion mode; samples were dissolved in acetonitrile and injected into the mass-spectrometer chamber from an HPLC system LC-40 Nexera (Shimadzu, Japan).

General procedure for hydroalkynylation of allenes with terminal alkynes.

Under argon in a schlenk tube with a magnetic stirring bar, corresponding allene (100 mg, 0.44 mmol, 1.0 equiv.) and corresponding alkyne (0.54 mmol, 1.2 equiv.) were dissolved in dry 1,4-dioxane (2 mL). Then $Pd(OAc)_2$ (2 mg, 9 μmol, 2 mol%) and PPh_3 (4.7 mg, 18 μmol, 4 mol%) were added, and the reaction mixture was stirred at 55°C for 4-8 h until the completion of the reaction monitored by TLC and ^{19}F NMR. The reaction mixture was cooled to room temperature and concentrated under reduced pressure. Purification by chromatography (gradient elution: petroleum ether/dichloromethane = 1:1, petroleum ether/ethyl acetate = 15:1) gave analytically pure desired product.

General Procedure for preparation of 5-Amino-1,2,3-triazole derivatives 5a and 5b via Dipolar Azide–Nitrile Cycloaddition (DCR)

5-amino-1,2,3-triazole derivatives **5a** and **5b** were synthesized according to literature procedure (*Molecules* **2024**, *29*, 215) with minor modifications. A screw-cap vial equipped with a magnetic stir bar was charged with 0.2 mmol of **3k** or **4c**, 3.0 equiv. of benzyl azide, DMSO (4 mL), and 0.5 equiv. of powdered potassium tert-butoxide. The reaction mixture was allowed to stir for 24 h at room temperature. Upon completion, the mixture was poured into water and extracted with dichloromethane. The combined organic phases were washed with brine, dried over $MgSO_4$, filtered, and concentrated under reduced pressure. Purification by chromatography (eluent–hexane: ethyl acetate 1:1) gave analytically pure desired product.

Characterization Data of new compounds

(E)-Methyl 2-(dimethylamino)-4-methyl-6-phenyl-2-(trifluoromethyl)hex-3-en-5-ynoate (**3a**)

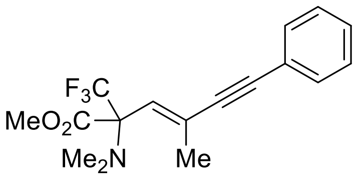

Yield 82% (110.7 mg) as a pale yellow thick oil. ^{1}H NMR (400 MHz, $CDCl_3$) δ 7.45 – 7.42 (m, 2H, Ar), 7.31 – 7.30 (m, 3H, Ar), 5.97 (s, 1H, CH), 3.80 (s, 3H, OCH_3), 2.52 (s, 6H, $N(CH_3)_2$), 2.11 (s, 3H, CH_3). ^{13}C NMR (101 MHz, $CDCl_3$) δ 167.3, 131.6, 128.5, 128.4, 128.3, 127.5, 125.8 (q, J = 297.0 Hz, CF_3), 122.8, 91.5, 88.7, 73.8 (q, J = 24.0 Hz, >*C*<), 52.7, 39.9, 18.6. ^{19}F NMR (376 MHz, $CDCl_3$) δ -66.70 (s, 3F, CF_3). HRMS (ESI): *m/z* calcd. for $C_{17}H_{19}F_3NO_2$ $[M+H]^+$ 326.1362, found 326.1362.

(E)-Methyl 2-(dimethylamino)-4-methyl-6-p-tolyl-2-(trifluoromethyl)hex-3-en-5-ynoate (**3b**)

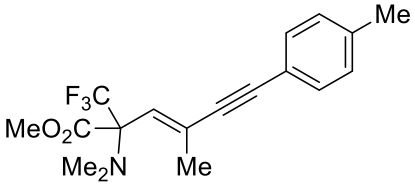

Yield 84% (83.7 mg) as a pale yellow thick oil. ^{1}H NMR (400 MHz, $CDCl_3$) δ 7.32 (d, J = 8.0 Hz, 2H, Ar), 7.11 (d, J = 7.8 Hz, 2H, Ar), 5.93 (s, 1H, CH), 3.79 (s, 3H, OCH_3), 2.51 (s, 6H, $N(CH_3)_2$), 2.33 (s, 3H, CH_3), 2.08 (s, 3H, CH_3). ^{13}C NMR (101 MHz, $CDCl_3$) δ 167.3, 138.6, 131.5, 129.1, 128.6, 127.1, 125.8 (q, J = 297.1 Hz, CF_3), 119.7, 90.9, 88.9, 73.8 (q, J = 24.3 Hz, >*C*<), 52.7, 40.0, 21.4, 18.7. ^{19}F NMR (376 MHz, $CDCl_3$) δ -66.72 (s, 3F, CF_3). HRMS (ESI): *m/z* calcd. for $C_{18}H_{21}F_3NO_2$ $[M+H]^+$ 340.1519, found 340.1522.

(E)-Methyl 6-(4-(cyanomethyl)phenyl)-2-(dimethylamino)-4-methyl-2-(trifluoromethyl)hex-3-en-5-ynoate (**3c**)

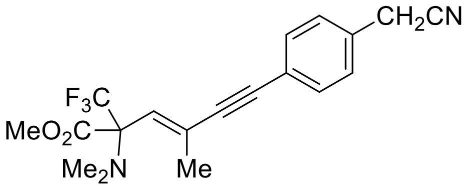

Yield 58% (57.5 mg) as a pale yellow thick oil. ^{1}H NMR (400 MHz, $CDCl_3$) δ 7.43 (d, J = 8.1 Hz, 2H, Ar), 7.27 (d, J = 8.1 Hz, 2H, Ar), 5.96 (s, 1H, CH), 3.79 (s, 3H, OCH_3), 3.73 (s, 2H, CH_2), 2.50 (s, 6H, $N(CH_3)_2$), 2.08 (s, 3H, CH_3). ^{13}C NMR (101 MHz, $CDCl_3$) δ 167.2, 132.2, 130.1, 128.3, 128.0, 127.9, 125.7 (q, J = 296.9 Hz, CF_3), 122.8, 117.3, 92.3, 87.7, 73.8 (q, J = 24.0 Hz, >*C*<), 52.7, 40.0, 23.5, 18.6. ^{19}F NMR (376 MHz, $CDCl_3$) δ -66.72 (s, 3F, CF_3). HRMS (ESI): *m/z* calcd. for $C_{19}H_{20}F_3N_2O_2$ $[M+H]^+$ 365.1471, found 365.1477.

(E)-Methyl 2-(dimethylamino)-6-(4-methoxyphenyl)-4-methyl-2-(trifluoromethyl)hex-3-en-5-ynoate (**3d**)

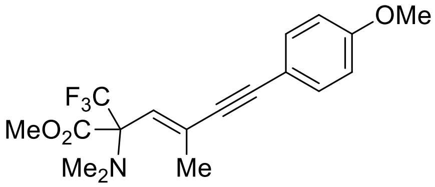

Yield 74% (91.2 mg) as a pale yellow thick oil. ^{1}H NMR (400 MHz, $CDCl_3$) δ 7.36 (d, J = 8.8 Hz, 2H, Ar), 6.82 (d, J = 8.8 Hz, 2H, Ar), 5.91 (s, 1H, CH), 3.79 (s, 6H, $2OCH_3$), 2.50 (s, 6H, $N(CH_3)_2$), 2.08 (s, 3H, CH_3). ^{13}C NMR (101 MHz, $CDCl_3$) δ 167.3, 159.7, 133.0, 128.7, 126.7, 125.8 (q, J = 297.0 Hz, CF_3), 114.8, 113.9, 90.3, 88.7, 73.8 (q, J = 24.1 Hz, >*C*<), 55.2, 52.6, 39.9, 18.7. ^{19}F NMR (376 MHz, $CDCl_3$) δ -66.73 (s, 3F, CF_3). HRMS (ESI): *m/z* calcd. for $C_{18}H_{21}F_3NO_3$ $[M+H]^+$ 356.1468, found 356.1471.

(E)-Methyl 2-(dimethylamino)-4-methyl-6-(4-nitrophenyl)-2-(trifluoromethyl)hex-3-en-5-ynoate (**3e**)

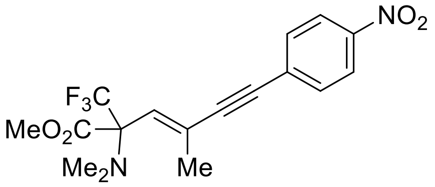

Yield 58% (67.3 mg) as a pale yellow thick oil. ^{1}H NMR (400 MHz, $CDCl_3$) δ 8.17 (d, J = 7.2 Hz, 2H, Ar), 7.56 (d, J = 8.0 Hz, 2H, Ar), 6.03 (s, 1H, CH), 3.81 (s, 3H, OCH_3), 2.50 (s, 6H, $N(CH_3)_2$), 2.11 (s, 3H, CH_3). ^{13}C NMR (101 MHz, $CDCl_3$) δ 167.0, 147.1, 132.3, 129.7, 129.6, 127.8, 125.6 (q, J = 296.8 Hz, CF_3), 123.6, 96.4, 86.6, 73.8 (q, J = 23.9 Hz, >*C*<), 52.8, 40.0, 18.4. ^{19}F NMR (376 MHz, $CDCl_3$) δ -66.68 (s, 3F, CF_3). HRMS (ESI): *m/z* calcd. for $C_{17}H_{18}F_3N_2O_4$ $[M+H]^+$ 371.1213, found 371.1224.

(E)-Methyl 6-(4-chlorophenyl)-2-(dimethylamino)-4-methyl-2-(trifluoromethyl)hex-3-en-5-ynoate (**3f**)

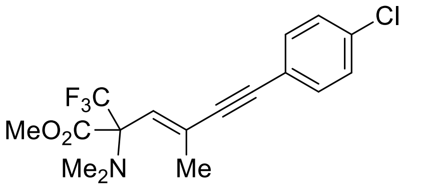

Yield 51% (52.6 mg) as a pale yellow thick oil. ^{1}H NMR (400 MHz, $CDCl_3$) δ 7.35 (d, J = 8.5 Hz, 2H, Ar), 7.28 (d, J = 8.4 Hz, 2H, Ar), 5.95 (s, 1H, CH), 3.80 (s, 3H, OCH_3), 2.50 (s, 6H, $N(CH_3)_2$), 2.08 (s, 3H, CH_3). ^{13}C NMR (101 MHz, $CDCl_3$) δ 167.2, 134.5, 132.8, 128.6, 128.3, 127.9, 125.7 (q, J = 297.0 Hz, CF_3), 121.3, 92.4, 87.5, 73.8 (q, J = 24.2 Hz, >*C*<), 52.7, 40.0, 18.5. ^{19}F NMR (376 MHz, $CDCl_3$) δ -66.71 (s, 3F, CF_3). HRMS (ESI): *m/z* calcd. for $C_{17}H_{18}ClF_3NO_2$ $[M+H]^+$ 360.0973, found 360.0971.

(E)-Methyl 6-(4-bromophenyl)-2-(dimethylamino)-4-methyl-2-(trifluoromethyl)hex-3-en-5-ynoate (**3g**)

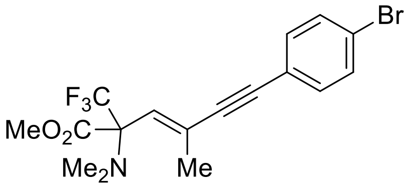

Yield 54% (99.2 mg) as a pale yellow thick oil. ^{1}H NMR (400 MHz, $CDCl_3$) δ 7.43 (d, J = 8.5 Hz, 2H, Ar), 7.28 (d, J = 8.5 Hz, 2H, Ar), 5.96 (s, 1H, CH), 3.80 (s, 3H, OCH_3), 2.50 (s, 6H, $N(CH_3)_2$), 2.08 (s, 3H, CH_3). ^{13}C NMR (101 MHz, $CDCl_3$) δ 167.2, 132.9, 131.6, 128.3, 127.9, 125.7 (q, J = 296.9 Hz, CF_3), 122.7, 121.7, 92.5, 87.5, 73.8 (q, J = 24.1 Hz, >*C*<), 52.7, 40.0, 18.5. ^{19}F NMR (376 MHz, $CDCl_3$) δ -66.70 (s, 3F, CF_3). HRMS (ESI): *m/z* calcd. for $C_{17}H_{17}BrF_3NO_2$ $[M]^+$ 404.0468, found 404.0473.

(E)-Methyl 6-(4-(9H-carbazol-9-yl)phenyl)-2-(dimethylamino)-4-methyl-2-(trifluoromethyl)hex-3-en-5-ynoate (**3h**)

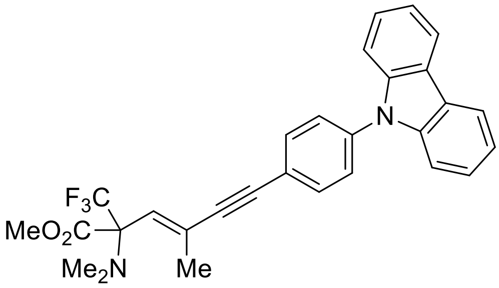

Yield 83% (118.7 mg) as a pale yellow thick oil. ^{1}H NMR (400 MHz, $CDCl_3$) δ 8.13 (d, J = 7.7 Hz, 2H, Ar), 7.66 (d, J = 8.3 Hz, 2H, Ar), 7.54 (d, J = 8.3 Hz, 2H, Ar), 7.44 – 7.39 (m, 4H, Ar), 7.31 – 7.27 (m, 2H, Ar), 6.03 (s, 1H, CH), 3.84 (s, 3H, OCH_3), 2.55 (s, 6H, $N(CH_3)_2$), 2.15 (s, 3H, CH_3). ^{13}C NMR (101 MHz, $CDCl_3$) δ 167.3, 140.5, 137.7, 133.1, 128.4, 127.9, 126.8, 126.0, 125.8 (q, J = 297.1 Hz, CF_3), 123.5, 121.7, 120.3, 120.2, 109.7, 92.4, 87.9, 73.7, 52.8, 40.1, 18.7. ^{19}F NMR (376 MHz, $CDCl_3$) δ -66.66 (s, 3F, CF_3). HRMS (ESI): *m/z* calcd. for $C_{29}H_{26}F_3N_2O_2$ $[M+H]^+$ 491.1936, found 491.1941.

(E)-Methyl 2-(dimethylamino)-6-(4-(diphenylamino)phenyl)-4-methyl-2-(trifluoromethyl)hex-3-en-5-ynoate (**3i**)

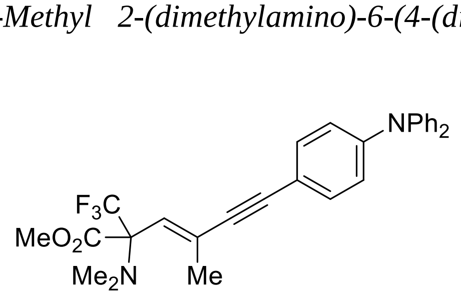

Yield 61% (66.5 mg) as a pale yellow thick oil. ^{1}H NMR (400 MHz, $CDCl_3$) δ 7.28 – 7.24 (m, 6H, Ar), 7.10 – 7.03 (m, 6H, Ar), 6.96 (d, J = 8.5 Hz, 2H, Ar), 5.91 (s, 1H, CH), 3.80 (s, 3H, OCH_3), 2.52 (s, 6H, $N(CH_3)_2$), 2.08 (s, 3H, CH_3). ^{13}C NMR (101 MHz, $CDCl_3$) δ 167.3, 148.1, 147.1, 132.5, 129.4, 128.7, 126.7, 125.8 (q, J = 297.1 Hz, CF_3), 125.0, 123.6, 122.0, 115.4, 90.9, 89.0, 73.8 (q, J = 24.7 Hz, >*C*<), 52.7, 40.0,

18.7. ^{19}F NMR (376 MHz, $CDCl_3$) δ -66.69 (s, 3F, CF_3). HRMS (ESI): *m/z* calcd. for $C_{29}H_{27}F_3N_2O_2$ $[M]^+$ 492.2019, found 492.2021.

(E)-Methyl 2-(dimethylamino)-4-methyl-6-o-tolyl-2-(trifluoromethyl)hex-3-en-5-ynoate (**3j**)

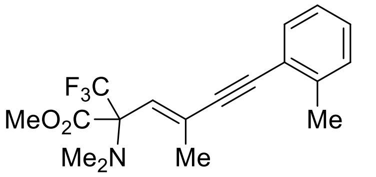

Yield 76% (86.1 mg) as a pale yellow thick oil. ^{1}H NMR (400 MHz, $CDCl_3$) δ 7.40 (d, J = 7.5 Hz, 1H, Ar), 7.23 – 7.18 (m, 2H, Ar), 7.15 – 7.11 (m, 1H, Ar), 5.95 (s, 1H, CH), 3.81 (s, 3H, OCH_3), 2.53 (s, 6H, $N(CH_3)_2$), 2.43 (s, 3H, CH_3), 2.12 (s, 3H, CH_3). ^{13}C NMR (101 MHz, $CDCl_3$) δ 167.3, 140.2, 131.9, 129.4, 128.7, 128.5, 126.9, 125.8 (q, J = 297.0 Hz, CF_3), 125.5, 122.5, 95.5, 87.7, 73.8 (q, J = 24.1 Hz, >*C*<), 52.7, 40.0, 20.6, 18.8. ^{19}F NMR (376 MHz, $CDCl_3$) δ -66.71 (s, 3F, CF_3). HRMS (ESI): *m/z* calcd. for $C_{18}H_{21}F_3NO_2$ $[M+H]^+$ 340.1519, found 340.1518.

(E)-Methyl 6-(2-(cyanomethyl)phenyl)-2-(dimethylamino)-4-methyl-2-(trifluoromethyl)hex-3-en-5-ynoate (**3k**)

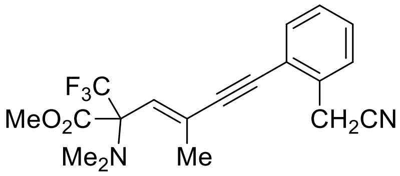

Yield 81% (78.7 mg) as a pale yellow thick oil. ^{1}H NMR (400 MHz, $CDCl_3$) δ 7.47 – 7.45 (m, 2H, Ar), 7.36 – 7.27 (m, 2H, Ar), 5.98 (s, 1H, CH), 3.86 (s, 2H, CH_2), 3.81 (s, 3H, OCH_3), 2.51 (s, 6H, $N(CH_3)_2$), 2.12 (s, 3H, CH_3). ^{13}C NMR (101 MHz, $CDCl_3$) δ 167.1, 132.4, 131.7, 129.2, 128.4, 128.1, 127.9, 125.6 (q, J = 296.7 Hz, CF_3), 122.3, 117.2, 97.6, 85.3, 73.8 (q, J = 24.3 Hz, >*C*<), 52.8, 40.0, 22.7, 18.6. ^{19}F NMR (376 MHz, $CDCl_3$) δ -66.70 (s, 3F, CF_3). HRMS (ESI): *m/z* calcd. for $C_{19}H_{20}F_3N_2O_2$ $[M+H]^+$ 365.1471, found 365.1472.

(E)-Methyl 2-(dimethylamino)-4-methyl-2-(trifluoromethyl)dec-3-en-5-ynoate (**3l**)

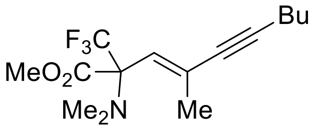

Yield 66% (72.2 mg) as a pale yellow thick oil. ^{1}H NMR (400 MHz, $CDCl_3$) δ 5.72 (s, 1H, CH), 3.75 (s, 3H, OCH_3), 2.45 (s, 6H, $N(CH_3)_2$), 2.26 (t, J = 7.0 Hz, 2H, CH_2), 1.93 (s, 3H, CH_3), 1.51 – 1.44 (m, 2H, CH_2), 1.42 – 1.33 (m, 2H, CH_2), 0.88 (t, J = 7.2 Hz, 3H, CH_3). ^{13}C NMR (101 MHz, $CDCl_3$) δ 167.4, 128.9, 125.9, 125.8 (q, J = 297.0 Hz, CF_3), 89.8, 82.9, 73.6 (q, J = 24.0 Hz, >*C*<), 52.5, 39.9, 30.6, 21.9, 18.9, 18.8,

13.5. ^{19}F NMR (376 MHz, $CDCl_3$) δ -66.91 (s, 3F, CF_3). HRMS (ESI): *m/z* calcd. for $C_{15}H_{23}F_3NO_2$ $[M+H]^+$ 306.1676, found: 306.1682.

(E)-Methyl 2-(dimethylamino)-4-methyl-2-(trifluoromethyl)dodec-3-en-5-ynoate (**3m**)

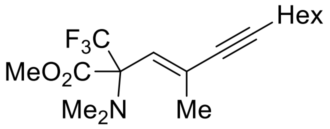

Yield 61% (63.8 mg) as a pale yellow thick oil. ^{1}H NMR (400 MHz, $CDCl_3$) δ 5.74 (s, 1H, CH), 3.77 (s, 3H, OCH_3), 2.46 (s, 6H, $N(CH_3)_2$), 2.26 (t, J = 7.1 Hz, 2H, CH_2), 1.95 (s, 3H, CH_3), 1.54 – 1.47 (m, 2H, CH_2), 1.40 – 1.33 (m, 2H, CH_2), 1.30 – 1.24 (m, 4H, CH_2), 0.87 (t, J = 6.8 Hz, 3H, CH_3). ^{13}C NMR (101 MHz, $CDCl_3$) δ 167.4, 128.9, 125.9, 125.8 (q, J = 297.1 Hz, CF_3), 89.9, 82.9, 73.6 (q, J = 23.9 Hz, >*C*<), 52.6, 39.9, 31.3, 28.5, 22.5, 19.2, 18.9, 13.9. ^{19}F NMR (376 MHz, $CDCl_3$) δ -66.87 (s, 3F, CF_3). HRMS (ESI): *m/z* calcd. for $C_{17}H_{27}F_3NO_2$ $[M+H]^+$ 334.1988, found 334.1988.

(E)-Methyl 2-(dimethylamino)-7-hydroxy-4,7-dimethyl-2-(trifluoromethyl)oct-3-en-5-ynoate (**3n**)

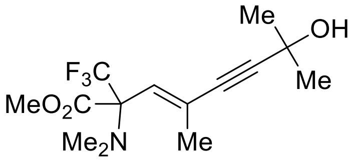

Yield 73% (81.4 mg) as a pale yellow thick oil. ^{1}H NMR (400 MHz, $CDCl_3$) δ 5.77 (s, 1H, CH), 3.75 (s, 3H, OCH_3), 2.44 (s, 6H, $N(CH_3)_2$), 2.29 (s, 1H, OH), 1.94 (s, 3H, CH_3), 1.49 (s, 6H, $2CH_3$). ^{13}C NMR (101 MHz, $CDCl_3$) δ 167.2, 128.0, 127.4, 125.7 (q, J = 296.9 Hz, CF_3), 93.0, 84.2, 73.6 (q, J = 24.0 Hz, >*C*<), 65.3, 52.6, 39.9, 31.3, 18.6. ^{19}F NMR (376 MHz, $CDCl_3$) δ -66.83 (s, 3F, CF_3). HRMS (ESI): *m/z* calcd. for $C_{14}H_{21}F_3NO_3$ $[M+H]^+$ 308.1468, found: 308.1468.

(E)-Methyl 2-(dimethylamino)-4-methyl-2-(trifluoromethyl)-6-(trimethylsilyl)hex-3-en-5-ynoate (**3o**)

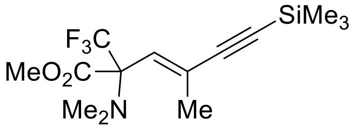

Yield 70% (58.6 mg) as a pale yellow thick oil. ^{1}H NMR (400 MHz, $CDCl_3$) δ 5.87 (s, 1H, CH), 3.76 (s, 3H, OCH_3), 2.46 (s, 6H, $N(CH_3)_2$), 1.97 (s, 3H, CH_3), 0.16 (s, 9H, $3CH_3$). ^{13}C NMR (101 MHz, $CDCl_3$) δ 167.1, 128.5, 128.2, 125.7 (q, J = 297.0 Hz, CF_3), 107.0, 93.3, 73.7 (q, J = 24.0 Hz, >*C*<), 52.6, 39.9, 18.5, -0.2. ^{19}F NMR (376 MHz, $CDCl_3$) δ -66.76 (s, 3F, CF_3). HRMS (ESI): *m/z* calcd. for $C_{14}H_{23}F_3NO_2Si$ $[M+H]^+$ 322.1445, found: 322.1443.

(E)-Methyl 2-(dimethylamino)-4,7,7-trimethyl-2-(trifluoromethyl)oct-3-en-5-ynoate (**3p**)

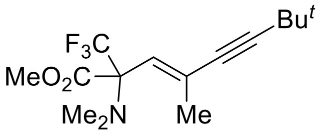

Yield 62% (52.4 mg) as a pale yellow thick oil. ^{1}H NMR (400 MHz, $CDCl_3$) δ 5.70 (s, 1H, CH), 3.77 (s, 3H, OCH_3), 2.46 (s, 6H, $N(CH_3)_2$), 1.94 (s, 3H, CH_3), 1.21 (s, 9H, CH_3). ^{13}C NMR (101 MHz, $CDCl_3$) δ 167.4, 129.0, 125.8 (q, J = 297.1 Hz, CF_3), 125.5, 97.8, 81.4, 73.6 (q, J = 24.2 Hz, >*C*<), 52.5, 39.9, 30.9, 27.7, 19.1. ^{19}F NMR (376 MHz, $CDCl_3$) δ -66.80 (s, 3F, CF_3). HRMS (ESI): *m/z* calcd. for $C_{15}H_{23}F_3NO_2$ $[M+H]^+$ 306.1675, found: 306.1673.

Methyl 2-(dimethylamino)-7,7-dimethyl-4-methylene-2-(trifluoromethyl)oct-5-ynoate (**3p'**)

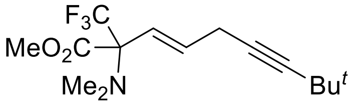

Yield 9% (7.5 mg) as a pale yellow thick oil. ^{1}H NMR (300 MHz, $CDCl_3$) δ 5.96 – 5.93 (m, 2H, 2CH), 3.85 (s, 3H, OCH_3), 3.01 (d, J = 3.1 Hz, 2H, CH_2), 2.52 (s, 6H, $N(CH_3)_2$), 1.23 (s, 9H, $3CH_3$). ^{19}F NMR (282 MHz, $CDCl_3$) δ -66.65 (s, 3F, CF_3). HRMS (ESI): *m/z* calcd. for $C_{15}H_{23}F_3NO_2$ $[M+H]^+$ 306.1675, found: 306.1674.

(E)-Diethyl 2-(dimethylamino)-2-(2-methyl-4-phenylbut-1-en-3-ynyl)malonate (**4a**)

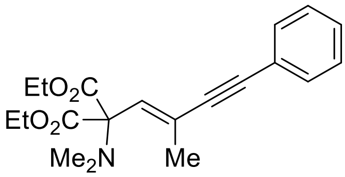

Yield 74% (76.5 mg) as a pale yellow thick oil. ^{1}H NMR (400 MHz, $CDCl_3$) δ 7.42 – 7.40 (m, 2H, Ar), 7.29 – 7.27 (m, 3H, Ar), 6.29 (s, 1H, CH), 4.26 – 4.20 (m, 4H, $2OCH_2$), 2.37 (s, 6H, $N(CH_3)_2$), 2.02 (s, 3H, CH_3), 1.26 (t, J = 7.1 Hz, 6H, $2CH_3$). ^{13}C NMR (101 MHz, $CDCl_3$) δ 167.5, 131.9, 131.5, 128.2, 128.1, 125.7, 123.1, 92.1, 87.8, 75.1, 61.3, 40.5, 18.6, 14.2. HRMS (ESI): *m/z* calcd. for $C_{20}H_{26}NO_4$ $[M+H]^+$ 344.1856, found 344.1854.

(E)-Diethyl 2-(dimethylamino)-2-(2-methyl-4-p-tolylbut-1-en-3-ynyl)malonate (**4b**)

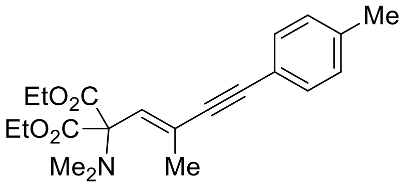

Yield 72% (72 mg) as a pale yellow thick oil. ^{1}H NMR (300 MHz, $CDCl_3$) δ 7.35 (d, J = 8.1 Hz, 2H, Ar), 7.13 (d, J = 7.9 Hz, 2H, Ar), 6.30 (s, 1H, CH), 4.31 – 4.24 (m, 4H, $2OCH_2$), 2.41 (s, 6H, $N(CH_3)_2$), 2.36

(s, 3H, CH_3), 2.05 (s, 3H, CH_3), 1.31 (t, J = 7.1 Hz, 6H, $2CH_3$). ^{13}C NMR (101 MHz, $CDCl_3$) δ 167.5, 138.2, 131.5, 131.4, 128.9, 125.8, 120.0, 91.5, 88.0, 75.1, 61.3, 40.4, 21.4, 18.6, 14.2. HRMS (ESI): *m/z* calcd. for $C_{21}H_{28}NO_4$ $[M+H]^+$ 358.2013, found 358.2007.

(E)-Diethyl 2-(4-(4-(cyanomethyl)phenyl)-2-methylbut-1-en-3-ynyl)-2-(dimethylamino)malonate (**4c**)

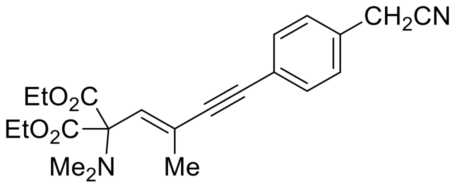

Yield 57% (72.3 mg) as a pale yellow thick oil. ^1H NMR (300 MHz, $CDCl_3$) δ 7.47 (d, J = 8.3 Hz, 2H, Ar), 7.31 – 7.28 (m, 2H, Ar), 6.35 (s, 1H, CH), 4.32 – 4.24 (m, 4H, $2OCH_2$), 3.77 (s, 2H, CH_2), 2.42 (s, 6H, $N(CH_3)_2$), 2.06 (s, 3H, CH_3), 1.31 (t, J = 7.1 Hz, 6H, $2CH_3$). ^{13}C NMR (101 MHz, $CDCl_3$) δ 167.4, 132.4, 132.1, 129.8, 127.9, 125.4, 123.1, 117.4, 92.9, 86.9, 75.1, 61.3, 40.4, 23.4, 18.5, 14.2. HRMS (ESI): *m/z* calcd. for $C_{22}H_{27}N_2O_4$ $[M+H]^+$ 383.1965, found 383.1966.

(E)-Diethyl 2-(dimethylamino)-2-(4-(4-methoxyphenyl)-2-methylbut-1-en-3-ynyl)malonate (**4d**)

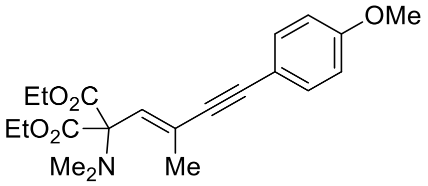

Yield 60% (65.7 mg) as a pale yellow thick oil. ^1H NMR (400 MHz, $CDCl_3$) δ 7.35 (d, J = 8.9 Hz, 2H, Ar), 6.81 (d, J = 8.9 Hz, 2H, Ar), 6.24 (s, 1H, CH), 4.26 – 4.20 (m, 4H, $2OCH_2$), 3.78 (s, 3H, OCH_3), 2.37 (s, 6H, $N(CH_3)_2$), 2.00 (s, 3H, CH_3), 1.26 (t, J = 7.1 Hz, 6H, $2CH_3$). ^{13}C NMR (101 MHz, $CDCl_3$) δ 167.5, 159.5, 132.9, 131.1, 125.9, 115.2, 113.9, 90.9, 87.8, 75.0, 61.3, 55.2, 40.5, 18.7, 14.2. HRMS (ESI): *m/z* calcd. for $C_{21}H_{28}NO_5$ $[M+H]^+$ 374.1962, found 374.1963.

(E)-Diethyl 2-(dimethylamino)-2-(2-methyl-4-(4-nitrophenyl)but-1-en-3-ynyl)malonate (**4e**)

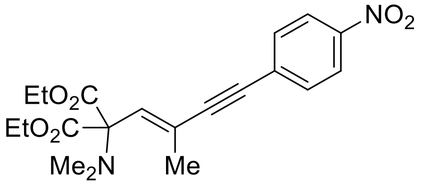

Yield 52% (53.1 mg) as a pale yellow thick oil. ^1H NMR (300 MHz, $CDCl_3$) δ 8.20 (d, J = 8.9 Hz, 2H, Ar), 7.59 (d, J = 8.9 Hz, 2H, Ar), 6.43 (s, 1H, CH), 4.33 – 4.25 (m, 4H, $2OCH_2$), 2.42 (s, 6H, $N(CH_3)_2$), 2.09 (s, 3H, CH_3), 1.32 (t, J = 7.1 Hz, 6H, $2CH_3$). ^{13}C NMR (101 MHz, $CDCl_3$) δ 167.3, 146.9, 134.1, 132.2, 130.1, 125.0, 123.5, 97.2, 85.9, 75.1, 61.4, 40.4, 18.2, 14.2. HRMS (ESI): *m/z* calcd. for $C_{20}H_{25}N_2O_6$ $[M+H]^+$ 389.1707, found 389.1709.

(E)-Diethyl 2-(4-(4-(9H-carbazol-9-yl)phenyl)-2-methylbut-1-en-3-ynyl)- z2-(dimethylamino)malonate (**4f**)

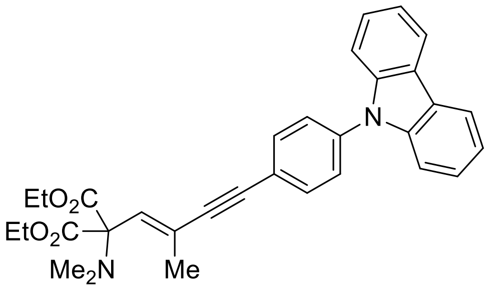

Yield 70% (75.1 mg) as a pale yellow thick oil. ^{1}H NMR (400 MHz, $CDCl_3$) δ 8.13 (d, J = 7.7 Hz, 2H, Ar), 7.66 (d, J = 8.2 Hz, 2H, Ar), 7.52 (d, J = 8.1 Hz, 2H, Ar), 7.41 – 7.38 (m, 4H, Ar), 7.31 – 7.27 (m, 2H, Ar), 6.41 (s, 1H, CH), 4.29 (q, J = 7.0 Hz, 4H, $2OCH_2$), 2.44 (s, 6H, $N(CH_3)_2$), 2.11 (s, 3H, CH_3), 1.31 (t, J = 7.1 Hz, 6H, $2CH_3$). ^{13}C NMR (101 MHz, $CDCl_3$) δ 167.5, 140.5, 137.5, 133.1, 132.4, 126.7, 126.0, 125.6, 123.5, 122.1, 120.3, 120.2, 109.7, 93.0, 87.2, 75.2, 61.4, 40.5, 18.6, 14.3. HRMS (ESI): *m/z* calcd. for $C_{32}H_{33}N_2O_4$ $[M+H]^+$ 509.2435, found 509.2430.

(E)-Diethyl 2-(dimethylamino)-2-(4-(4-(diphenylamino)phenyl)-2-methylbut-1-en-3-ynyl)malonate (**4g**)

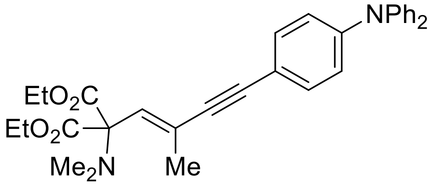

Yield 61% (63.6 mg) as a pale yellow thick oil. ^{1}H NMR (400 MHz, $CDCl_3$) δ 7.27 – 7.23 (m, 6H, Ar), 7.09 – 7.07 (m, 4H, Ar), 7.05 – 7.01 (m, 2H, Ar), 6.95 (d, J = 8.7 Hz, 2H, Ar), 6.25 (s, 1H, CH), 4.24 (q, J = 7.1 Hz, 4H, $2OCH_2$), 2.38 (s, 6H, $N(CH_3)_2$), 2.02 (s, 3H, CH_3), 1.28 (t, J = 7.1 Hz, 6H, $2CH_3$). ^{13}C NMR (101 MHz, $CDCl_3$) δ 167.6, 147.8, 147.1, 132.5, 129.4, 124.9, 123.5, 122.2, 115.9, 91.4, 88.1, 75.1, 61.3, 40.6, 18.8, 14.2. HRMS (ESI): *m/z* calcd. for $C_{32}H_{35}N_2O_4$ $[M+H]^+$ 511.2591, found 511.2581.

(E)-Diethyl 2-(dimethylamino)-2-(2-methyl-4-o-tolylbut-1-en-3-ynyl)malonate (**4h**)

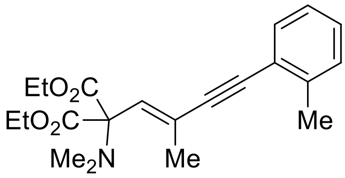

Yield 70% (76.4 mg) as a pale yellow thick oil. ^{1}H NMR (400 MHz, $CDCl_3$) δ 7.37 (d, J = 7.6 Hz, 1H, Ar), 7.19 – 7.16 (m, 2H, Ar), 7.13 – 7.08 (m, 1H, Ar), 6.27 (s, 1H, CH), 4.24 (q, J = 7.1 Hz, 4H, $2OCH_2$), 2.41 (s, 3H, CH_3), 2.38 (s, 6H, $N(CH_3)_2$), 2.04 (s, 3H, CH_3), 1.27 (t, J = 7.1 Hz, 6H, $2CH_3$). ^{13}C NMR (101 MHz, $CDCl_3$) δ 167.5, 140.1, 131.8, 131.4, 129.3, 128.2, 125.9, 125.5, 122.8, 96.1, 86.9, 75.1, 61.3, 40.4, 20.6, 18.7, 14.2. HRMS (ESI): *m/z* calcd. for $C_{21}H_{28}NO_4$ $[M+H]^+$ 358.2013, found 358.2015.

(E)-Diethyl 2-(4-(2-(cyanomethyl)phenyl)-2-methylbut-1-en-3-ynyl)-2-(dimethylamino)malonate (**4i**)

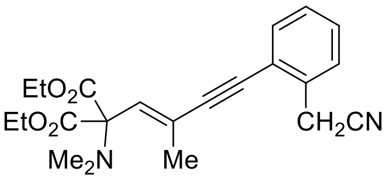

Yield 69% (82.6 mg) as a pale yellow thick oil. ^{1}H NMR (400 MHz, $CDCl_3$) δ 7.44 – 7.43 (m, 2H, Ar), 7.33 – 7.24 (m, 2H, Ar), 6.31 (s, 1H, CH), 4.23 (q, J = 6.8 Hz, 4H, $2OCH_2$), 3.85 (s, 2H, CH_2), 2.36 (s, 6H, $N(CH_3)_2$), 2.04 (s, 3H, CH_3), 1.26 (t, J = 6.9 Hz, 6H, $2CH_3$). ^{13}C NMR (101 MHz, $CDCl_3$) δ 167.3, 132.9, 132.3, 131.6, 128.9, 128.1, 128.0, 125.2, 122.6, 117.3, 98.2, 84.5, 75.1, 61.4, 40.4, 22.6, 18.5, 14.2. HRMS (ESI): *m/z* calcd. for $C_{22}H_{27}N_2O_4$ $[M+H]^+$ 383.1965, found 383.1966.

(E)-Diethyl 2-(dimethylamino)-2-(2-methyloct-1-en-3-ynyl)malonate (**4j**)

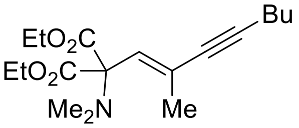

Yield 58% (52.1 mg) as a pale yellow thick oil. ^{1}H NMR (300 MHz, $CDCl_3$) δ 6.11 (s, 1H, CH), 4.25 (q, J = 7.1 Hz, 4H, $2OCH_2$), 2.38 (s, 6H, $N(CH_3)_2$), 2.31 (t, J = 6.9 Hz, 2H, CH_2), 1.92 (s, 3H, CH_3), 1.57 – 1.37 (m, 4H, $2CH_2$), 1.29 (t, J = 7.1 Hz, 6H, $2CH_3$), 0.93 (t, J = 7.1 Hz, 3H, CH_3). ^{13}C NMR (101 MHz, $CDCl_3$) δ 167.6, 130.1, 126.1, 88.8, 83.3, 74.9, 61.2, 40.4, 30.7, 21.9, 18.9, 18.9, 14.1, 13.5. HRMS (ESI): *m/z* calcd. for $C_{18}H_{30}NO_4$ $[M+H]^+$ 324.2169, found 324.2170.

(E)-Diethyl 2-(dimethylamino)-2-(2-methyldec-1-en-3-ynyl)malonate (**4k**)

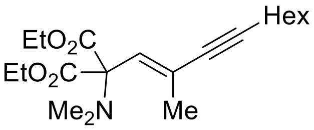

Yield 56% (61.6 mg) as a pale yellow thick oil. ^{1}H NMR (400 MHz, $CDCl_3$) δ 6.05 (s, 1H, CH), 4.20 (q, J = 7.2 Hz, 4H, $2OCH_2$), 2.32 (s, 6H, $N(CH_3)_2$), 2.25 (t, J = 7.1 Hz, 2H, CH_2), 1.87 (s, 3H, CH_3), 1.48 (p, J = 7.1 Hz, 2H, CH_2), 1.38 – 1.31 (m, 2H, CH_2), 1.28 – 1.22 (m, 10H, $2CH_2$, $2CH_3$), 0.85 (t, J = 6.7 Hz, 3H, CH_3). ^{13}C NMR (101 MHz, $CDCl_3$) δ 167.6, 130.0, 126.2, 88.9, 83.3, 74.9, 61.2, 40.4, 31.3, 28.6, 28.5, 22.5, 19.2, 18.9, 14.2, 13.9. HRMS (ESI): *m/z* calcd. for $C_{20}H_{34}NO_4$ $[M+H]^+$ 352.2482, found 352.2477.

(E)-Diethyl 2-(dimethylamino)-2-(5-hydroxy-2,5-dimethylhex-1-en-3-ynyl)malonate (**4l**)

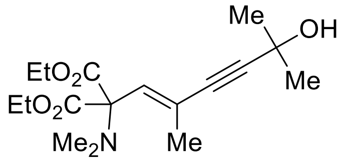

Yield 65% (63.5 mg) as a pale yellow thick oil. ^{1}H NMR (300 MHz, $CDCl_3$) δ 6.17 (s, 1H, CH), 4.29 – 4.21 (m, 4H, $2OCH_2$), 2.36 (s, 6H, $N(CH_3)_2$), 2.04 (br s, 1H, OH), 1.93 (s, 3H, CH_3), 1.54 (s, 6H, $2CH_3$), 1.28 (t, J = 7.1 Hz, 6H, $2CH_3$). ^{13}C NMR (101 MHz, $CDCl_3$) δ 167.4, 131.6, 125.2, 92.1, 84.6, 74.9, 65.3, 61.3, 40.4, 31.4, 18.5, 14.1. HRMS (ESI): *m/z* calcd. for $C_{17}H_{28}NO_5$ $[M+H]^+$ 326.1962, found 326.1968.

(E)-Diethyl 2-(dimethylamino)-2-(2-methyl-4-(trimethylsilyl)but-1-en-3-ynyl)malonate (**4m**)

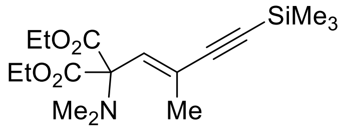

Yield 67% (68.8 mg) as a pale yellow thick oil. ^{1}H NMR (300 MHz, $CDCl_3$) δ 6.27 (s, 1H, CH), 4.30 – 4.22 (m, 4H, $2OCH_2$), 2.38 (s, 6H, $N(CH_3)_2$), 1.96 (s, 3H, CH_3), 1.30 (t, J = 7.1 Hz, 6H, $2CH_3$), 0.20 (s, 9H, $3CH_3$). ^{13}C NMR (101 MHz, $CDCl_3$) δ 167.4, 132.6, 125.7, 107.7, 92.1, 74.9, 61.3, 40.4, 18.4, 14.1, -0.1. HRMS (ESI): *m/z* calcd. for $C_{17}H_{30}NO_4Si$ $[M+H]^+$ 340.1939, found 340.1941.

(E)-Diethyl 2-(dimethylamino)-2-(2,5,5-trimethylhex-1-en-3-ynyl)malonate (**4n**)

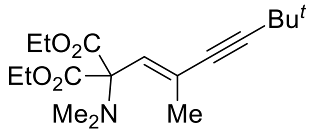

Yield 68% (85.6 mg) as a pale yellow thick oil. ^{1}H NMR (300 MHz, $CDCl_3$) δ 6.08 (s, 1H, CH), 4.30 – 4.22 (m, 4H, $2OCH_2$), 2.38 (s, 6H, $N(CH_3)_2$), 1.91 (s, 3H, CH_3), 1.29 (t, J = 7.1 Hz, 6H, $2CH_3$), 1.24 (s, 9H, $3CH_3$). ^{13}C NMR (101 MHz, $CDCl_3$) δ 167.7, 129.7, 126.2, 96.8, 81.7, 74.9, 61.2, 40.4, 30.9, 27.7, 19.0, 14.2. HRMS (ESI): *m/z* calcd. for $C_{18}H_{30}NO_4$ $[M+H]^+$ 324.2169, found 324.2173.

(E)-Diethyl 2-(dimethylamino)-2-(6,6-dimethylhept-1-en-4-ynyl)malonate (**4n'**)

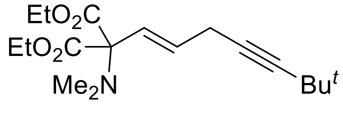

Yield 5% (6.2 mg) as a pale yellow thick oil. ^{1}H NMR (300 MHz, $CDCl_3$) δ 6.17 (dt, J = 15.7, 1.8 Hz, 1H, CH), 5.92 (dt, J = 15.8, 5.2 Hz, 1H, CH), 4.27 (q, J = 7.1 Hz, 4H, $2OCH_2$), 2.99 (dd, J = 5.2, 1.8 Hz, 2H, CH_2), 2.42 (s, 6H, $N(CH_3)_2$), 1.30 (t, J = 7.1 Hz, 6H, $2CH_3$), 1.23 (s, 9H, $3CH_3$). ^{13}C NMR (101 MHz, $CDCl_3$) δ 168.3, 130.8, 126.7, 74.4, 62.0, 61.3, 51.4, 40.5, 31.2, 27.3, 22.0, 14.1. HRMS (ESI): *m/z* calcd. for $C_{18}H_{30}NO_4$ $[M+H]^+$ 324.2169, found: 324.2173.

(E)-methyl 6-(2-(5-amino-1-benzyl-1H-1,2,3-triazol-4-yl)phenyl)-2-(dimethylamino)-4-methyl-2-(trifluoromethyl)hex-3-en-5-ynoate (**5a**)

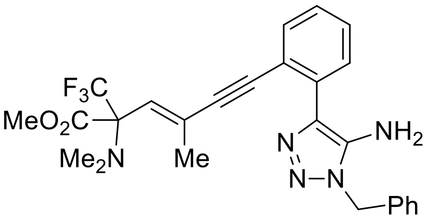

Yield 63% (49.0 mg) as a pale yellow oil. ^{1}H NMR (400 MHz, Chloroform-*d*) δ 7.70 (d, *J* = 7.7 Hz, 1H), 7.50 (d, *J* = 7.5 Hz, 1H), 7.40 (t, *J* = 8.6 Hz, 1H), 7.37 – 7.32 (m, 3H), 7.31 – 7.27 (m, 3H), 5.96 (s, 1H), 5.44 (s, 2H), 3.80 (s, 3H), 3.72 (s, 2H), 2.48 (s, 6H), 1.88 (s, 3H). ^{19}F NMR (376 MHz, Chloroform-*d*) δ -66.66 (s, 3F, CF_3). ^{13}C NMR (101 MHz, Chloroform-*d*) δ 167.3, 138.1, 134.5, 133.9, 132.9, 132.1, 130.3, 129.4, 129.3, 128.7, 128.3, 128.2, 127.7, 127.5, 127.3, 125.9 (q, *J* = 295.8 Hz, *C*F_3), 120.1, 95.4, 87.9, 73.9 (q, *J* = 24.0 Hz, >*C*<), 52.9, 50.9, 40.2, 18.3. HRMS (ESI): *m/z* calcd. for $C_{26}H_{27}F_3N_5O_2$ $[M+H]^+$ 498.2111, found 498.2114.

(E)-diethyl 2-(4-(4-(5-amino-1-benzyl-1H-1,2,3-triazol-4-yl)phenyl)-2-methylbut-1-en-3-yn-1-yl)-2-(dimethylamino)malonate (**5b**)

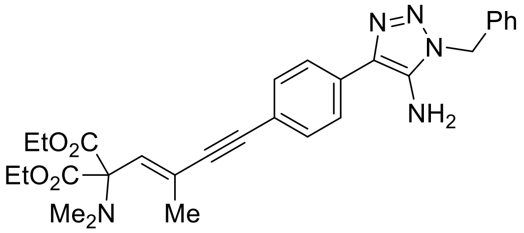

Yield 69% (54.0 mg) as a pale yellow oil. ^{1}H NMR (400 MHz, Chloroform-*d*) δ 7.59 (d, *J* = 8.0 Hz, 2H), 7.46 (d, *J* = 8.2 Hz, 2H), 7.36 – 7.31 (m, 3H), 7.22 (d, *J* = 6.6 Hz, 2H), 6.30 (s, 1H), 5.41 (s, 2H), 4.24 (dd, *J* = 7.0, 2.1 Hz, 4H), 3.81 (s, 2H), 2.38 (s, 6H), 2.03 (s, 3H), 1.28 (t, *J* = 7.1 Hz, 6H). ^{13}C NMR (101 MHz, Chloroform-*d*) δ 167.6, 137.7, 134.1, 132.3, 132.1, 131.6, 130.7, 129.4, 128.7, 127.4, 125.8, 125.3, 121.6, 92.8, 88.0, 75.2, 61.5, 50.8, 40.6, 29.8, 18.7, 14.3. HRMS (ESI+) of $C_{29}H_{33}N_5O_4$, *m/z*: calcd for $[M+H]^+$ 516.2606, found 516.2601; for $[M-NMe_2]^+$ 471.2032, found 471.2024.

^{1}H NMR, ^{19}F NMR and ^{13}C NMR Spectra.

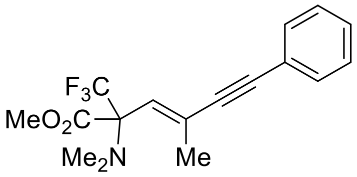

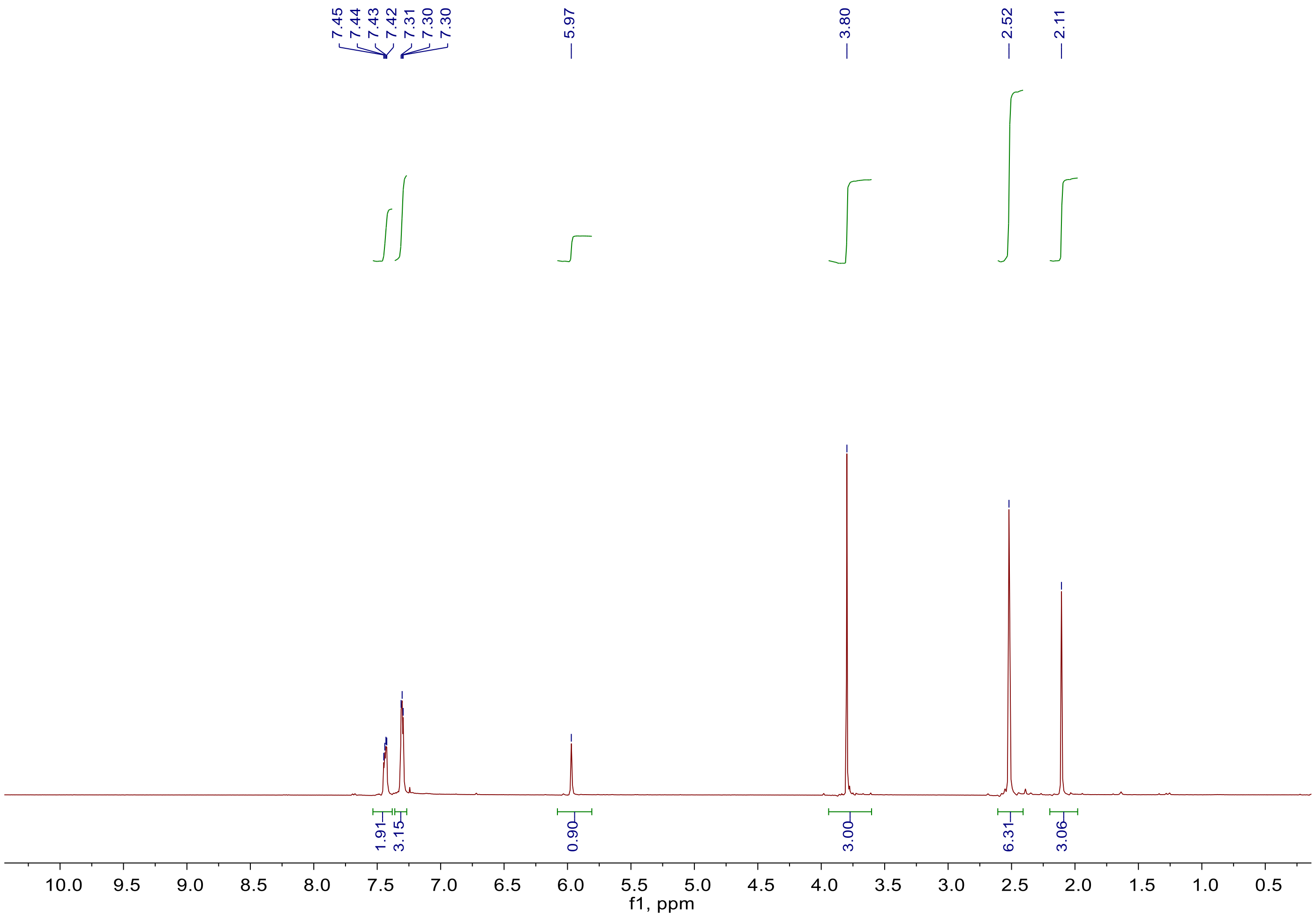

^{1}H spectrum of **3a** in $CDCl_3$

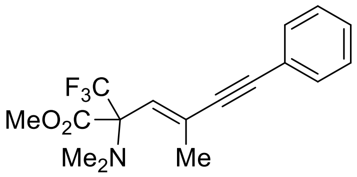

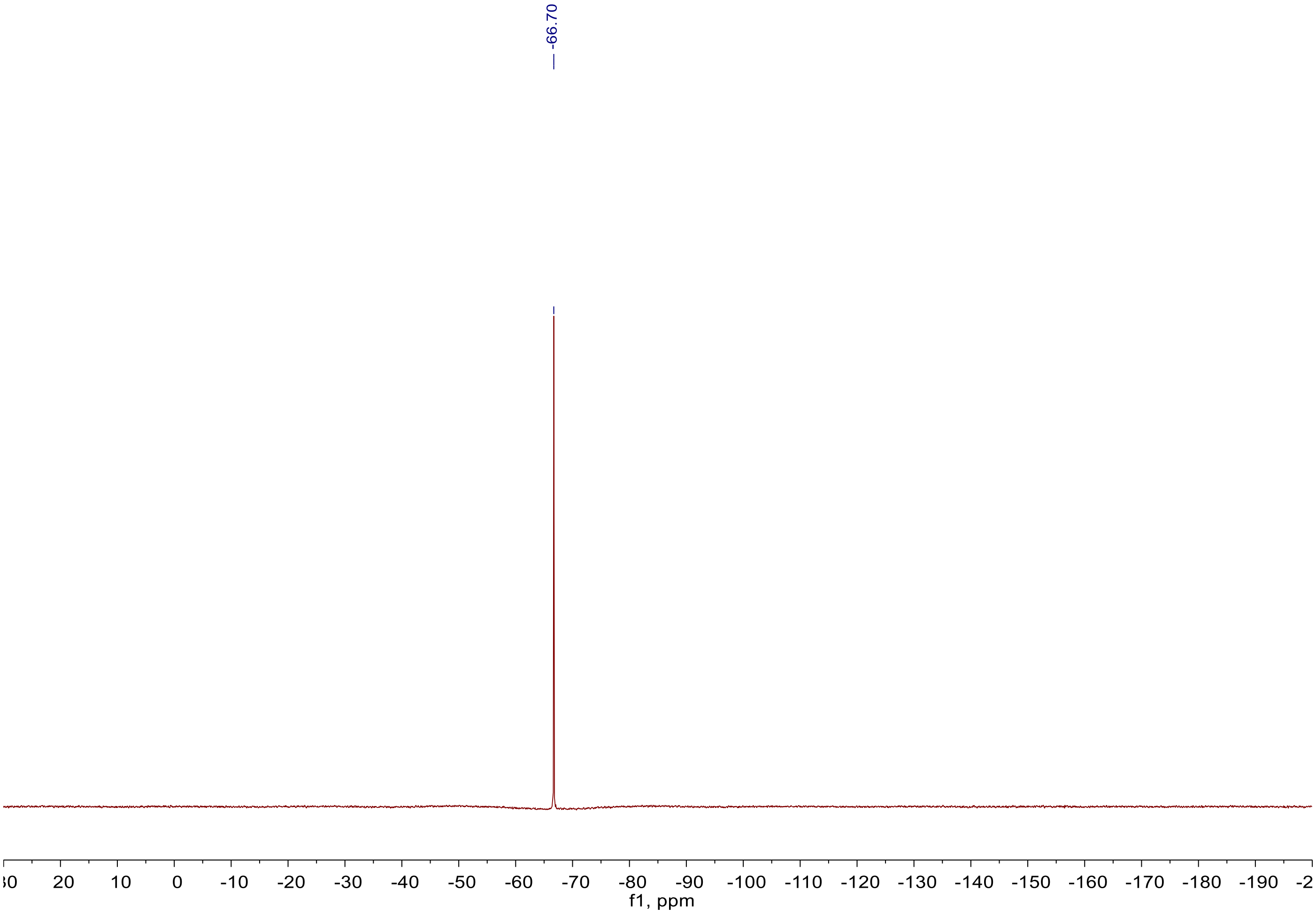

^{19}F spectrum of **3a** in $CDCl_3$

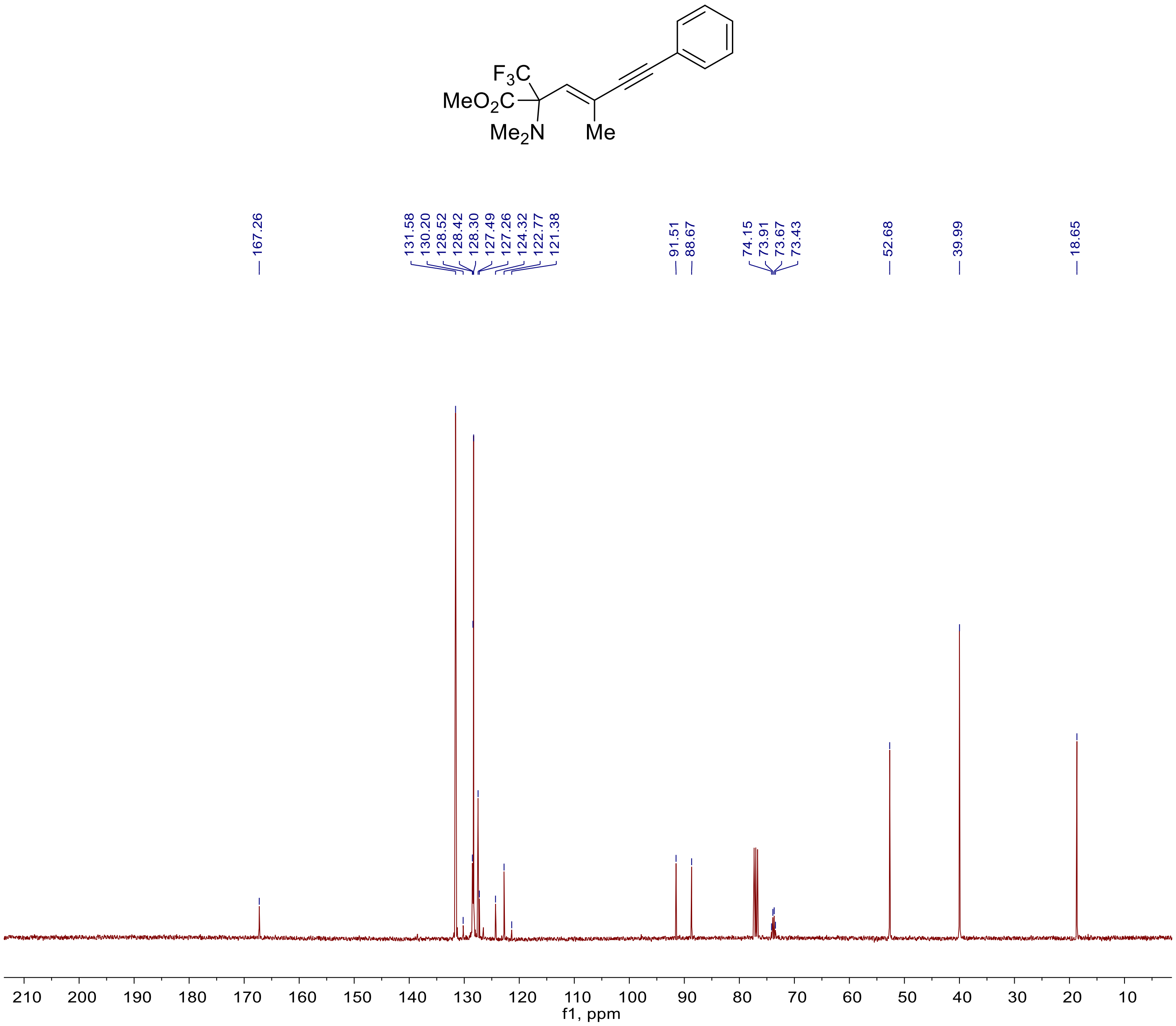

^{13}C spectrum of **3a** in $CDCl_3$

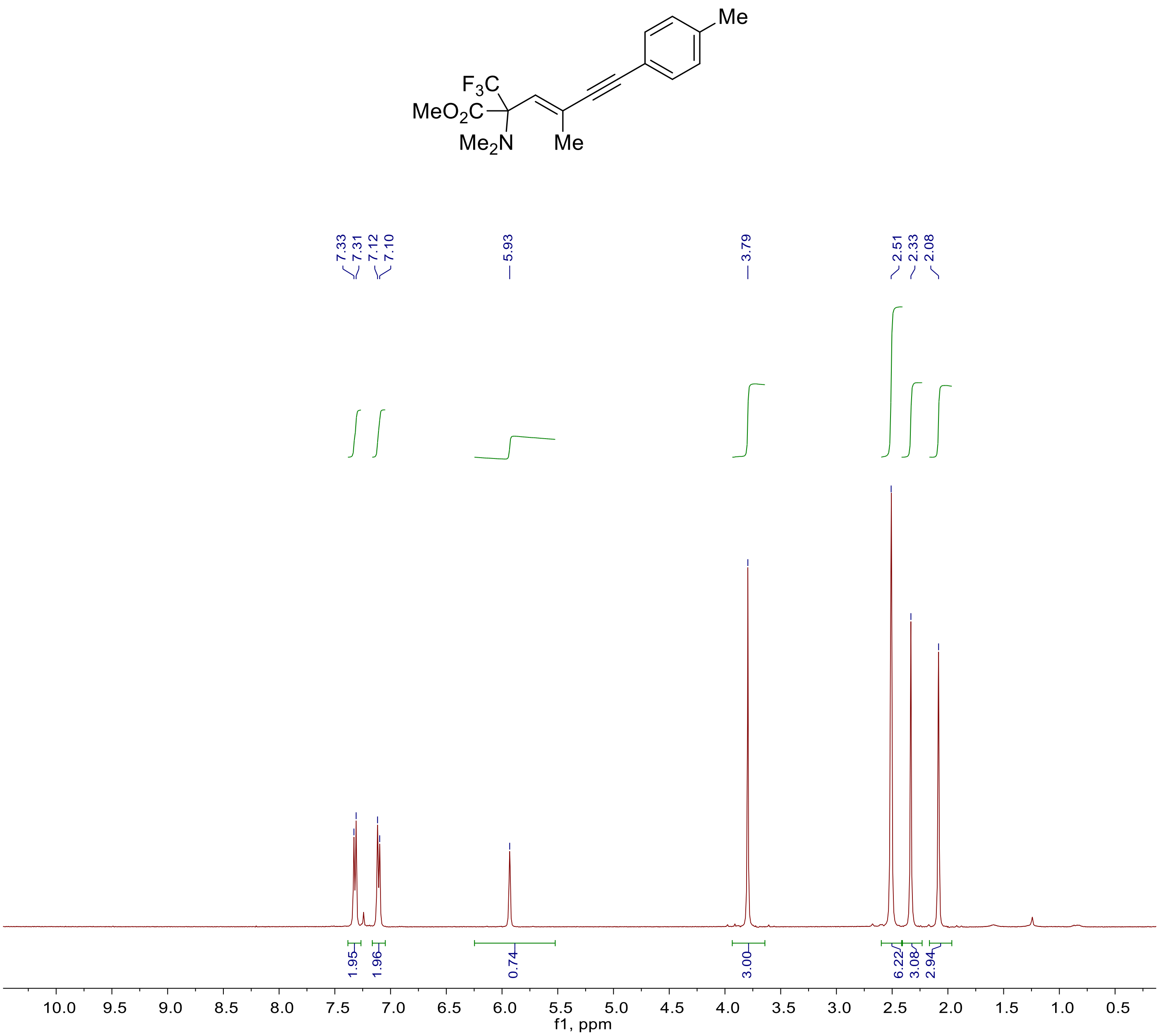

^{1}H spectrum of **3b** in $CDCl_3$

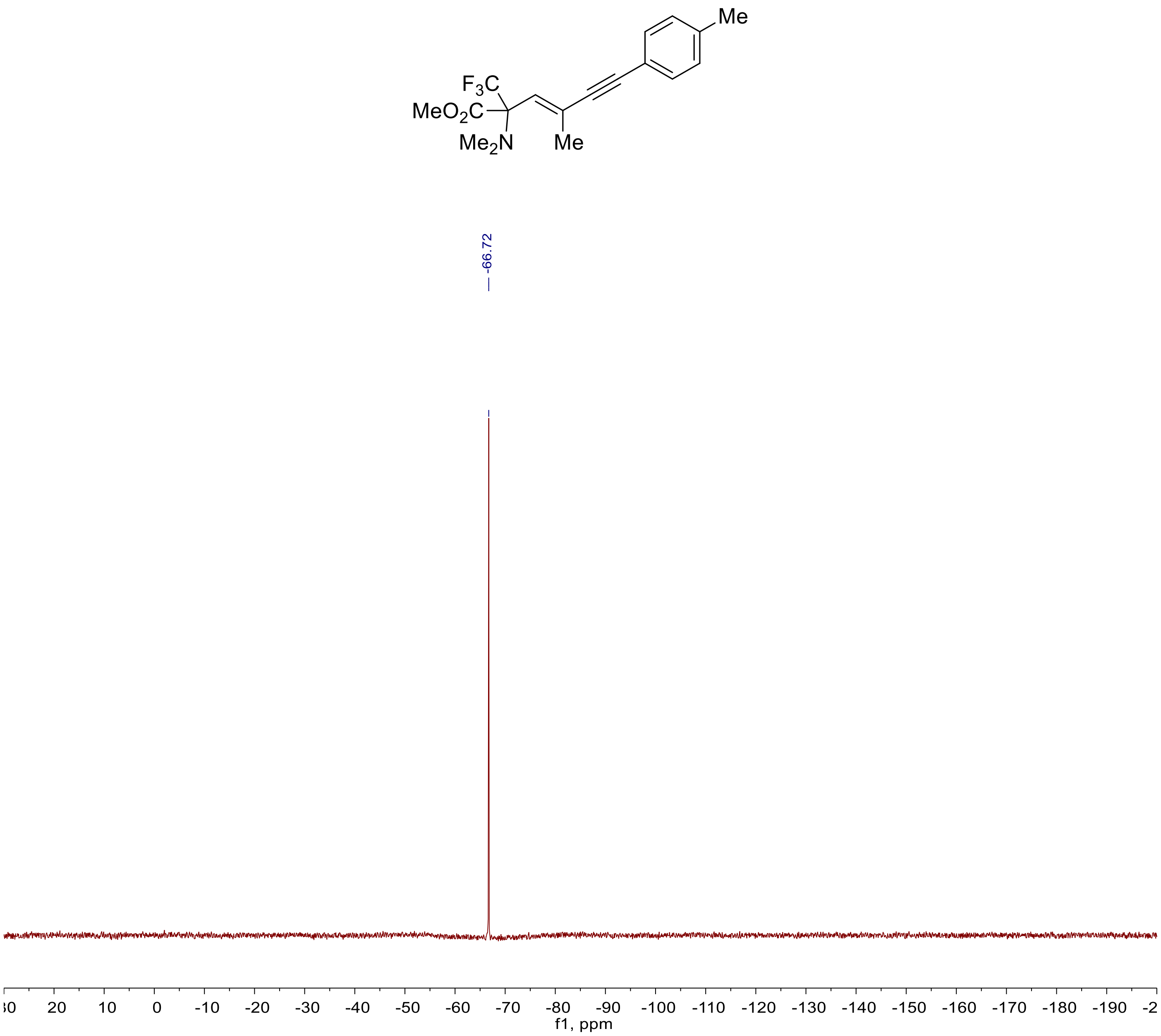

^{19}F spectrum of **3b** in $CDCl_3$

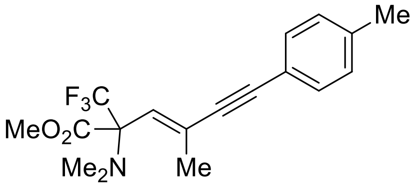

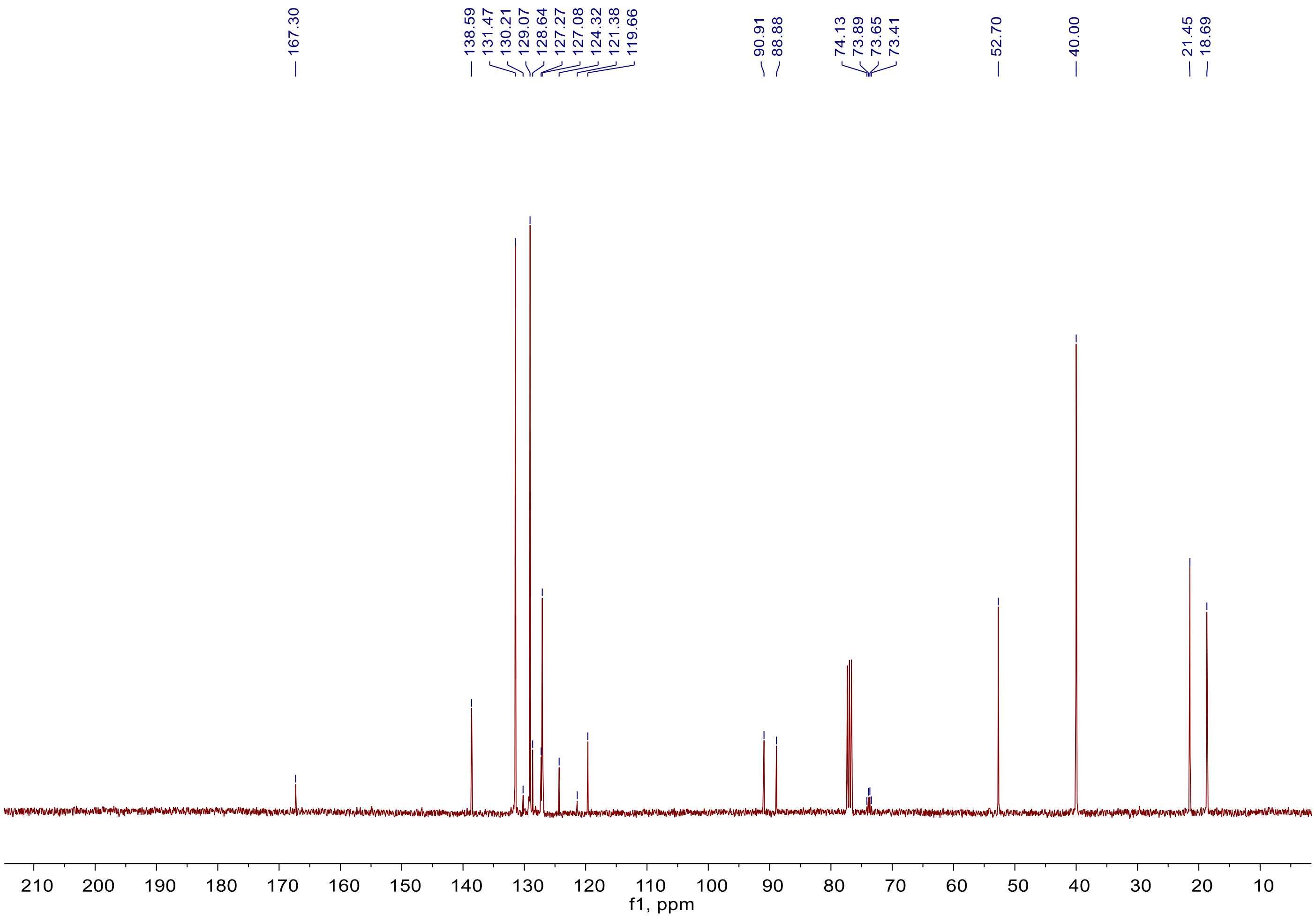

^{13}C spectrum of **3b** in $CDCl_3$

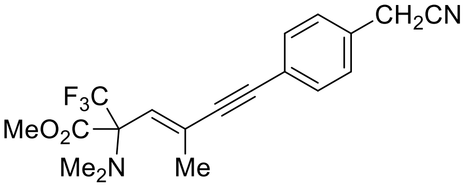

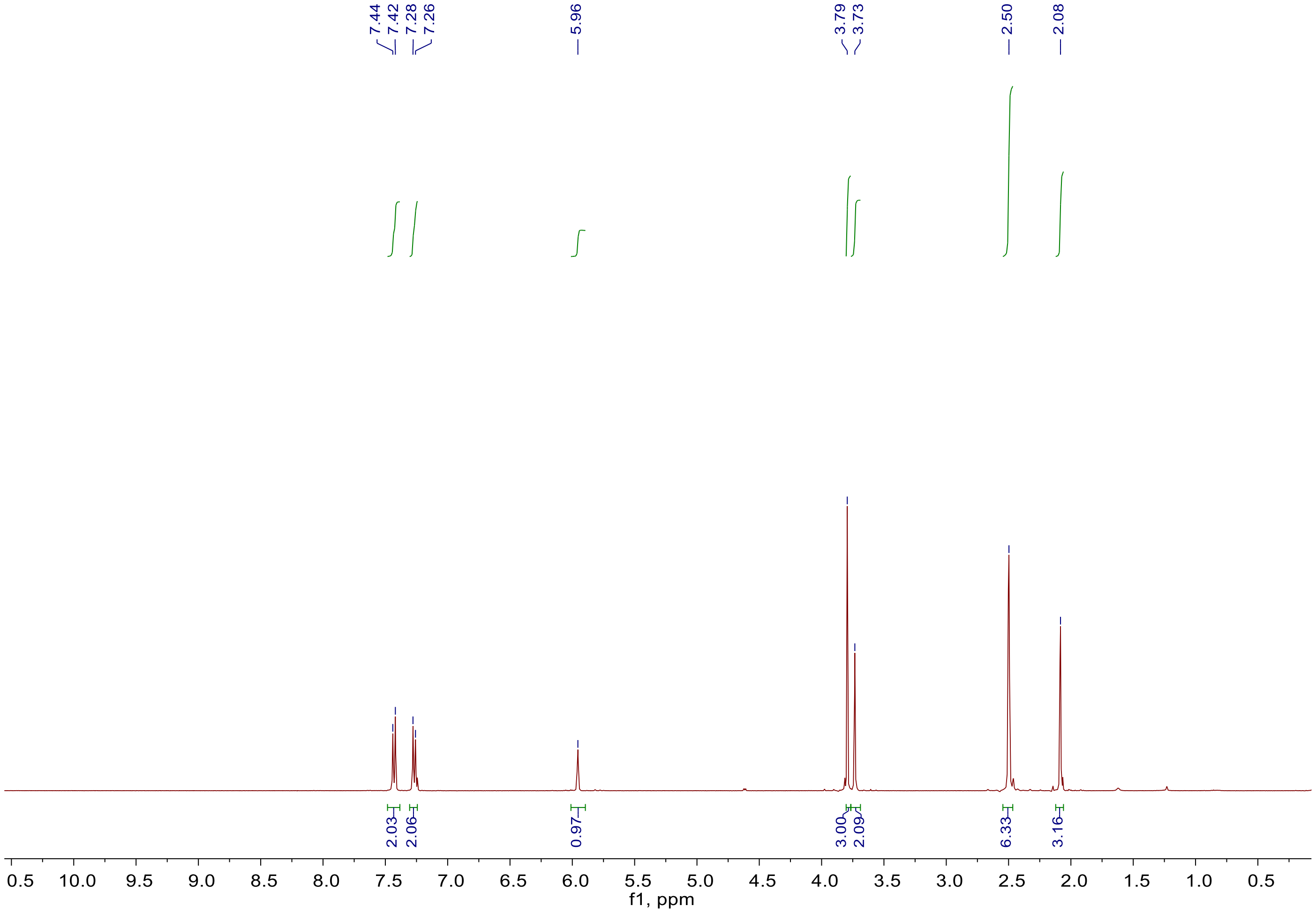

^{1}H spectrum of **3c** in $CDCl_3$

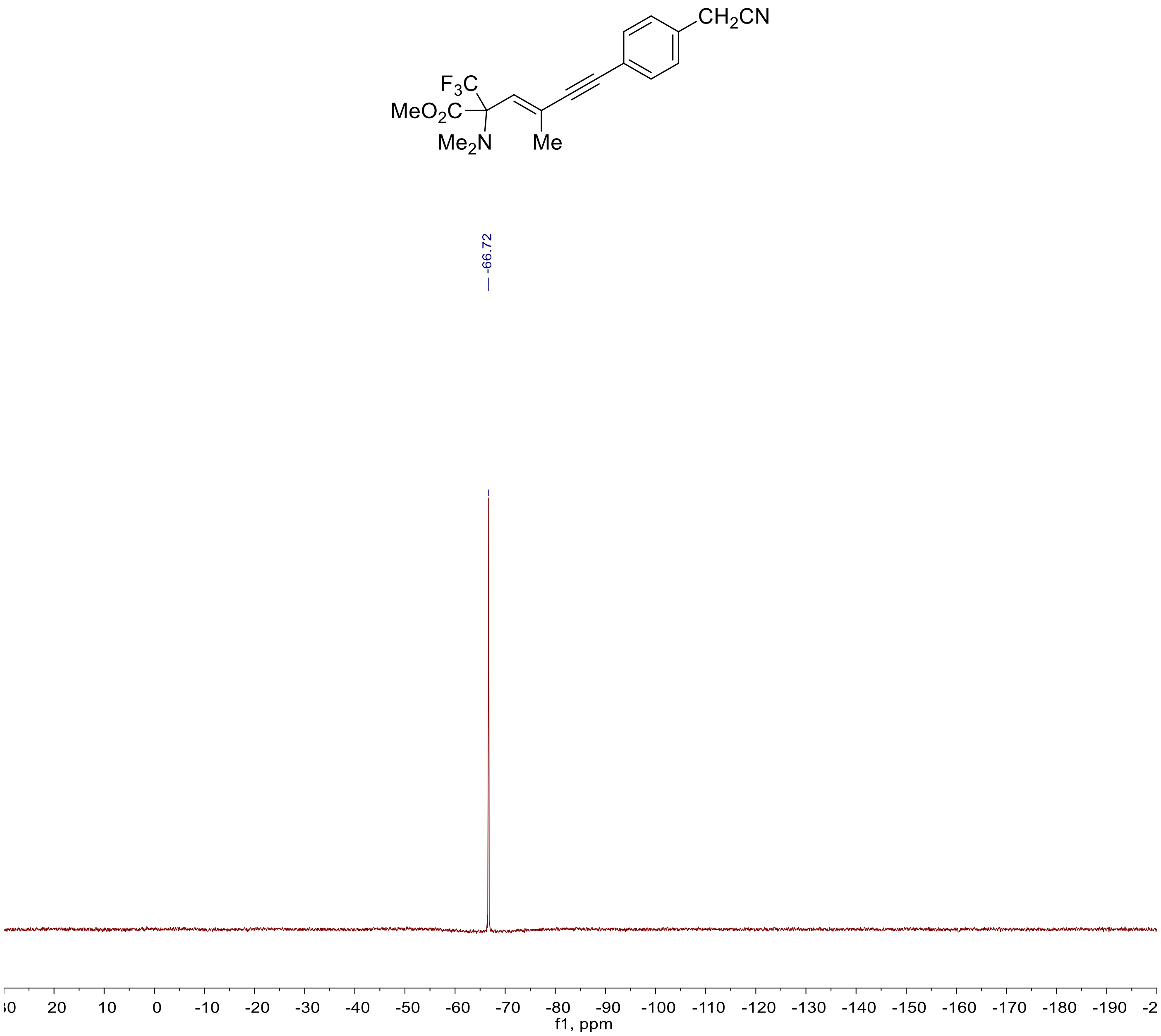

^{19}F spectrum of **3c** in $CDCl_3$

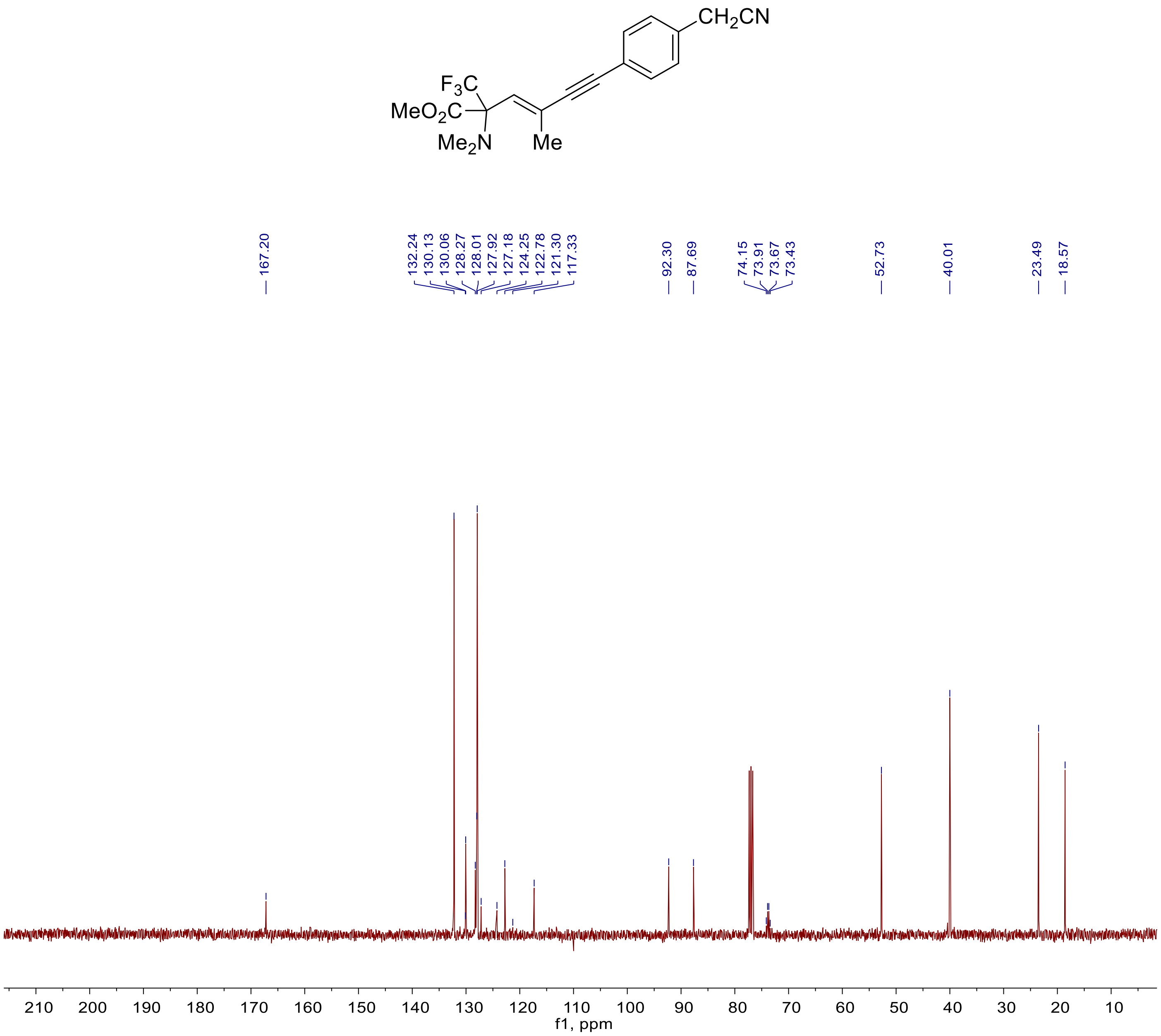

^{13}C spectrum of **3c** in $CDCl_3$

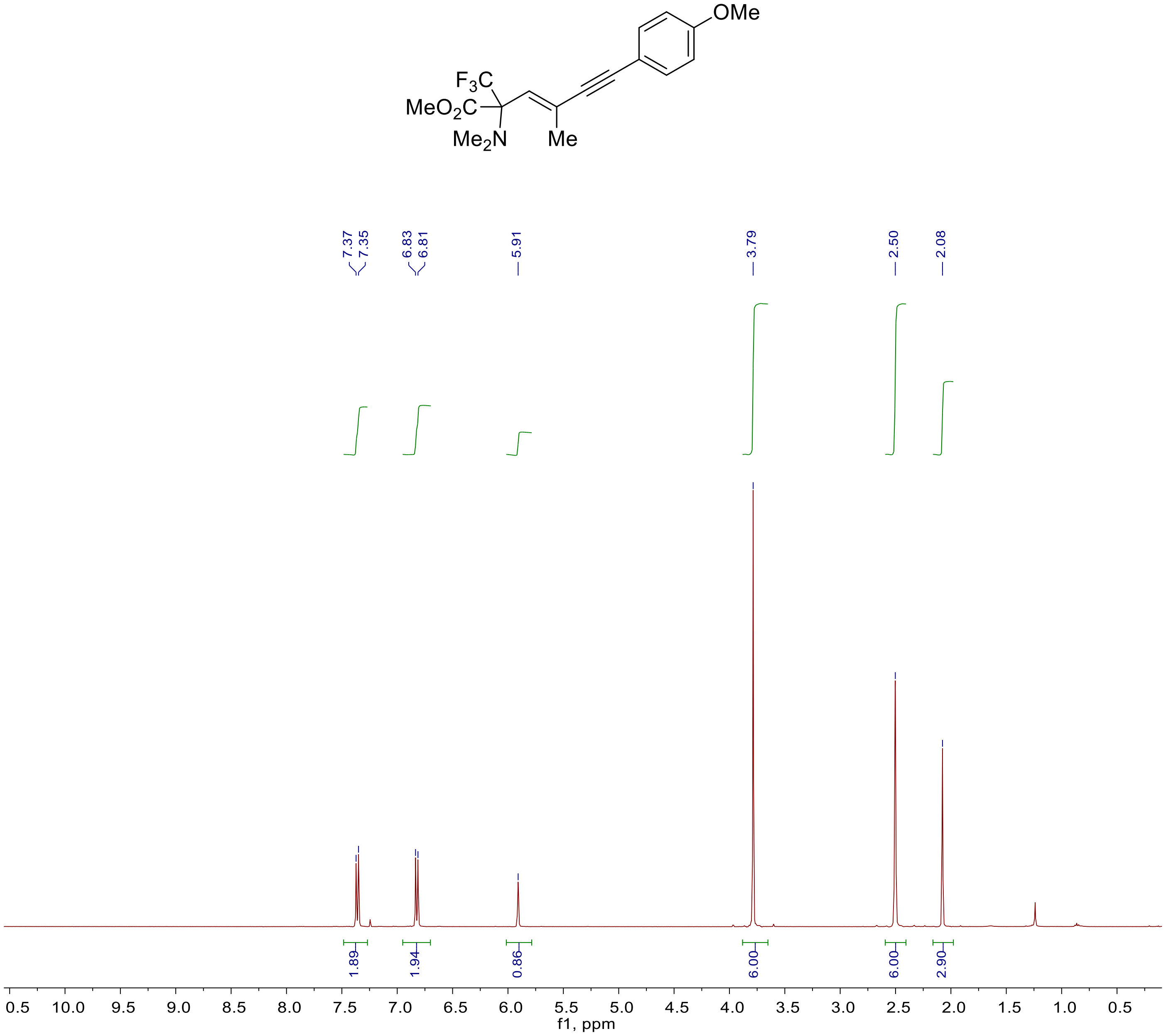

^{1}H spectrum of **3d** in $CDCl_3$

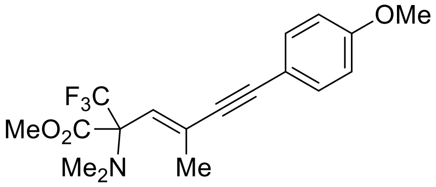

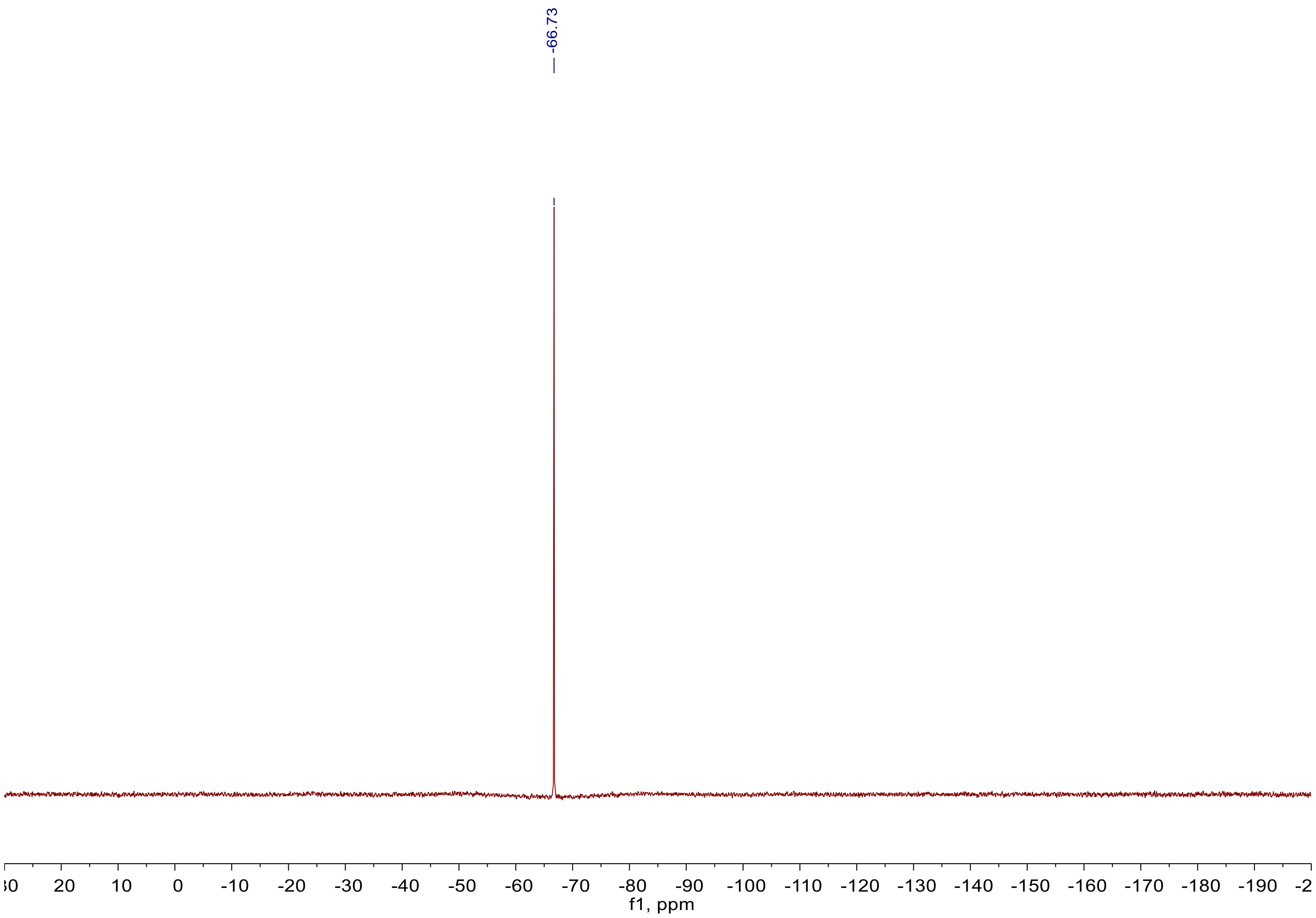

^{19}F spectrum of **3d** in $CDCl_3$

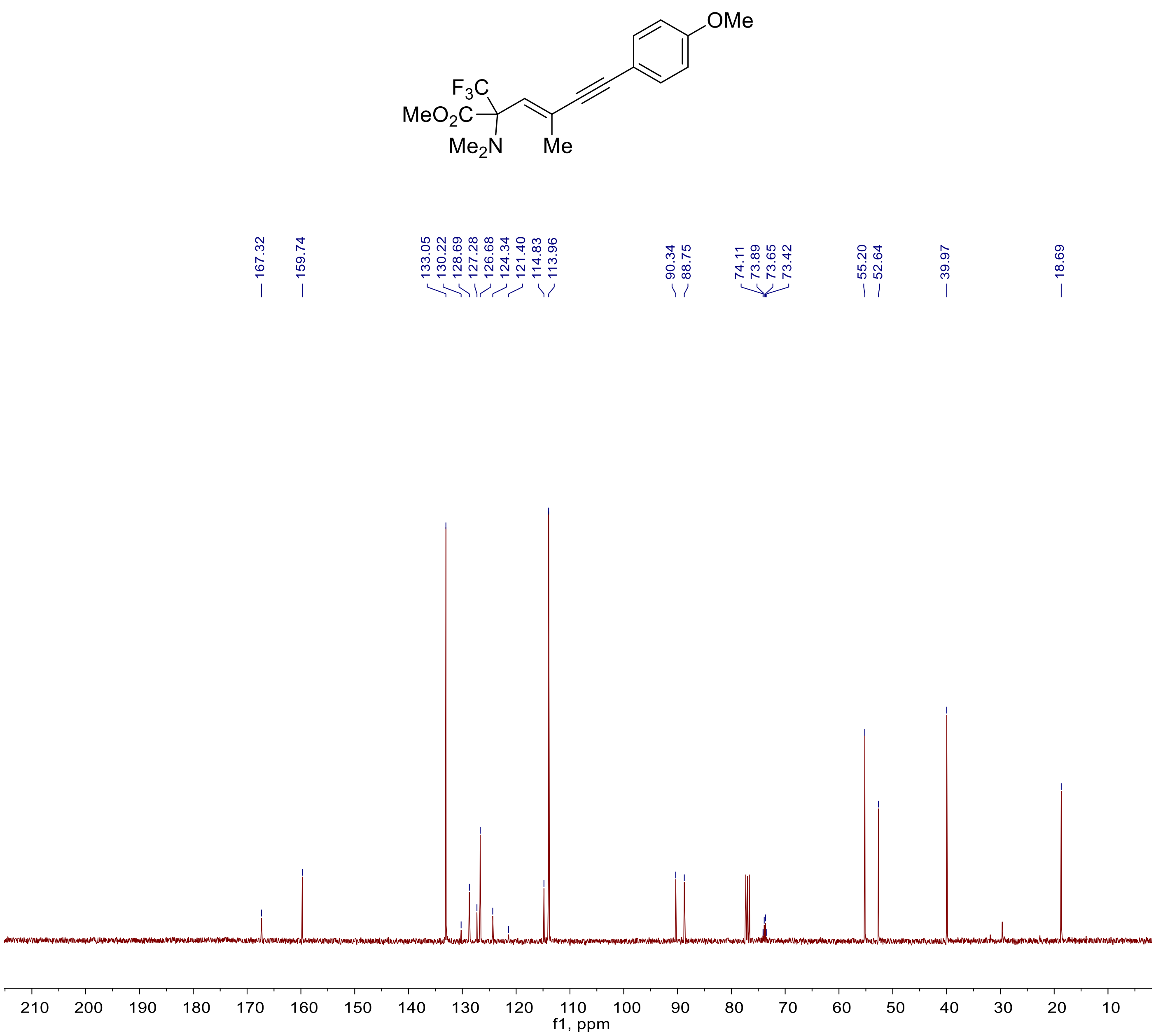

^{13}C spectrum of **3d** in $CDCl_3$

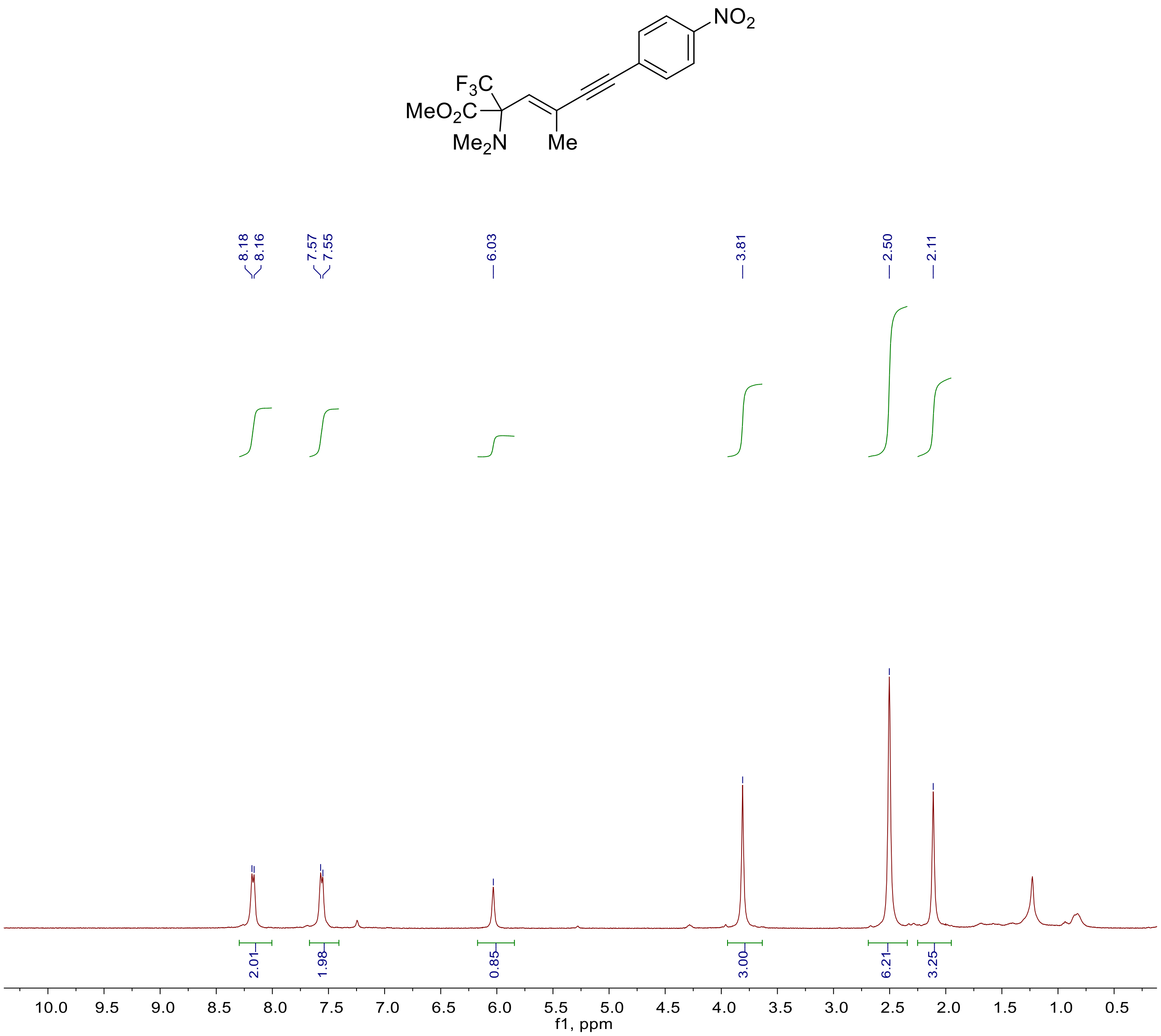

^{1}H spectrum of **3e** in $CDCl_3$

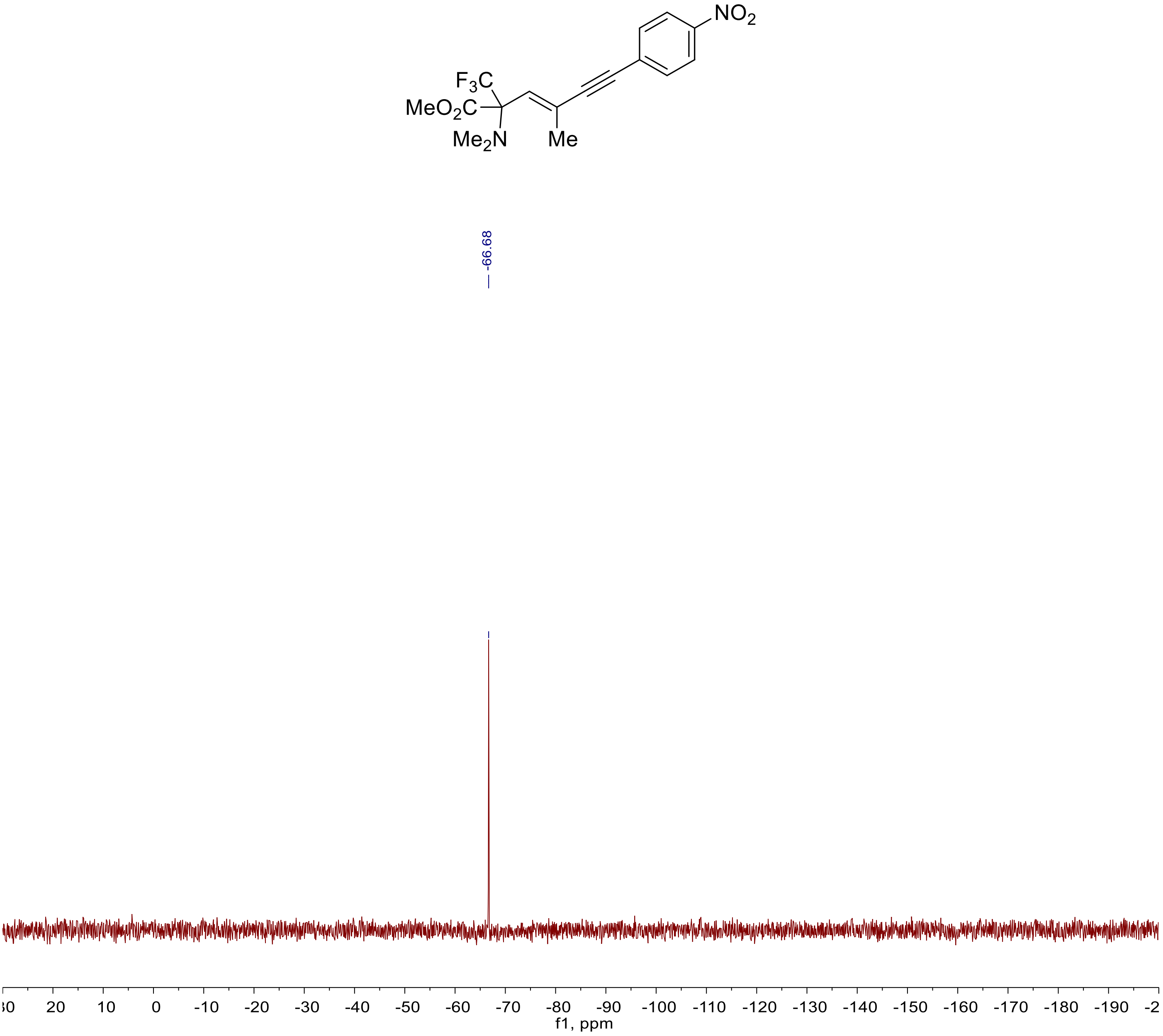

^{19}F spectrum of **3e** in $CDCl_3$

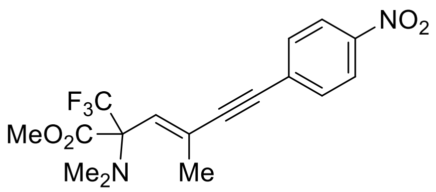

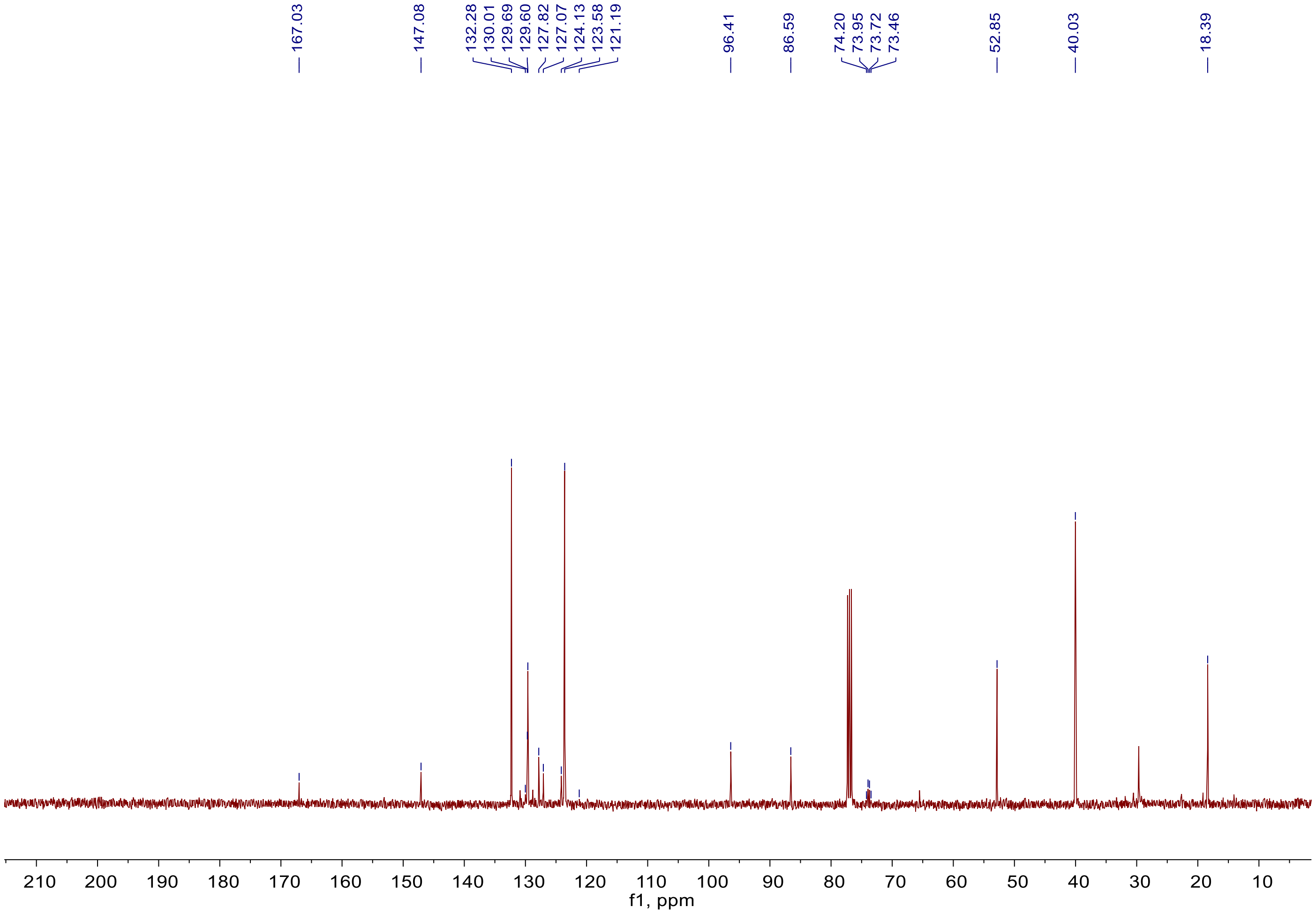

^{13}C spectrum of **3e** in $CDCl_3$

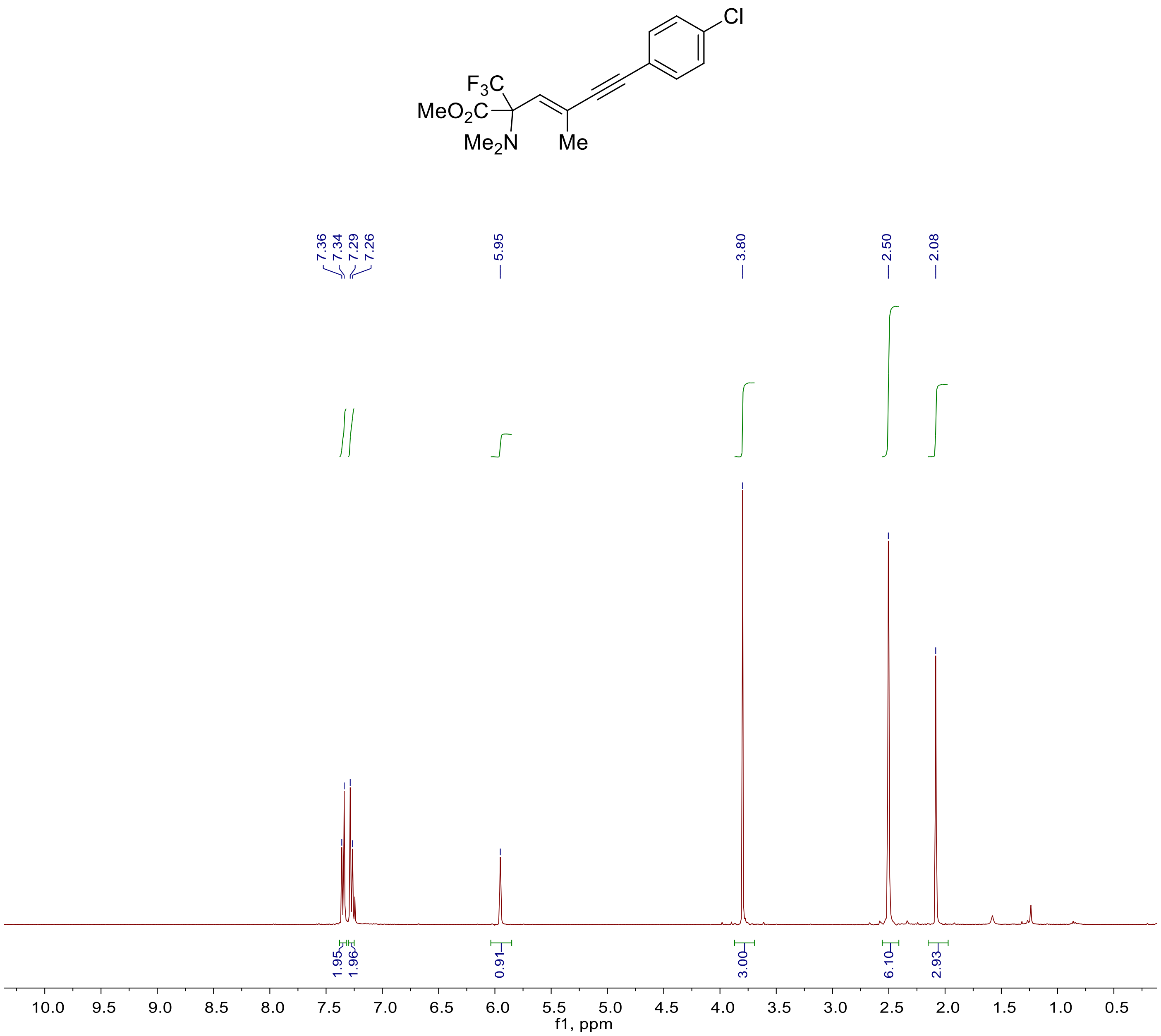

^{1}H spectrum of **3f** in $CDCl_3$

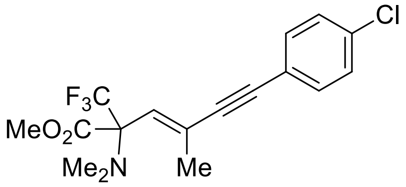

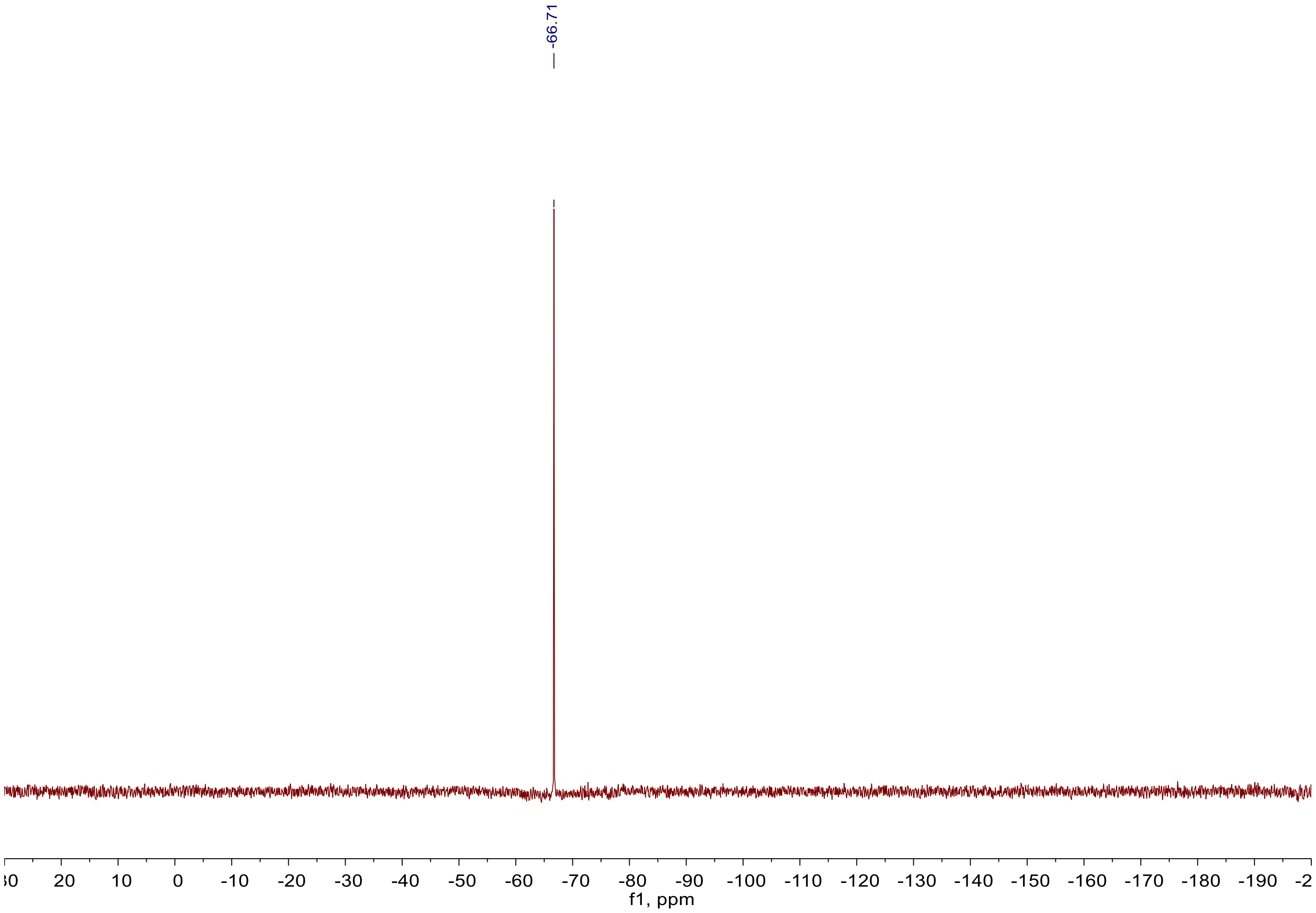

^{19}F spectrum of **3f** in $CDCl_3$

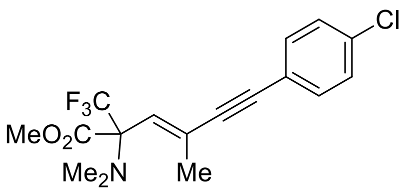

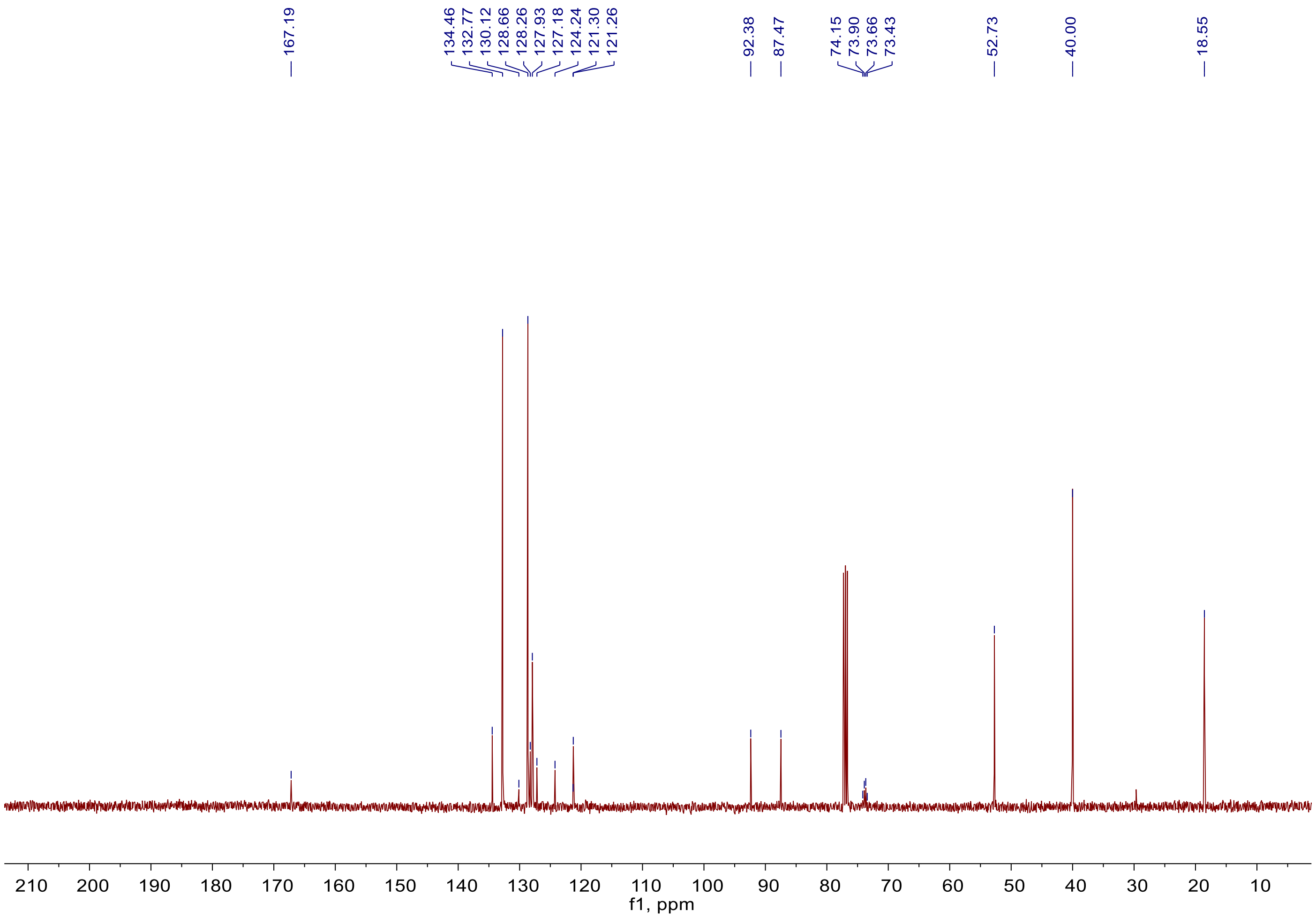

^{13}C spectrum of **3f** in $CDCl_3$

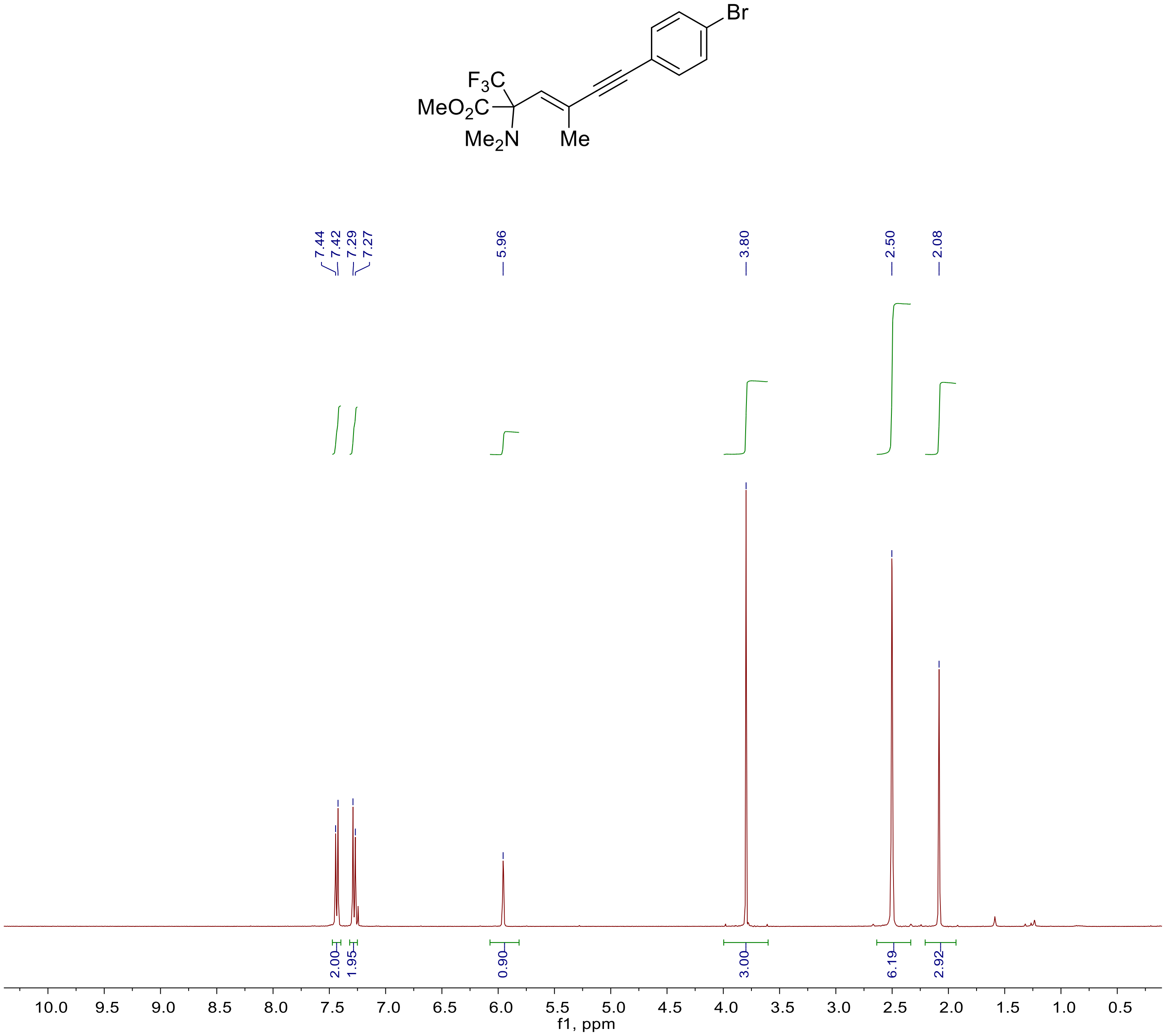

^{1}H spectrum of **3g** in $CDCl_3$

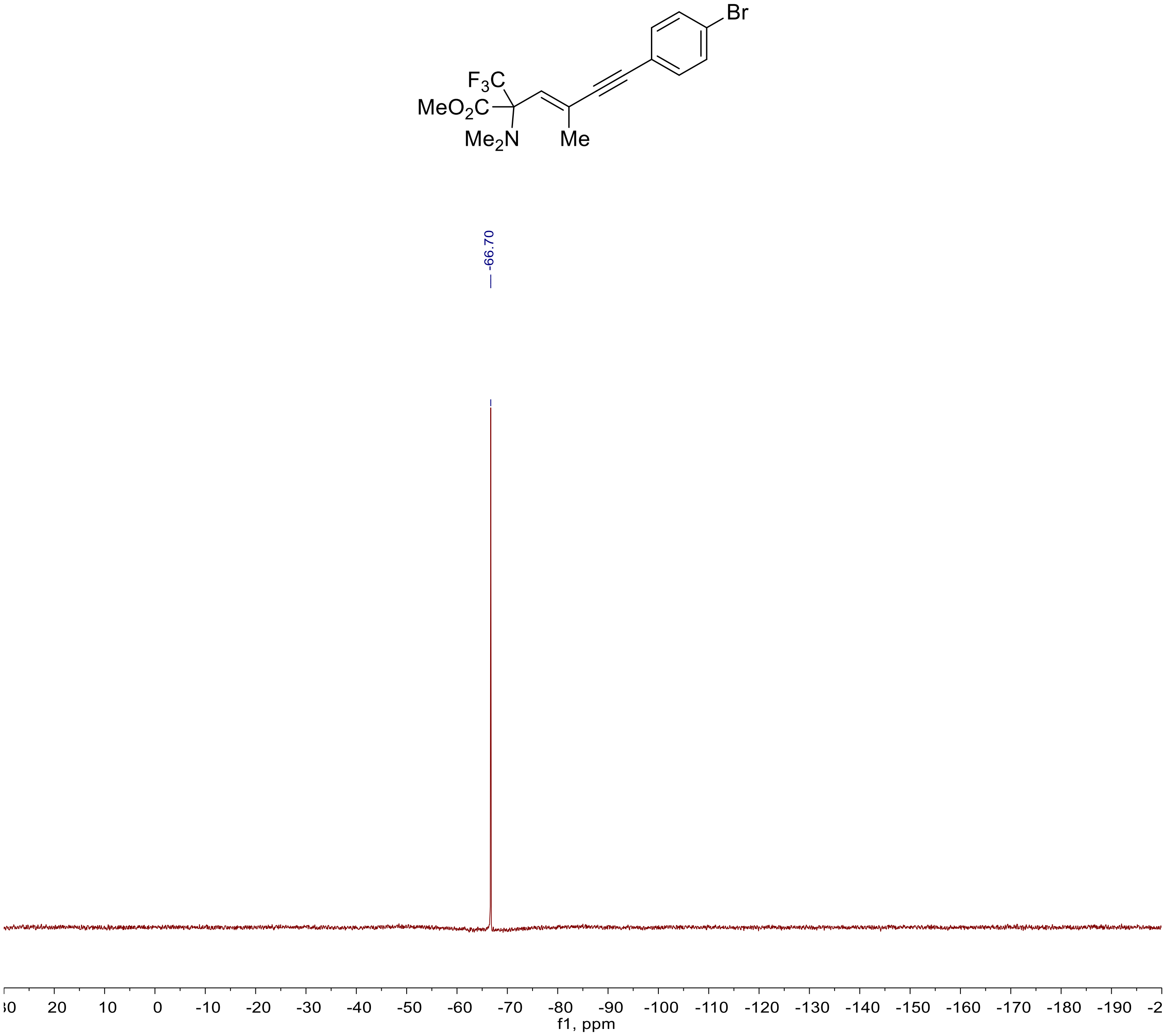

^{19}F spectrum of **3g** in $CDCl_3$

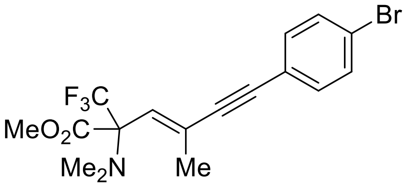

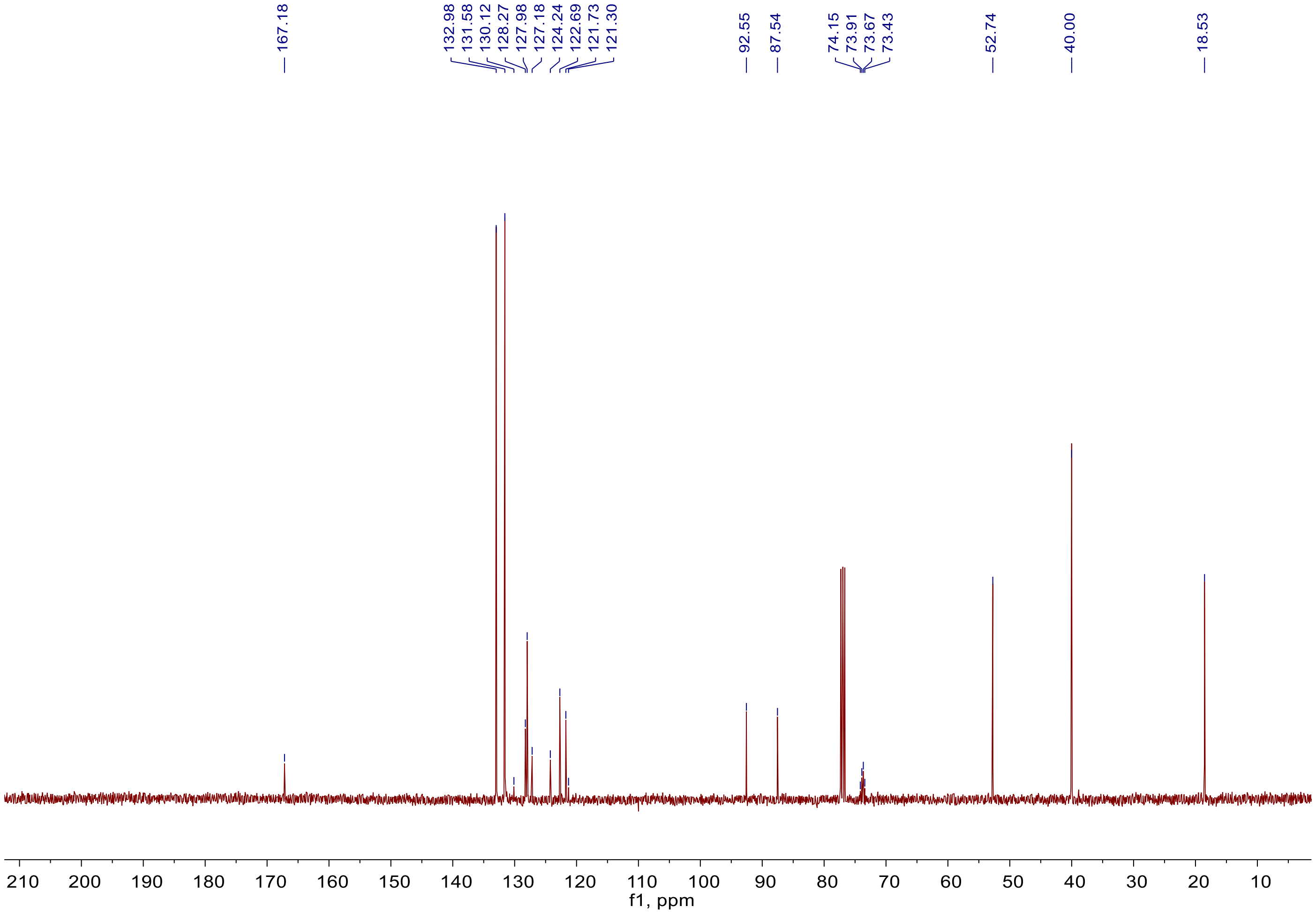

^{13}C spectrum of **3g** in $CDCl_3$

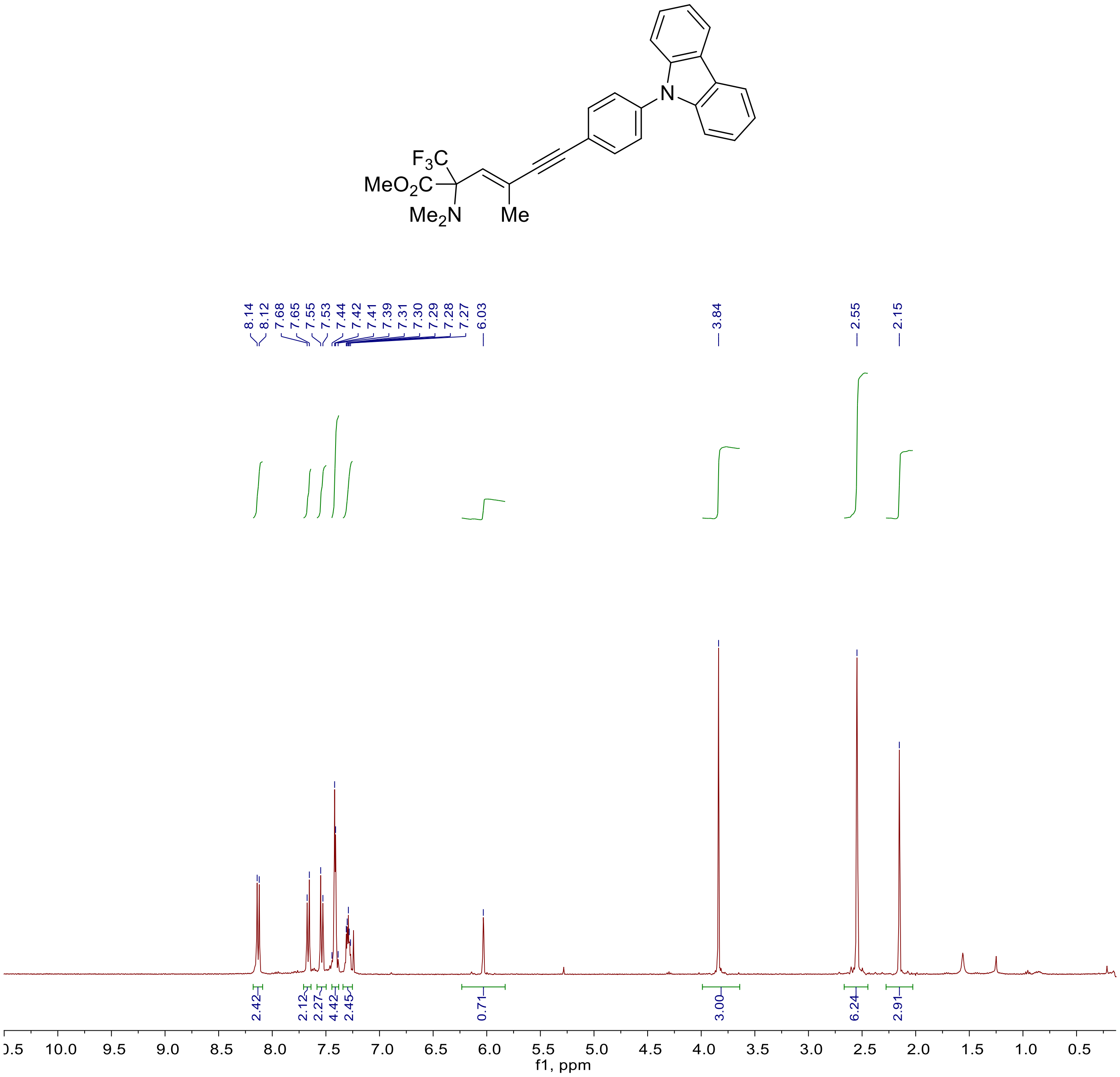

^{1}H spectrum of **3h** in $CDCl_3$

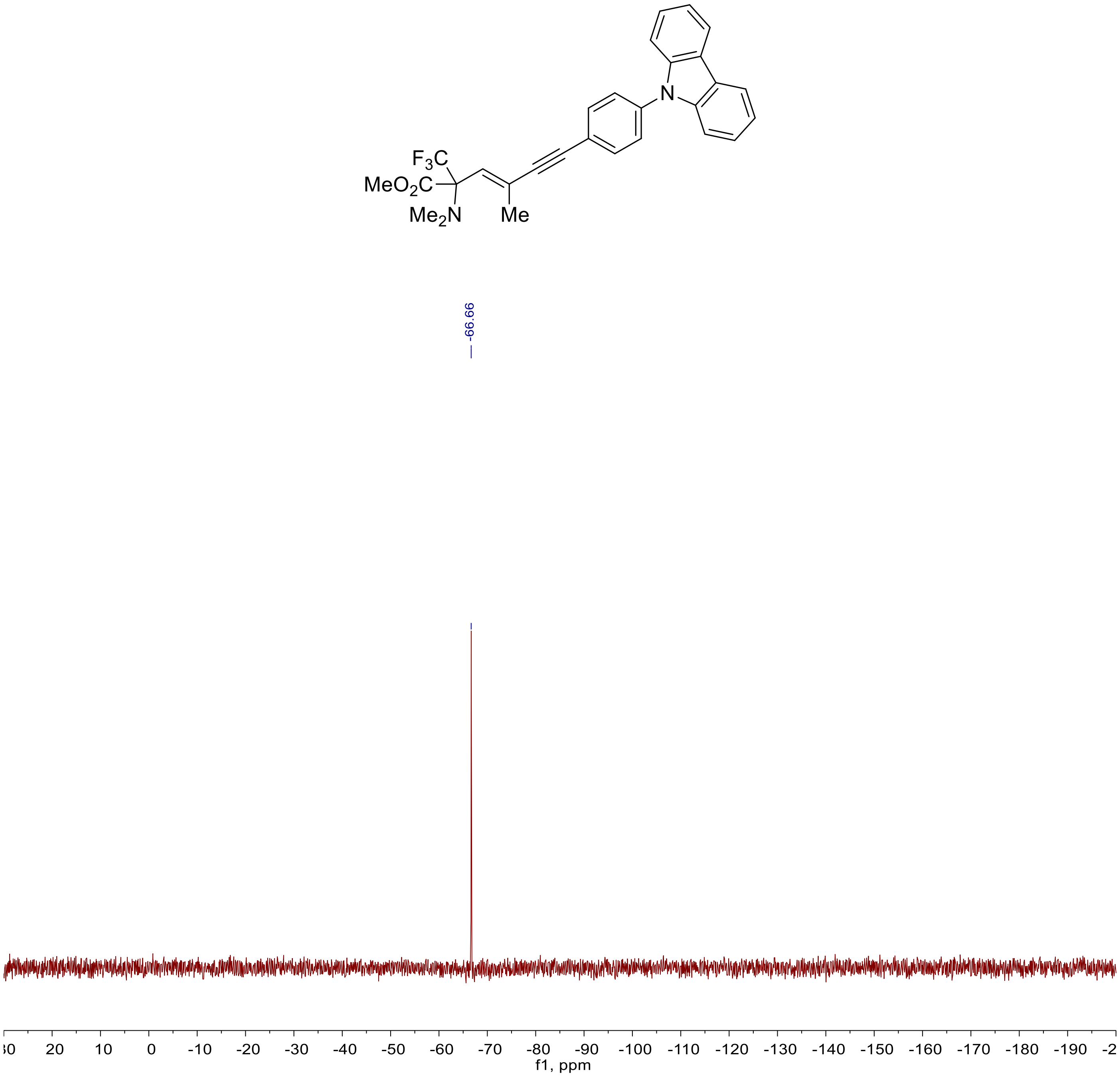

^{19}F spectrum of **3h** in $CDCl_3$

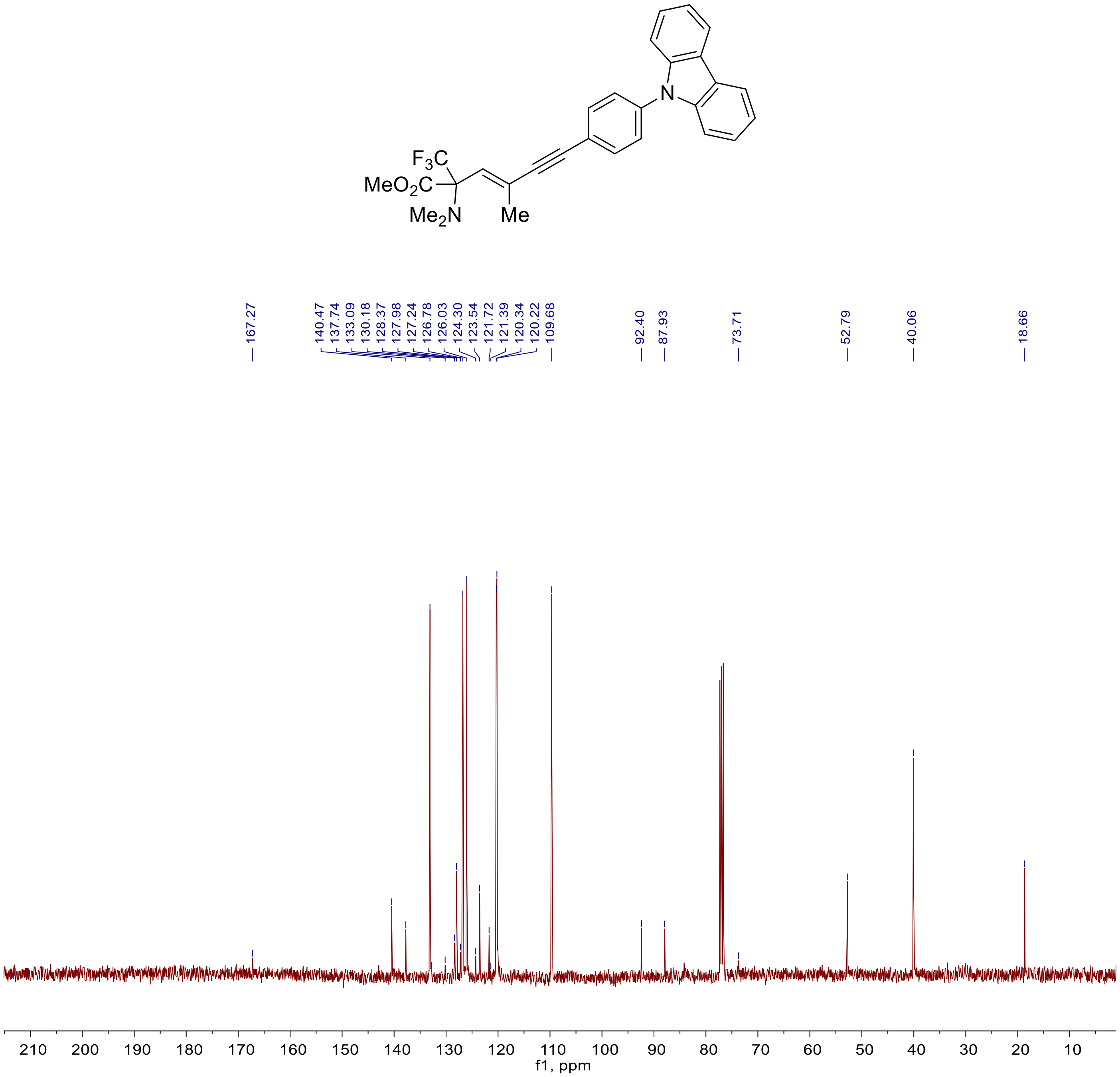

^{13}C spectrum of **3h** in $CDCl_3$

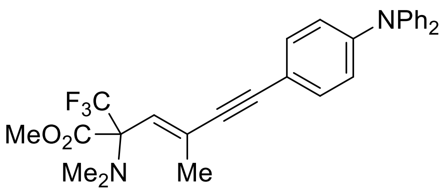

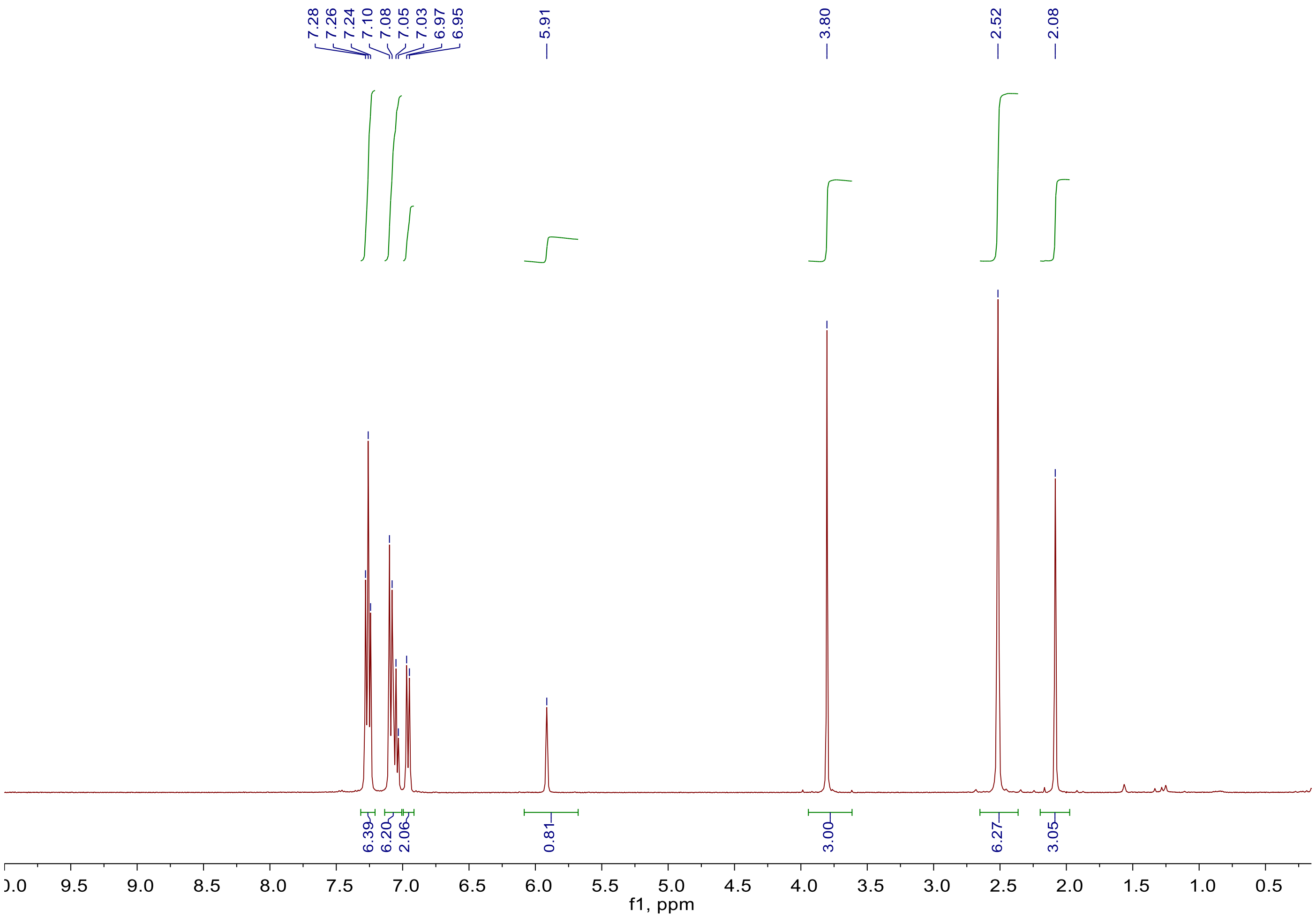

^{1}H spectrum of **3i** in $CDCl_3$

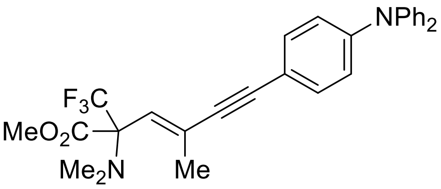

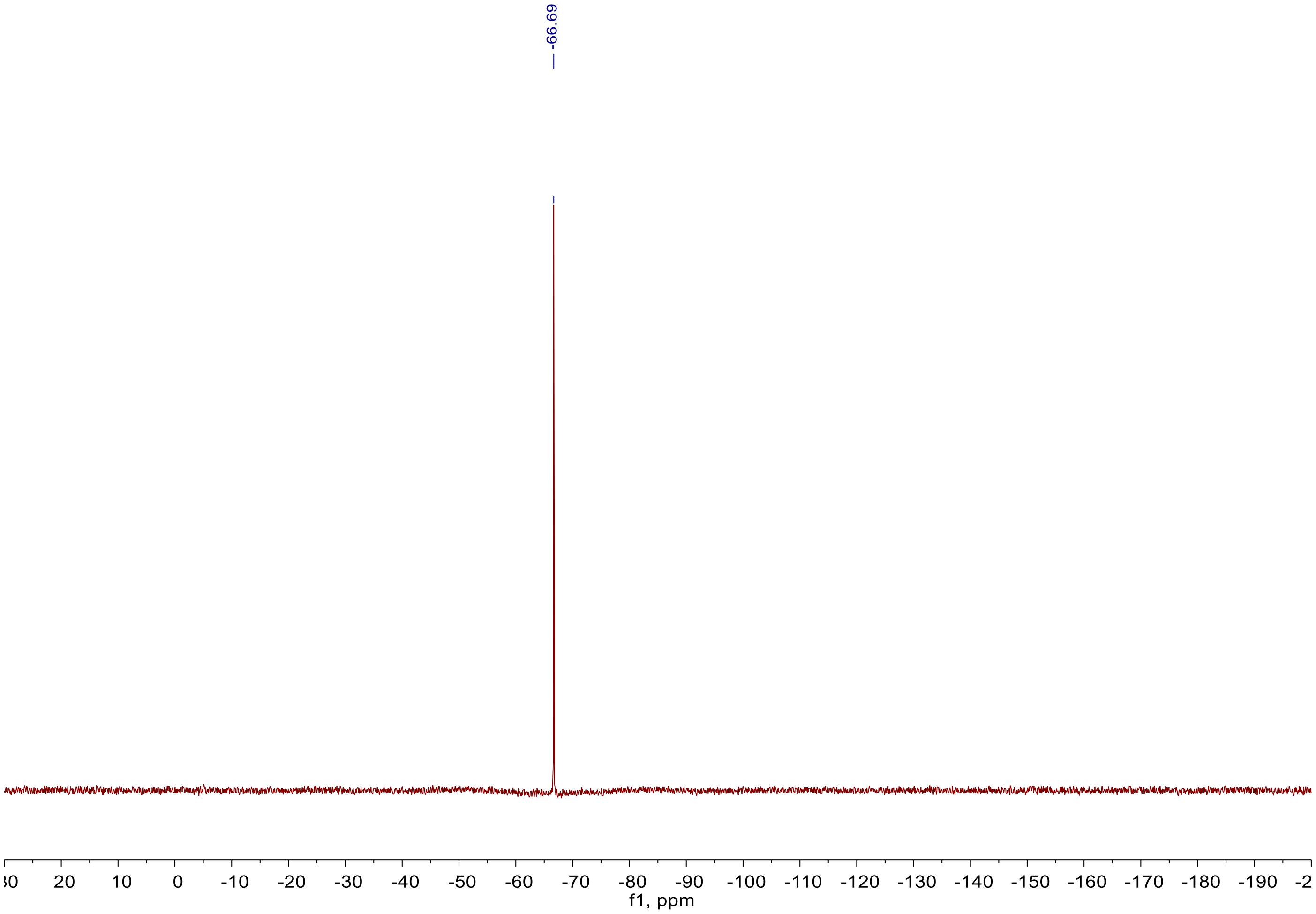

^{19}F spectrum of **3i** in $CDCl_3$

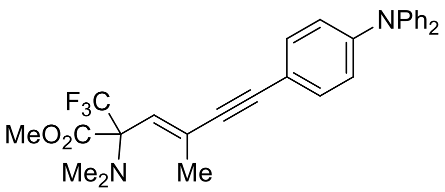

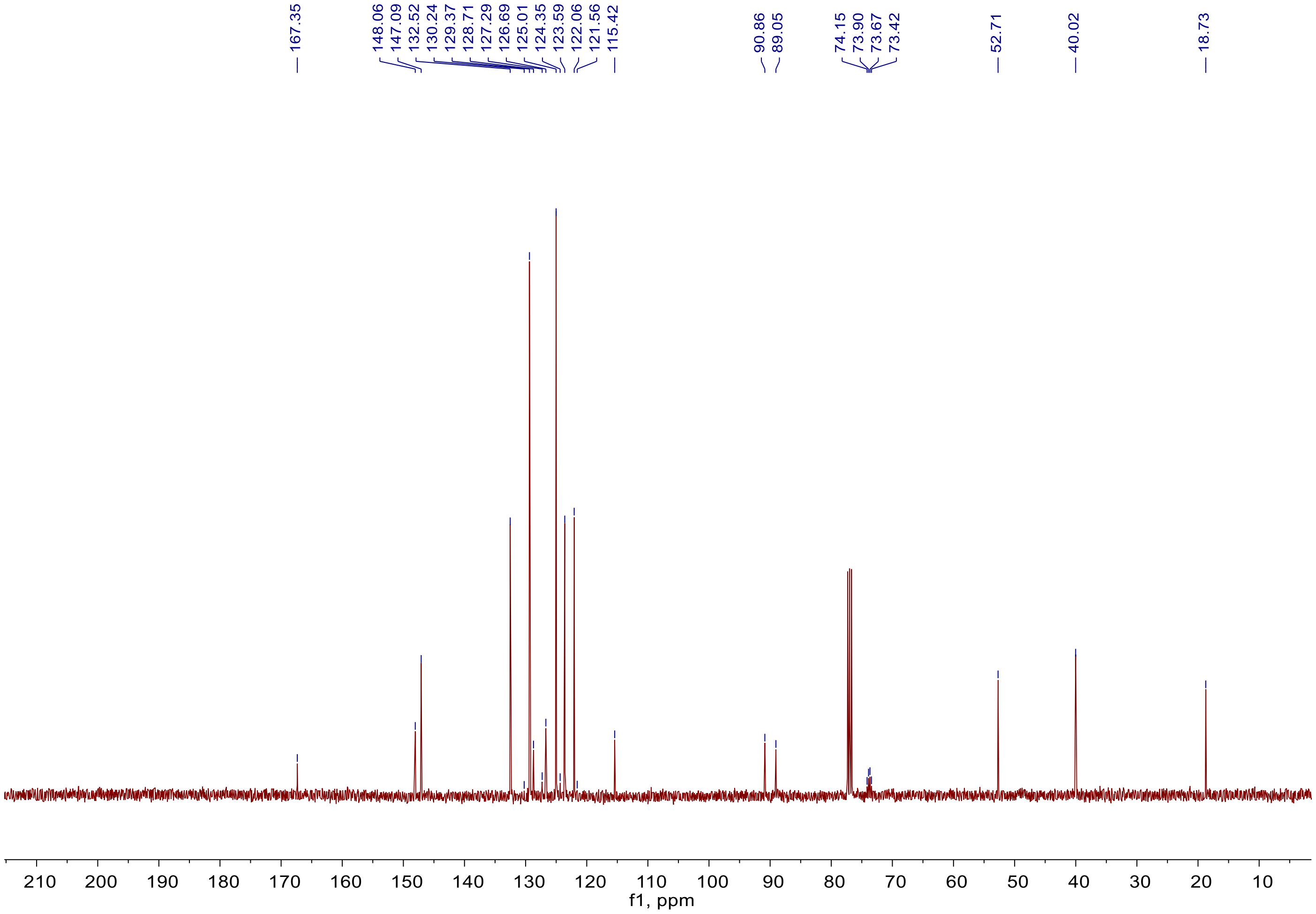

^{13}C spectrum of **3i** in $CDCl_3$

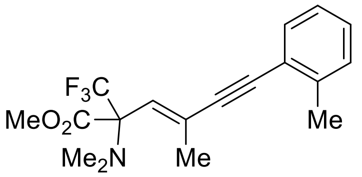

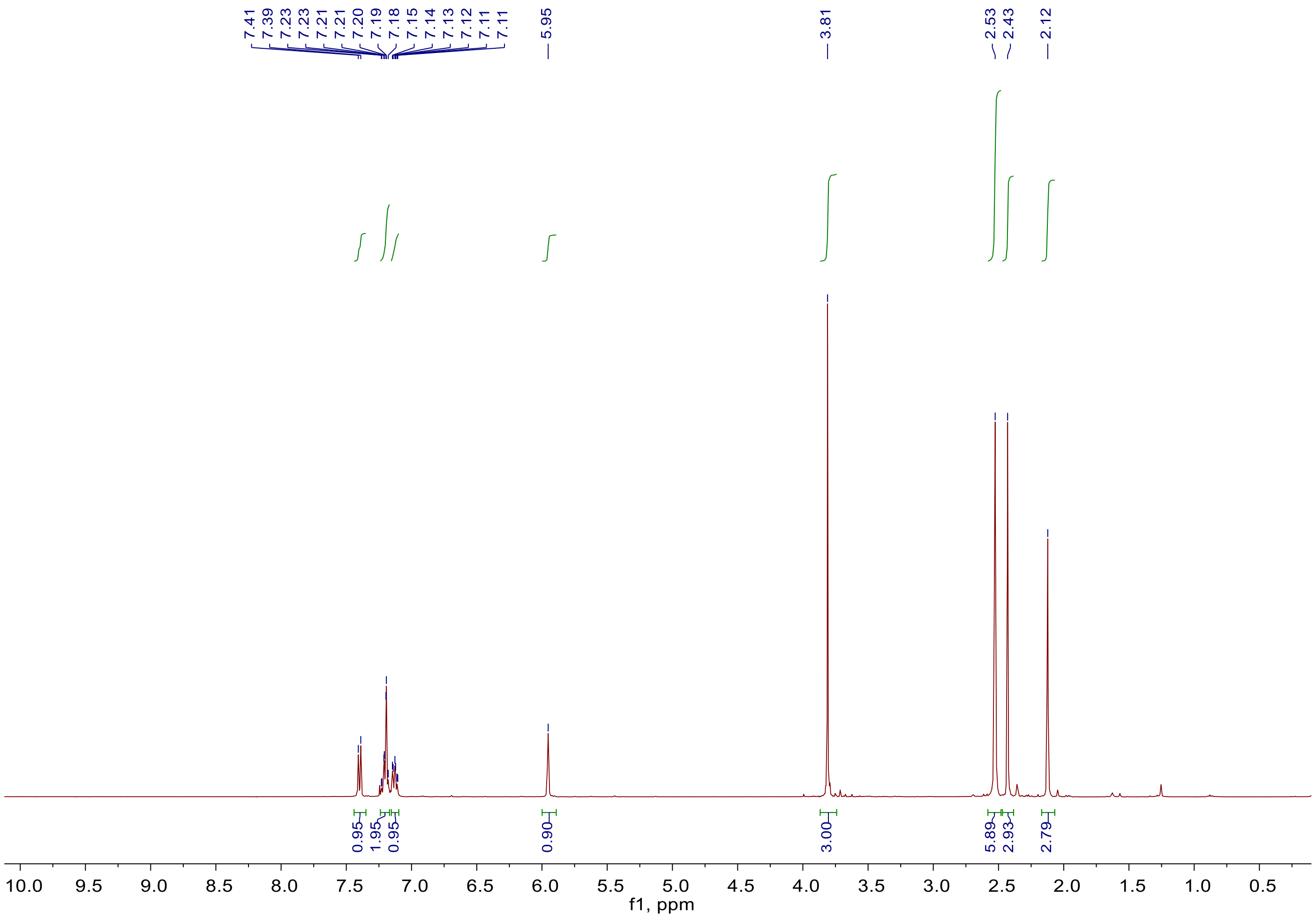

^{1}H spectrum of **3j** in $CDCl_3$

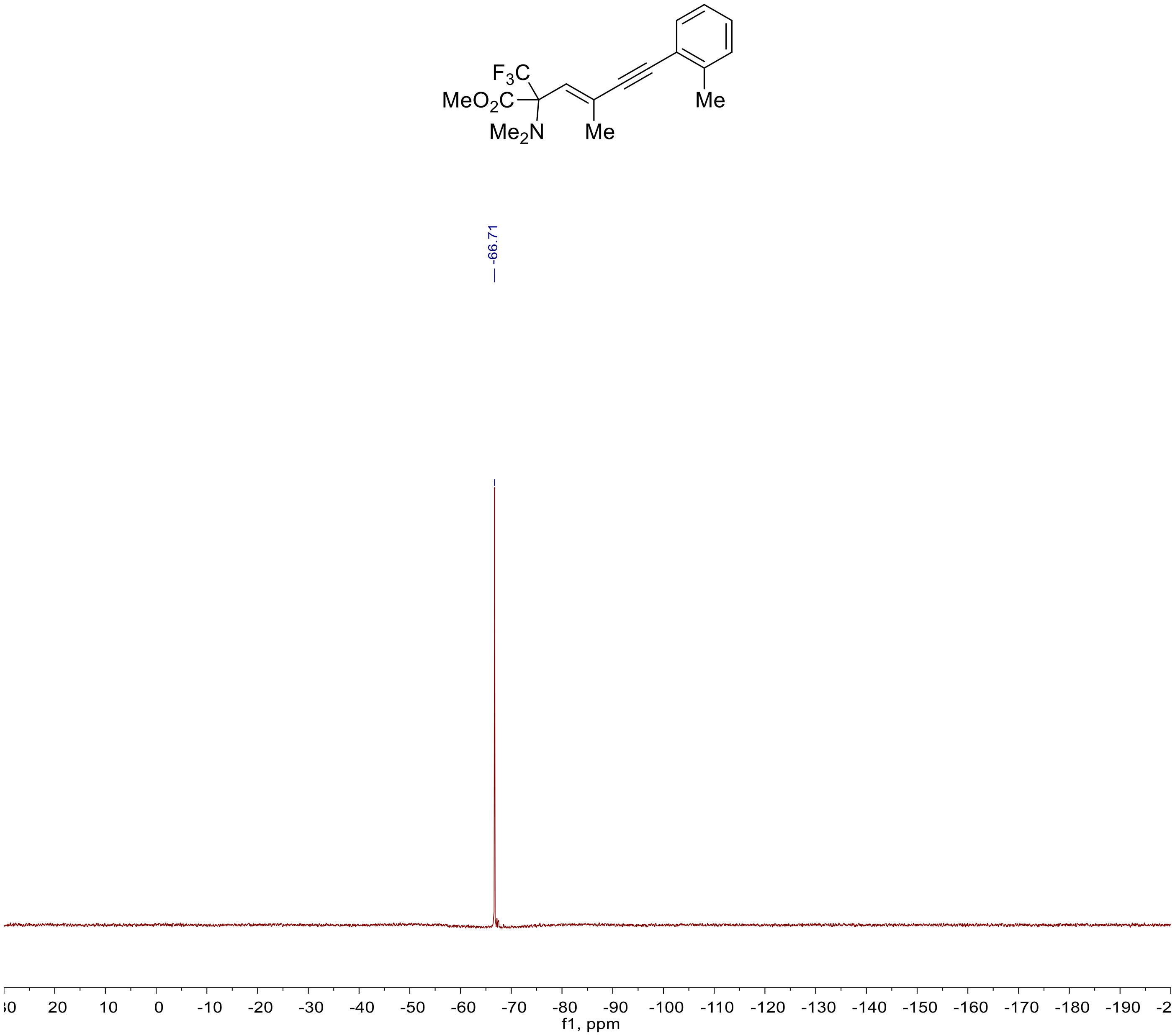

^{19}F spectrum of **3j** in $CDCl_3$

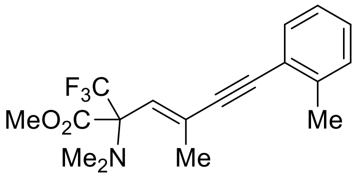

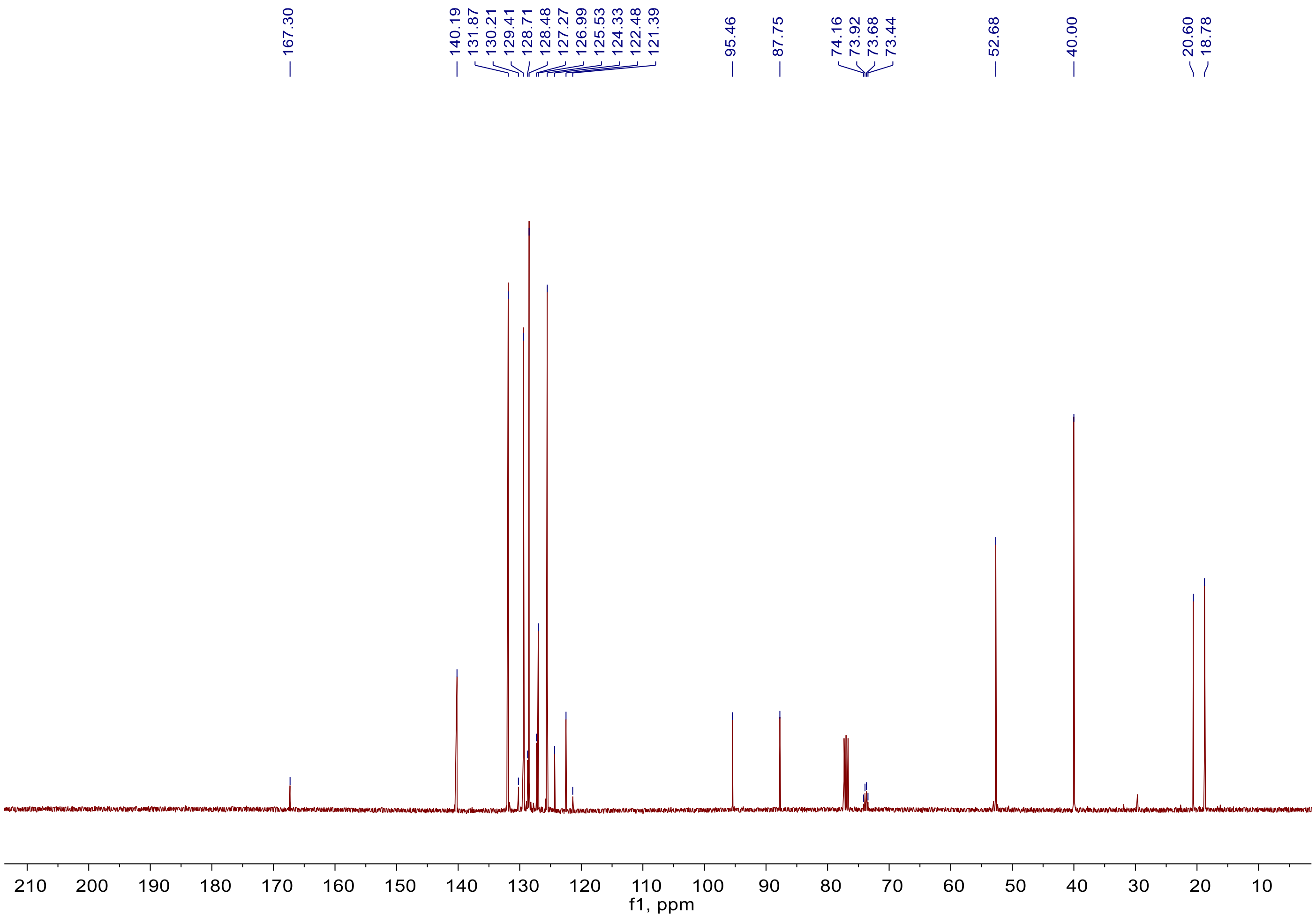

^{13}C spectrum of **3j** in $CDCl_3$

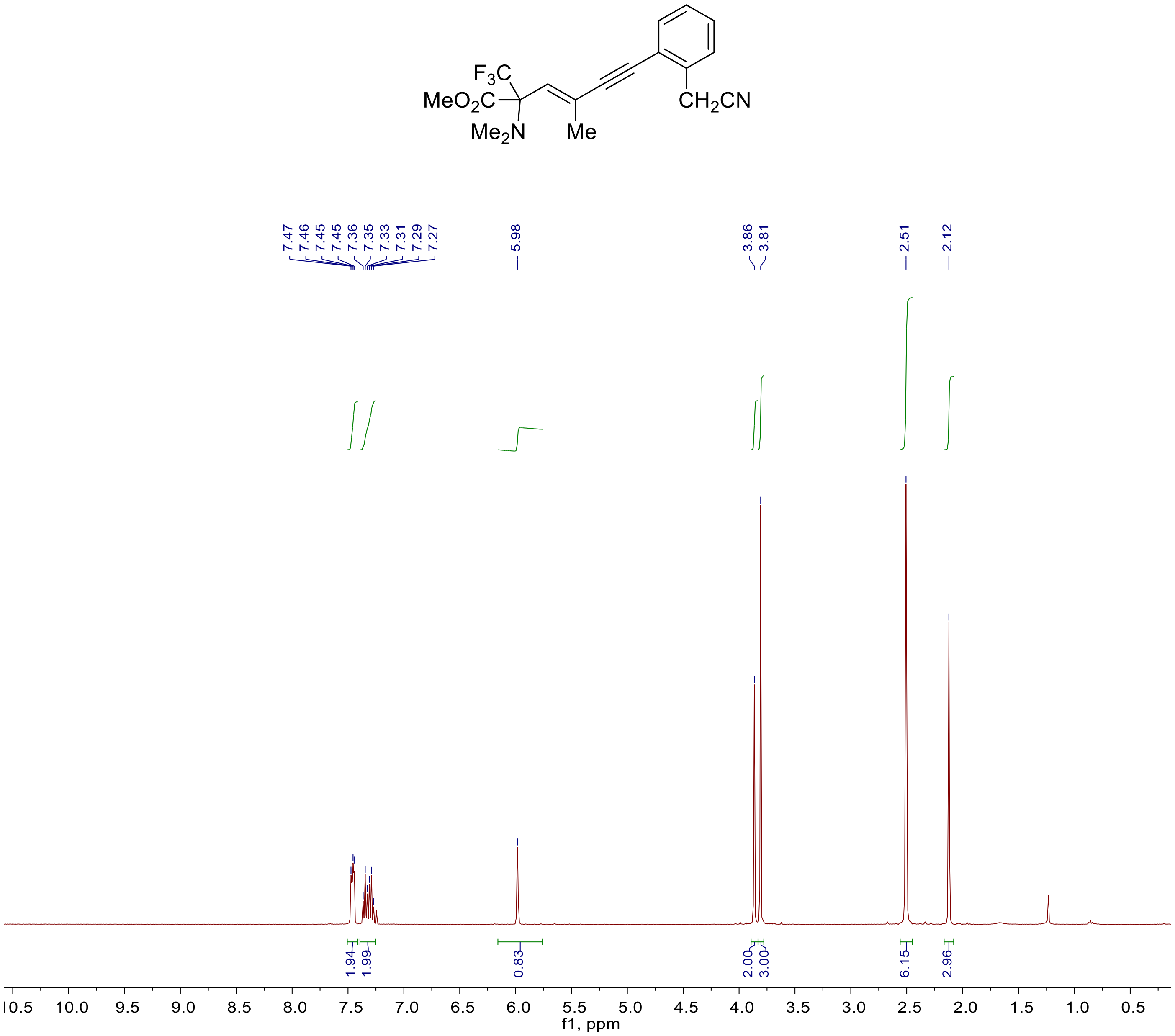

^{1}H spectrum of **3k** in $CDCl_3$

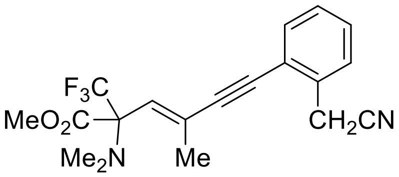

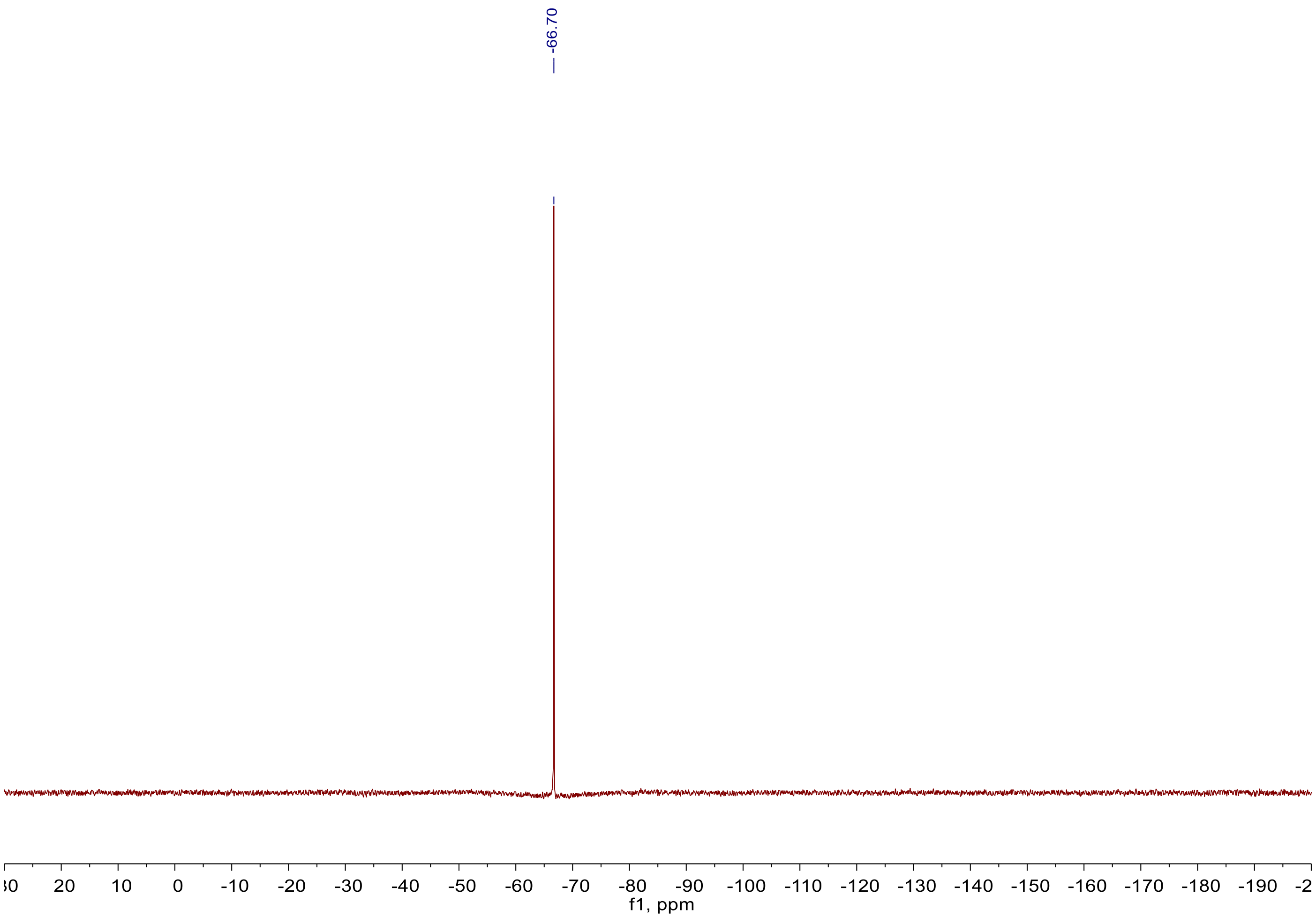

^{19}F spectrum of **3k** in $CDCl_3$

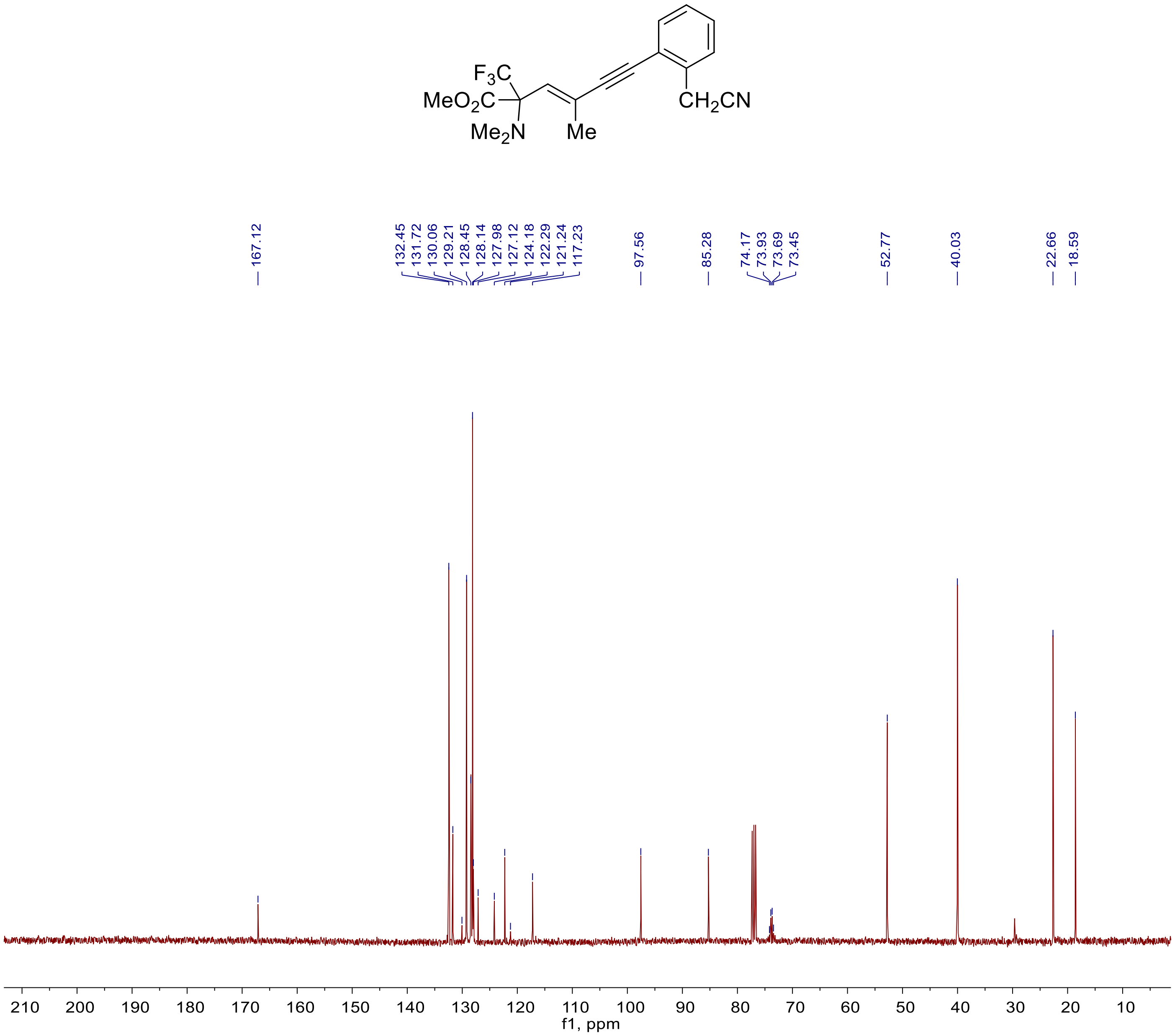

^{13}C spectrum of **3k** in $CDCl_3$

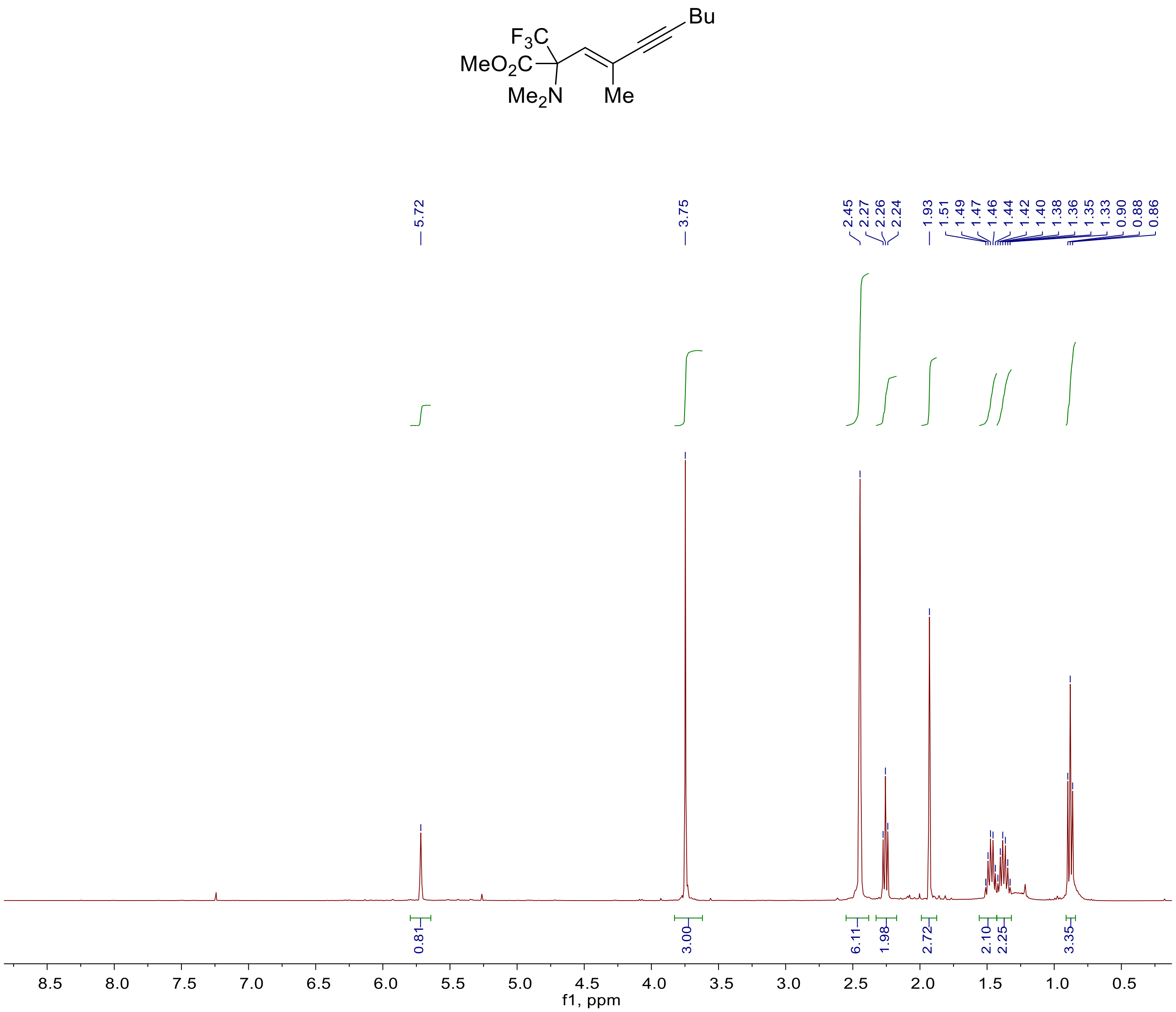

^{1}H spectrum of **3l** in $CDCl_3$

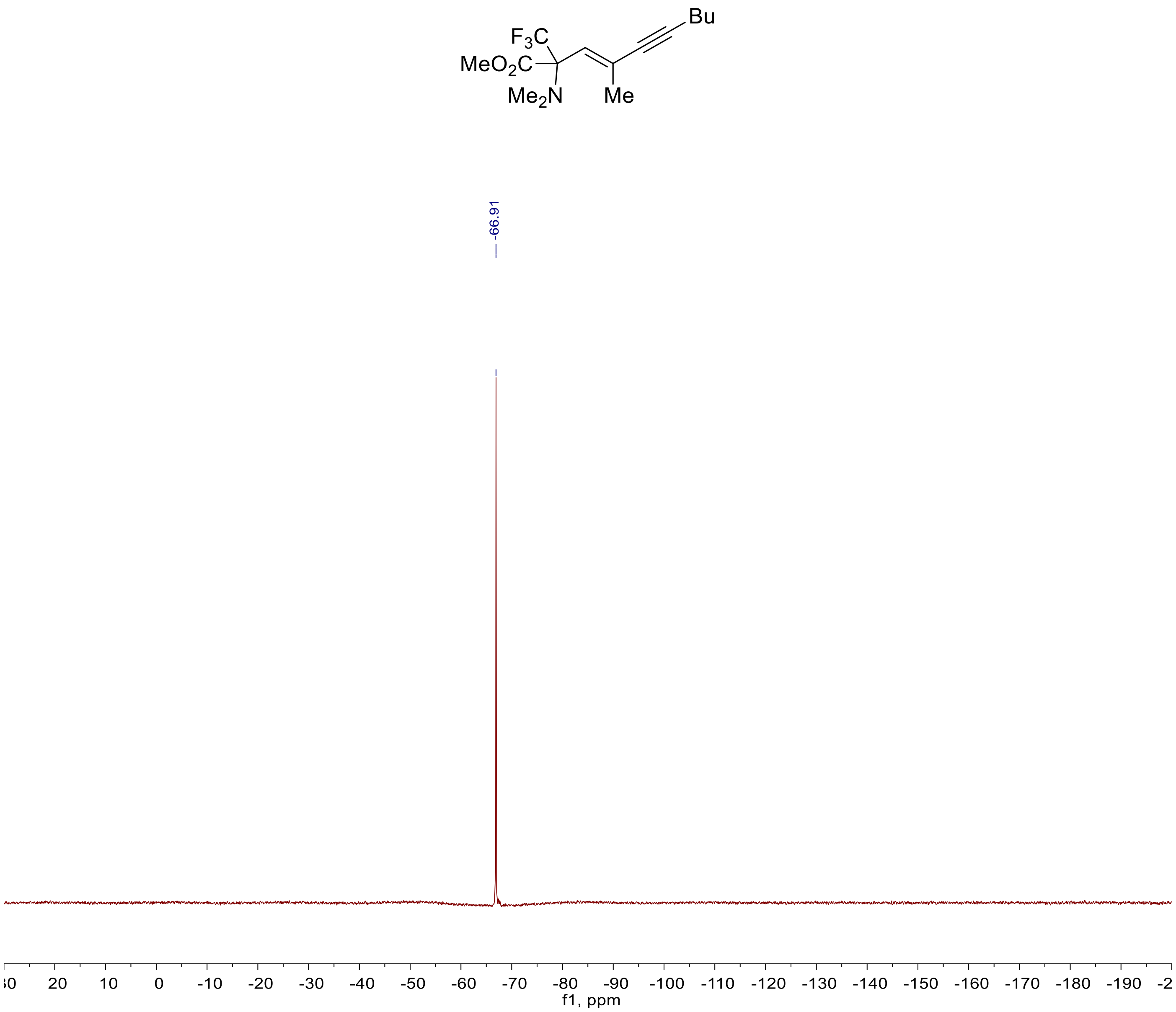

^{19}F spectrum of **3l** in $CDCl_3$

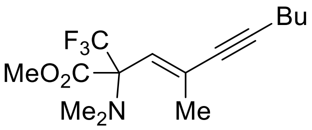

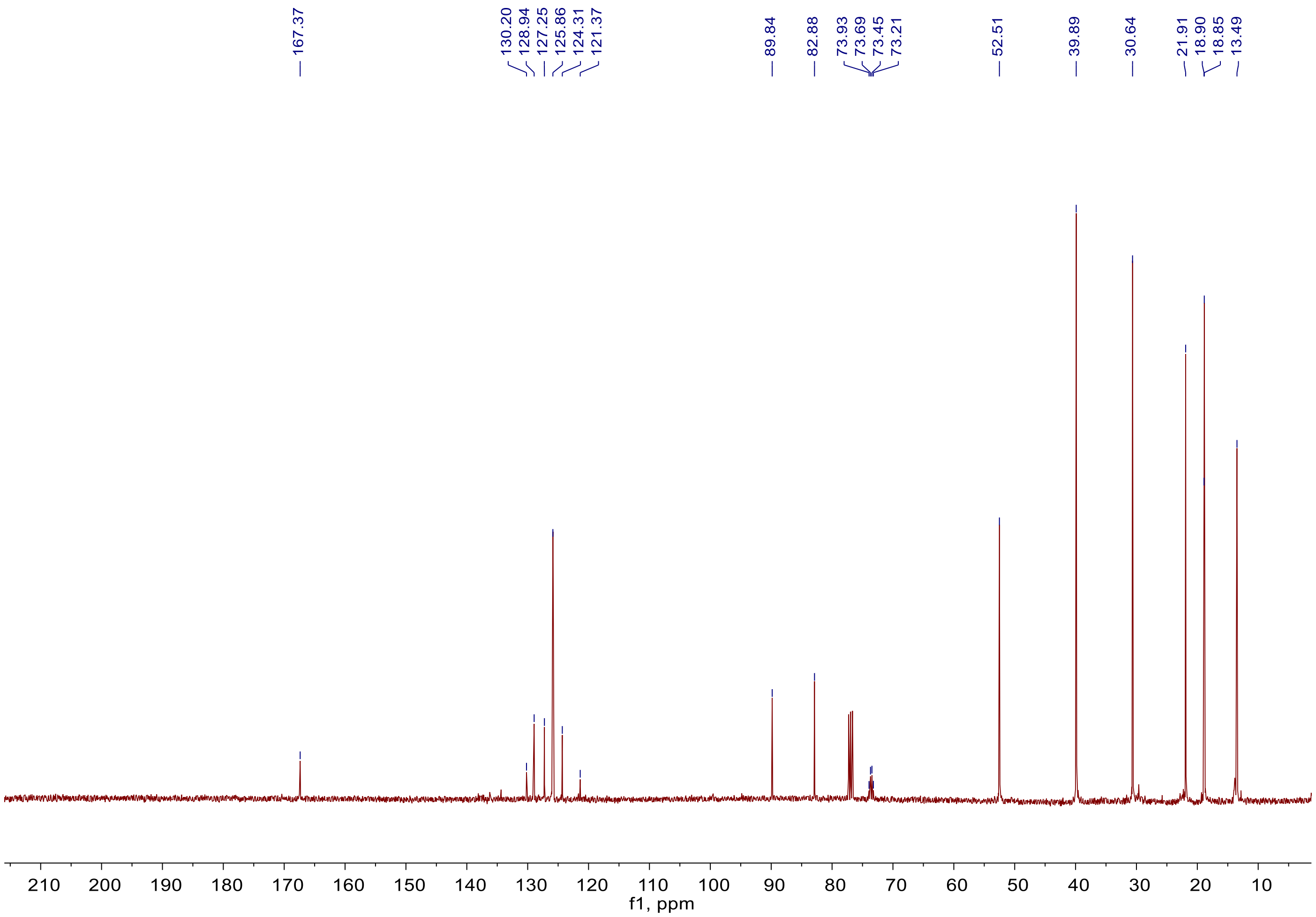

^{13}C spectrum of **3l** in $CDCl_3$

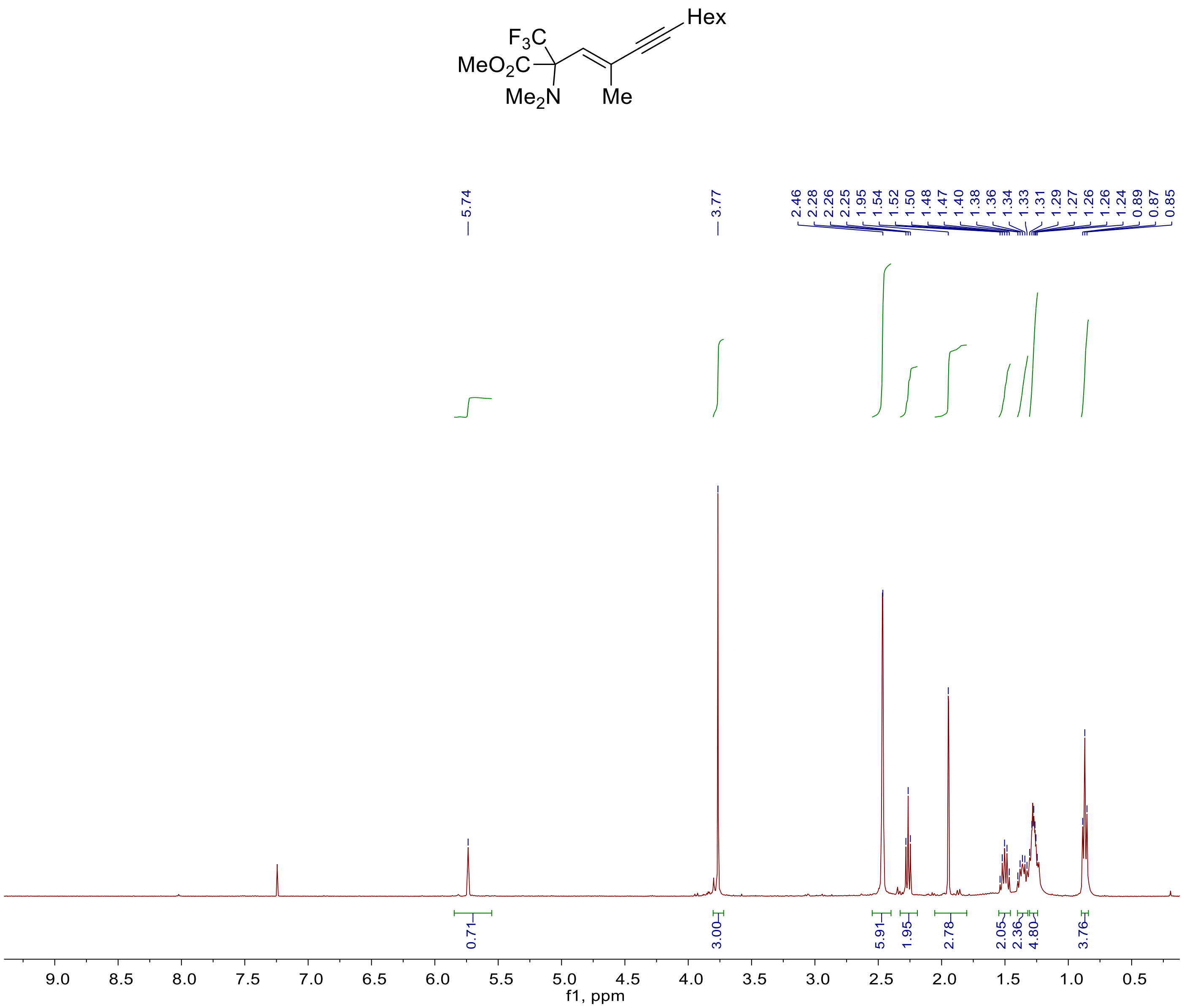

^{1}H spectrum of **3m** in $CDCl_3$

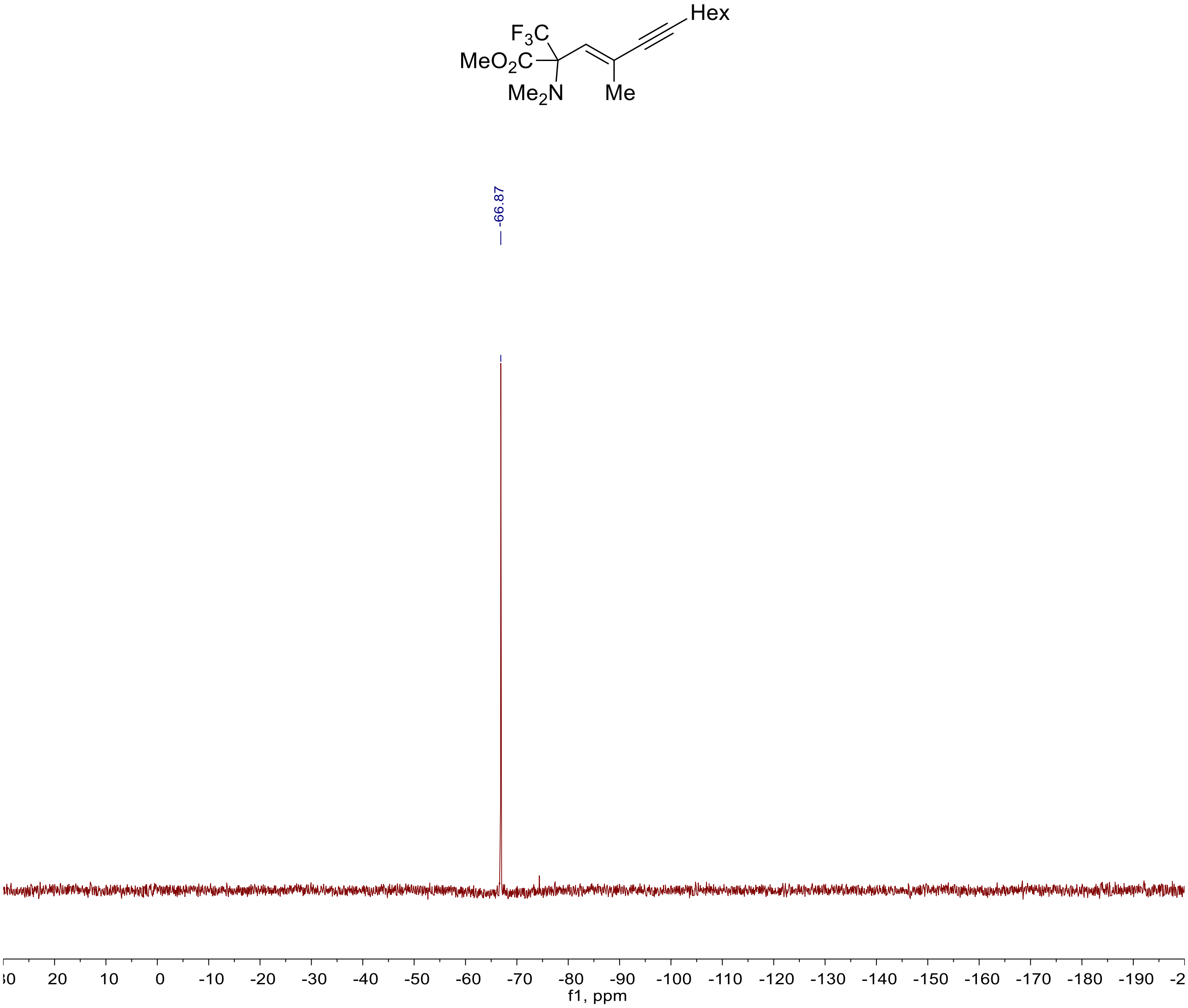

^{19}F spectrum of **3m** in $CDCl_3$

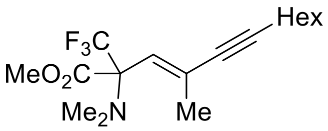

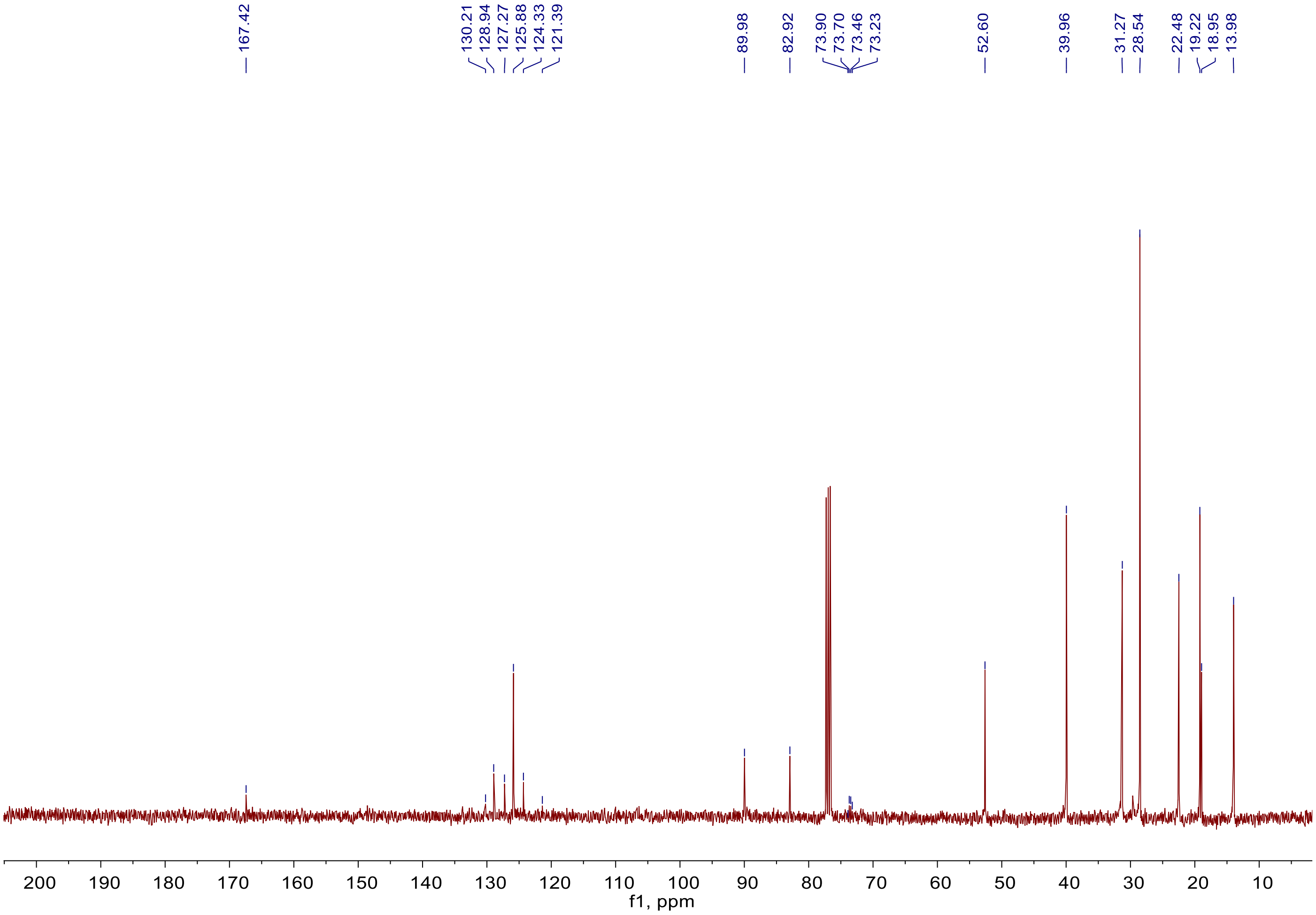

^{13}C spectrum of **3m** in $CDCl_3$

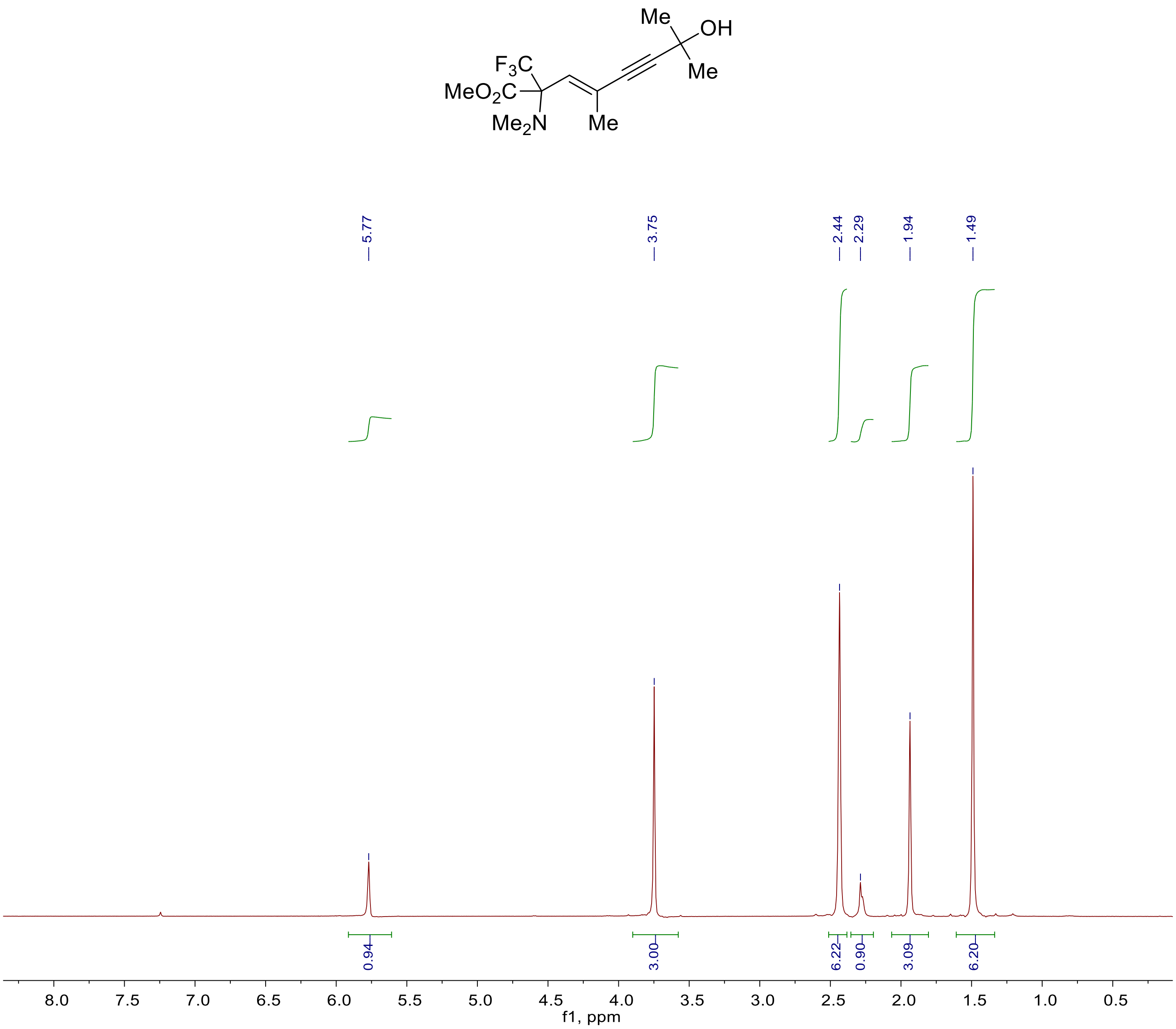

^{1}H spectrum of **3n** in $CDCl_3$

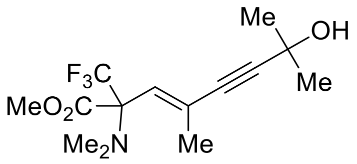

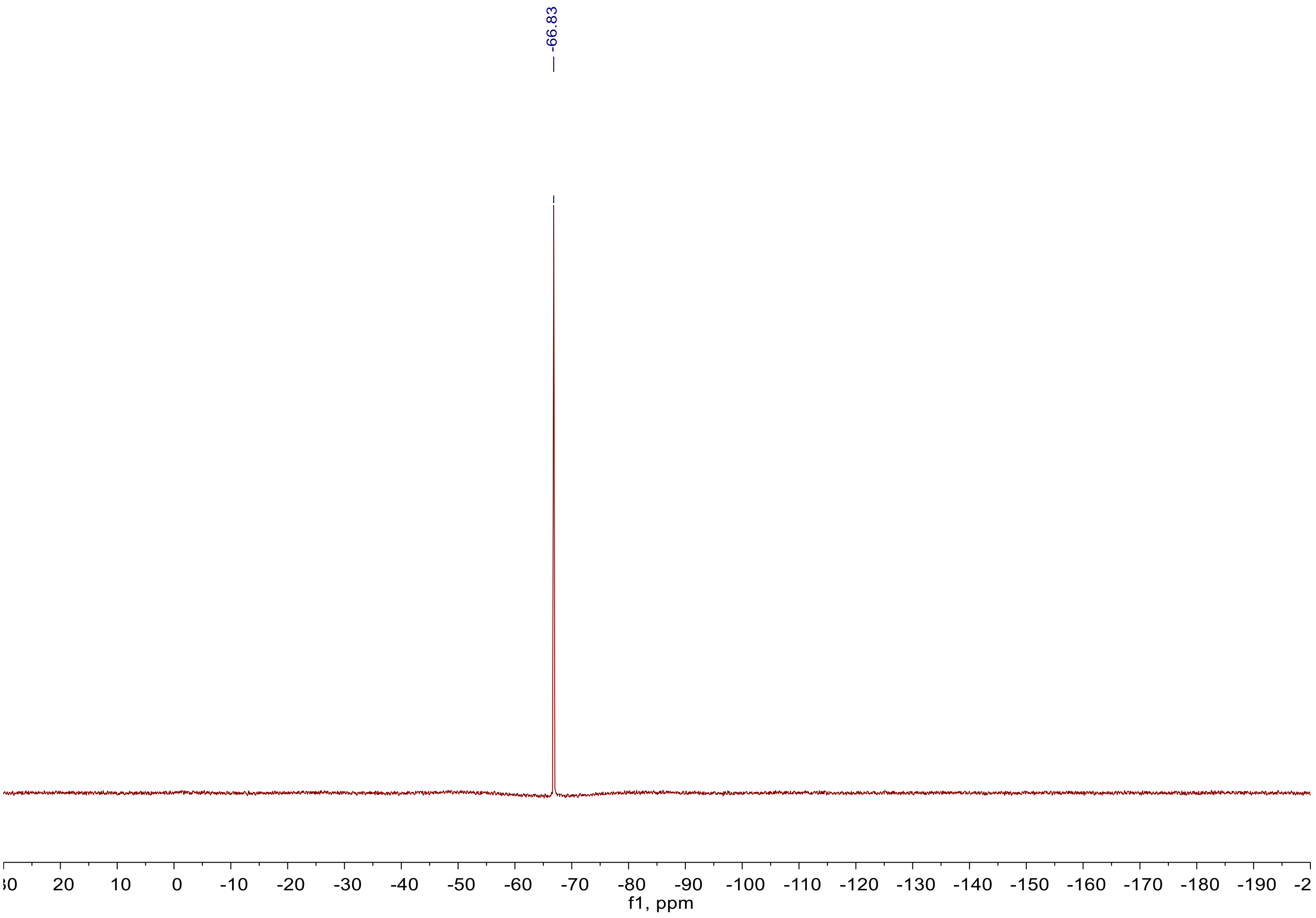

^{19}F spectrum of **3n** in $CDCl_3$

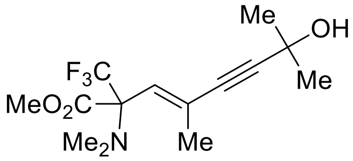

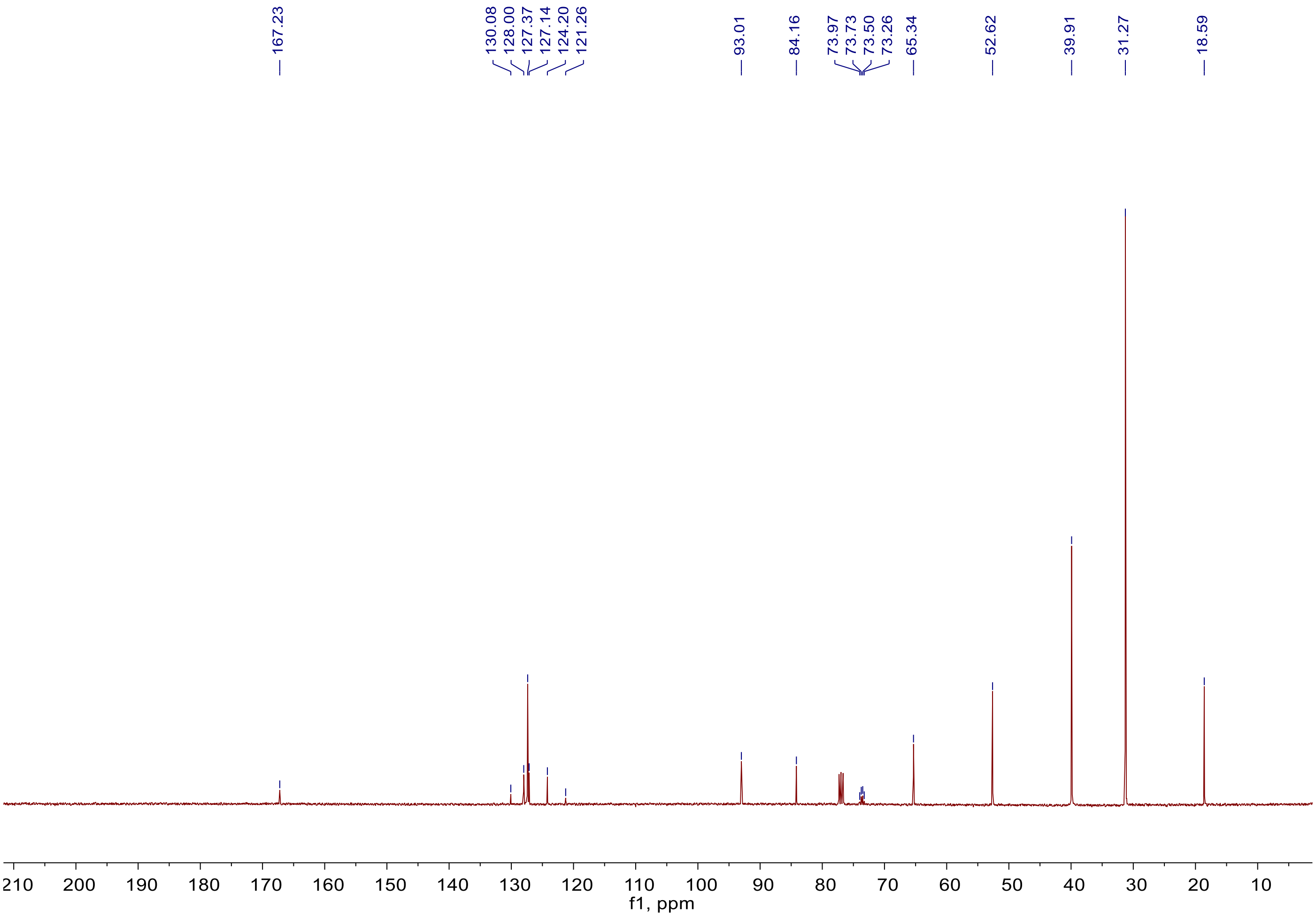

^{13}C spectrum of **3n** in $CDCl_3$

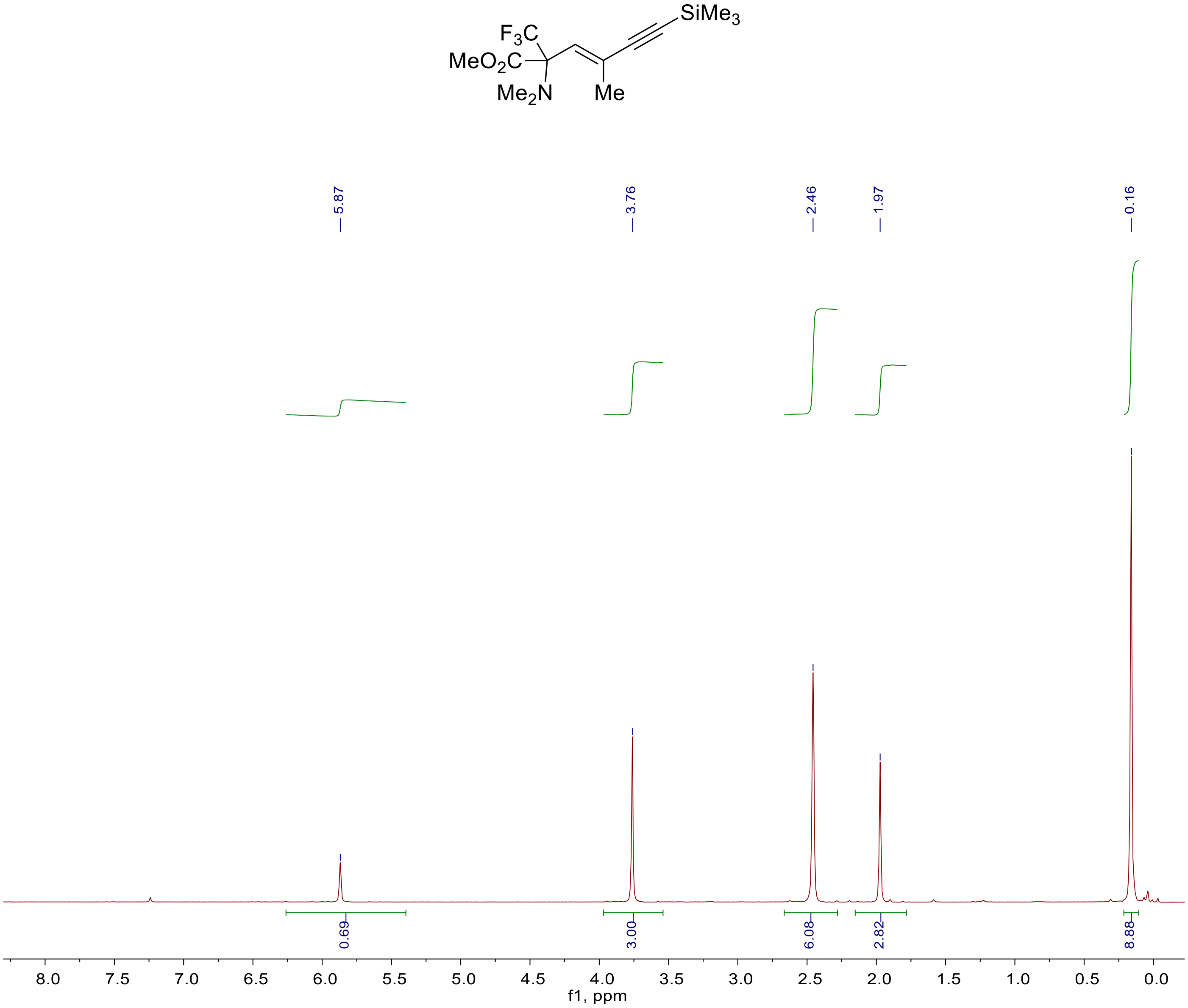

^{1}H spectrum of **3o** in $CDCl_3$

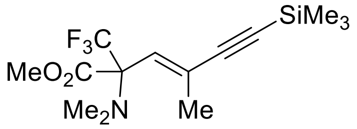

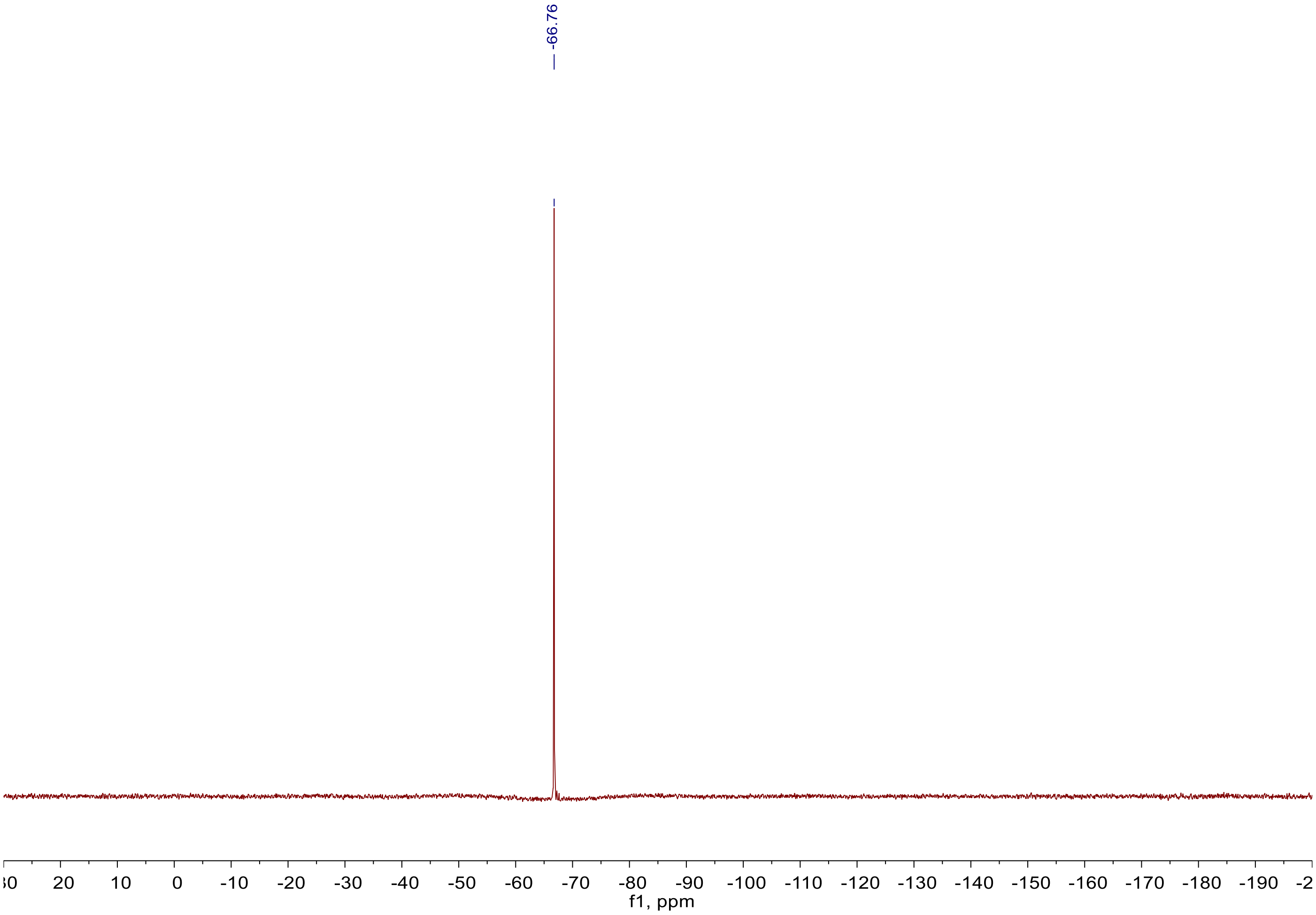

^{19}F spectrum of **3o** in $CDCl_3$

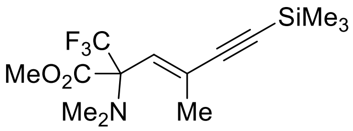

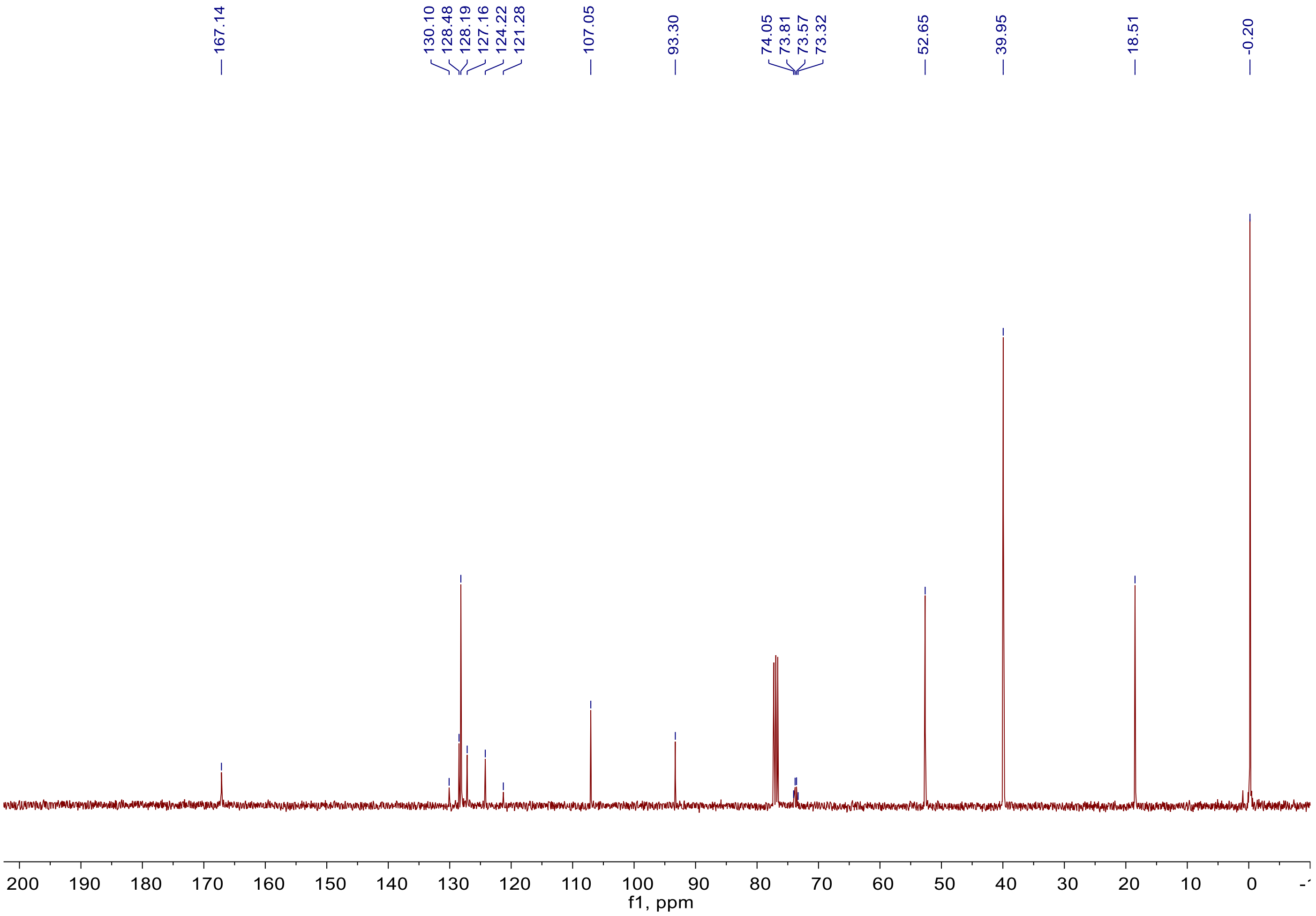

^{13}C spectrum of **3o** in $CDCl_3$

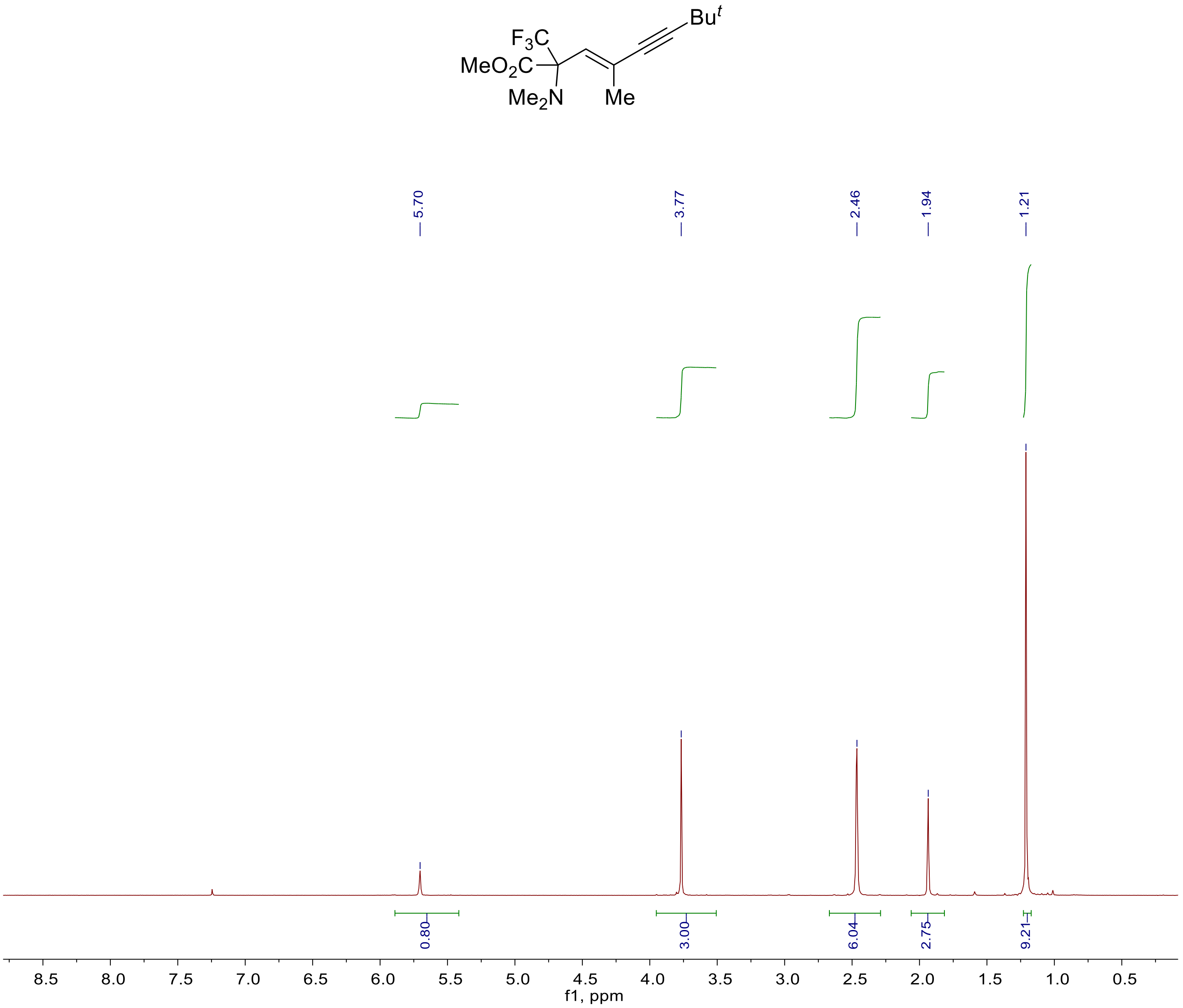

^1H spectrum of **3p** in $CDCl_3$

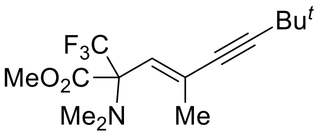

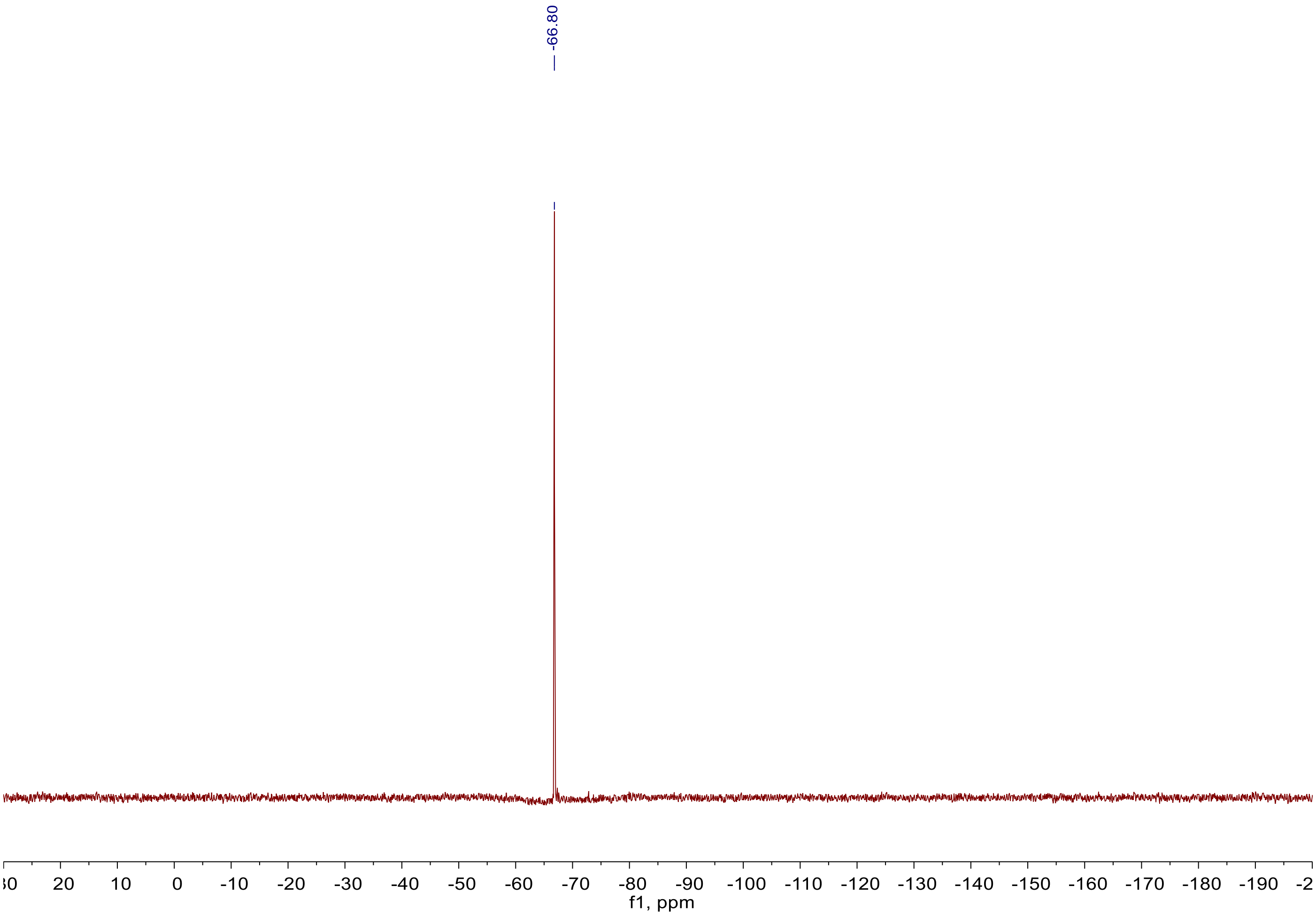

^{19}F spectrum of **3p** in $CDCl_3$

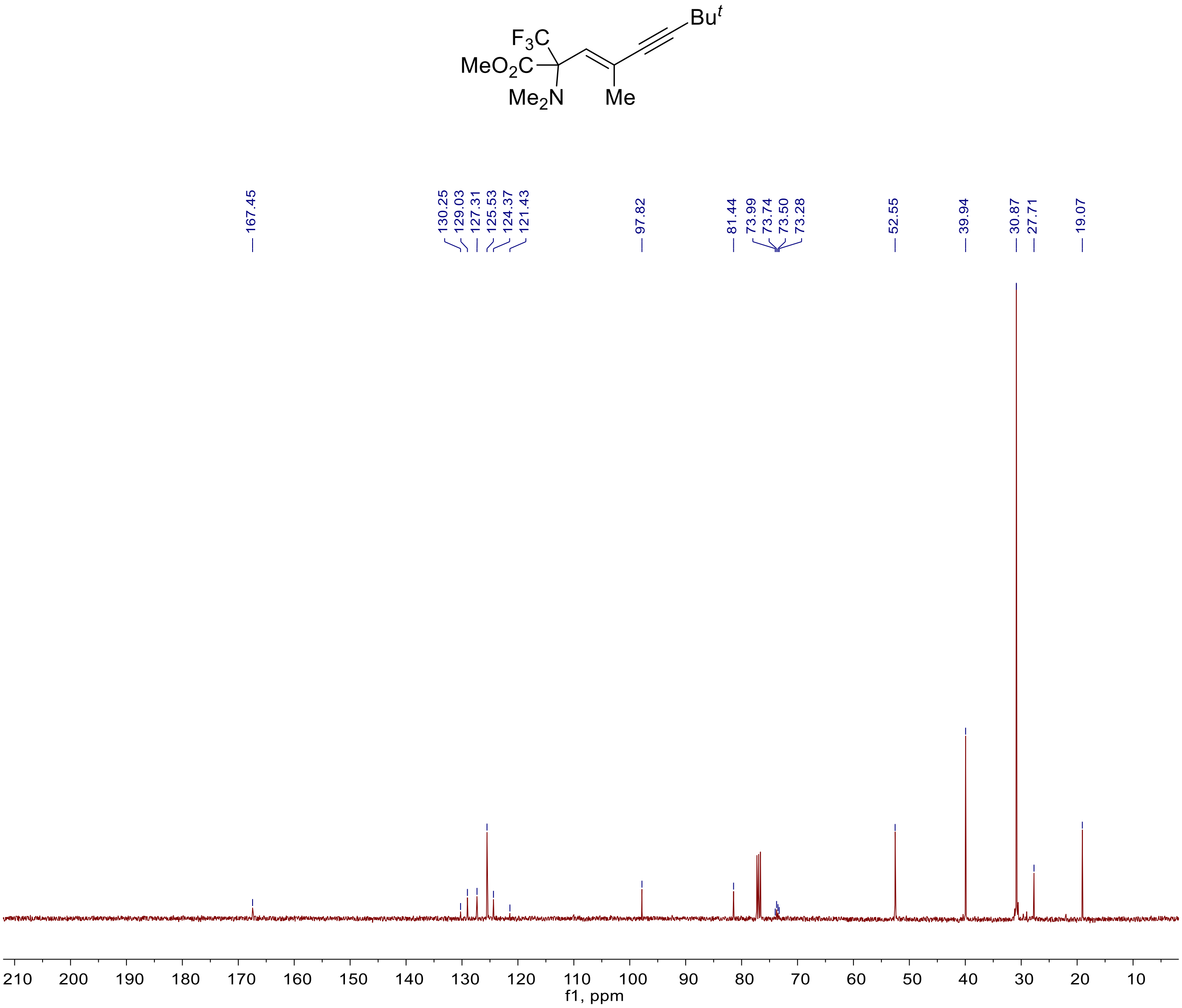

^{13}C spectrum of **3p** in $CDCl_3$

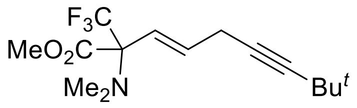

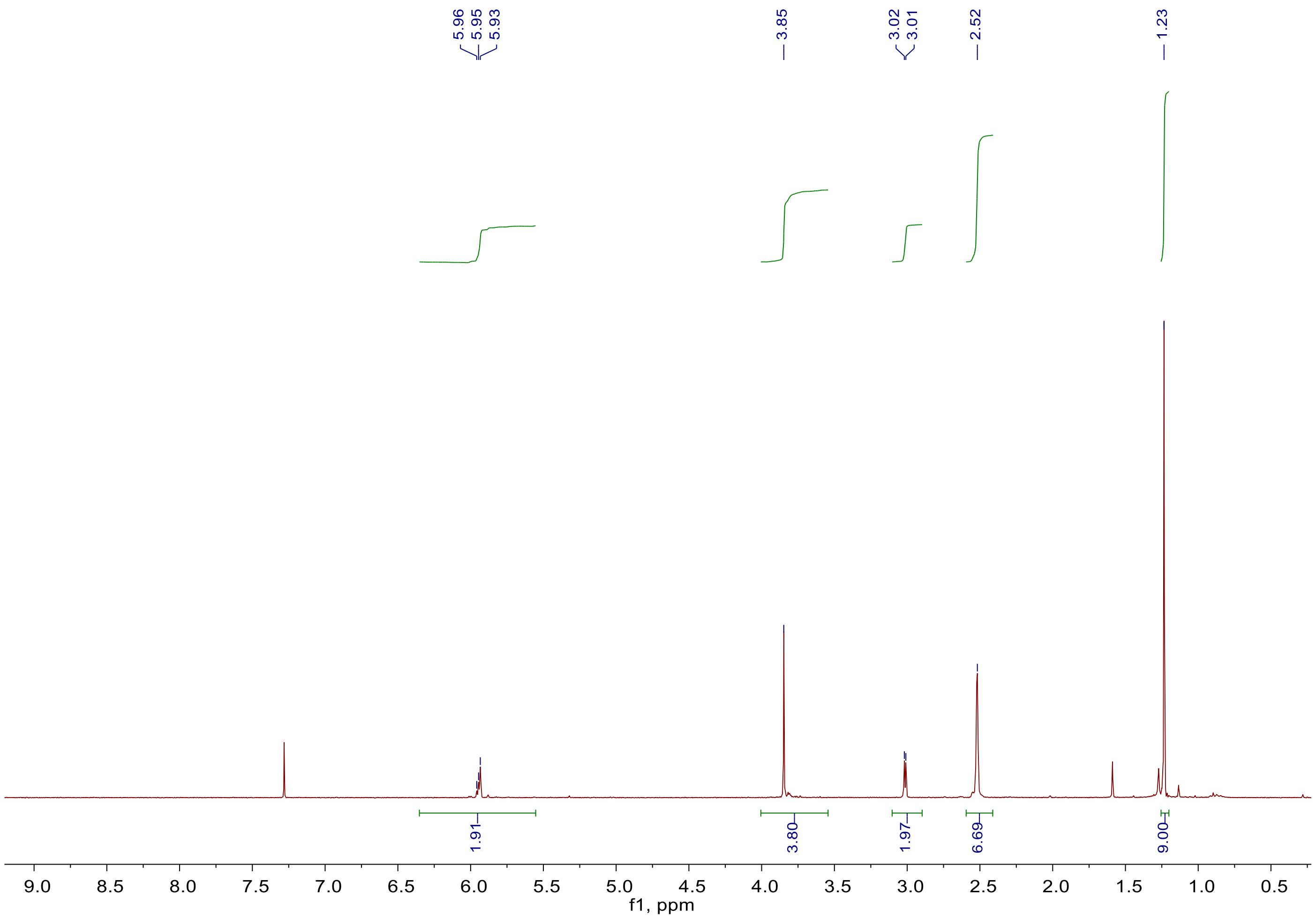

^{1}H spectrum of **3p’** in $CDCl_3$

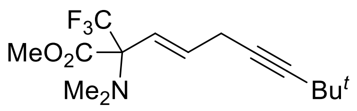

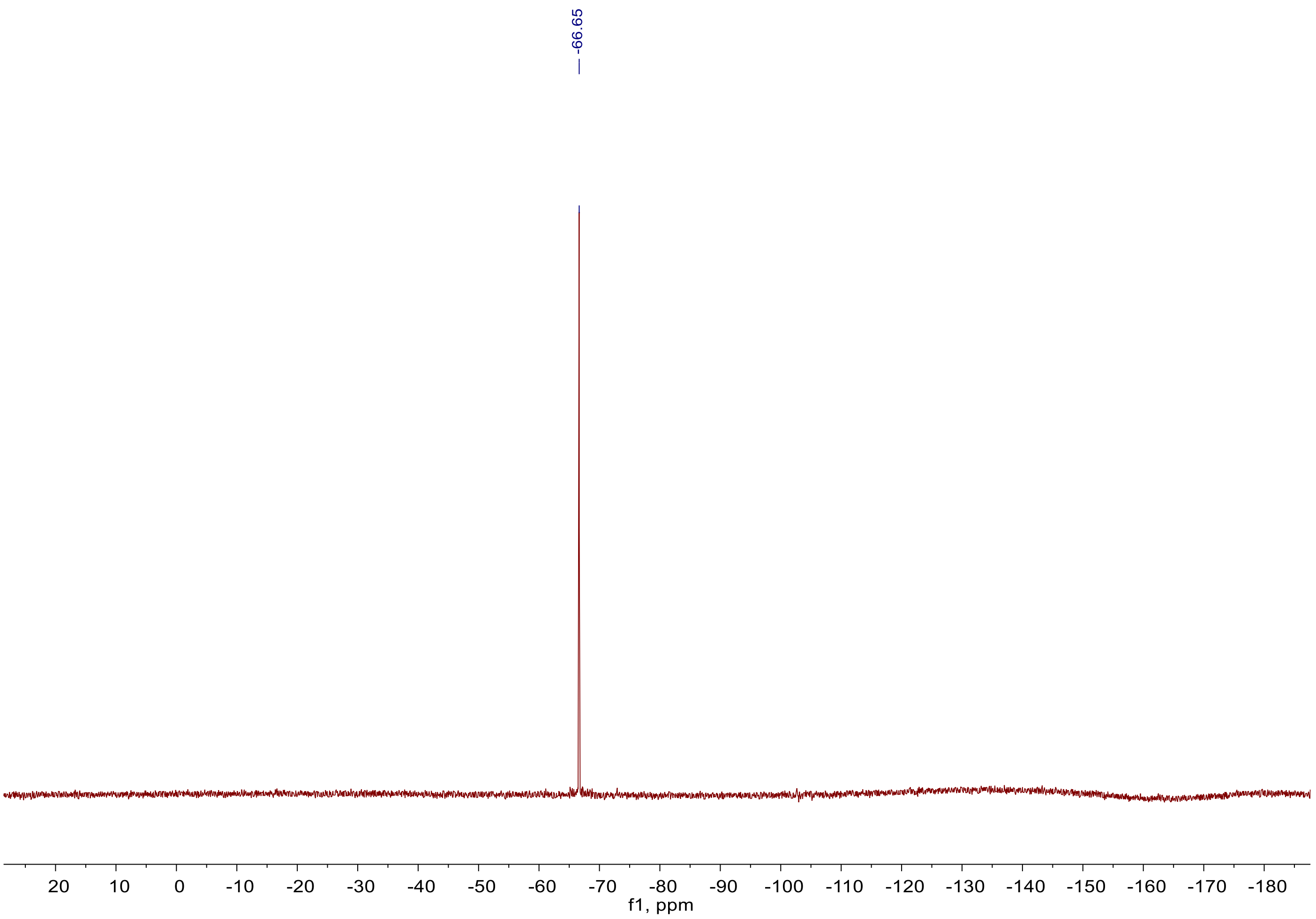

^{19}F spectrum of **3p'** in $CDCl_3$

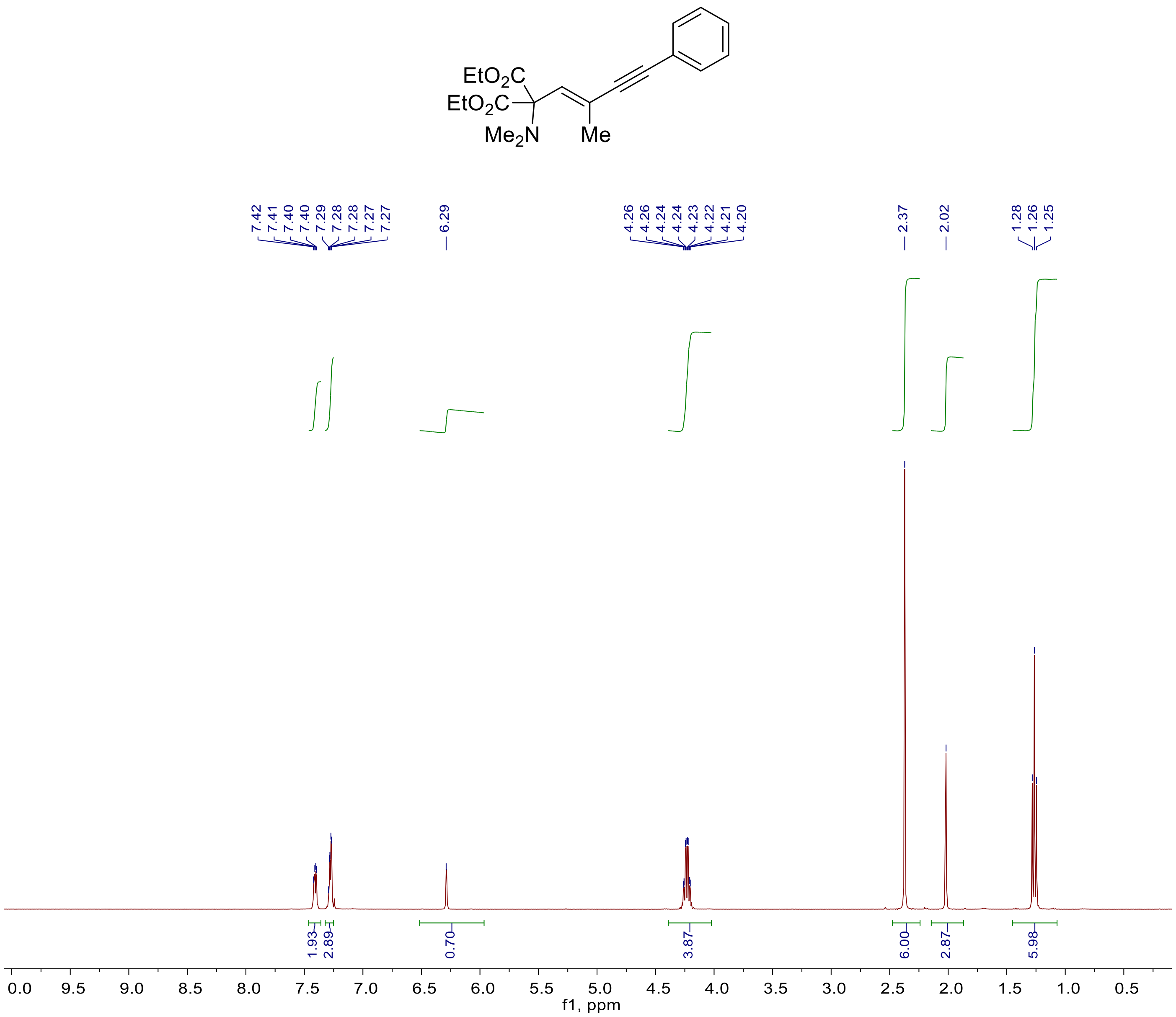

^{1}H spectrum of **4a** in $CDCl_3$

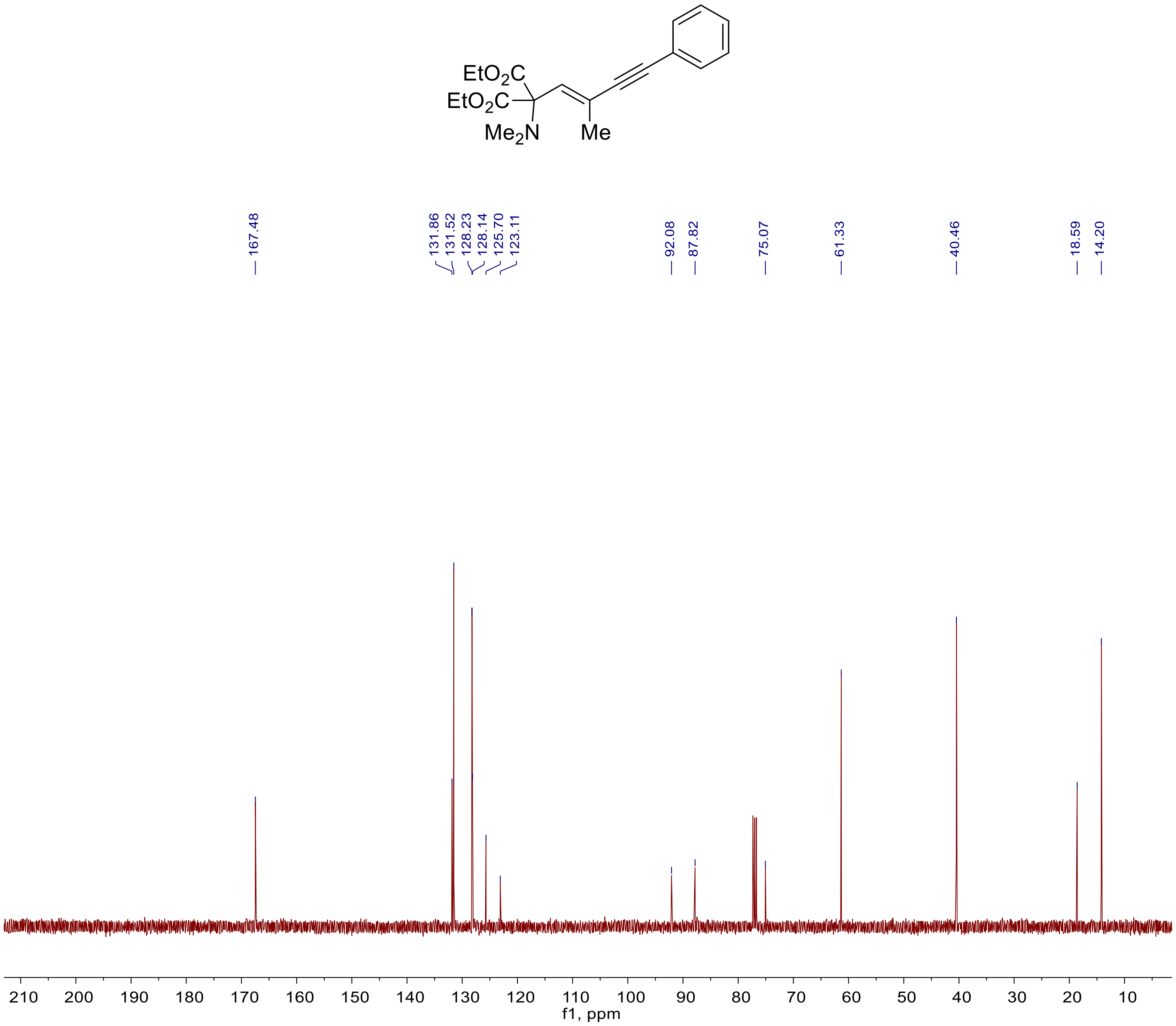

^{13}C spectrum of **4a** in $CDCl_3$

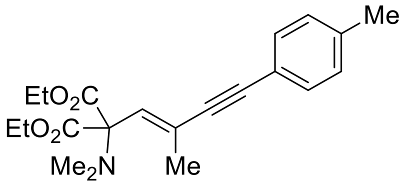

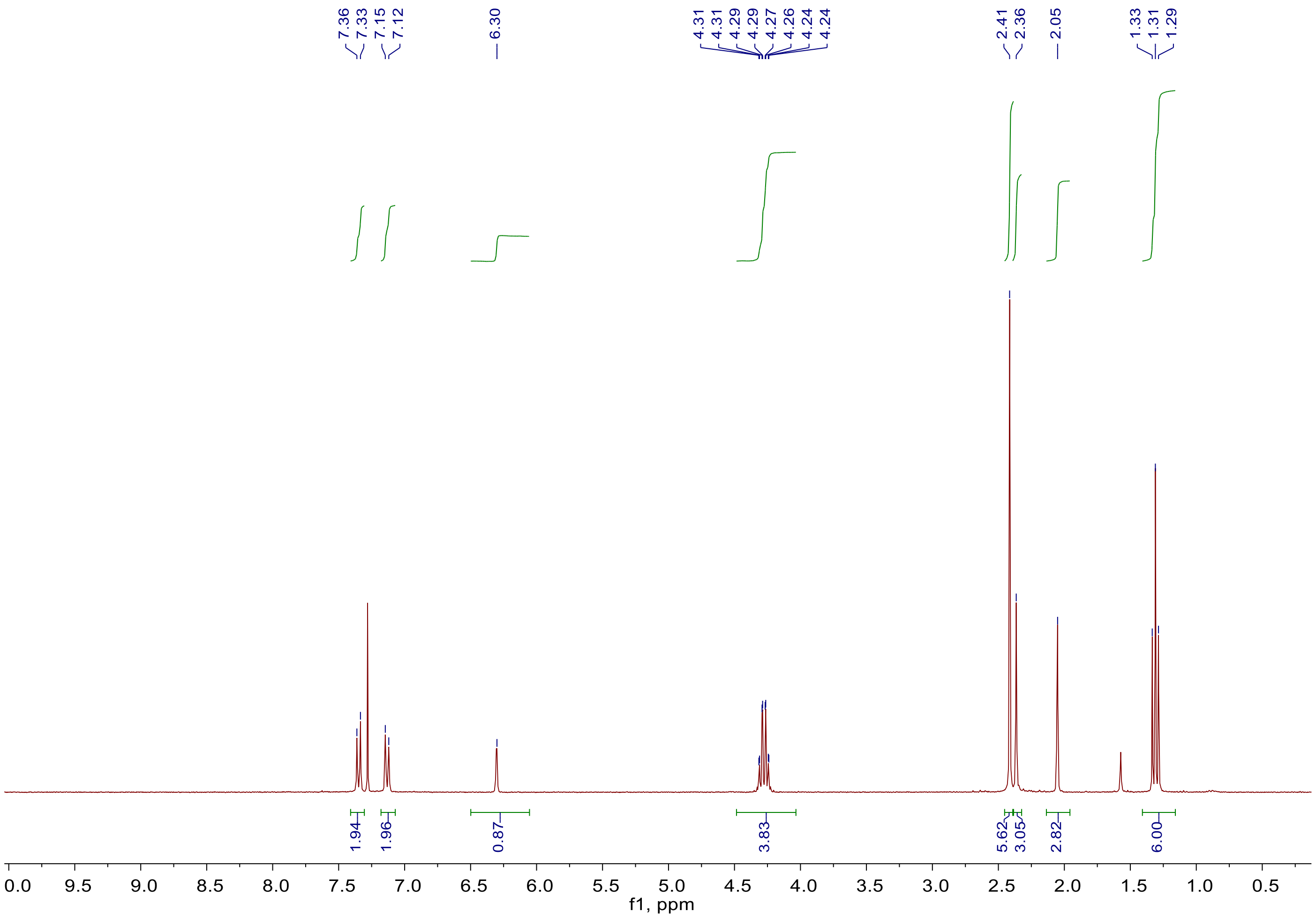

^{1}H spectrum of **4b** in $CDCl_3$

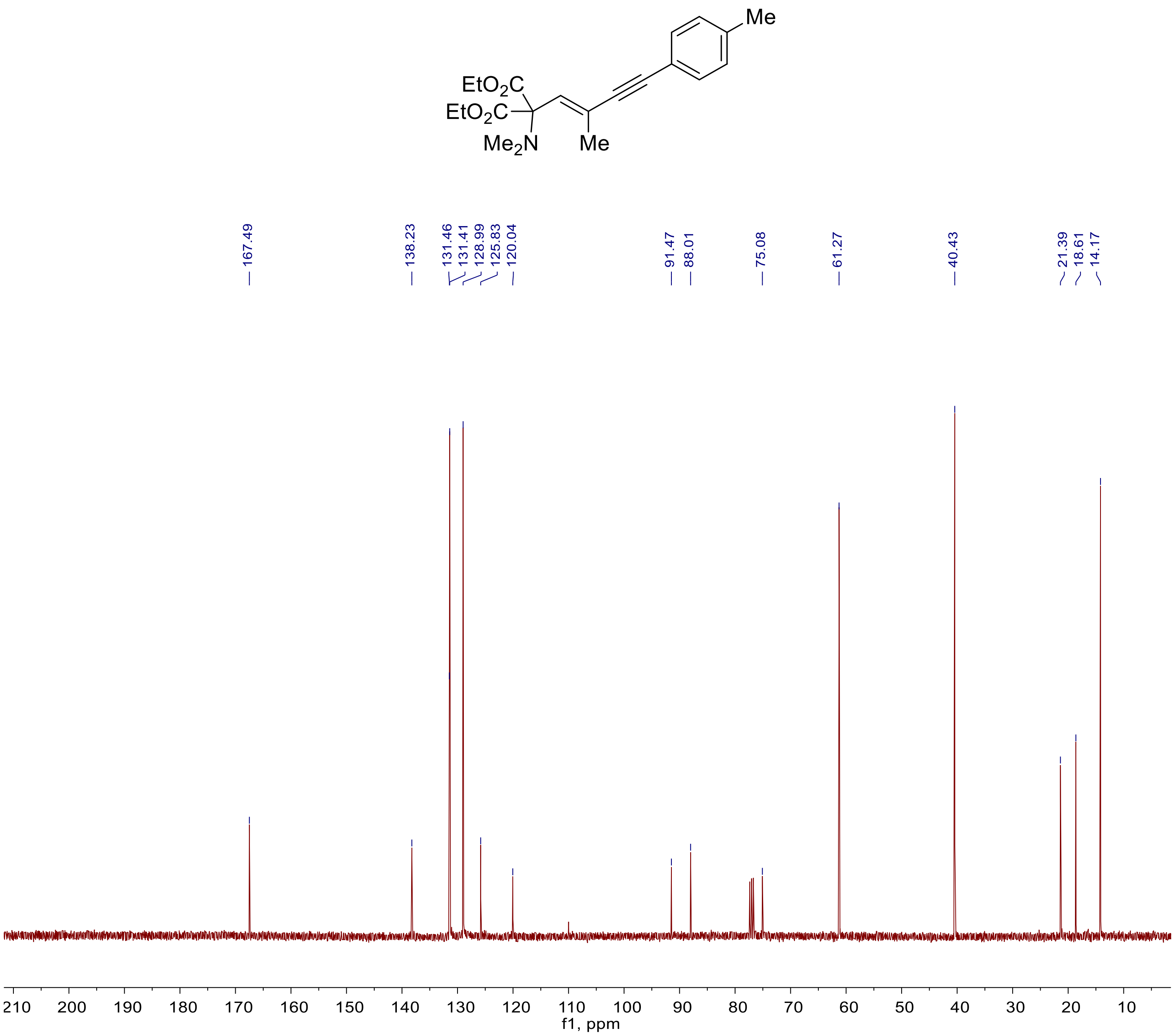

^{13}C spectrum of **4b** in $CDCl_3$

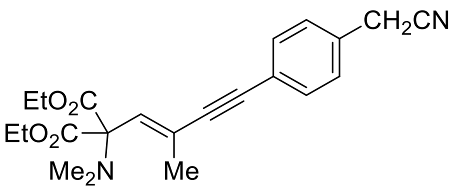

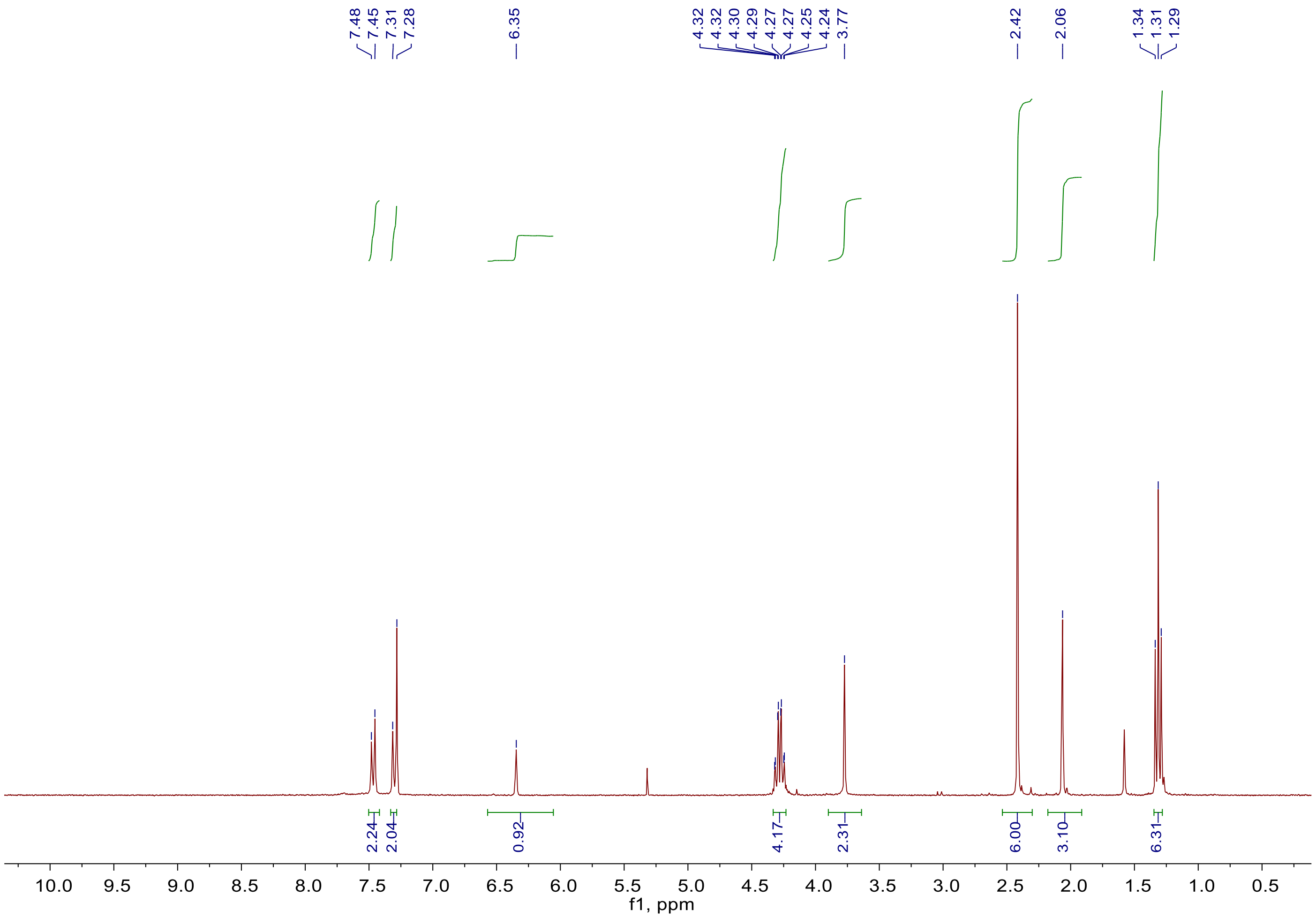

^{1}H spectrum of **4c** in $CDCl_3$

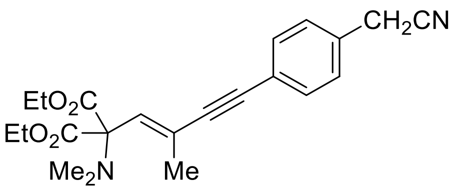

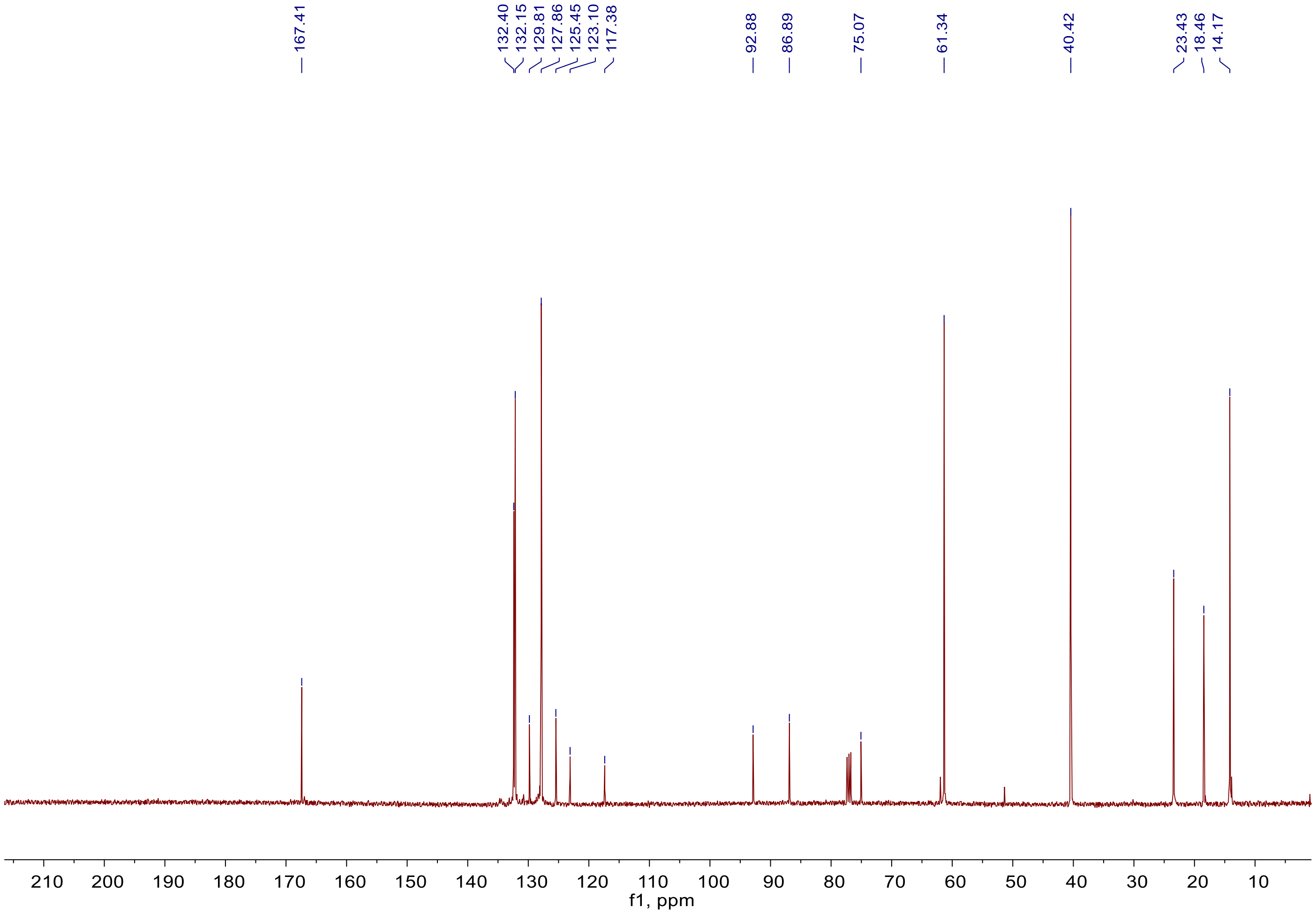

^{13}C spectrum of **4c** in $CDCl_3$

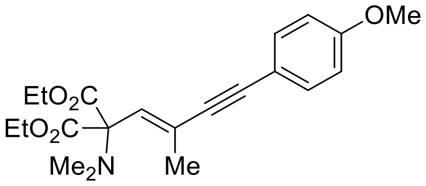

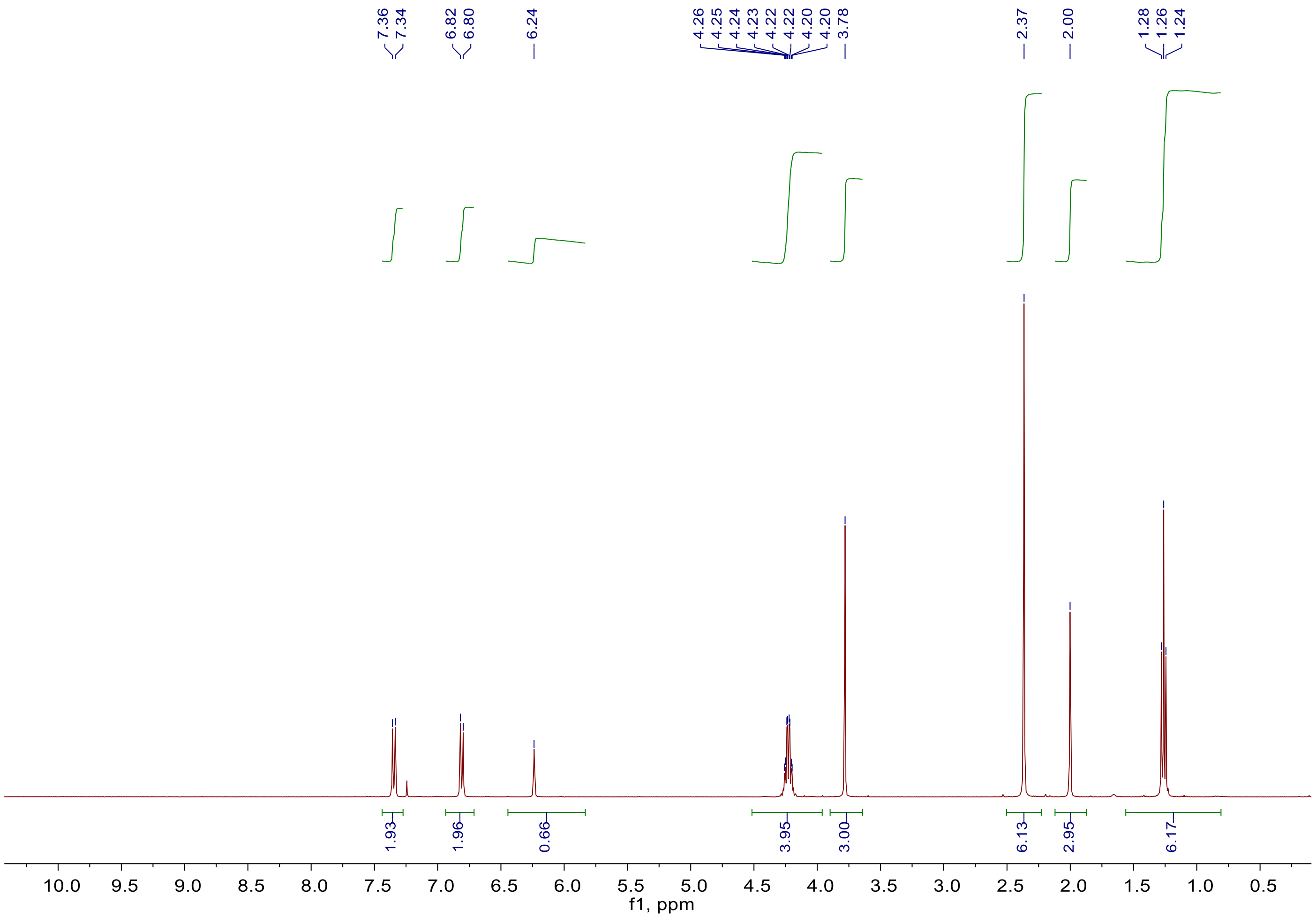

^{1}H spectrum of **4d** in $CDCl_3$

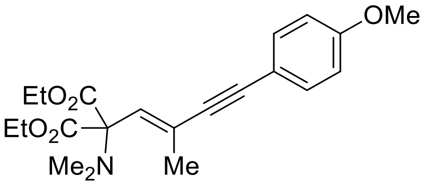

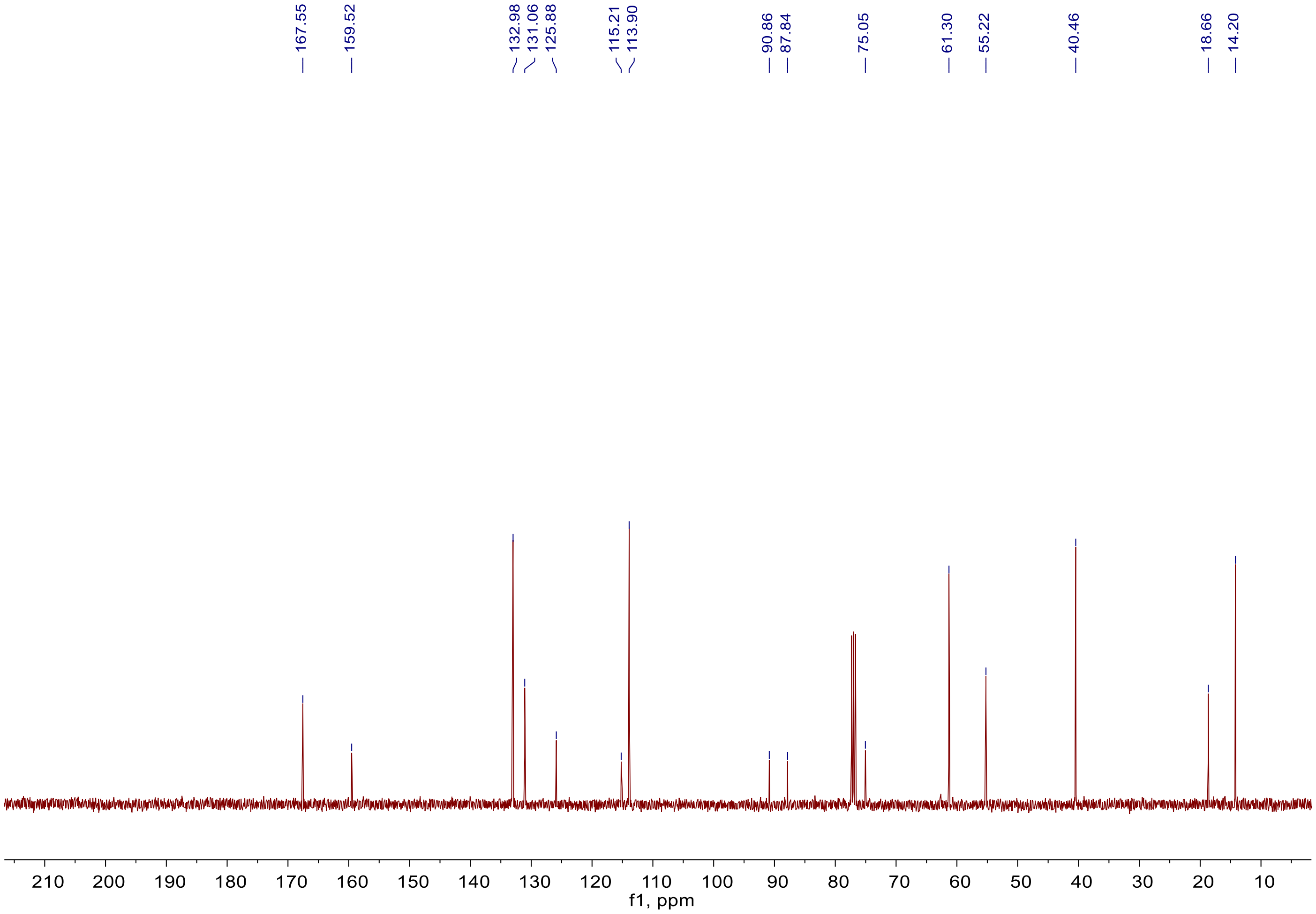

^{13}C spectrum of **4d** in $CDCl_3$

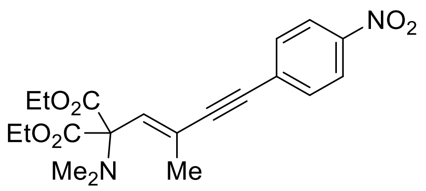

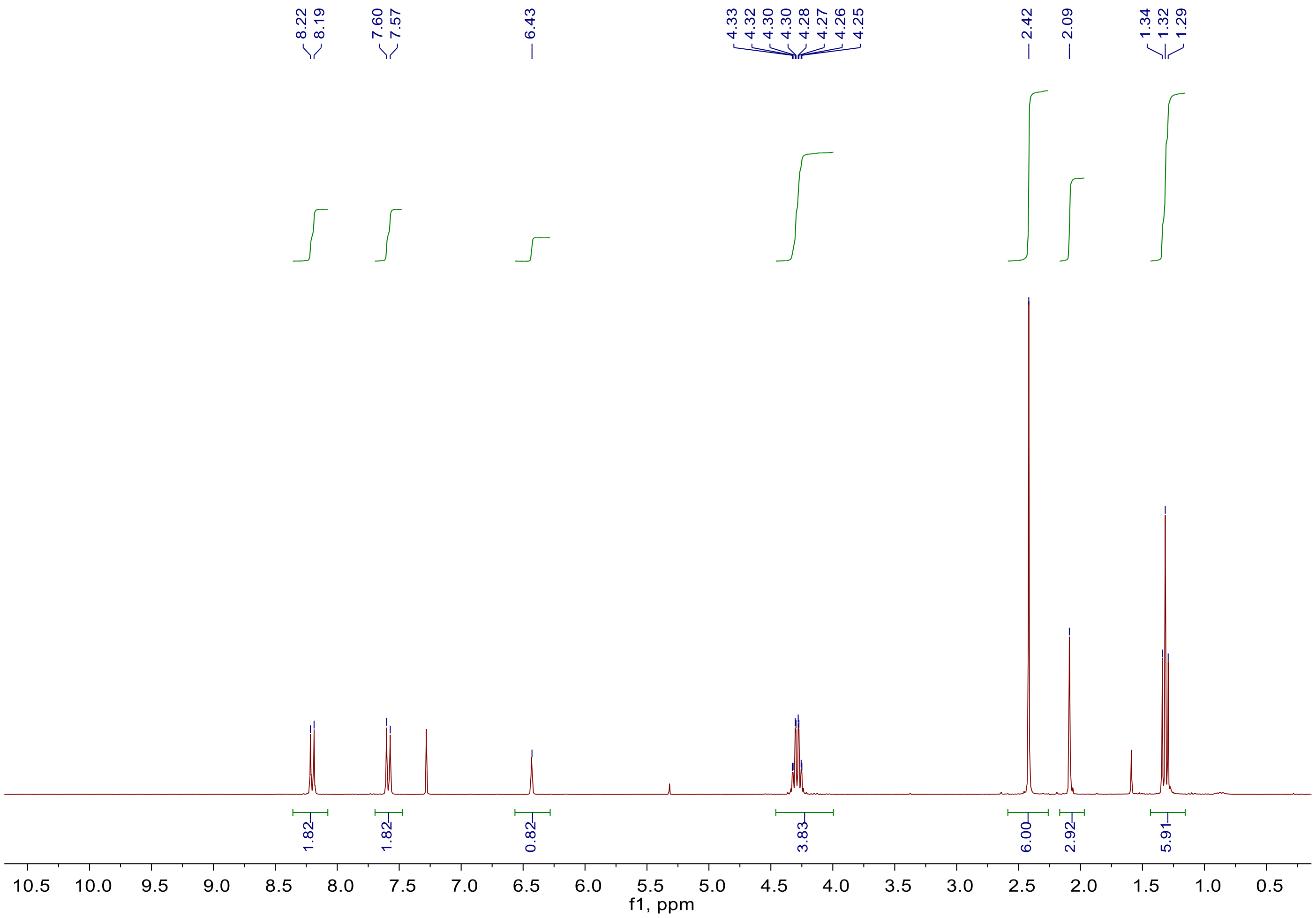

^{1}H spectrum of **4e** in $CDCl_3$

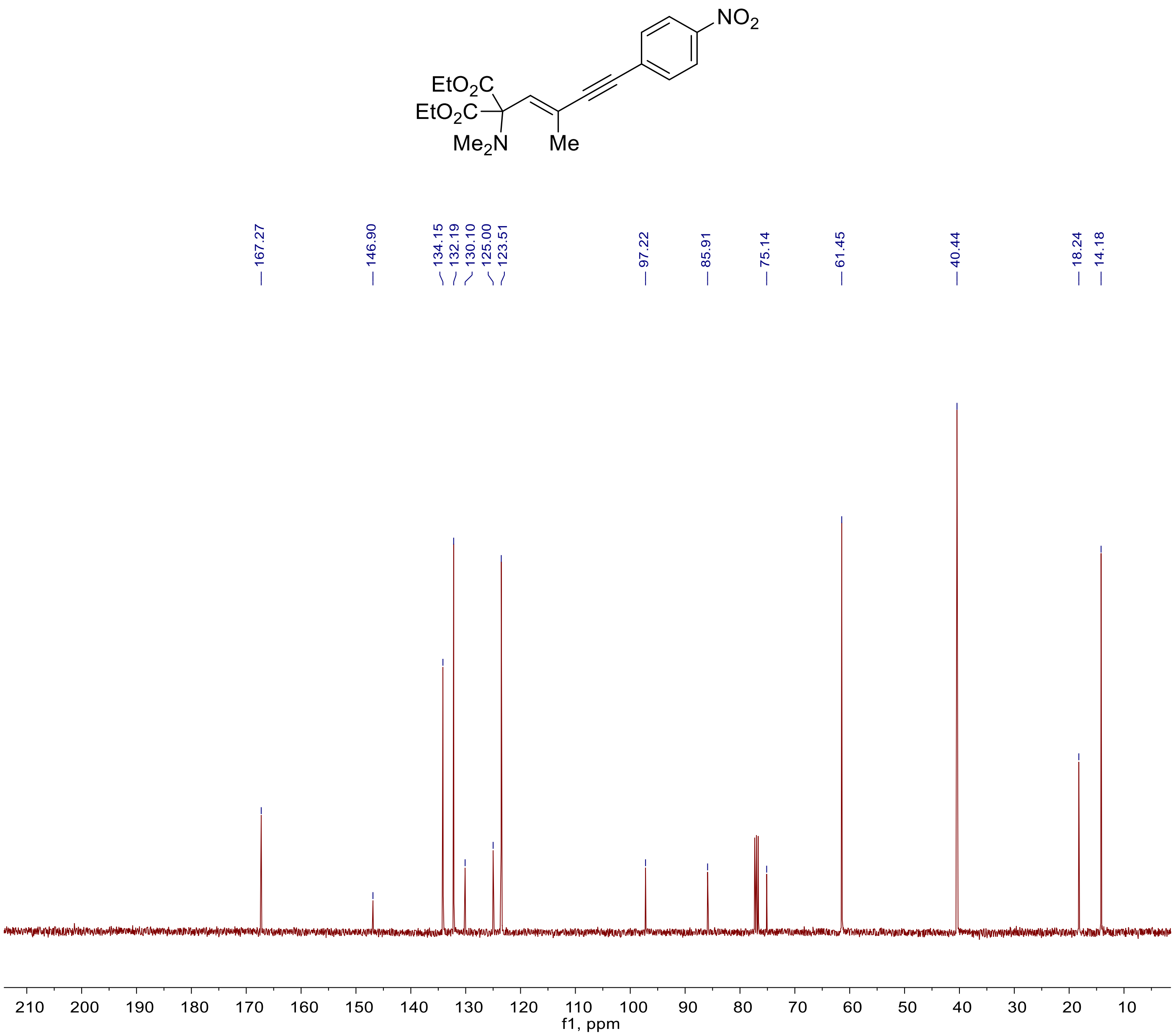

^{13}C spectrum of **4e** in $CDCl_3$

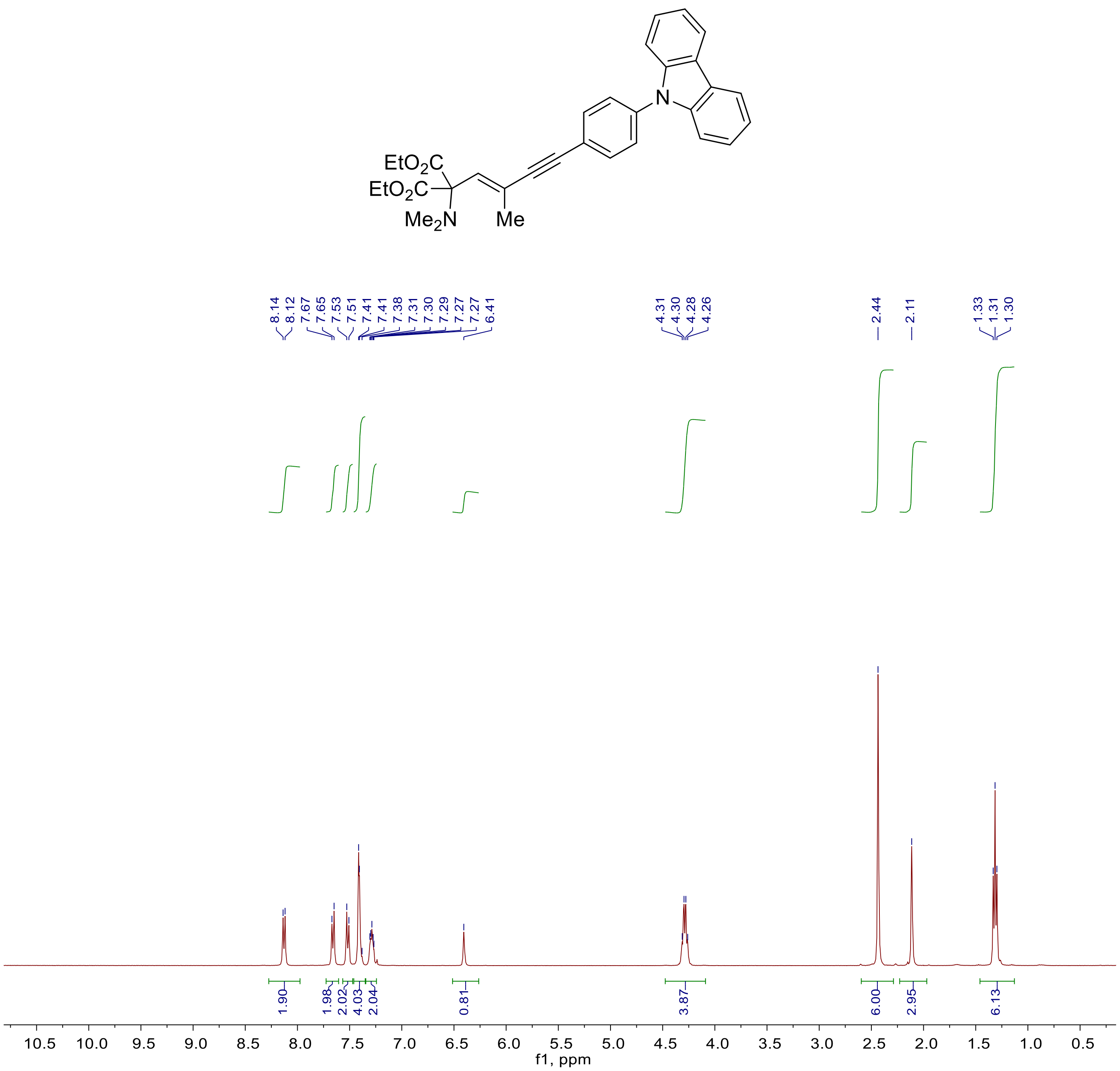

^{1}H spectrum of **4f** in $CDCl_3$

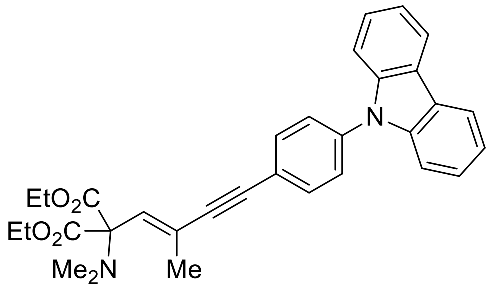

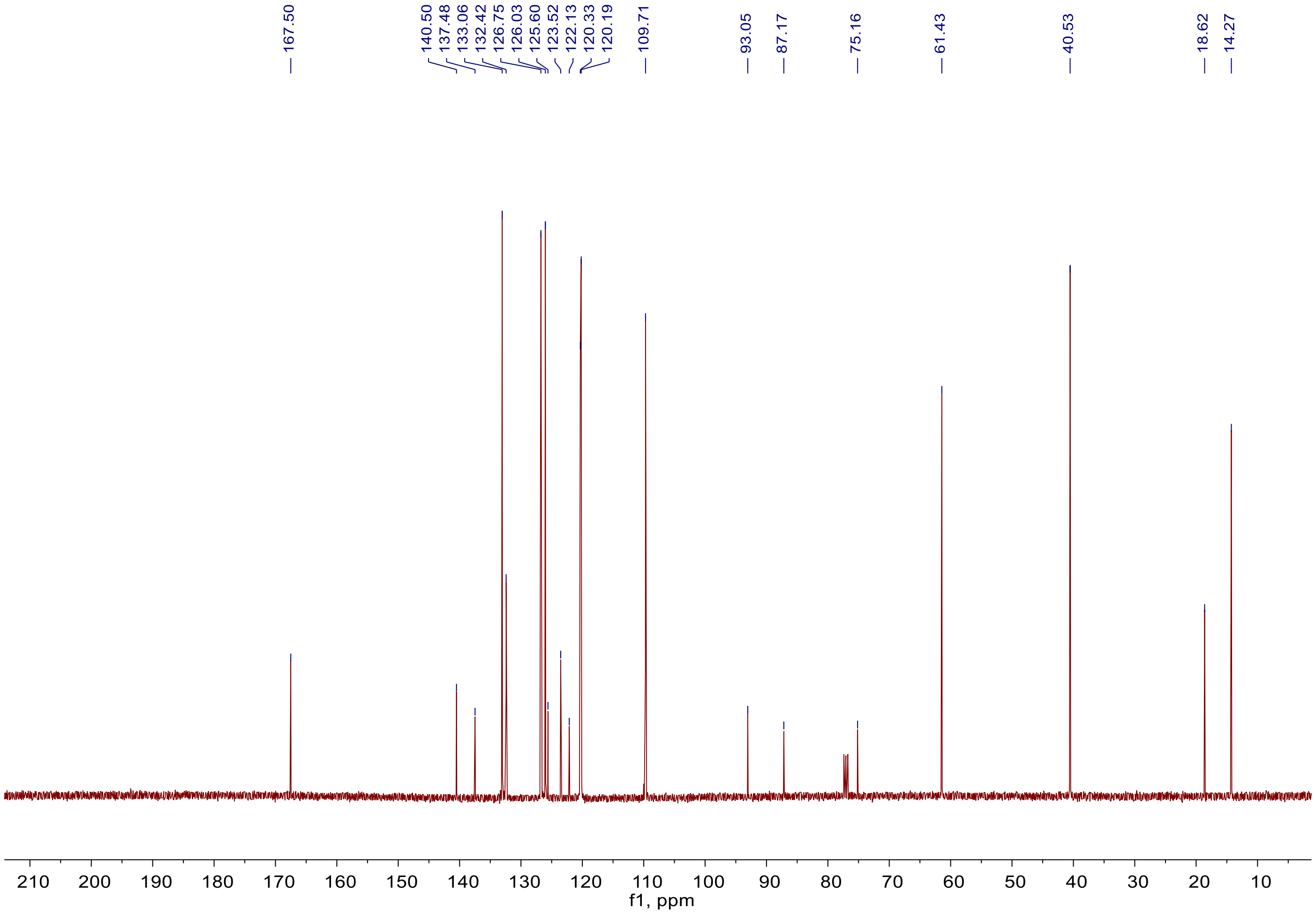

^{13}C spectrum of **4f** in $CDCl_3$

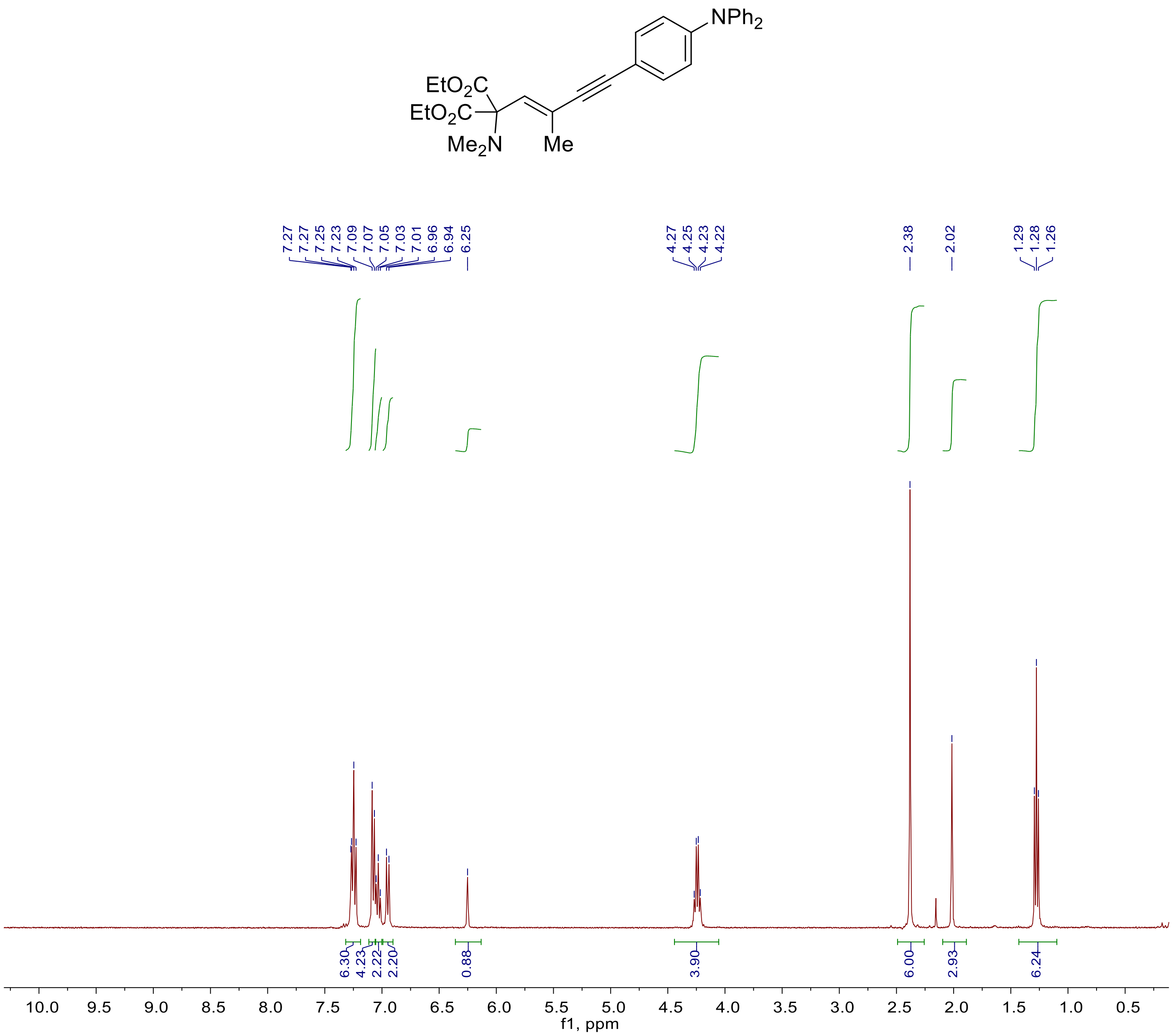

^{1}H spectrum of **4g** in $CDCl_3$

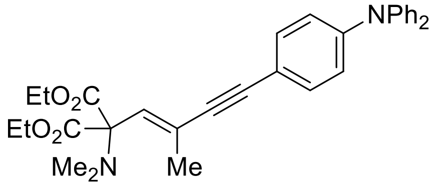

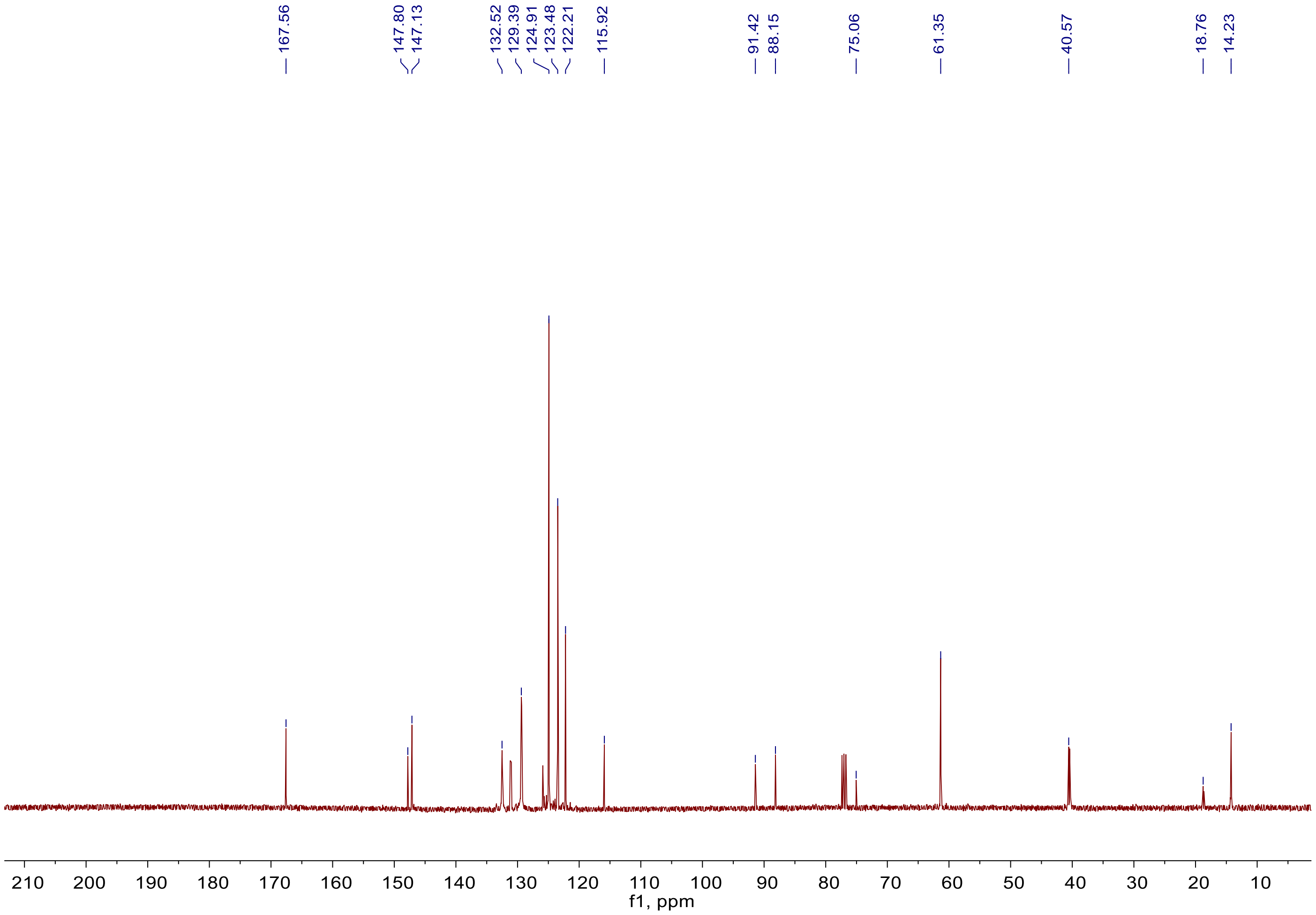

^{13}C spectrum of **4g** in $CDCl_3$

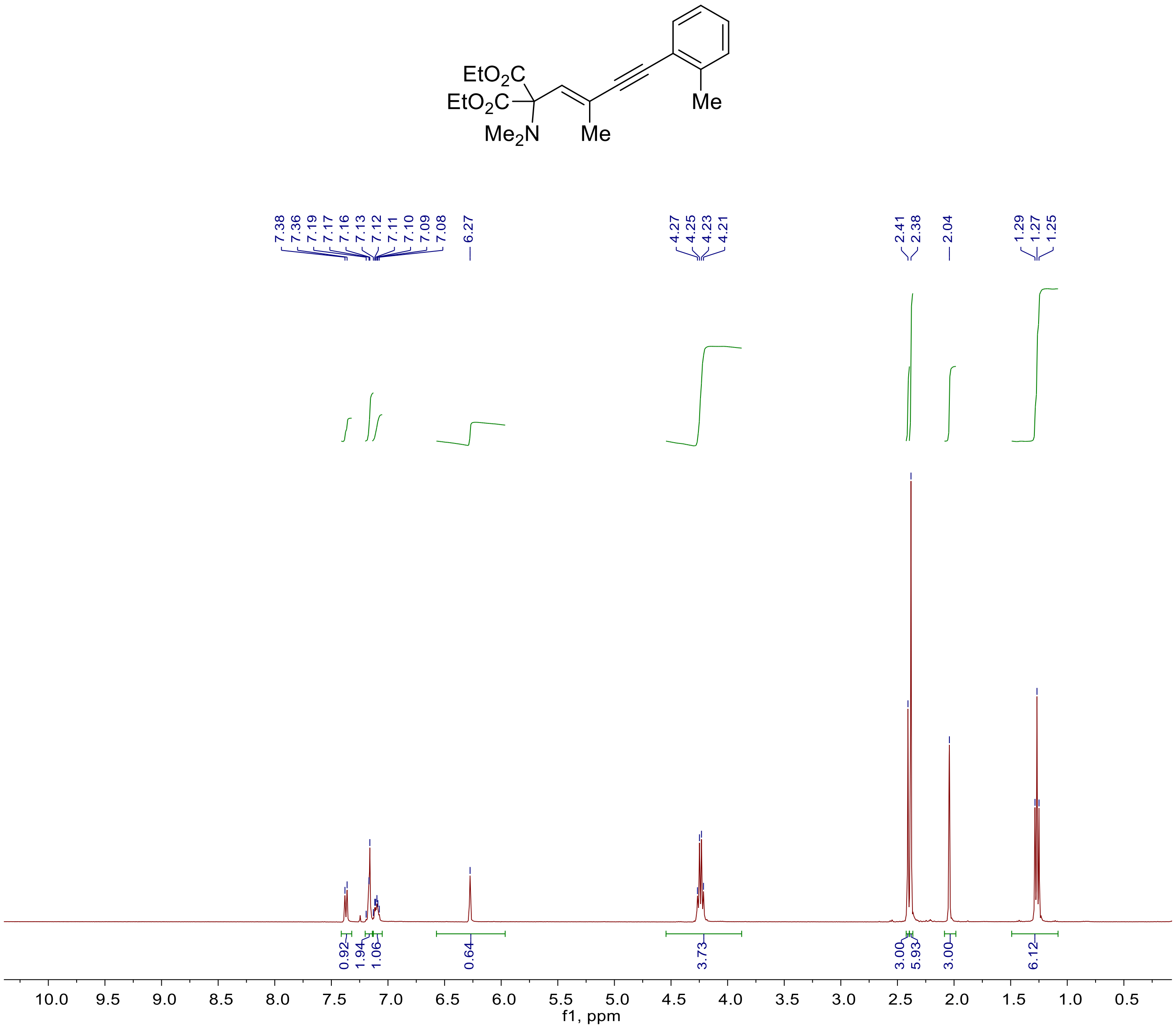

^{1}H spectrum of **4h** in $CDCl_3$

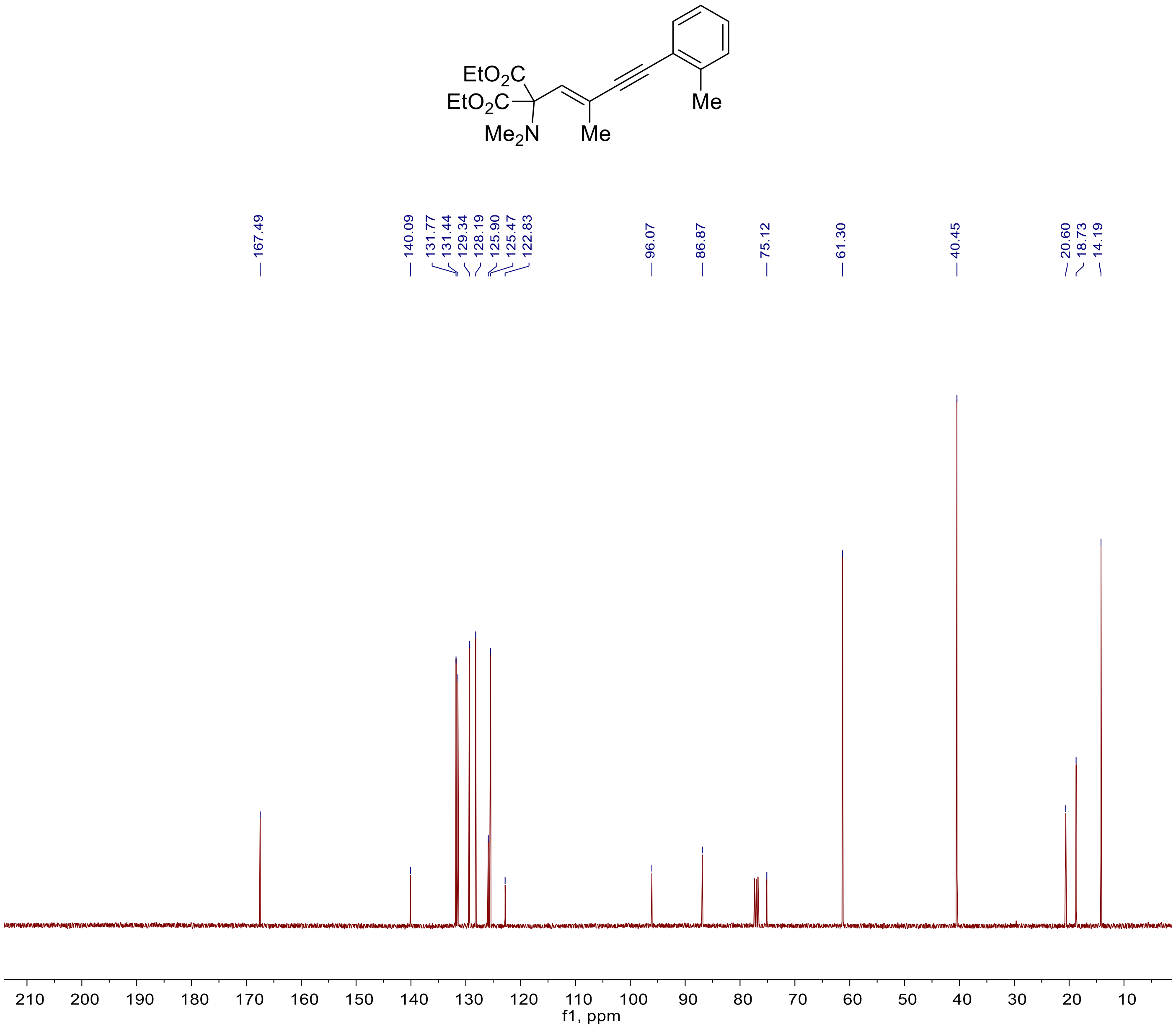

^{13}C spectrum of **4h** in $CDCl_3$

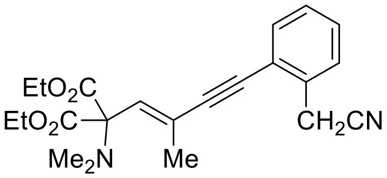

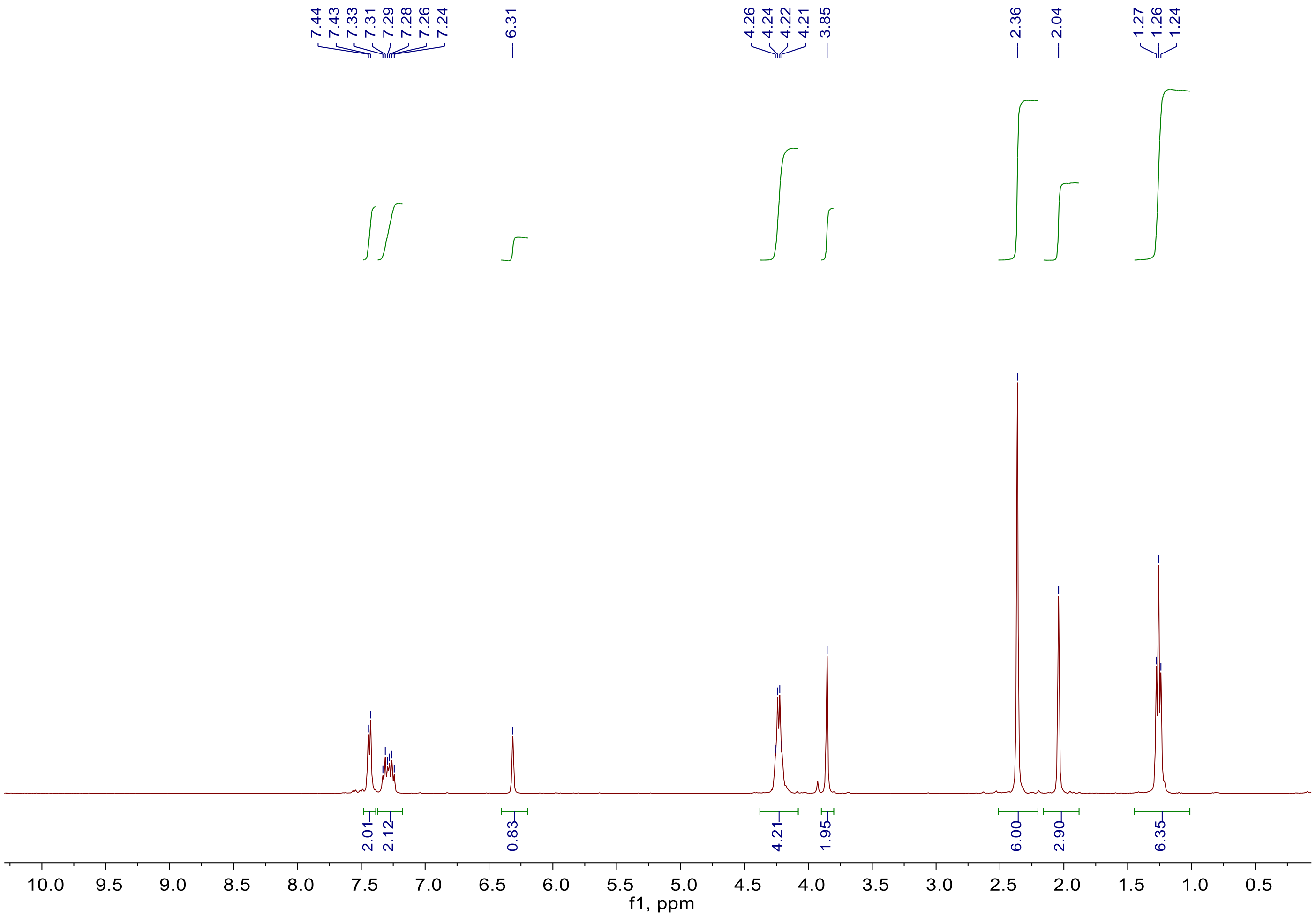

^{1}H spectrum of **4i** in $CDCl_3$

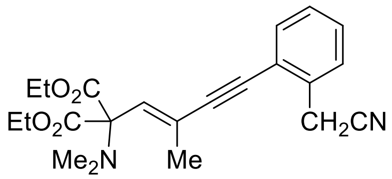

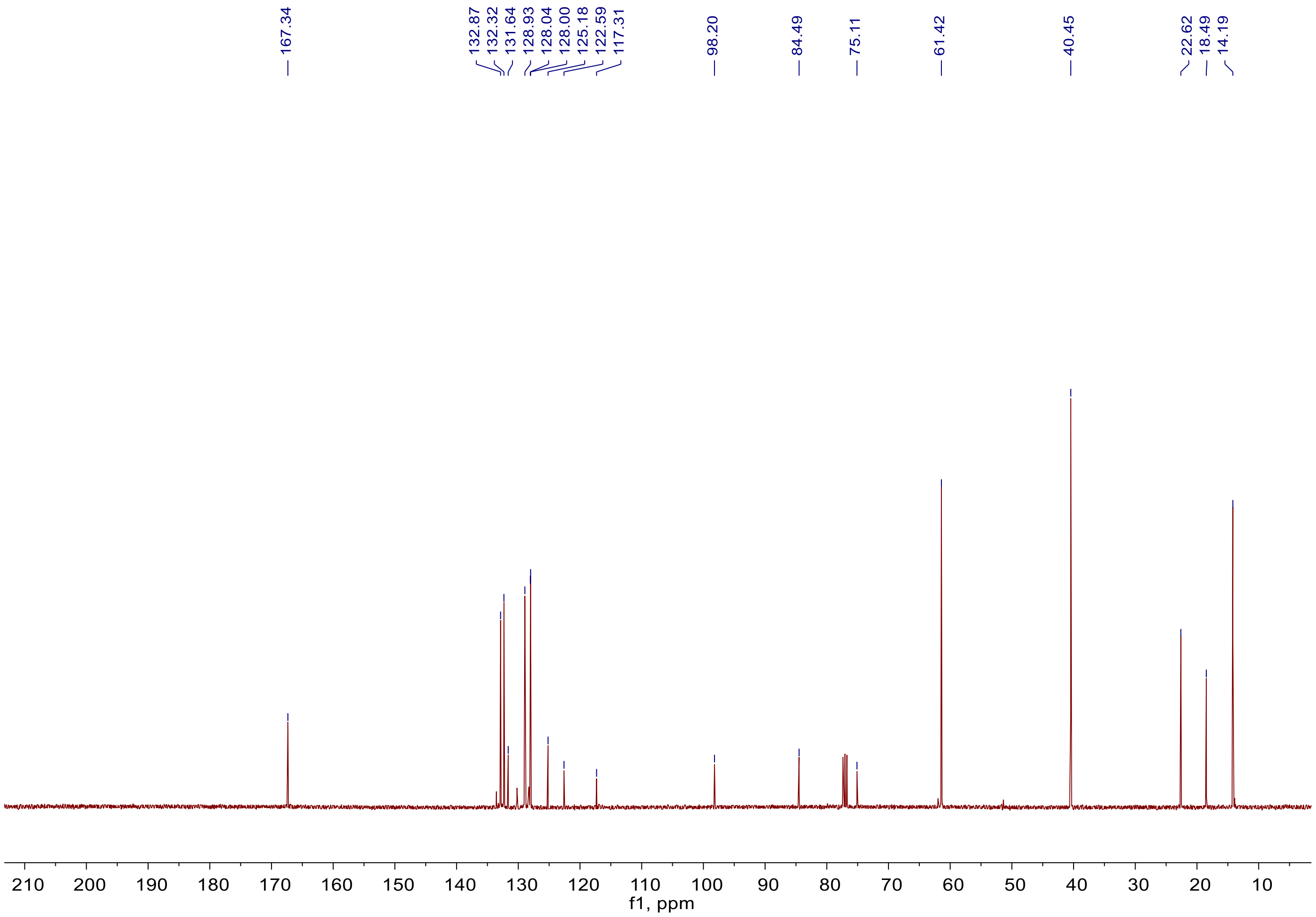

^{13}C spectrum of **4i** in $CDCl_3$

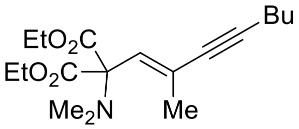

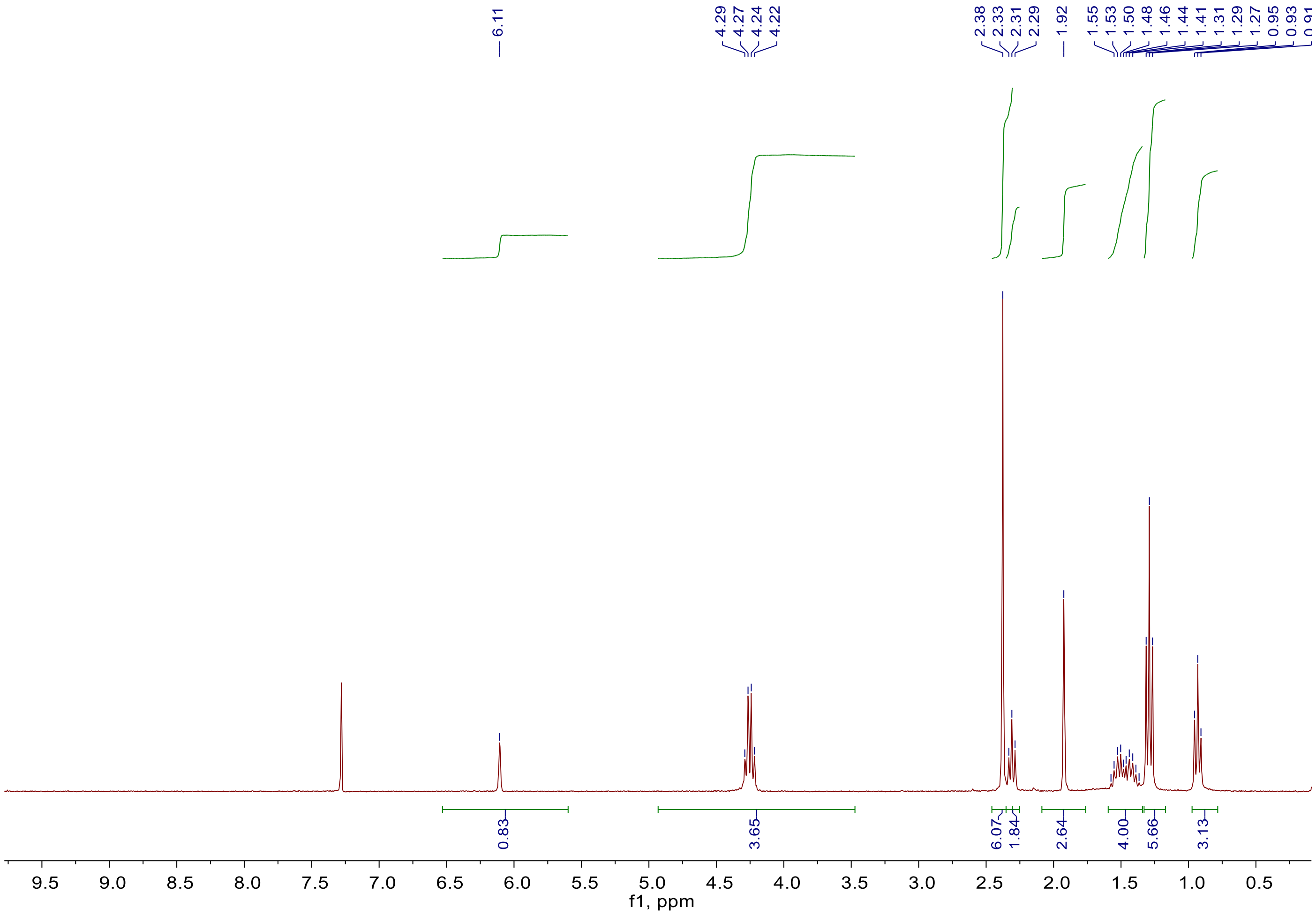

^{1}H spectrum of **4j** in $CDCl_3$

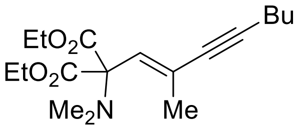

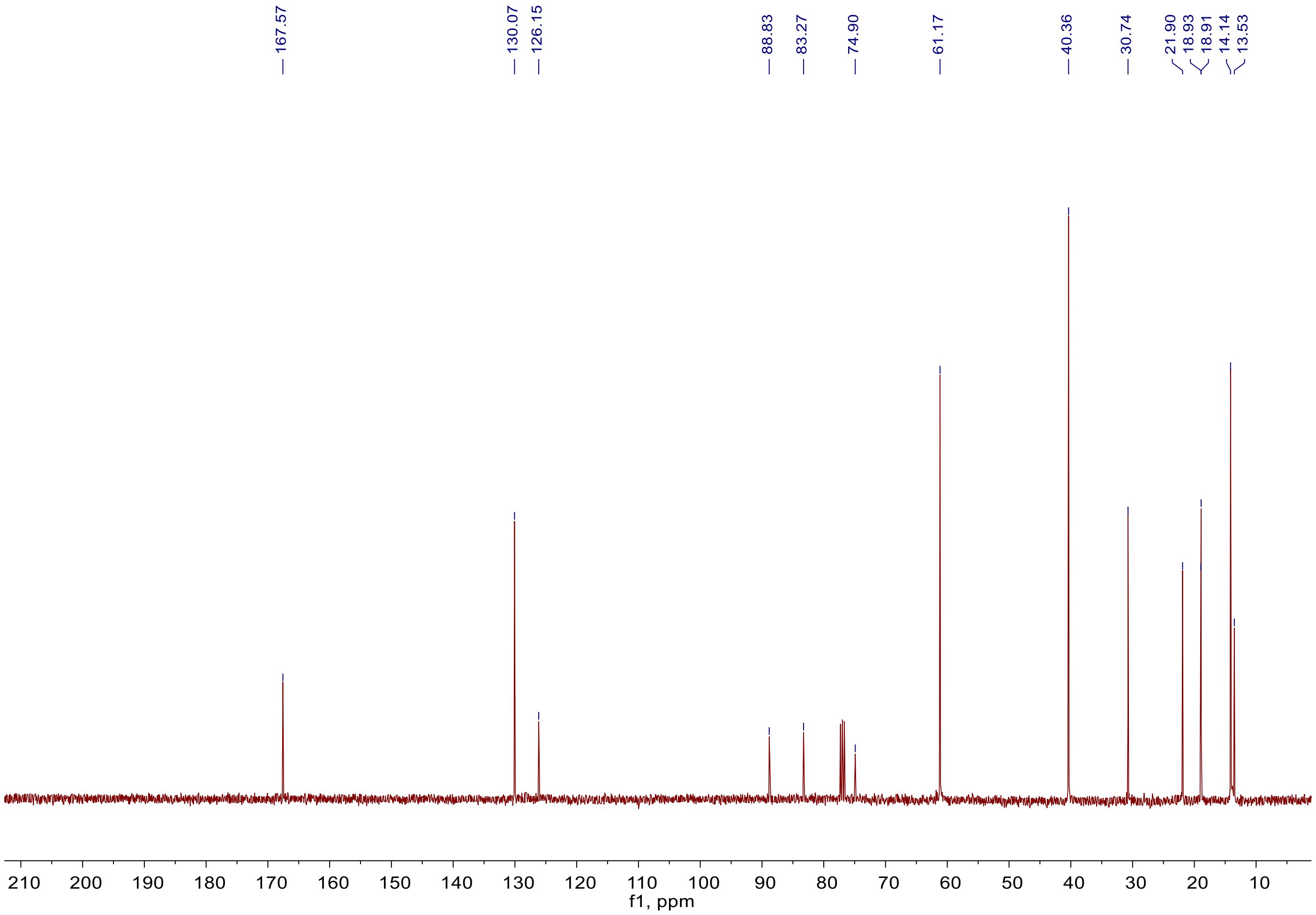

^{13}C spectrum of **4j** in $CDCl_3$

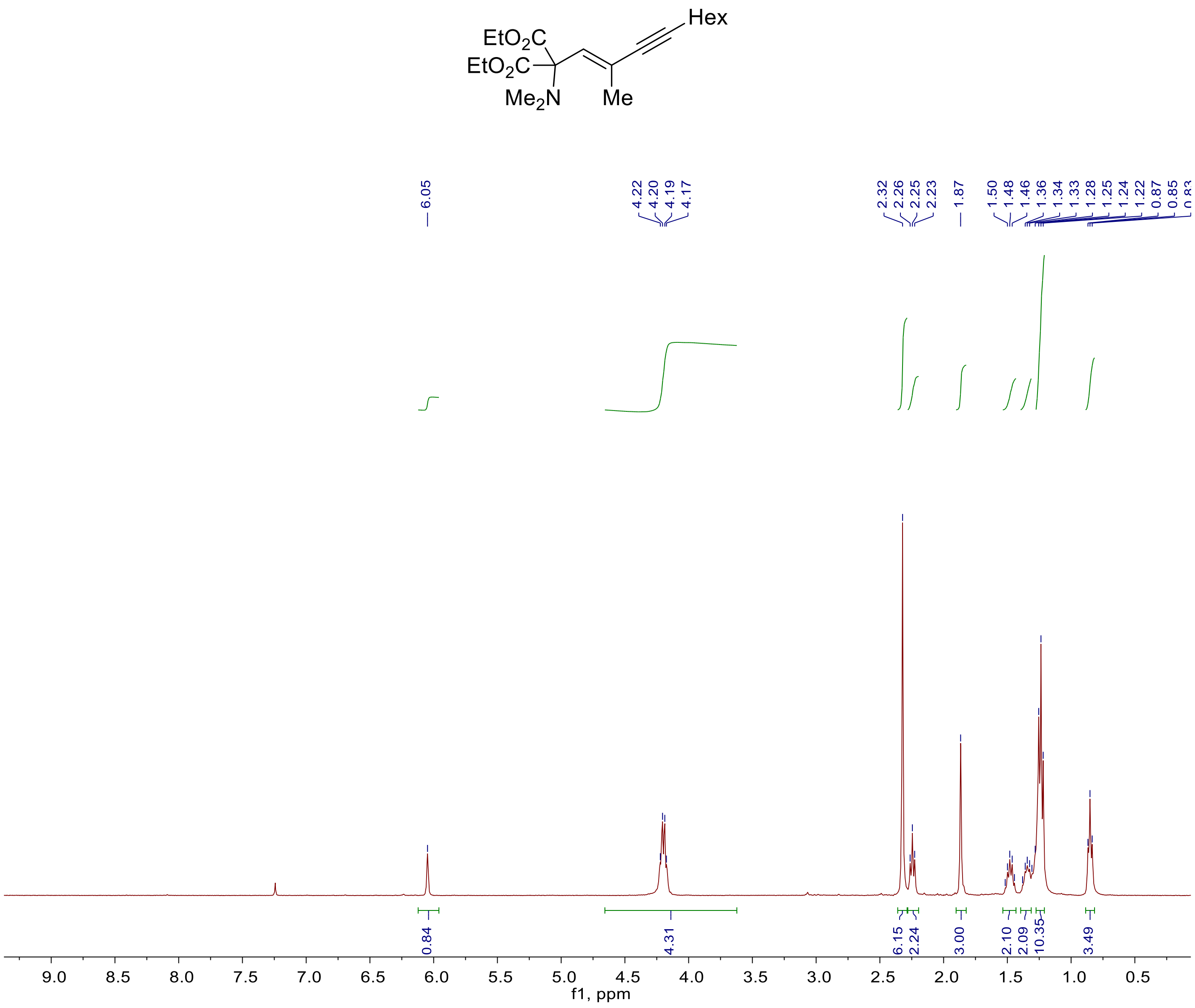

^{1}H spectrum of **4k** in $CDCl_3$

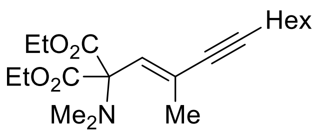

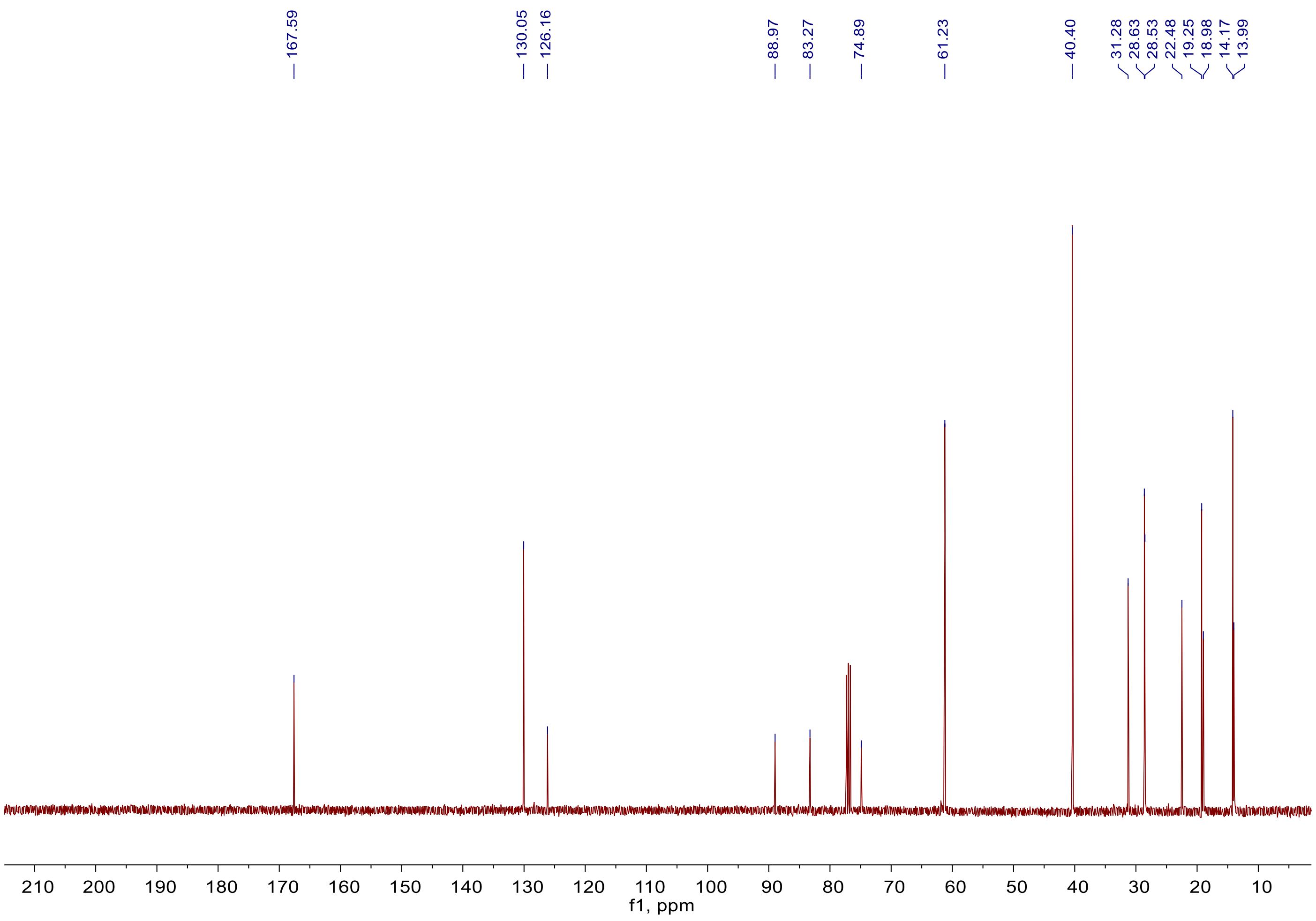

^{13}C spectrum of **4k** in $CDCl_3$

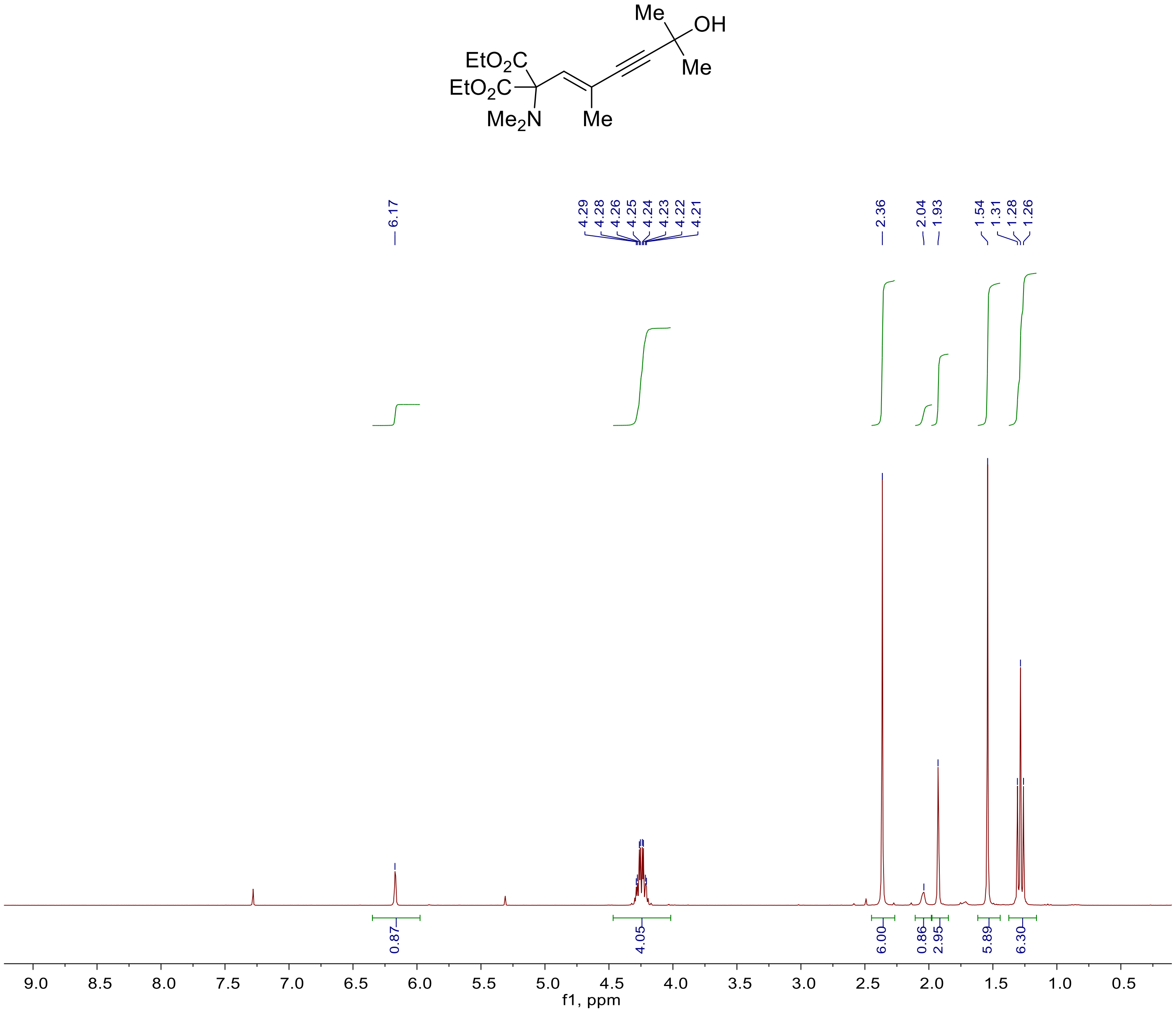

^{1}H spectrum of **4l** in $CDCl_3$

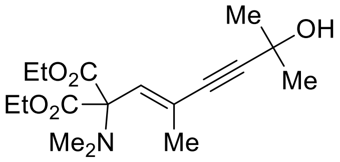

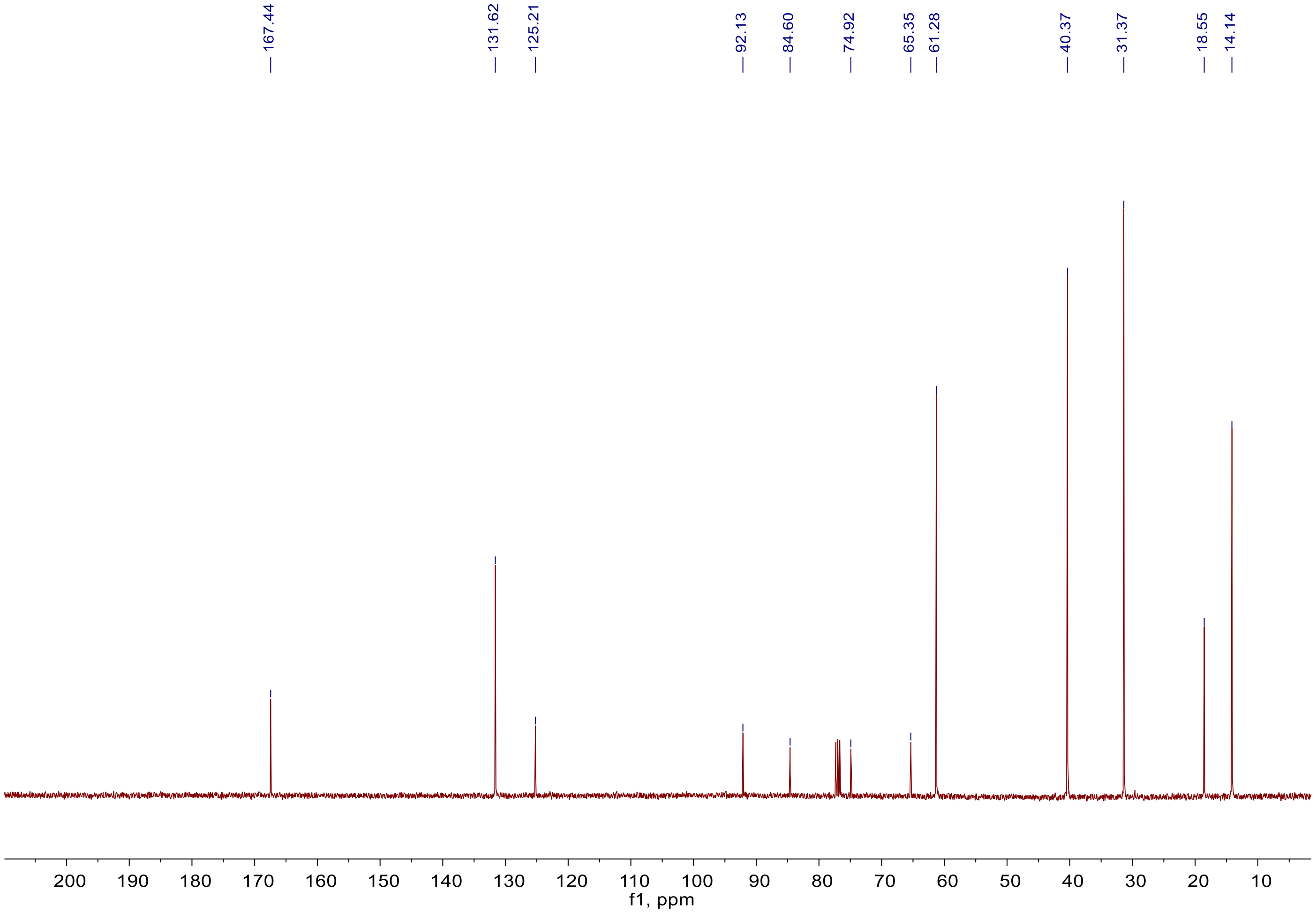

^{13}C spectrum of **4l** in $CDCl_3$

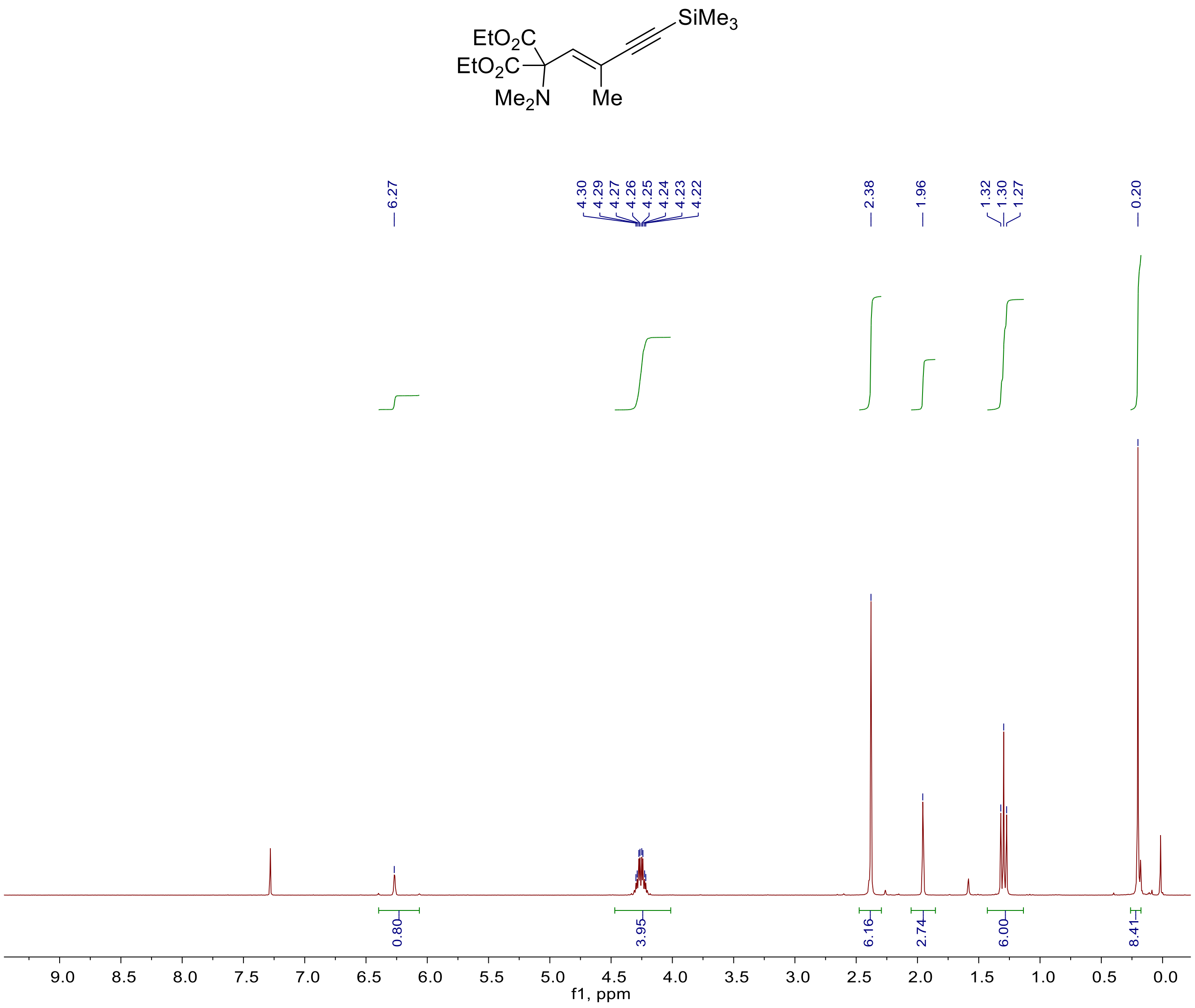

^{1}H spectrum of **4m** in $CDCl_3$

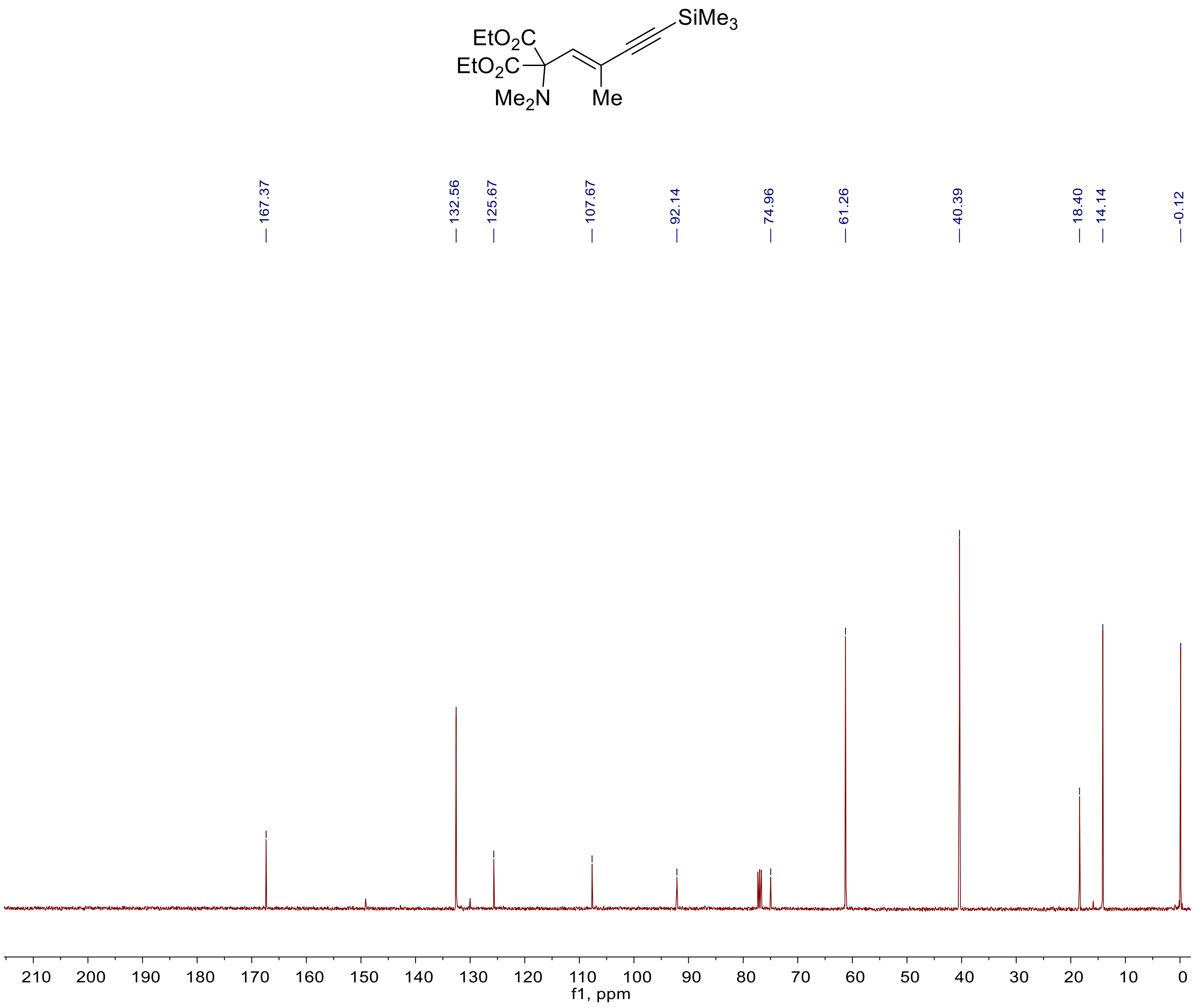

^{13}C spectrum of **4m** in $CDCl_3$

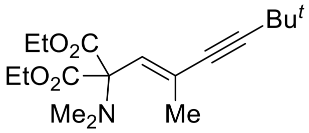

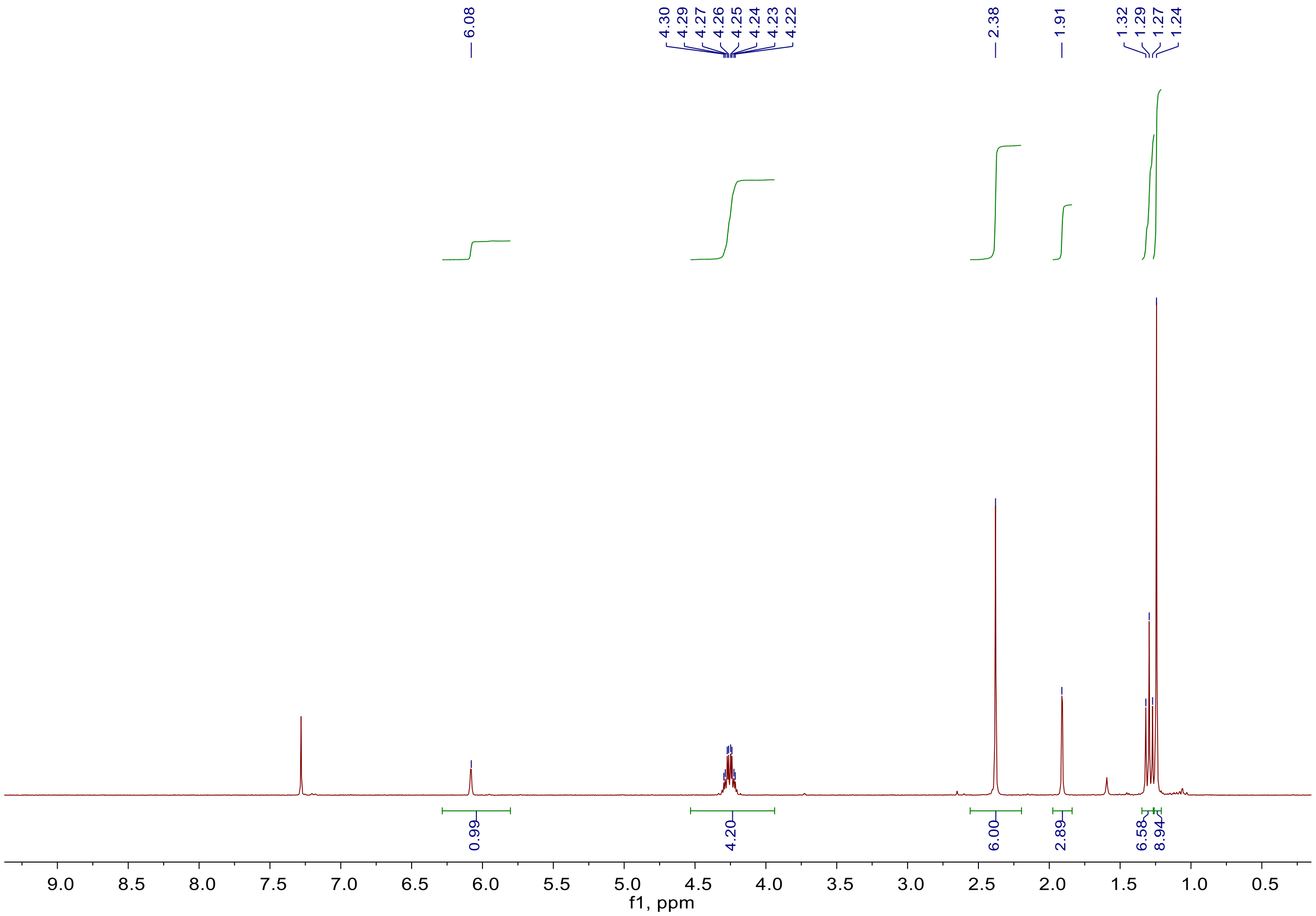

^{1}H spectrum of **4n** in $CDCl_3$

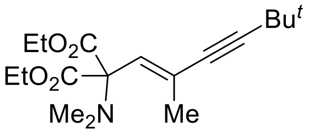

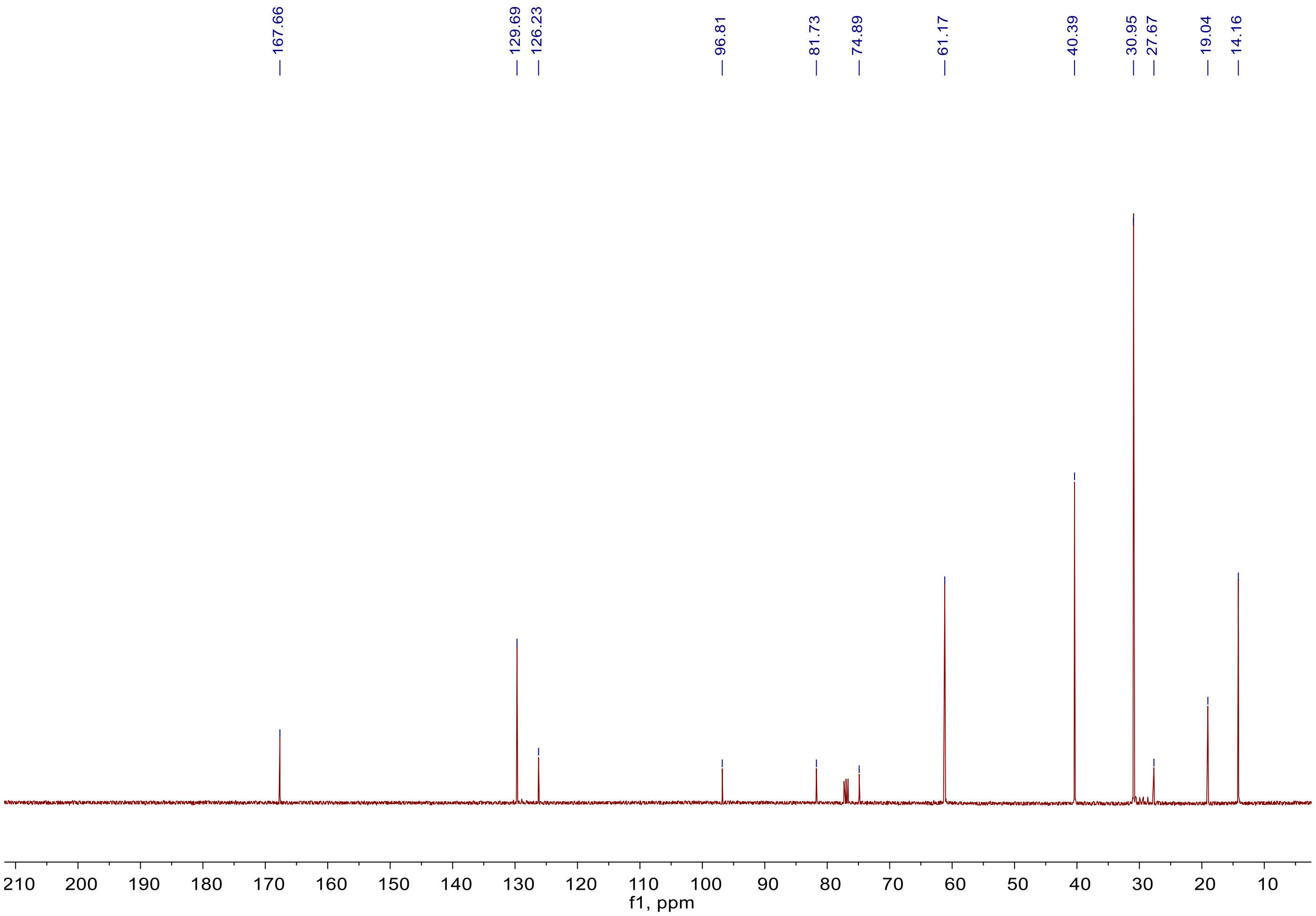

^{13}C spectrum of **4n** in $CDCl_3$

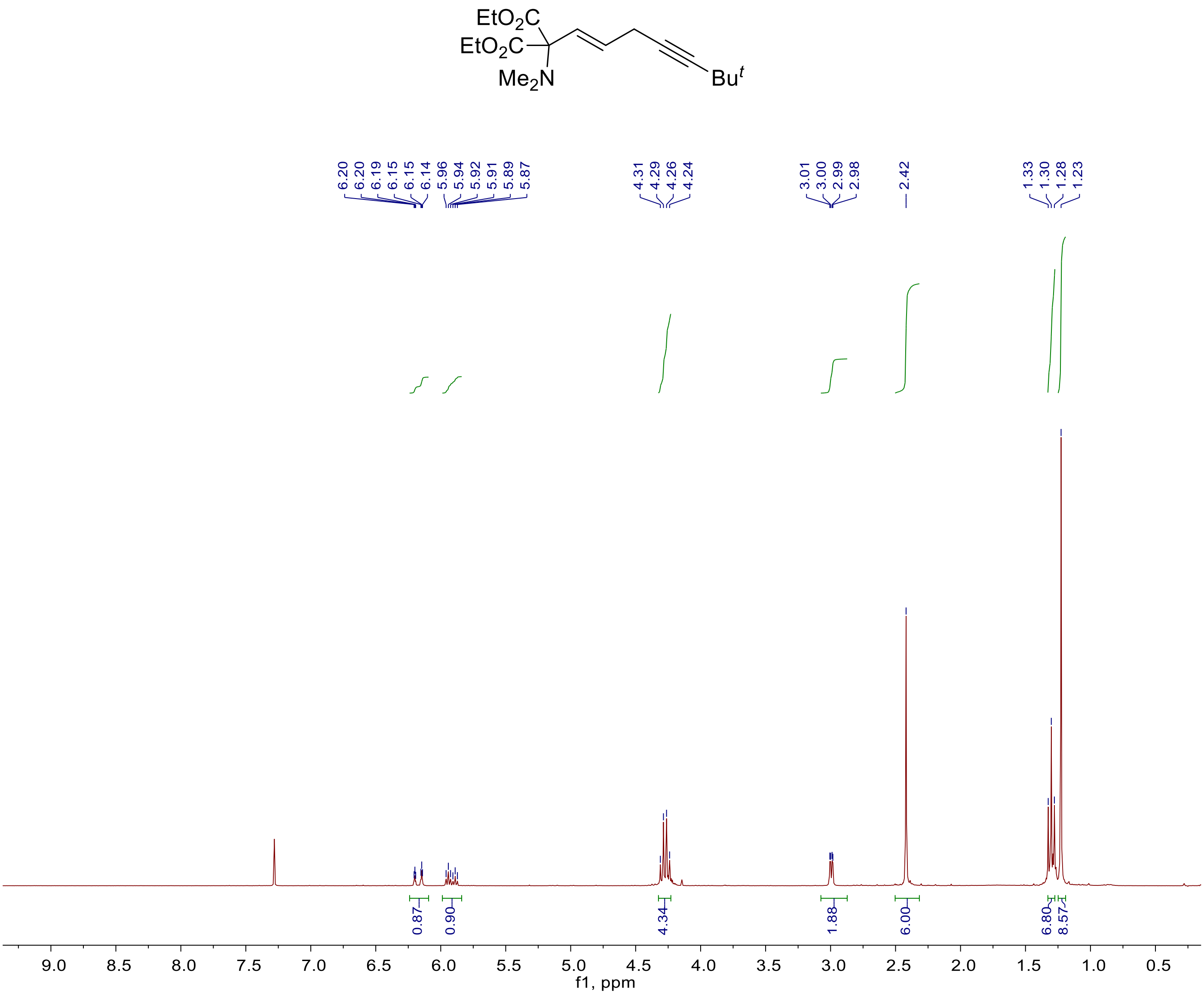

^{1}H spectrum of **4n’** in $CDCl_3$

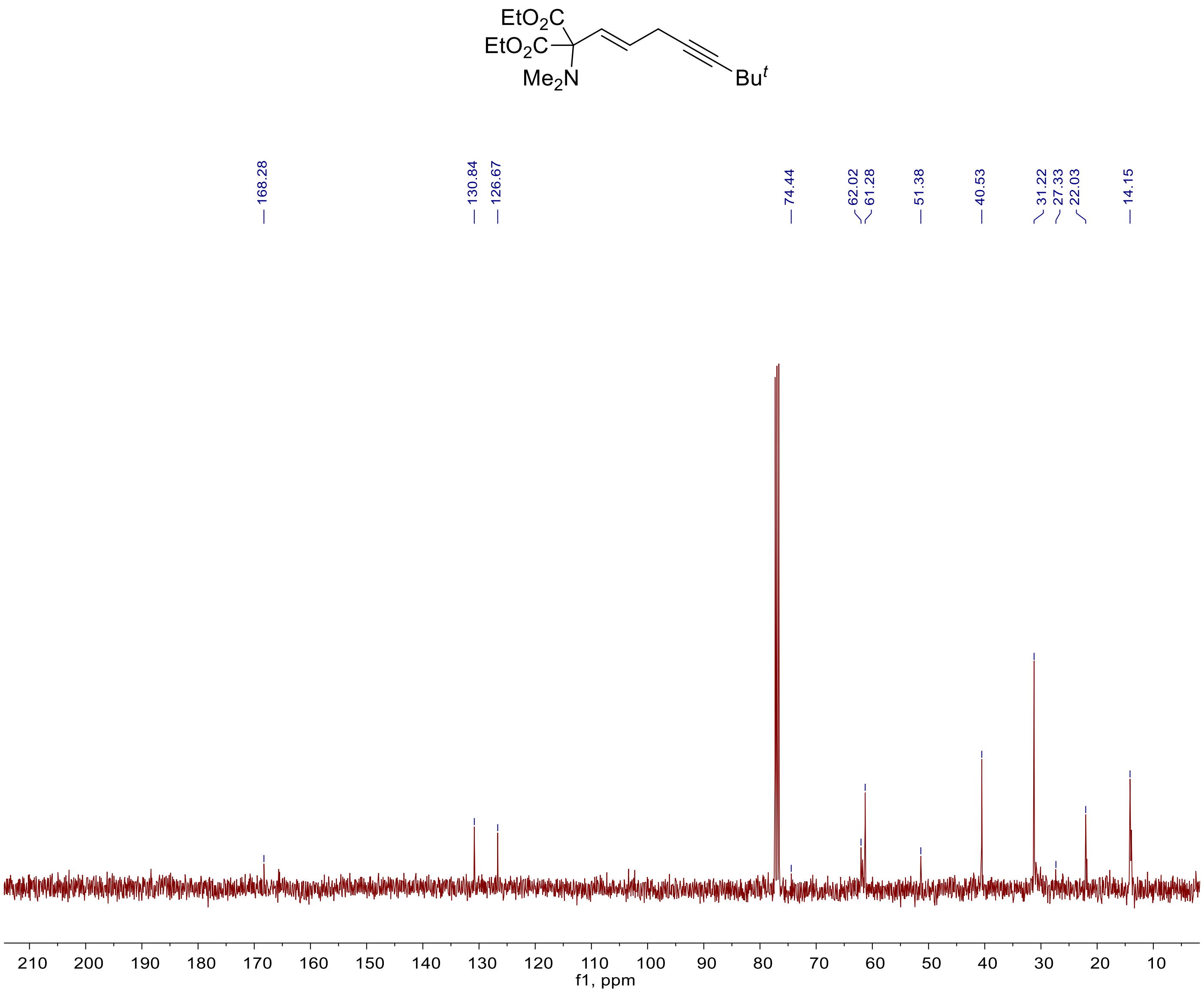

^{13}C spectrum of **4n’** in $CDCl_3$

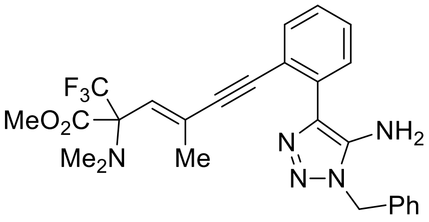

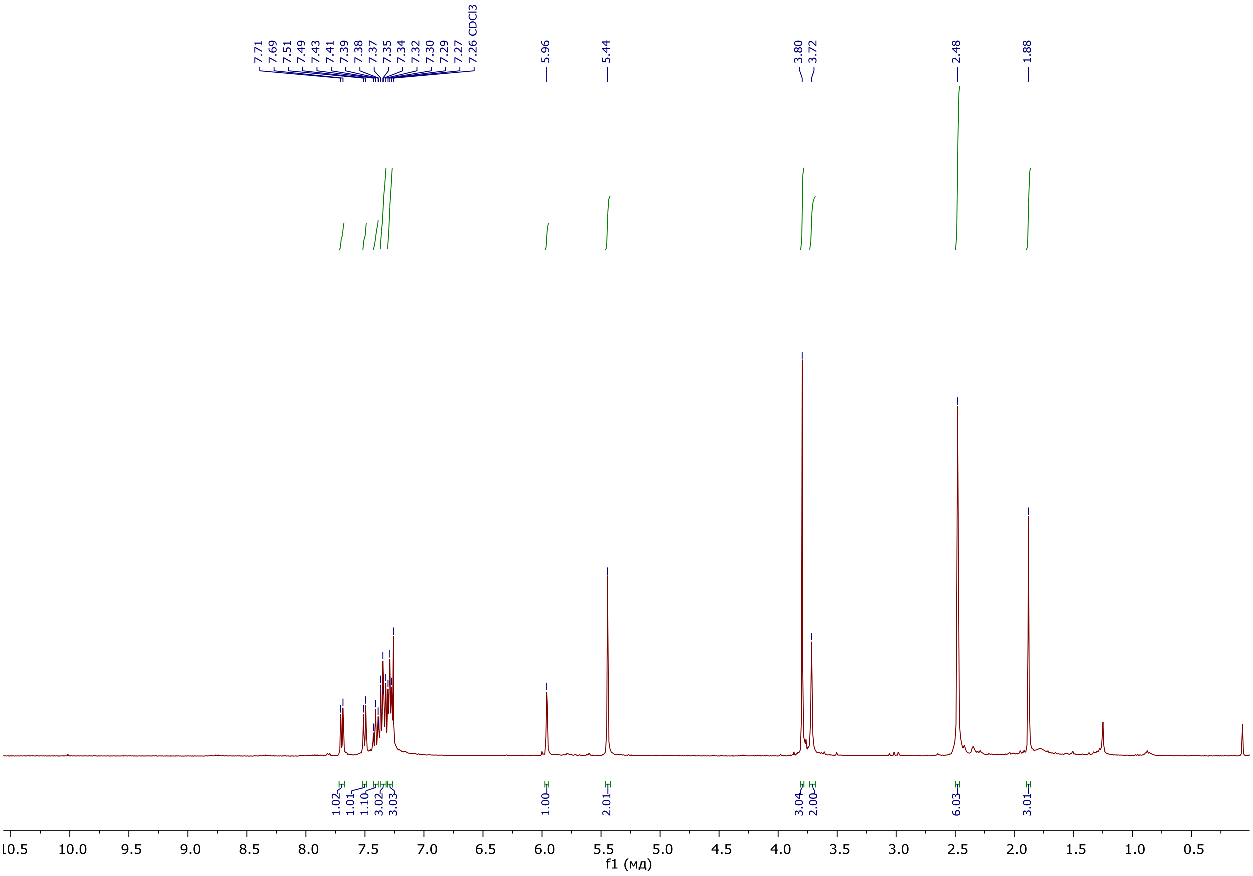

^{1}H spectrum of **5a** in $CDCl_3$

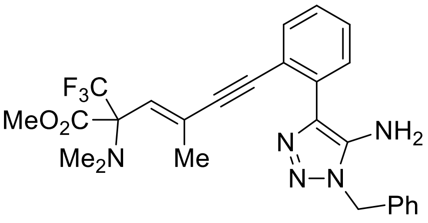

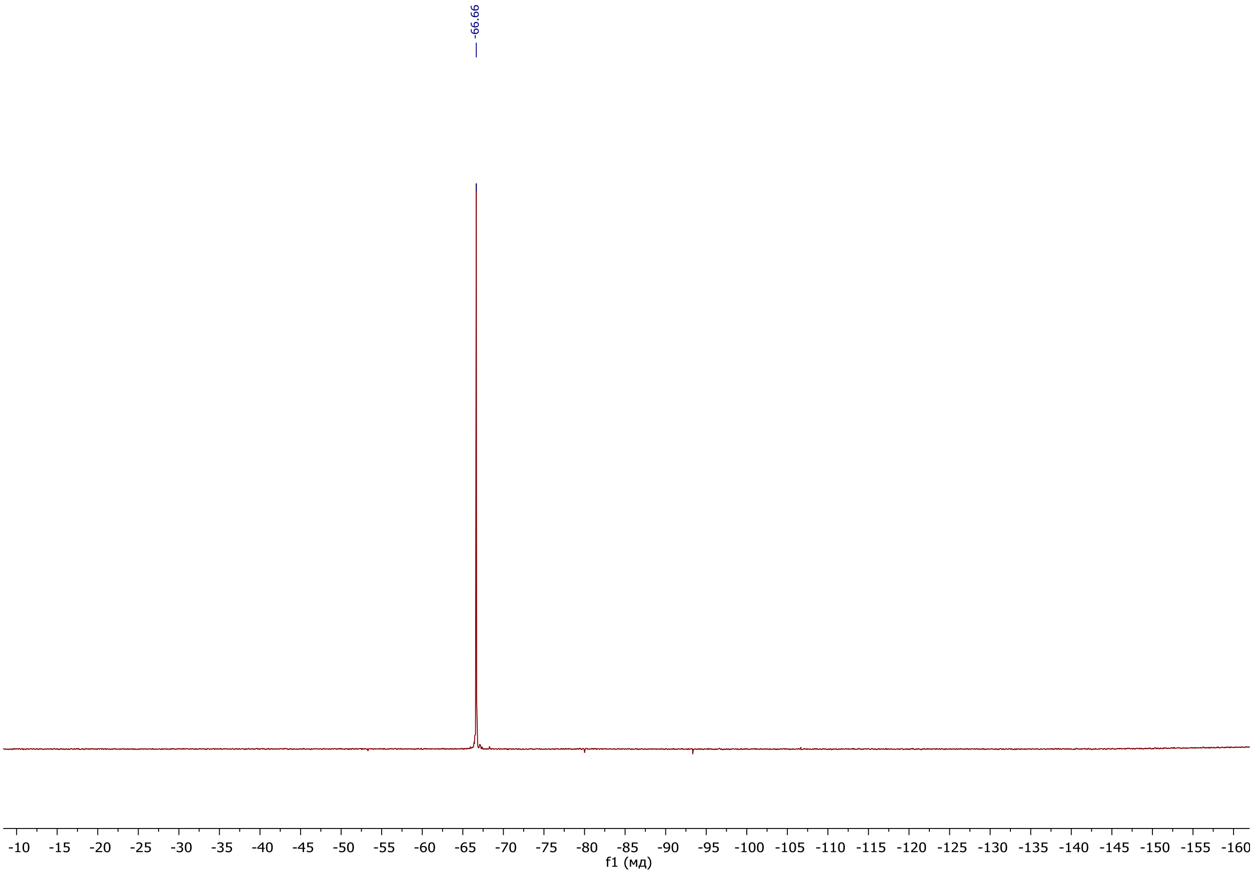

^{19}F spectrum of **5a** in $CDCl_3$

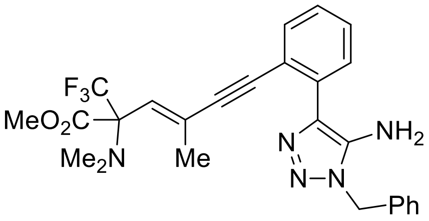

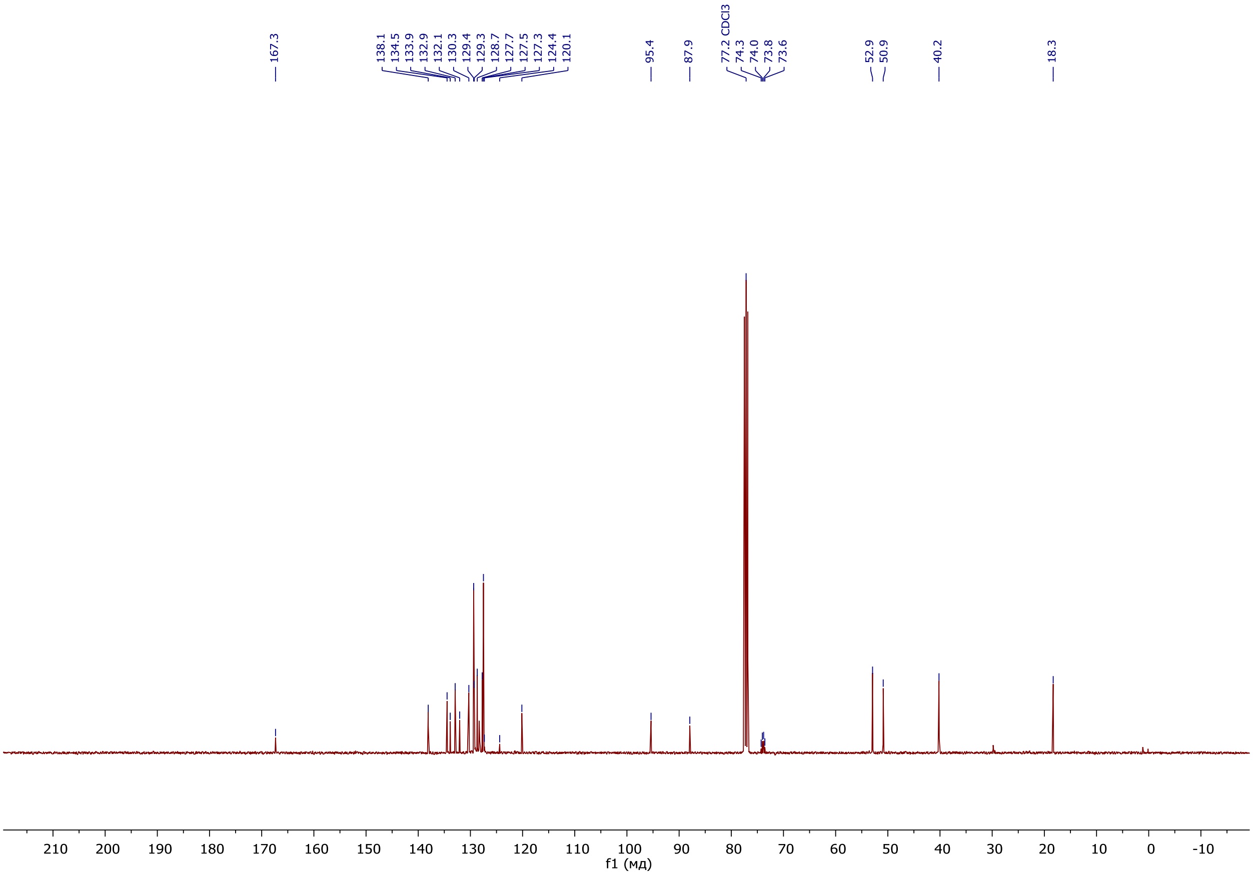

^{13}C spectrum of **5a** in $CDCl_3$

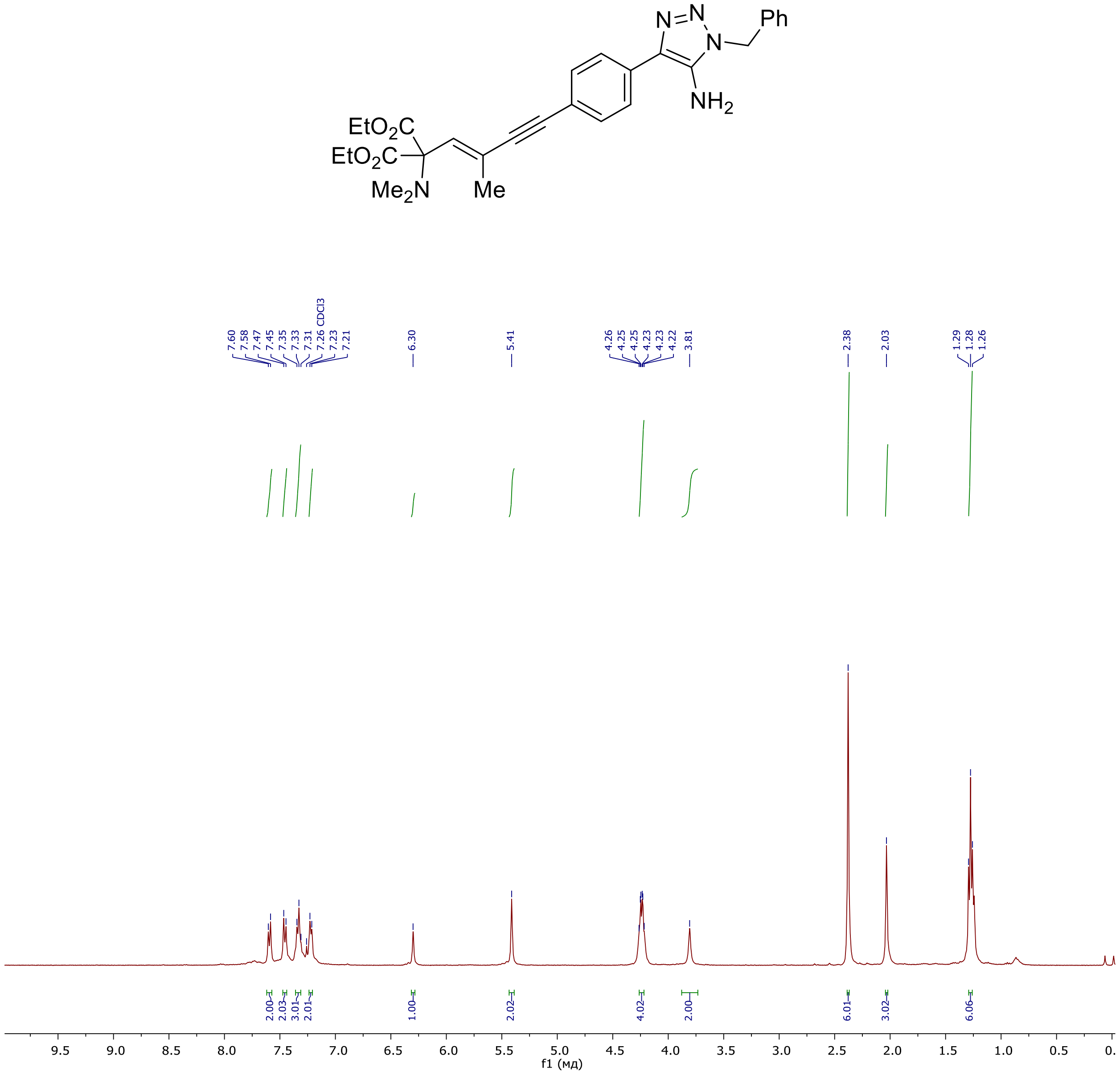

^1H spectrum of **5b** in $CDCl_3$

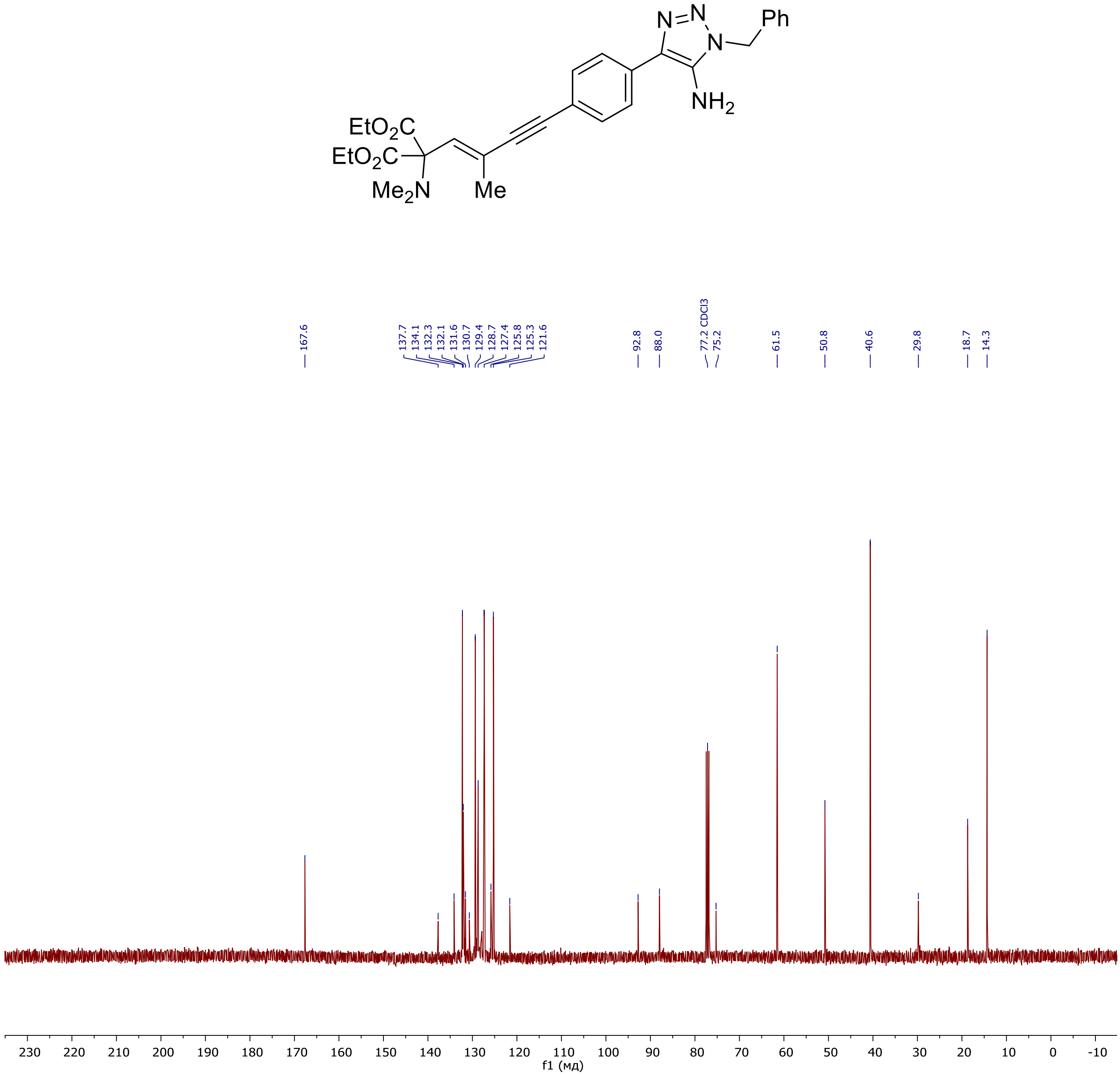

^{13}C spectrum of **5b** in $CDCl_3$

2D $^{1}H^{19}F$-HOESY NMR Spectrum for 3a

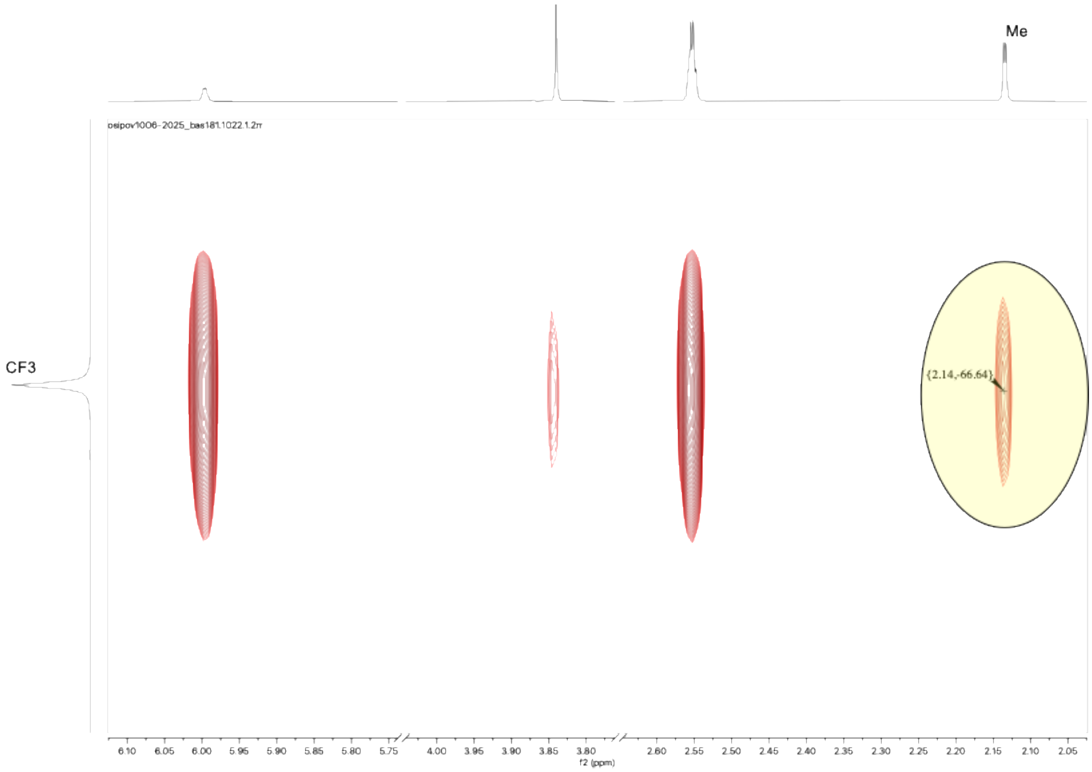

Supplement: Supplementary file 1 [file molecules-30-03623-s001.zip › molecules-3829144-supplementary.pdf]
